# Galaxy as a gateway to bioinformatics: Multi-Interface Galaxy Hands-on Training Suite (MIGHTS) for scRNA-seq

**DOI:** 10.1093/gigascience/giae107

**Published:** 2025-01-08

**Authors:** Camila L Goclowski, Julia Jakiela, Tyler Collins, Saskia Hiltemann, Morgan Howells, Marisa Loach, Jonathan Manning, Pablo Moreno, Alex Ostrovsky, Helena Rasche, Mehmet Tekman, Graeme Tyson, Pavankumar Videm, Wendi Bacon

**Affiliations:** Eccles Institute of Human Genetics, University of Utah, Salt Lake City, UT, 84112, USA; School of Chemistry, University of Edinburgh, Edinburgh, EH9 3FJ, UK; Department of Computer Science, John Hopkins Medical Institution, Baltimore, MD, 21224, USA; Erasmus Medical Center, Rotterdam, Zuid-Holland, 3015 GD, Netherlands; School of Computing & Communications, The Open University, Milton Keynes, Buckinghamshire, MK7 6AA, UK; School of Life, Health & Chemical Sciences, The Open University, Milton Keynes, Buckinghamshire, MK7 6AA, UK; European Bioinformatics Institute, European Molecular Biology Laboratory, Hinxton, CB10 1SD, UK; Early Computational Oncology, AstraZeneca, Cambridge, CB2 0AA, UK; Department of Computer Science, John Hopkins Medical Institution, Baltimore, MD, 21224, USA; Erasmus Medical Center, Rotterdam, Zuid-Holland, 3015 GD, Netherlands; Division of Pharmacology and Toxicology, University of Freiburg, Freiburg im Breisgau, Baden-Württemberg, 79098, Germany; School of Life, Health & Chemical Sciences, The Open University, Milton Keynes, Buckinghamshire, MK7 6AA, UK; Department of Computer Science, University of Freiburg, Freiburg im Breisgau,Baden-Württemberg, 79098, Germany; School of Life, Health & Chemical Sciences, The Open University, Milton Keynes, Buckinghamshire, MK7 6AA, UK

**Keywords:** training, STEM education, Galaxy project, single-cell RNA-seq analysis, scRNA-seq, bioinformatics, reproducibility, sustainability

## Abstract

**Background:**

Bioinformatics is fundamental to biomedical sciences, but its mastery presents a steep learning curve for bench biologists and clinicians. Learning to code while analyzing data is difficult. The curve may be flattened by separating these two aspects and providing intermediate steps for budding bioinformaticians. Single-cell analysis is in great demand from biologists and biomedical scientists, as evidenced by the proliferation of training events, materials, and collaborative global efforts like the Human Cell Atlas. However, iterative analyses lacking reinstantiation, coupled with unstandardized pipelines, have made effective single-cell training a moving target.

**Findings:**

To address these challenges, we present a Multi-Interface Galaxy Hands-on Training Suite (MIGHTS) for single-cell RNA sequencing (scRNA-seq) analysis, which offers parallel analytical methods using a graphical interface (buttons) or code. With clear, interoperable materials, MIGHTS facilitates smooth transitions between environments. Bridging the biologist–programmer gap, MIGHTS emphasizes interdisciplinary communication for effective learning at all levels. Real-world data analysis in MIGHTS promotes critical thinking and best practices, while FAIR data principles ensure validation of results. MIGHTS is freely available, hosted on the Galaxy Training Network, and leverages Galaxy interfaces for analyses in both settings. Given the ongoing popularity of Python-based (Scanpy) and R-based (Seurat & Monocle) scRNA-seq analyses, MIGHTS enables analyses using both.

**Conclusions:**

MIGHTS consists of 11 tutorials, including recordings, slide decks, and interactive visualizations, and a demonstrated track record of sustainability via regular updates and community collaborations. Parallel pathways in MIGHTS enable concurrent training of scientists at any programming level, addressing the heterogeneous needs of novice bioinformaticians.

## Introduction

Although bioinformatics is critical to basic biological and applied biomedical research, there remains a shortage of scientists with bioinformatics expertise [1]. As computational domains of biology continue to grow, bioinformatics play an important role in groundbreaking discoveries [2–5]. Thinking computationally about biological processes has been shown to produce more accurate models [6] and enhance problem-solving [7]. However, it is important to note that bioinformatics often requires many expensive resources, such as computational infrastructure, maintenance, and training [8]. Financial barriers can limit access to training and research [9–13]. As such, many bioinformaticians rarely receive formal training in the field [8], and teaching bioinformatics is notably difficult.

Integrating bioinformatics into undergraduate curricula may address this gap [1, 14]. Bioinformatics has been introduced in high schools, where it was shown to improve awareness, engagement, and self-efficacy of students, leading to increased interest in STEM careers [15]. Pharmaceutical companies need biomedical analysts [16], most employers in the life sciences prefer some competency in software analyses [17], and the use of bioinformatic analyses to characterize novel cell types and lineages [18] has surged. In response, institutes are beginning to teach foundational computing skills to biologists [14, 19].

Materials that focus on problem-solving, interactivity, and cooperative learning have demonstrated enhanced learning outcomes [20], and bioinformatics has effectively been taught by emphasizing interdisciplinary problem-solving [21]. To standardize training, a list of “rules” was identified to teach scientists to program: beginning with the end in mind, taking small steps forward, and focusing on individual tasks [20]. The “end in mind” requires domain-specific understanding (i.e., identifying cell types via marker genes) while the individual tasks require programming skills (R, Python, troubleshooting, etc.). This duality forces new biofinformaticians to learn and apply 2 new skill sets simultaneously [22–24]. The need to embed computing into science is not novel [25], but blending skills across disciplines is not without challenges [26].

The Galaxy Training Network (GTN) boasts tutorials for analysis across a range of fields, all publicly available and accessible by URL [27]. The GTN provides free training infrastructure to fast-track trainees via live courses in which trainers are available to monitor and assist participants [28]. This supports all, but especially low-resource institutions’, engagement with bioinformatic training and has additionally been tested for native Spanish speakers [28]. Integrating these free resources into undergraduate curricula has been successful [27], as training materials include interactive features based on research-backed pedagogies. Separation of learning components has previously been suggested as an effective method [29] but raises the question: how can coding and complex bioinformatic analyses be isolated from one another?

Here, we directly address the need to separate the two for training. Leveraging the Galaxy Graphical User Interface (GUI) and the GTN, we present the Multi-Interface Galaxy Hands-on Training Suite (MIGHTS): a single-cell RNA sequencing (scRNA-seq) tutorial suite enabling a smooth transition from data analysis in a button-based, user-friendly environment [30] to a more advanced, flexible programming environment. Using a sample dataset, MIGHTS guides users through the steps necessary to accomplish commonly published scRNA-seq analyses and visualizations, including generating a matrix, combining datasets, filtering, plotting, and general exploration of the data, as well as trajectory inference of a dataset known to represent a developmental spectrum. The sample dataset provided for use with the suite reveals a delay in thymic maturation in growth-restricted neonatal mice [31]. MIGHTS offers multiple routes of scRNA-seq analysis, allowing a button-based or coding-based version of the same commonly published workflows. Notably, MIGHTS offers opportunities for a heterogeneous student population ranging from programming-friendly to programming-fearful, expanding access to critical skills required for effective bioinformatic analyses and biomedical and life science research.

## Methods

### Multienvironment

MIGHTS consists of 11 tutorials: 6 button-based (BB) and 5 in a programming environment (PE) each making use of common analysis packages (Table 1).

**Table 1:** MIGHTS tutorials with used packages and data types

Analysis	Environment | tutorial (Package/Language)	Packages	Data types
**Preprocessing**	BB | Generating a single-cell matrix using Alevin	Salmon [32] with Alevin [33]	FASTQ
	BB | Combining single-cell datasets after preprocessing	dropletUtils [34, 35] (emptyDrops [34])	FASTA
	PE | Generating a single-cell matrix using Alevin and combining datasets (bash + R [36])	atlas-gene-annotation-manipulation [37]	GTF
		tximeta [38] (PE)	SingleCellExperiment Object
		biomaRt [39, 40] (PE)	SummarizedExperiment; AnnData
**Plotting and interpretation**	BB | Filter, plot, and explore single-cell RNA-seq data (Scanpy)	Scanpy [41]	AnnData
	PE | Filter, plot, and explore single-cell RNA-seq data (Scanpy, Python [42])	igraph [43] (PE)	
		louvain [44] (PE)	
		pandas [45] (PE)	
	BB | Filter, plot, and explore single-cell RNA-seq data (Seurat)	Seurat [46–50]	AnnData (for conversion to Seurat)
	PE | Filter, plot, and explore single-cell RNA-seq data (Seurat, R)	Matrix [51] (PE)	Seurat Object
		dplyr [52] (PE)	
**Trajectories**	BB | Inferring single-cell trajectories (Scanpy)	Scanpy [41]	AnnData
	PE | Inferring single-cell trajectories (Scanpy, Python)	fa2 [53] (PE)	
		igraph [43] (PE)	
		louvain [44] (PE)	
		numpy [54] (PE)	
		matplotlib [55] (PE)	
	BB | Inferring single-cell trajectories (Monocle3)	Monocle [56]	Cell Data Set
	PE | Inferring single-cell trajectories (Monocle3, R)	anndata [57] (PE)	AnnData (for conversion to Cell Data Set)
		viridislite [58] (PE)	
		magrittr [59] (PE)	
		Rcpp [60] (PE)	
		biomaRt [39, 40] (PE)	

**Figure 1:**
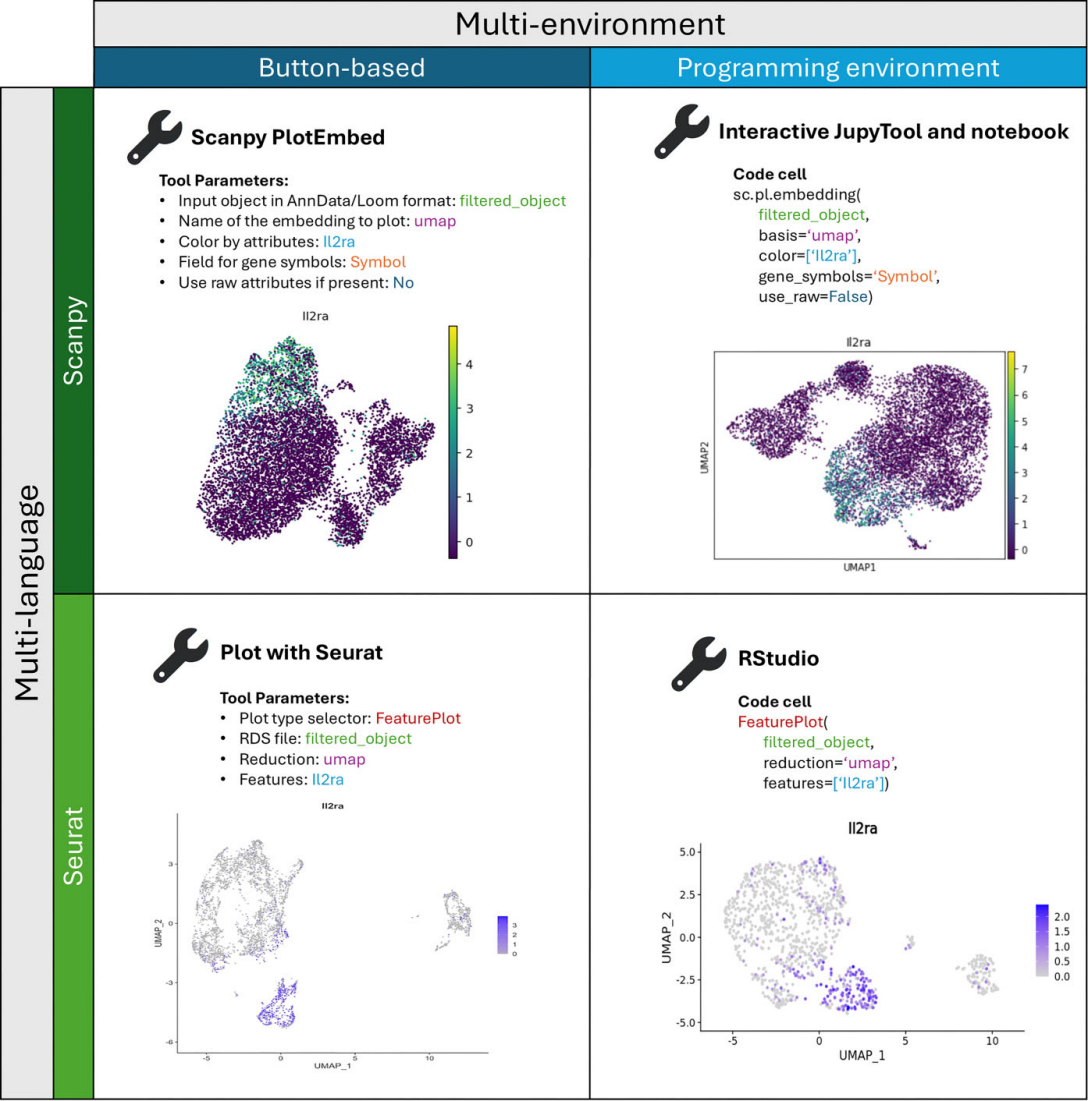
Performing the same step (plotting a marker gene: Il2ra on UMAP embedding) using Galaxy GUI and programming environment with both Python-based Scanpy and R-based Seurat packages. The resulting plots, although slightly different, represent the same biological information, no matter if the BB or PE method was used.

The Galaxy GUI features “click-to-run” buttons that execute programming functions [30].Users select andset parameters from drop-down lists and input boxes (Fig. 1, left column: button-based). Each tool includes help text to guide users anddescribe the flexibility ofthe tool’s function.

Galaxy’s interactive programming environments [61] are where the PE tutorials take place. Tutorials may be downloaded as RMarkdown or Jupyter notebooks [62, 63], or users may copy, paste, and run each executable code-containing cell from the PE text (Fig. 1, right column: programming environment). Jupyter and RMarkdown notebooks may be exported at the conclusion of each coded tutorial for easy reference or repetition.

### Multilevel

**Figure 2:**
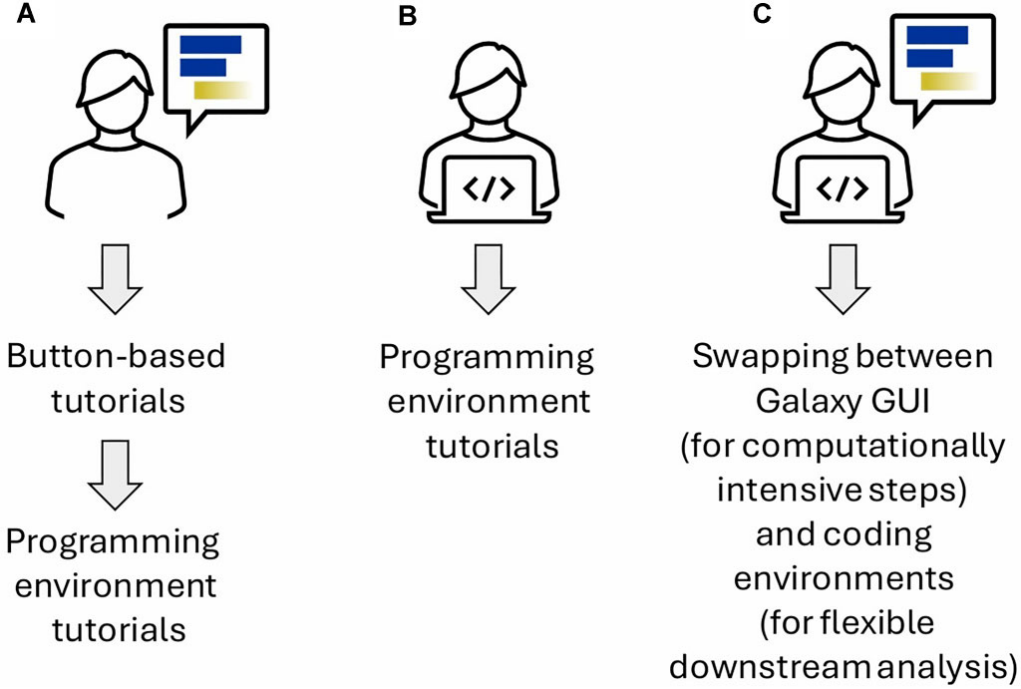
Representation of 3 possible user journeys using MIGHTS. (A) A beginner starting from button-based (BB) tutorials who can then move to the programming environment (PE). (B) An experienced programmer who can start the analysis directly from the PE, skipping introductory BB tutorials. (C) A skilled user who can optimize analyses by swapping between Galaxy GUI to perform computationally intensive steps and a programming environment for more flexible analyses.

MIGHTS caters to the following 3 learning pathways: BB to PE, straight to PE, and PE with BB (Fig. 2).

In the first case, BB tutorials guide beginners through the key steps of scRNA-seq analysis, establishing familiarity with the methods and learning to interpret results. Next, users may repeat the analysis in the PE, focusing on programming skills while becoming familiar with the languages and libraries commonly used for scRNA-seq analysis (Fig. 2A). If a user has experience programming and wants a more flexible analysis, they may begin with the PE tutorials, learning methods with more advanced functionality (Fig. 2B). Alternatively, experienced bioinformaticians may utilize Galaxy’s Interactive Environments to learn new analyses or run computationally demanding steps that they are unable to do locally (Fig. 2C).

### Multilanguage

**Figure 3:**
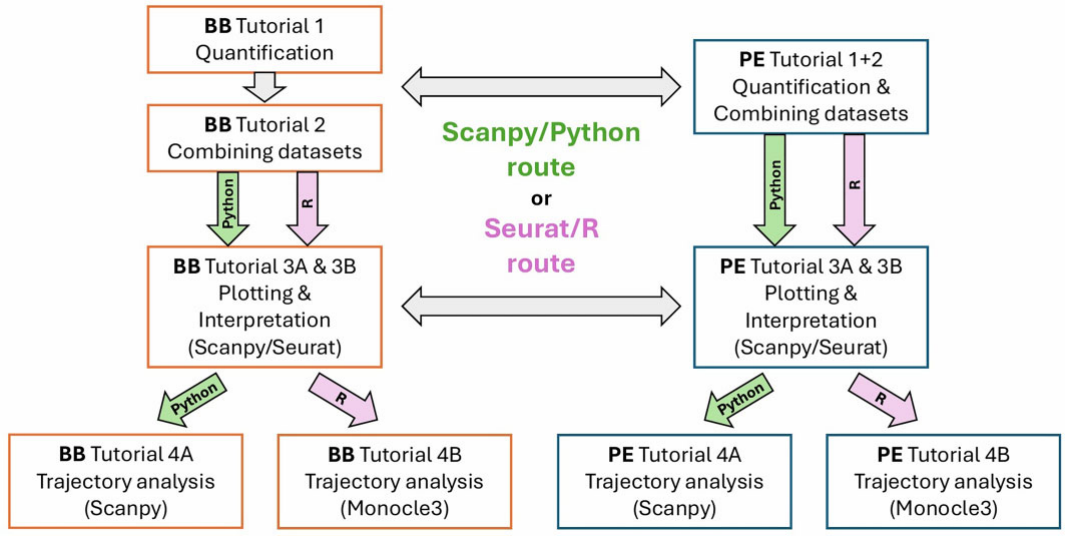
A diagram of the connections of tutorials. It highlights that the languages and packages used in BB and PE tutorials are consistent and allow moving between them easily.

scRNA-seq analysis is commonly performed in both R-based (using Seurat [46–50] & Monocle [56]) and Python-based (using Scanpy [41]) environments. Therefore, parallel analyses were created across BB and PE as well as across programming languages—effectively demonstrating multiple methods of analysis and data validation (Fig. 3). Users may conduct a typical, full scRNA-seq analysis workflow in R or Python in addition to on a GUI or in a PE.

### Research-relevant skills

MIGHTS demonstrates the use of many frequently used data types and packages for scRNA-seq analyses (Table 1), preparing users with research-relevant skills. Broadly applicable use of programming functions, algorithms, and troubleshooting lends itself to increased creativity and critical thinking [64, 65]. This also improves users’ employability and reaches scientists in various research groups, regardless of the method they prefer.

### Tutorials

Each tutorial begins with data import. The data used in MIGHTS come from a published study by Bacon et al. [31], describing a mouse model of fetal growth restriction that is publicly available from the EMBL-EBI ArrayExpress under accession number E-MTAB-6945 and may additionally be explored in the Single Cell Expression Atlas [66]. Tutorials in MIGHTS work with the same data throughout to demonstrate analyses using different methods and tools. Tutorials use real, uncurated data, which have simply been subsampled to enhance computational efficiency. The source data are the same, but each analysis allows import of a unique data file to start. Tutorials are designed to be completed in order but may be performed out of order—if a user wishes to learn how to cluster cells using Scanpy (RRID:SCR_018139), for example, they may select the dedicated tutorial and start with the provided, preprocessed file(s).

MIGHTS’s full workflow consists of three sequential analyses aligning with standard scRNA-seq pipelines [67] and allowing users to compare results across methods.

#### Generating a single-cell matrix using Alevin and combining datasets

**Figure 4:**
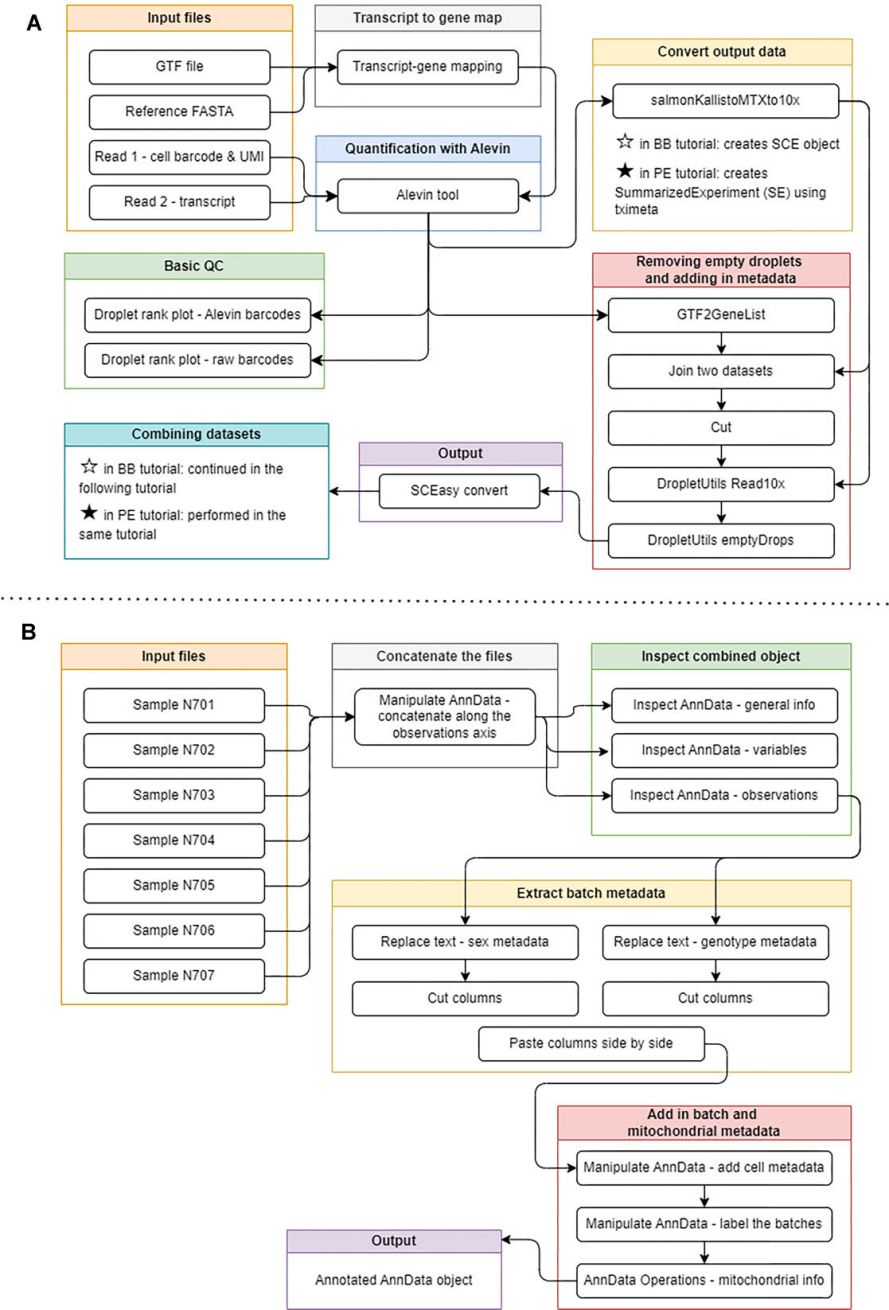
A diagram of workflows in the preprocessing tutorials. (A) Workflow for tutorial “Generating a single-cell matrix using Alevin.” Solid stars denote steps specific to the PE tutorial while unfilled stars represent BB-specific ones. (B) Workflow for tutorial “Combining single-cell datasets after pre-processing.” Both workflows featured in the BB tutorial are combined in the PE. A figure of an extracted Galaxy workflow is available in Supplementary Figs. S1 and S2.

The first two tutorials demonstrate the transformation of a FASTA sequencing file into a count matrix (Fig. 4, Supplementary Figs. S1, S2). The BB tutorial describes principles of transcriptome quantification, while the PE tutorial introduces users to the many means of installing required packages. This tutorial will take users from aligned read counts to a single-cell experiment (SCE) object, which may be further analyzed and converted in RStudio (RRID:SCR_000432) or Jupyter Notebook.

Users first generate a transcript-to-gene map using FASTQ files, a GTF file, and a reference FASTA transcriptome. A Salmon index of the transcriptome is created, and a cell-by-gene count matrix is built using Alevin. The BB tutorial combines these two steps using one Galaxy tool and demonstrates basic quality control checks, including a description of the barcode rank plot “knee detection” method.

The PE tutorial identifies empty droplets, adds cell and gene-level metadata, and flags empty droplets based on transcript count. Droplet annotation is corrected for false discovery, and the matrix is filtered before combining the datasets manually. PE users save and export files while converting formats to SCE so they are compatible with downstream analyses.

The BB tutorial incorporates metadata straight from a GTF file using a tool to extract gene names and IDs and to label mitochondrial transcripts. The generated gene information is assigned to the matrix, which is subsequently transposed for compatibility with tools meant for 10x Genomics software. EmptyDrops is then used to remove empty droplets.

Half of the remaining suite emphasizes use of AnnData-compatible packages. To prepare users, tutorials conclude with 1 final format conversion from SCE to AnnData with the SCEasy tool. Once each object has been converted, the BB user concatenates them with a Galaxy tool. The BB tutorial sets the user and their objects up for the next tutorial by adding a number of useful metrics to help visualize the data in the coming tutorial(s). Workflows for each tutorial are shown in Fig. 4.

#### Filter, plot, and explore with Scanpy

**Figure 5:**
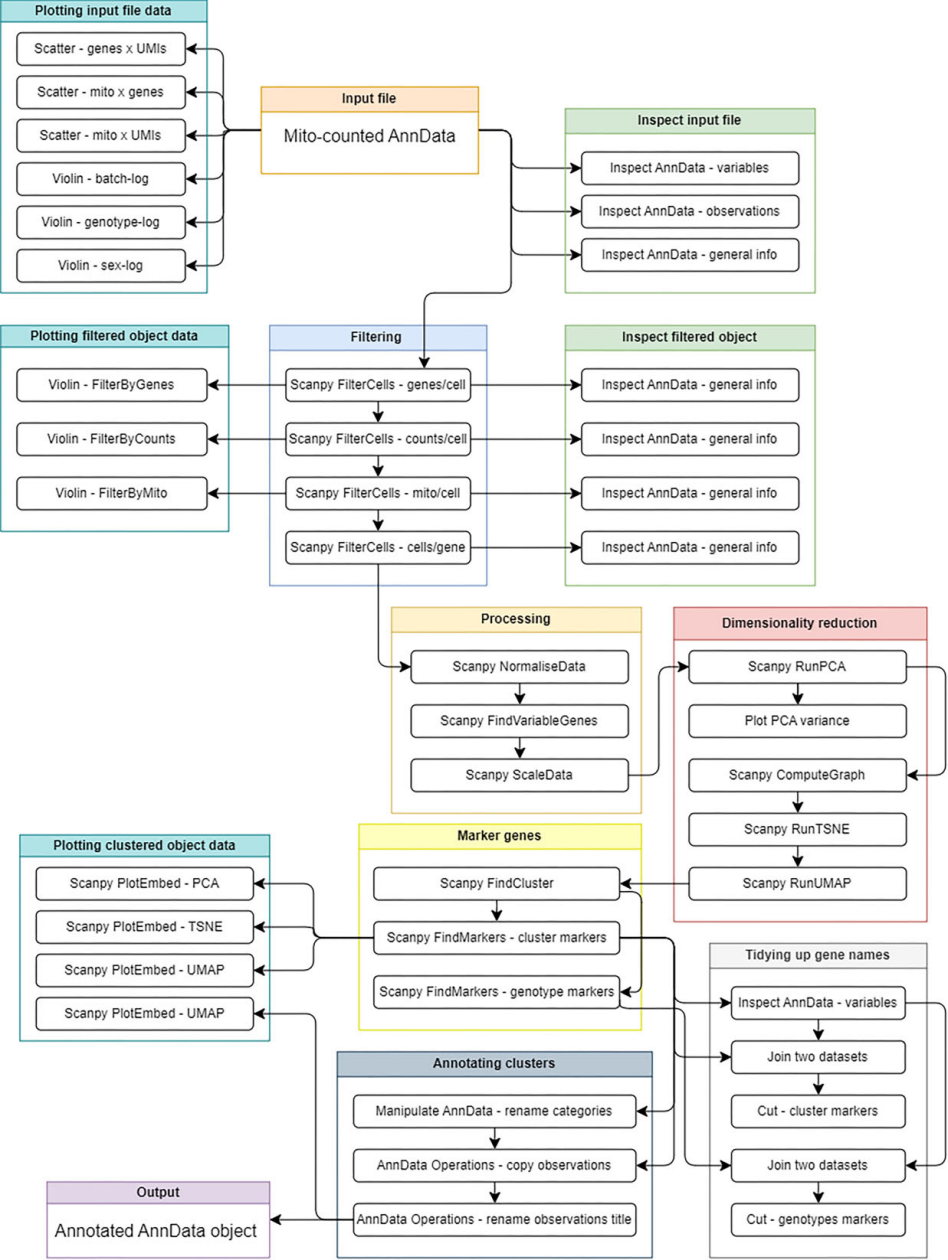
Workflows of plotting and interpretation tutorials: filter, plot, and explore with Scanpy. Features creation of single-cell objects, normalizing data, identifying variable genes, performing dimensionality reduction, identifying clusters, finding marker genes, and interpreting plots. A figure of the extracted Galaxy workflow is available in Supplementary Fig. S3.

These tutorials filter and analyze a preprocessed scRNA-seq matrix using Scanpy (Fig. 5). PE users leverage Python via a Jupyter Notebook to filter the data for noise, as well as accomplish common visualizations and differential expression analysis across clusters for the purpose of cell type labeling.

The PE tutorial imports a raw AnnData file and demonstrates storage as a pandas dataframe, while users iteratively visualize data with violin and scatter plots to determine filtering thresholds. Users filter the data to remove technical artifacts and poor-quality cells. The PE alternatively uses Boolean indexing for this rather than Scanpy’s built-in functions. Users remove transcripts no longer expressed in more than 3 cells and are prompted to compare different thresholds for the filtering of genes.

Log normalization aligns gene expression along a normal distribution. The PE tutorial includes a description of how normalization works and what other methods exist. Variable genes are flagged for use in more computationally demanding steps. Scaling the data ensures all genes have equal variance and a zero mean, creating a matrix that is compatible with downstream analyses.

Users next reduce the dimensionality of the matrix to allow visualization and interpretation. Principal component analysis (PCA) is performed to calculate the most descriptive principal components (PCs). Users plot PCs against the standard variation they describe, visualizing how PCs relate to variance in their data. The PCs are used to compute a k-nearest-neighbors graph, storing a representation of connections between and across cells. Final dimensionality reductions are performed with t-distributed stochastic neighbor embedding (tSNE) [68] and Uniform Manifold Approximation and Projection (UMAP) [69]—both methods reducing the data down to 2 dimensions for visualization.

Scanpy’s clustering function(s) assign each cell to a cluster based on transcriptomic similarity. The tutorials describe clustering algorithms and prompt users to experiment with multiple clustering resolutions, adjusting such that the assigned clusters visually represent what is understood to be biologically accurate. Scanpy’s rank_genes_groups identifies the most representative transcripts for each cluster and genotype, and PE users transform the output into a data frame.

Users visualize all three dimensionality reductions, different clustering resolutions, and the expression of marker genes. A table of marker genes per cell type from the literature is provided so that the user may inspect their expression patterns and map them to the correct cluster(s). Users label each cluster with a cell type, and plots are saved to the Galaxy history or notebook to be exported. BB users are additionally introduced to the CELLxGENE (RRID:SCR_021059) [70] tool: an interactive environment for visualizing and exploring scRNA-seq data. Tutorial workflows are shown in Fig. 5 and Supplementary Fig. S3.

#### Filter, plot, and explore with Seurat

**Figure 6:**
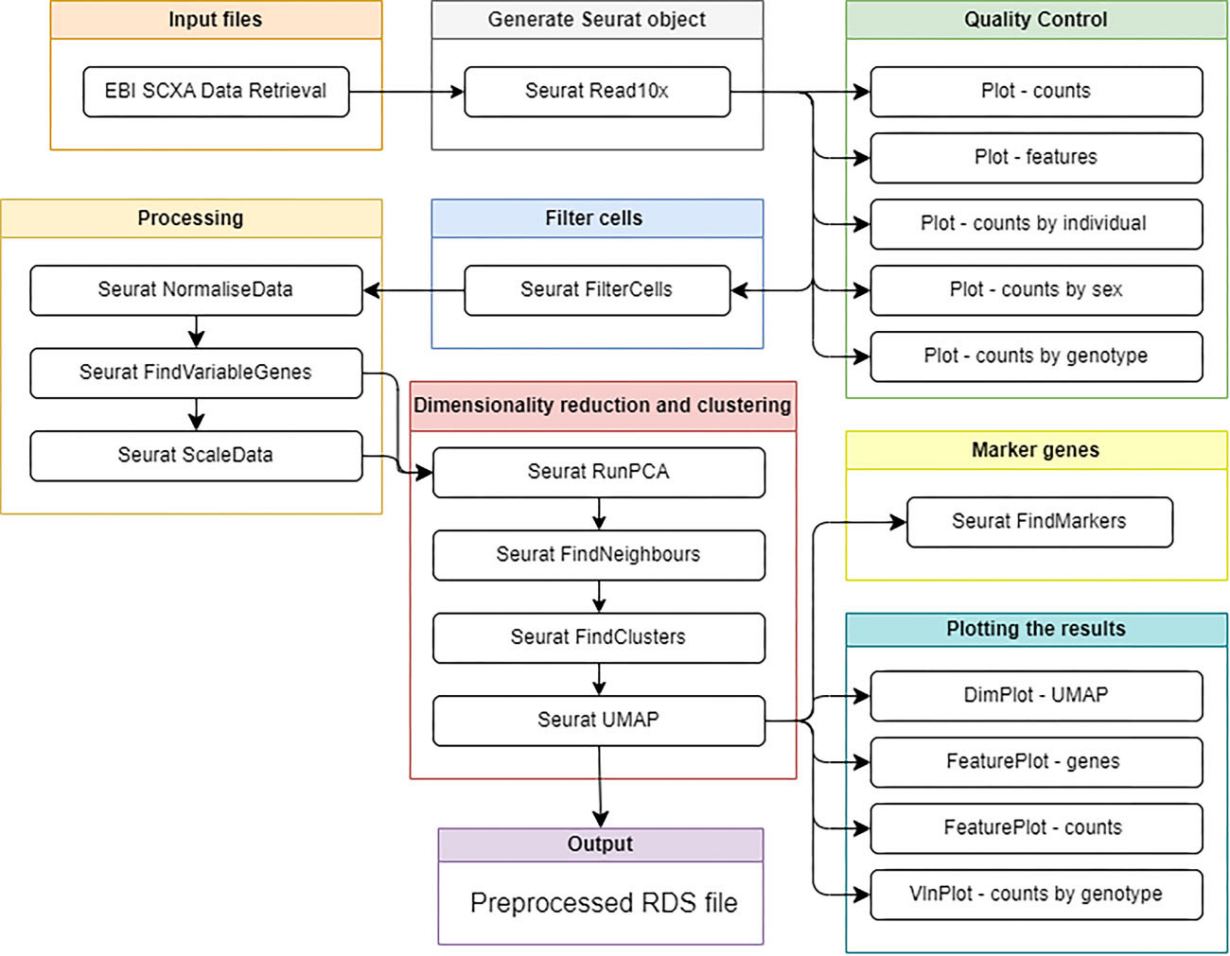
Workflow of the Filter, Plot, and Explore tutorial with Seurat. Features generation of a Seurat object, quality control plots, filtering cells, processing, dimensionality reduction, clustering, finding marker genes, and creating many plots to analyze the results. A figure of the extracted Galaxy workflow is available in Supplementary Fig. S4.

These tutorials closely resemble the workflows of the preceding Scanpy ones, this time making use of the R package Seurat. The workflows teach users the basics of scRNA-seq data analysis, including typical preprocessing; basic visualization with FeaturePlots, DimPlots, and UMAPs; and exploration of differentially expressed genes across clusters and experimental variables (Fig. 6 and Supplementary Fig. S4).

Users import raw counts in both the PE and BB pathways. PE users transition to Galaxy’s Interactive RStudio environment, where they are shown how to set up an environment and given an explanation of how and why packages must be loaded prior to use, as well as how to use Galaxy’s gx_get() function for data import. Users manually change the column names of the experimental design data for compatibility with Seurat.

Users next generate a Seurat object: BB users with Seurat’s Read10X function and PE users by manually applying barcode and feature labels to the matrix for input to Seurat’s CreateSeuratObject function. Each method is accompanied by descriptions of the alternatives for creating the same Seurat object.

Users apply cell-level metadata to their objects. PE users incorporate percent of gene expression (per cell) mapping to the mitochondrial genome—a commonly used parameter for quality control and filtering. Tools are currently being updated to enable BB users to do the same.

Users produce and interpret quality control plots to identify filtering thresholds: assessing potential confounders in the data and developing an understanding of how different variables drive this process. The purpose and theory behind commonly used filtering parameters are described so that users may bring the same (or different) strategies to their own analyses. PE users are additionally shown how to preview the number of cells that would be included based on their choice of filtering parameters.

Both users subset their Seurat object–removing cells outside the chosen threshold(s). PE users additionally remove genes that are now expressed at such low frequencies that they will not contribute biological insight.

Next, users process their filtered object. In the BB, processing of the data includes sequentially normalizing the data, identifying variable features, and scaling. In a more recent update to Seurat’s workflow, the SCTransform function [71, 72] was introduced, which combinatorially conducts the three steps in a manner optimized for downstream analyses. SCTransform is used in the PE tutorial while the BB tutorial follows a similar workflow to the one originally published by Seurat. Both users subsequently cover dimensionality reduction via PCA, deciding on the number of PCs to use, finding neighbors, identifying clusters, and using UMAP before guided visualization and exploration of the data.

#### Inferring single-cell trajectories with Scanpy

Trajectory inferences (TIs), or pseudotime analysis, provide an alternative means of grouping cells based on gradients of expression. It is worth noting that not all TI algorithms are fit for all datasets—these tutorials begin to explore the reasons why and guide users through the decision-making process. These parallel tutorials conduct the typical TI pipeline using Galaxy buttons or in a Python coded environment to characterize transitions between cell states using Scanpy.

Tutorials are significantly based on Scanpy documentation, beginning with import of an annotated AnnData object into Galaxy. Users filter the data to retain a single cell type. The PE tutorial additionally demonstrates installation of modules before transferring their h5ad data to their Jupyter Notebook with the Galaxy–Jupyter cross-talk feature.

Users calculate force directed graphs (FDGs), which represent the data more appropriately for TI than the previously generated tSNE or UMAP visualizations [73]. Optionally, they may create diffusion maps, which can be used in place of PCs to recompute the nearest neighbors visualized in the FDGs.

**Figure 7:**
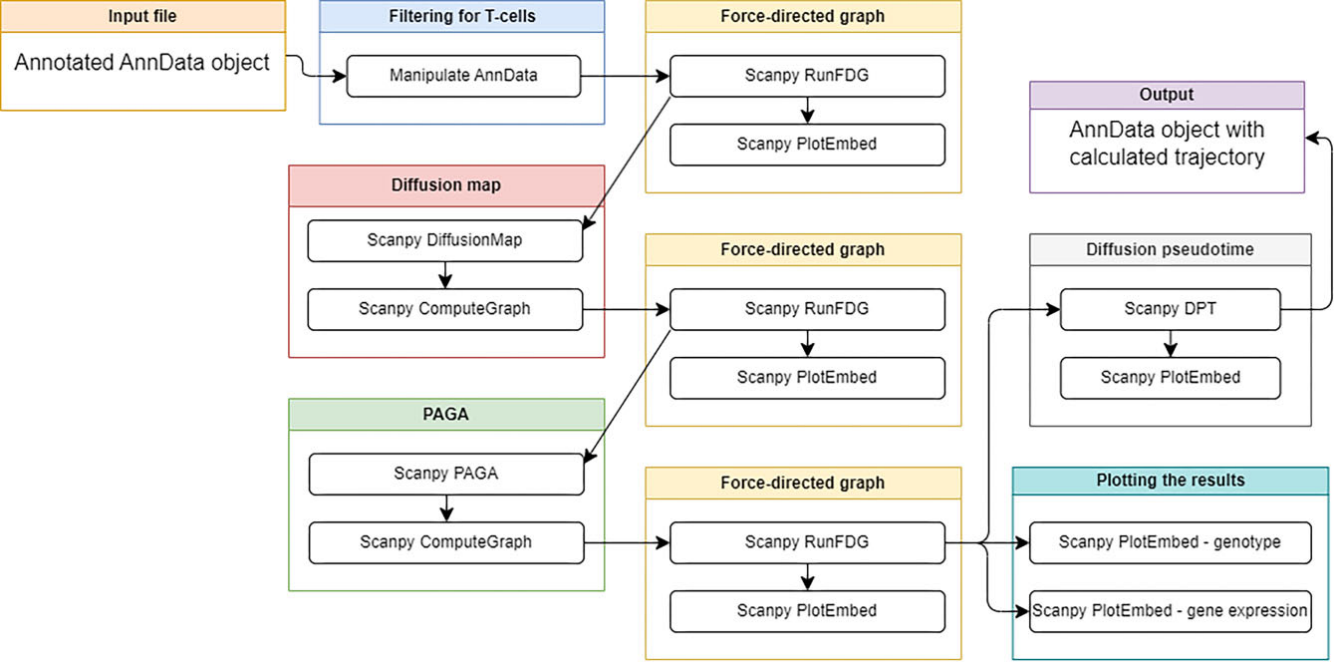
Workflow of inferring trajectories with Scanpy tutorial. Features methods such as force-directed graphs, diffusion maps, and PAGA used to infer the cells’ trajectory in pseudotime. A figure of the extracted Galaxy workflow is available in Supplementary Fig. S5.

Both BB and PE users order cells in pseudotime using Scanpy’s diffusion approach, which accepts root cluster assignment indicating to the algorithm which population of cells the trajectory begins with. Users visualize inferred trajectories colored by pseudotime, as well as save and export their data, plots, and notebook. Users are encouraged to consider other changes across the identified trajectories beyond the scope of the tutorial. Tutorial workflows are shown in Fig. 7 and Supplementary Fig. S5.

#### Inferring single-cell trajectories with Monocle3

**Figure 8:**
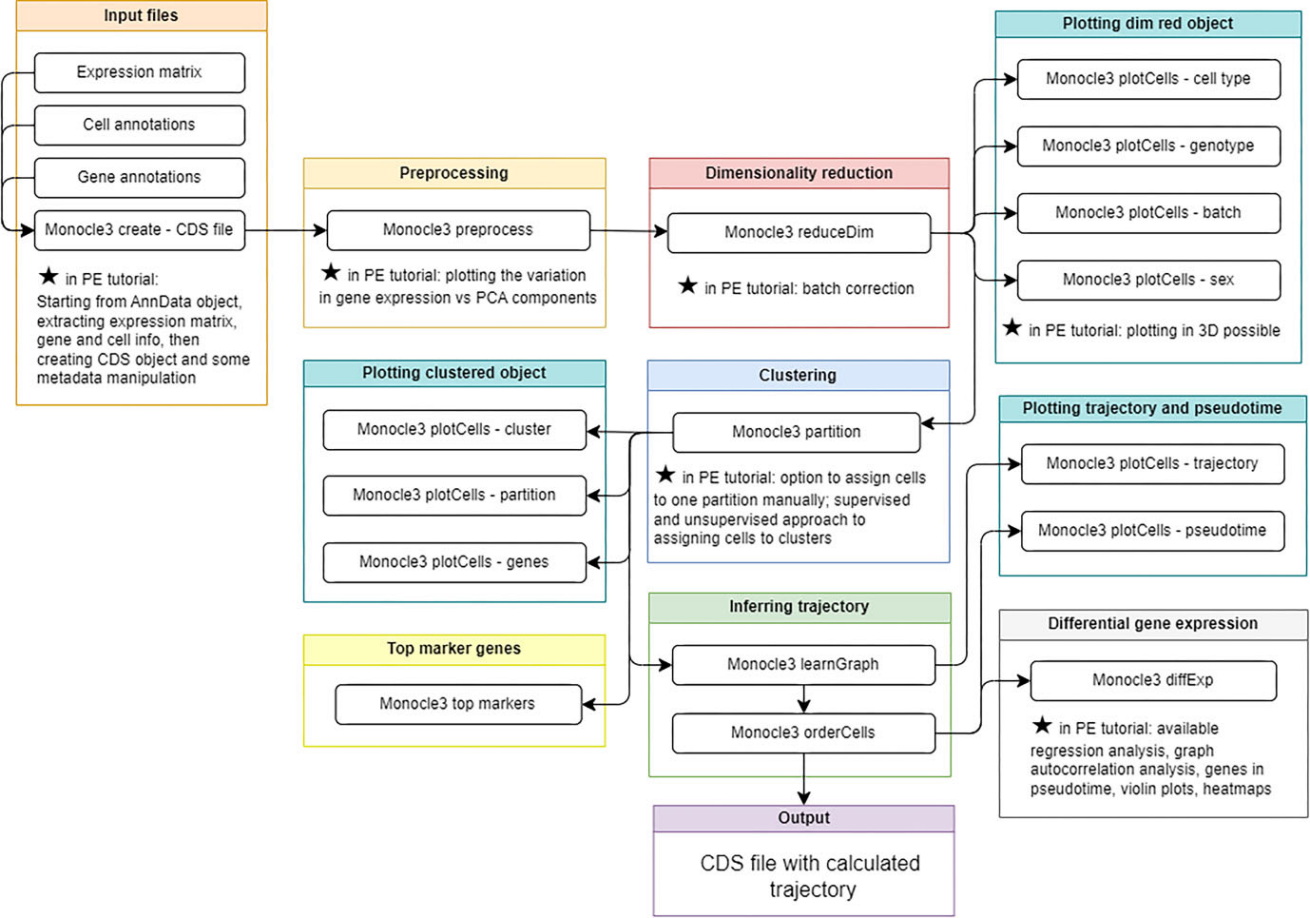
Workflow of Monocle3 inferring trajectories tutorial. Features data type changes for package compatibility, Monocle-specific preprocessing, and trajectory inference on a CDS object, followed by differential gene expression. A figure of an extracted Galaxy workflow is available in Supplementary Fig. S6.

Similarly to the aforementioned, the Monocle3 tutorials teach users to conduct TI (Fig. 8 and Supplementary Fig. S6). These tutorials demonstrate the variability that may arise when trajectories are inferred by different algorithms—this time using the algorithms employed by Monocle3. PE users may implement RStudio or Jupyter Notebook through Galaxy’s Interactive Environments. In collaboration with the Scanpy TI tutorial, users accomplish another TI method to additionally validate their results.

PE users are shown the installation of necessary libraries and modules, and they import a filtered AnnData object and familiarize themselves with the data’s structure. They extract the expression matrix, cell, and gene metadata and prepare them for generation of a Cell Data Set (CDS) object—Monocle’s preferred data type—with format and column name changes, as well as transposition. BB users may import a CDS file ready for downstream analysis in Monocle or the precursor files to create a CDS manually.

PE users utilize the BioMart database to retrieve gene symbols and associated gene IDs. Although not necessary to complete the tutorial’s workflow, this ability is of use to users analyzing their own data.

Users preprocess with Monocle3, beginning with dimensionality reduction. PCA is the method used in these tutorials, although latent semantic indexing (LSI), UMAP, and tSNE options are also available. PE users visualize each PC in relation to gene variance: to identify how many PCs are needed to capture appropriate variability. Users are provided with visualizations of the output data given different choices in PC.

BB users plot the data in a PCA space, visualizing the effects of various experimental design variables. PE users may optionally correct for batch effects and enjoy customizable plots for a more tailored analysis prior to final dimensionality reduction.

Users cluster the data using Monocle3 as the tutorial describes the differences between clusters and partitions. The PE tutorial additionally demonstrates manual partitioning of cells: an important step for reliable trajectory inference.

The PE tutorial demonstrates three combined means of assigning cell types to the clusters—a supervised, an unsupervised, and an automated method. Users next infer trajectories relying on Monocle’s trajectory graph. Once cells have been ordered in pseudotime, starting from the user-directed root cell, cells are visualized colored by pseudotime. BB users end here, comparing the results of the Monocle3-derived trajectory with the Scanpy algorithms.

PE users are presented with more options for differential expression analysis, visualizing results, identifying the visualization method best suited for them, and exporting plots, data, and their Python or RStudio notebook.

## Discussion

We present MIGHTS, a Multi-Interface Galaxy Hands-on Training Suite, where users may embark on three possible learning trajectories: (i) first learning to analyze scRNA-seq data with buttons in a GUI and subsequently performing the same, more flexible analysis in a programming environment; (ii) learning to run the code behind commonly published scRNA-seq analyses; or (iii) supplementing their preexisting analyses and skills with Galaxy tools.

MIGHTS performs analysis from raw reads, guiding users through filtering, normalization, dimensionality reduction, data quality assessment, and biological interpretations. The suite demonstrates filtering, clustering, annotation, and trajectory inference for a well-rounded scRNA-seq skill set. Each analysis is demonstrated using methods based on different packages, libraries, and programming languages with the hope that MIGHTS will prepare users to conduct their own, more complex, analyses.

### Training features

**Figure 9:**
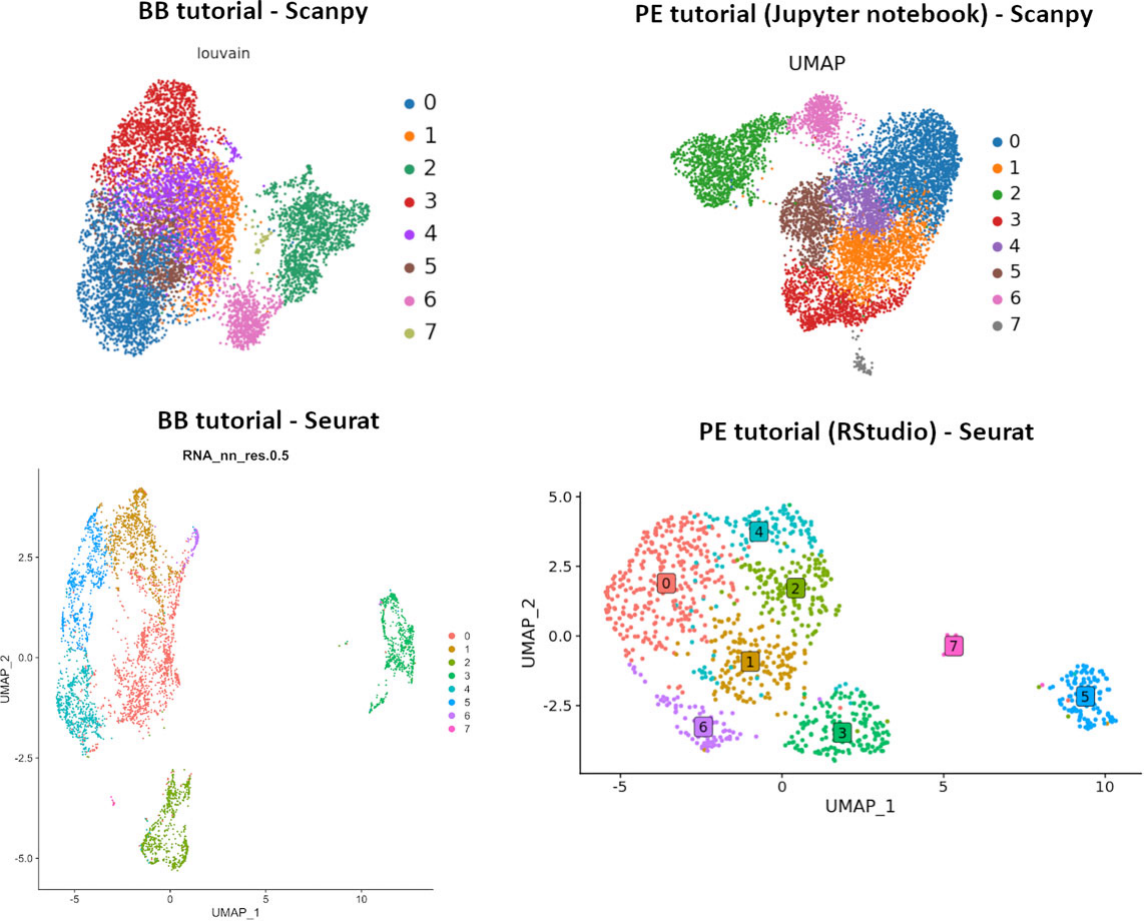
The final “cluster plot” as an output of plotting and interpretation tutorials across 4 paths: BB tutorial with Scanpy, PE (Jupyter notebook) with Scanpy, BB tutorial with Seurat, and PE (RStudio) tutorial with Seurat. The numbers correspond to the identified clusters in the dataset. No matter which method (BB vs. PE) or language (Scanpy vs. Seurat) is used, the biological interpretation is consistent in identifying 7 clusters.

Users of MIGHTS may start at any step by importing preprocessed input files, using output files from the preceding tutorial or their own data. Regardless, the analyses will be replicated across languages, methods, and starting points (Fig. 9), allowing users to follow the trajectory best suited for their skill level and analysis goals. To facilitate choosing the correct starting point depending on experience and goals, single-cell-oriented Learning Pathways were introduced. “Applying single-cell RNA-seq analysis” [74] and “Applying single-cell RNA-seq analysis in Coding Environments” [75] pathways are based on BB and PE tutorials, respectively, and may be used to facilitate a smooth transition between button-based tutorials and a programming environment (Fig. 2A) or a direct start in the programming environment (Fig. 2B). Additionally, to allow for easy identification of the tutorials described here, each tutorial has been tagged and can be found by entering “MIGHTS” in the GTN search box to get access to the relevant materials.

Each tutorial builds on the preceding, with no behind-the-scenes data formatting or annotation required between tutorials. With visual examples and analysis of various data types, live training courses found that trainees who performed tutorials during the day could successfully apply the analyses to their own data in the evenings [27, 28].

**Figure 10:**
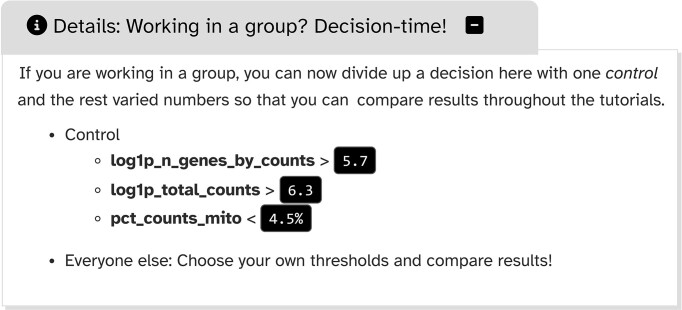
Exemplary “Decision-Time” feature box in tutorial “Filter, plot and explore single-cell RNA-seq data (Scanpy).”

Learning how to set parameters has long been a difficulty in bioinformatics training [76]. By highlighting parameters that are adjusted often, users learn to prioritize what would otherwise be a never-ending list of decision-making. These “Decision-Time” features enable training for individuals and groups, with the option to vary parameter values and compare results (Fig. 10).

Testing of this feature has shown that, broadly, results remain the same regardless of parameter choice, demonstrating the relevance of robust, iterative analyses and data validation [27, 28].

To facilitate effective comprehension and a self-led learning environment, tutorials are interspersed with question boxes and collapsible solutions, allowing users to solidify their understanding of the material while they learn.

**Figure 11:**
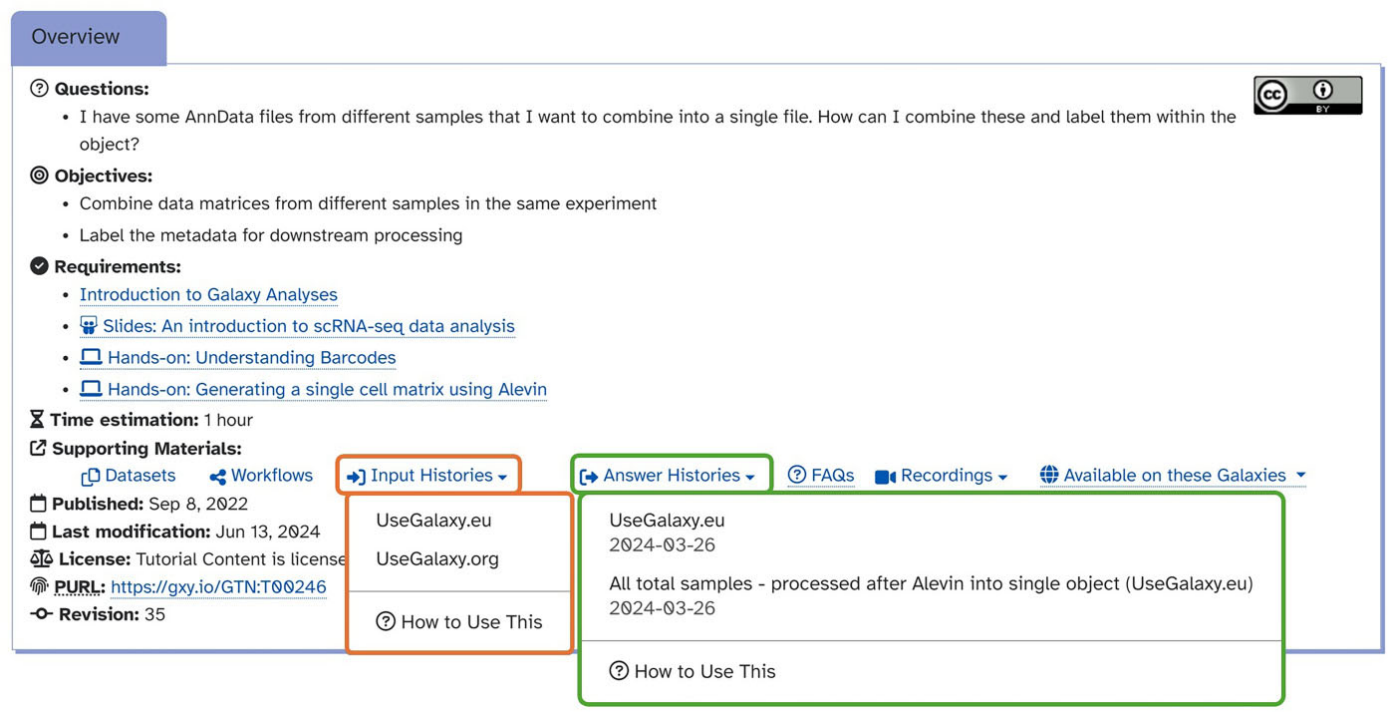
An overview box found at the beginning of the BB tutorial “Combining single cell datasets after pre-processing.” It showcases a header feature, which allows for a quick access to the input histories (orange frame) and answer histories (green frame).

MIGHTS additionally pilots multiple import strategies, ensuring reliability for live training events. This includes direct import from Zenodo [77], import tools linked to data atlases [66], and import from “input” and “answer-key” Galaxy histories—which led to the development of a new feature within the GTN to signpost the option as supporting material (Fig. 11).

“Answer-key” histories follow datasets along every step of analyses, providing a final contingency for delivering live training and protecting users from frustration. Tutorials are additionally accompanied by slide decks (which can act as a general introduction to the topic), as well as recordings of the step-by-step analysis performed by an instructor.

### Learn to code in a beginner-friendly way

As sequencing strategies and tools continue to advance, it is important that the field of bioinformatics “trains the trainer” in response to continued growth. To support comprehension, each tutorial provides detailed explanations of biological and computational concepts, including simplified troubleshooting and multiple interactive elements. By showing alternative methods to perform a single analysis, users become familiar with the most common programming languages used in the life sciences: Python and R, as well as command language Bash. This provides users with well-rounded examples of how to analyze scRNA-seq data and how they may begin to leverage analyses (and Galaxy) as a means to learn new programming skills [78]. These PE tutorials introduce users to relevant packages, functions, and data types used in today’s published bioinformatic analyses (Table 1).

The transition from Galaxy-button tutorials into the coded environment is facilitated by interactive tools such as RStudio or Jupyter Notebook, such that all the analysis may be completed within Galaxy as opposed to on local computers. Importantly, there is no need for any software installation—all tutorials provide necessary tools to complete them, including example datasets, slides, videos, workflows, and public Galaxy servers where the analysis may be performed. Internet access is the only additional necessary resource [79]. This approach specifically facilitates accessible bioinformatics analyses by eliminating installation hang-ups, minimizing the time spent setting one’s environment, and increasing computing capacity for users.

Additionally, if users embrace programming such that they are looking to program their own button-based tools or create new training material, opportunities to do so exist on GTN pages dedicated to development in Galaxy [80] and contributing to the Galaxy Training Material [81].

### FAIR data

MIGHTS tutorials were created on an interface with embedded findable, accessible, interoperable, and reusable (FAIR) data usage [80]. The FAIR principles can and should be applied in all life science domains where large amounts of data are produced. FAIR data management is particularly important in scRNA-seq analysis, which looks at large expression matrices. Unfortunately, it is often the case that published datasets come with missing, or incomplete, metadata—rendering the dataset less useful than it would be with complete annotation(s). By completing MIGHTS tutorials, users become equipped with the skills helpful in formatting such demanding datasets.

### Sustainable

**Figure 12:**
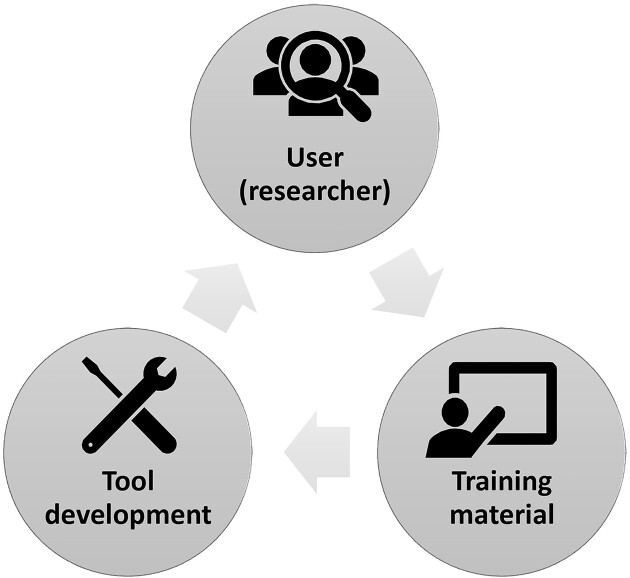
Galaxy Circle of Life demonstrating the interdisciplinary, multilevel sustainability practiced by the GTN. Users report changes they wish to see made in the training material, prompting new tool development and updates that can be sustainably utilized and tested by researchers.

An important feature characterizing MIGHTS is its sustainability. As previously reported, the evolving nature of bioinformatics requires a sustainable bridge between the fields of biology and informatics [81]. Therefore, collaboration between developers and domain experts is critical. The GTN emphasizes that users be included in this collaboration, whereby users have the opportunity to report issues and request additional resources. This facilitates involvement of developers who are aware of user needs and users who are actively contributing to the improvement of materials. Any issues may be reported back to tutorial developers themselves, demonstrating the sustainability of Galaxy’s Circle of Life (Fig. 12).

Tutorials on the GTN are, at minimum, updated annually in preparation for the Galaxy Community Conference (GCC). The Galaxy Circle of Life functions such that tutorials will continue to meet evolving user needs—largely thanks to the commitment of the growing Galaxy Single-Cell & Spatial Omics Community. Notably, MIGHTS tutorials have been updated, on average, 7 times a year since their respective publication (Table 2).

**Table 2:** Number of revisions made to each tutorial featured in MIGHTS as of August 2024

Tutorial topic	BB tutorials		PE tutorials	
	Months since tutorial publication	Number of revisions	Months since tutorial publication	Number of revisions
*Generating a single-cell matrix using Alevin*	41	16	8	2
*Combining single-cell datasets after preprocessing*	23	16		
*Filter plot explore scRNA-seq data with Scanpy*	40	18	11	9
*Filter plot and explore scRNA-seq data with Seurat*	4	3	10	8
*Inferring single-cell trajectories with Scanpy*	8	5	40	15
*Inferring single-cell trajectories with Monocle3*	22	19	15	10

The number of revisions demonstrates continued sustainability of tutorials featured in the suite.

Notably, the GTN offers tutorials on additional bioinformatic analyses in a variety of fields. These tutorials are similarly monitored and revised, although the rate of growth specifically for single-cell tutorials is worth noting.

### Addressing modern challenges in bioinformatics

MIGHTS addresses many broad challenges of bioinformatics training, emphasizing that effective bioinformatics involves understanding key principles and gaining experience [22] with real-world data, problem-solving [24], reproducibility [82], and validation [83].

One challenge in bioinformatics is the application of analyses from training courses to real, messy, lab-generated data. Uniquely, MIGHTS uses raw, unannotated data from a published analysis—Bacon et al. [31]—and guides users through reformatting, annotating, and conducting biological analyses.

Reproducibility and reinstantiation, keystones of quality bioinformatic analyses, are ensured by MIGHTS thanks to published workflows and answer key histories for each dataset (Fig. 11). Workflows are available on the Galaxy servers, providing a stable way to perform a particular analysis in an identical environment. By linking detailed tool versions to the tutorial workflows themselves, it is possible to submit new input files, adjust parameter thresholds, and wait for an output. This is particularly helpful for analyzing multiple samples that require the same pipeline, allowing reproducible results, minimizing time spent rerunning code, and eliminating the need for complex coding skills to develop pipelines.

To address another challenge of the field, MIGHTS emphasizes the importance of validating one’s results: to determine whether results reflect an actual biological process versus artifacts of the pipeline. Using tools based in various programming languages and multiple algorithms allows users to feel confident that their results are uncovering true biological insights no matter the analysis method used (Fig. 9). MIGHTS can act as a guide on how to validate results. The suite may additionally be used directly by novice to intermediate bioinformaticians to check whether their results are consistent via alternative methods without needing to learn another programming language.

Because bioinformatics combines numerous STEM fields, it faces interdisciplinary and intergenerational challenges [84]. Software developers often do not understand the underlying biology their programs analyze, and biologists often do not know how analytical algorithms function [85]. MIGHTS aims to fill this gap by introducing step-by-step analyses, while simultaneously demonstrating the biological interpretation of results and how they were uncovered. Coded tutorials provide additional opportunities to become familiar with the algorithms behind the analyses. The suite can act as a resource to educate and inspire future generations of bioinformaticians: ones who are able to speak across disciplines, effectively identify areas for improvement, and build flexible, long-term solutions. Tutorials are available by link on Galaxy [86–96]

### Limitations and further steps

The main limitation of the Galaxy GUI tutorials is that the analyses are limited to the packages and functions that have been wrapped into tools. As such, some analysis steps might be limited in the BB tutorials. However, users have the opportunity to submit “tool requests”: an ongoing effort to mitigate this limitation.

Additionally, tool versions must be compatible with one another. To mitigate this limitation, tools are regularly tested and updated to allow for analysis using the most recent versions and ensuring outputs are compatible inputs for downstream steps. Issues with tools may be reported on Galaxy forums, where experts and developers respond quickly to issues.

The main limitations of the PE tutorials are limited resources allocated to Interactive Environments and inconsistencies between the notebooks on different public Galaxy servers (.eu vs. .org vs. .au). However, the educational purpose of the coded-tutorials is to familiarize users with coding environments, so downsampled data provide the same benefits and enable most analyses to be done within the resource limit. Even so, should a user need or want more resources allocated, they can request that from the Galaxy admins.

There are ongoing efforts to expand the functionality of MIGHTS to enable more bespoke analyses of datasets, in response to community needs.

## Availability of Source Code and Requirements

- Project name: Multi-Interface Galaxy Hands-on Training Suite for scRNA-seq
- Project homepage: https://github.com/galaxyproject/training-material/tree/main/topics/single-cell/tutorials
- Operating system(s): web-based, platform independent
- Programming languages: R, Python, Bash
- License: MIT

## Supplementary Material

giae107_Supplemental_Files

giae107_GIGA-D-24-00327_Original_Submission

giae107_GIGA-D-24-00327_Revision_1

giae107_Response_to_Reviewer_Comments_Original_Submission

giae107_Reviewer_1_Report_Original_Submission Prashanth Suravajhala -- 9/25/2024 Reviewed

giae107_Reviewer_2_Report_Original_Submission Delphine Lariviere -- 9/29/2024 Reviewed

## Data Availability

All the tutorials are available at the dedicated Single Cell subpage of the Galaxy Training Network (GTN) [97]. The used experimental data come from a published study by Bacon et al. [31], which is publicly available from the EMBL-EBI ArrayExpress under accession number E-MTAB-6945 and can also be browsed from the Single Cell Expression Atlas [66]. The input datasets used in tutorials are stored at Zenodo [98–103], and all generated data files are available in the shared Galaxy histories, included in each tutorial. The tutorials comprise many different tools that can be freely used at the Galaxy public servers, such as Galaxy Europe [104], Galaxy US [105], Galaxy Australia [106], and others. The tool wrappers with detailed information are stored at the Galaxy ToolShed [107].

## Additional Files

**Supplementary Fig. S1**. Galaxy workflow for tutorial “Generating a single-cell matrix using Alevin.” The tools used for the analysis are shown, together with their outputs and connectivities, as well as high-level descriptors of performed steps. Solid stars denote steps specific to the PE tutorial while unfilled stars represent BB-specific ones.

**Supplementary Fig. S2**. Galaxy workflow for tutorial “Combining single-cell datasets after pre-processing.” All tools used for the analysis are shown, as well as their connectivities and high-level descriptors of performed steps. Solid star denotes steps specific to the PE tutorial.

**Supplementary Fig. S3**. Galaxy workflow for tutorial “Filter, plot and explore single-cell RNA-seq data (Scanpy).” All tools used for the analysis are shown, as well as their connectivities and high-level descriptors of performed steps.

**Supplementary Fig. S4**. Galaxy workflow for tutorial “Filter, plot and explore single-cell RNA-seq data (Seurat).” All tools used for the analysis are shown, as well as their connectivities and high-level descriptors of performed steps.

**Supplementary Fig. S5**. Galaxy workflow for tutorial “Inferring single-cell trajectories (Scanpy).” All tools used for the analysis are shown, as well as their connectivities and high-level descriptors of performed steps.

**Supplementary Fig. S6**. Galaxy workflow for tutorial “Inferring single-cell trajectories (Monocle3).” All tools used for the analysis are shown, as well as their connectivities and high-level descriptors of performed steps.

## Abbreviations

BB: button based; CDS: cell data set; DPT: diffusion pseudotime; EMBL-EBI: European Molecular Biology Laboratory–European Bioinformatics Institute; FAIR: findable, accessible, interoperable, and reusable; FDG: force-directed graph; GCC: Galaxy Community Conference; GTF: gene transfer format; GTN: Galaxy Training Network; GUI: graphical user interface; LSI: latent semantic indexing; MIGHTS: Multi-Interface Galaxy Hands-on Training Suite for scRNA-seq; PAGA: partition-based graph abstraction; PC: principal component; PCA: principal component analysis; PE: programming environment; QC: quality control; SCE: single-cell experiment; scRNA-seq: single-cell RNA sequencing; SE: summarized experiment; STEM: Science, Technology, Engineering, and Mathematics; TI: trajectory inference; tSNE: t-distributed stochastic neighbor embedding; UMAP: Uniform Manifold Approximation and Projection.

## Notes

Co-first authors and middle authors have been ordered alphabetically.
