# Supplementary figures and images for: Galaxy as a gateway to bioinformatics: Multi-Interface Galaxy Hands-on Training Suite (MIGHTS) for scRNA-seq
Source: Gigascience. 2025 Jan 8;14:giae107. doi: 10.1093/gigascience/giae107 (PMC11707610; doi:10.1093/gigascience/giae107)

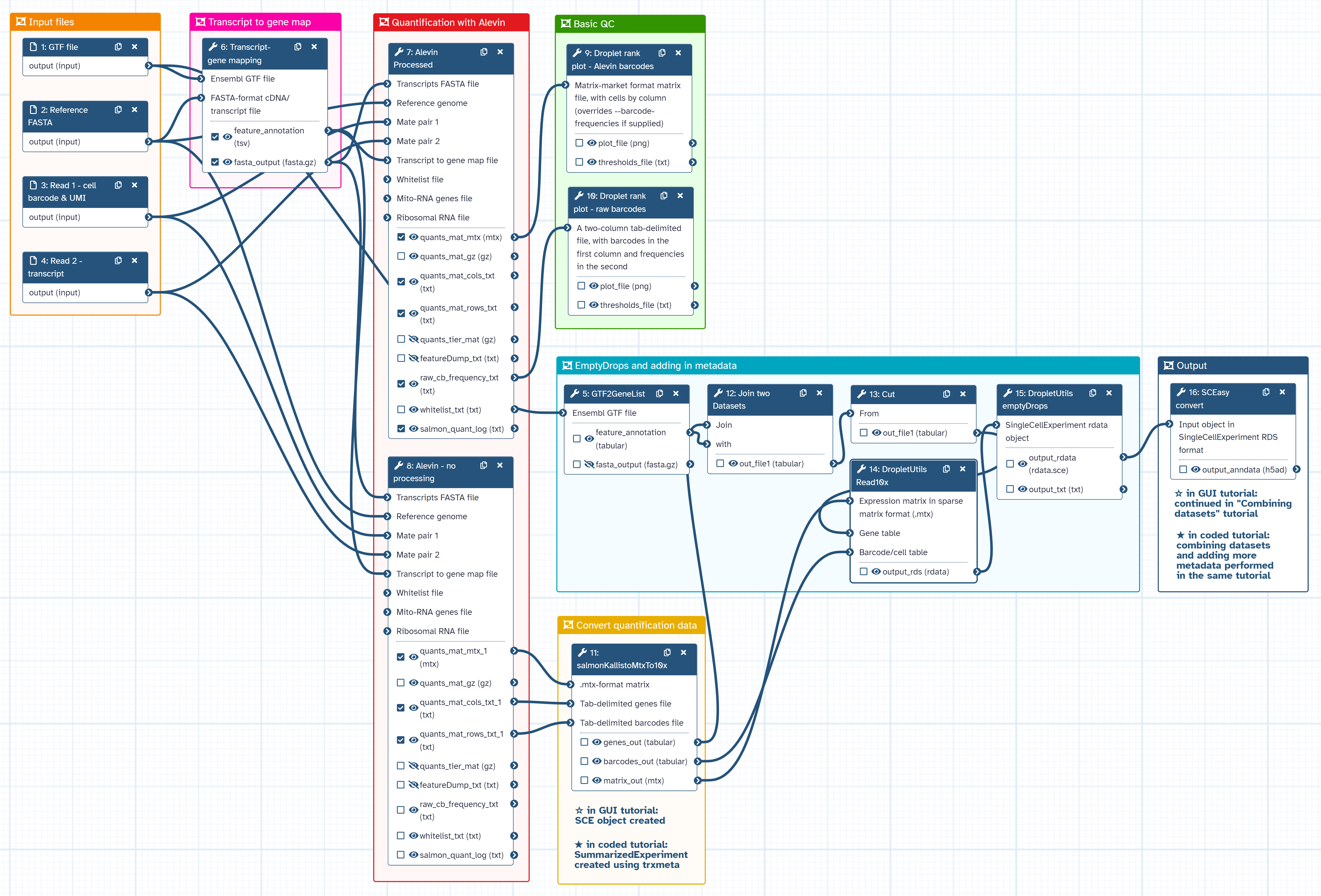

Supplement: giae107_Supplemental_Files [file giae107_supplemental_files.zip › FigS1_Alevin_workflow-min.png]

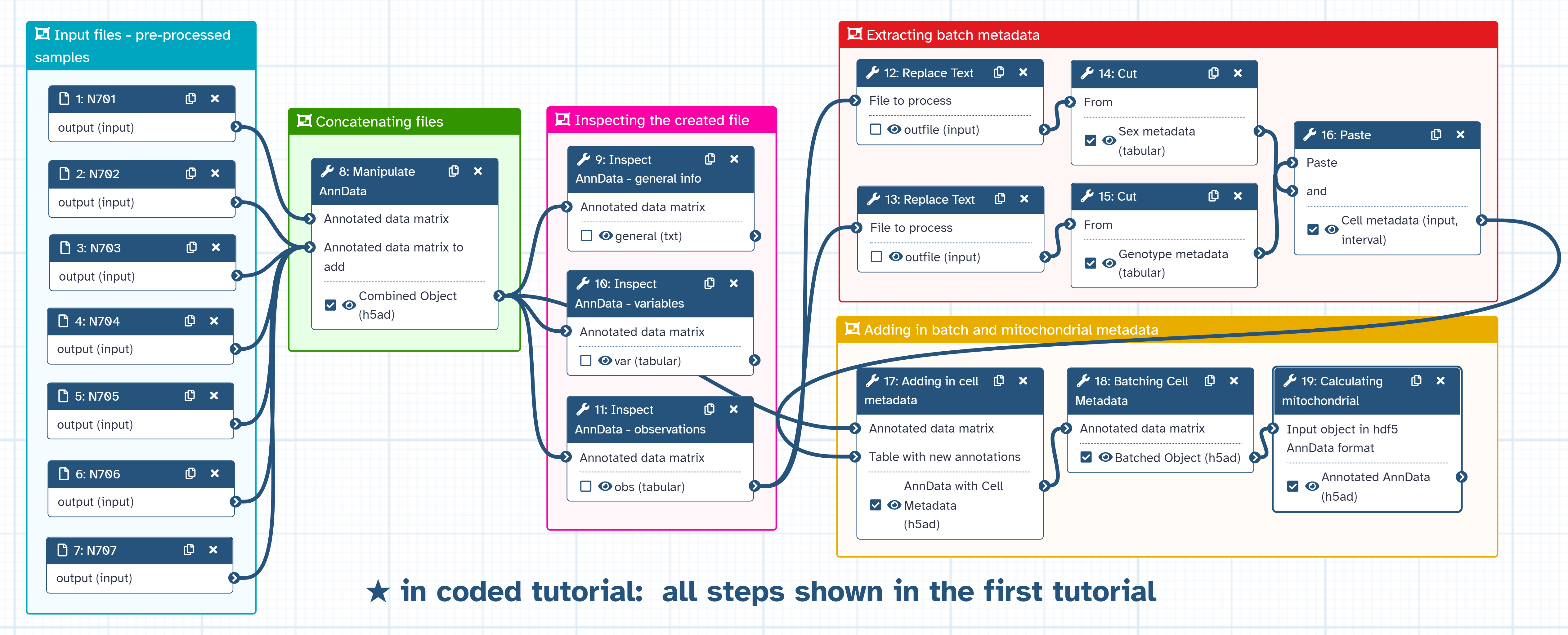

Supplement: giae107_Supplemental_Files [file giae107_supplemental_files.zip › FigS2_combining_datasets-min.png]

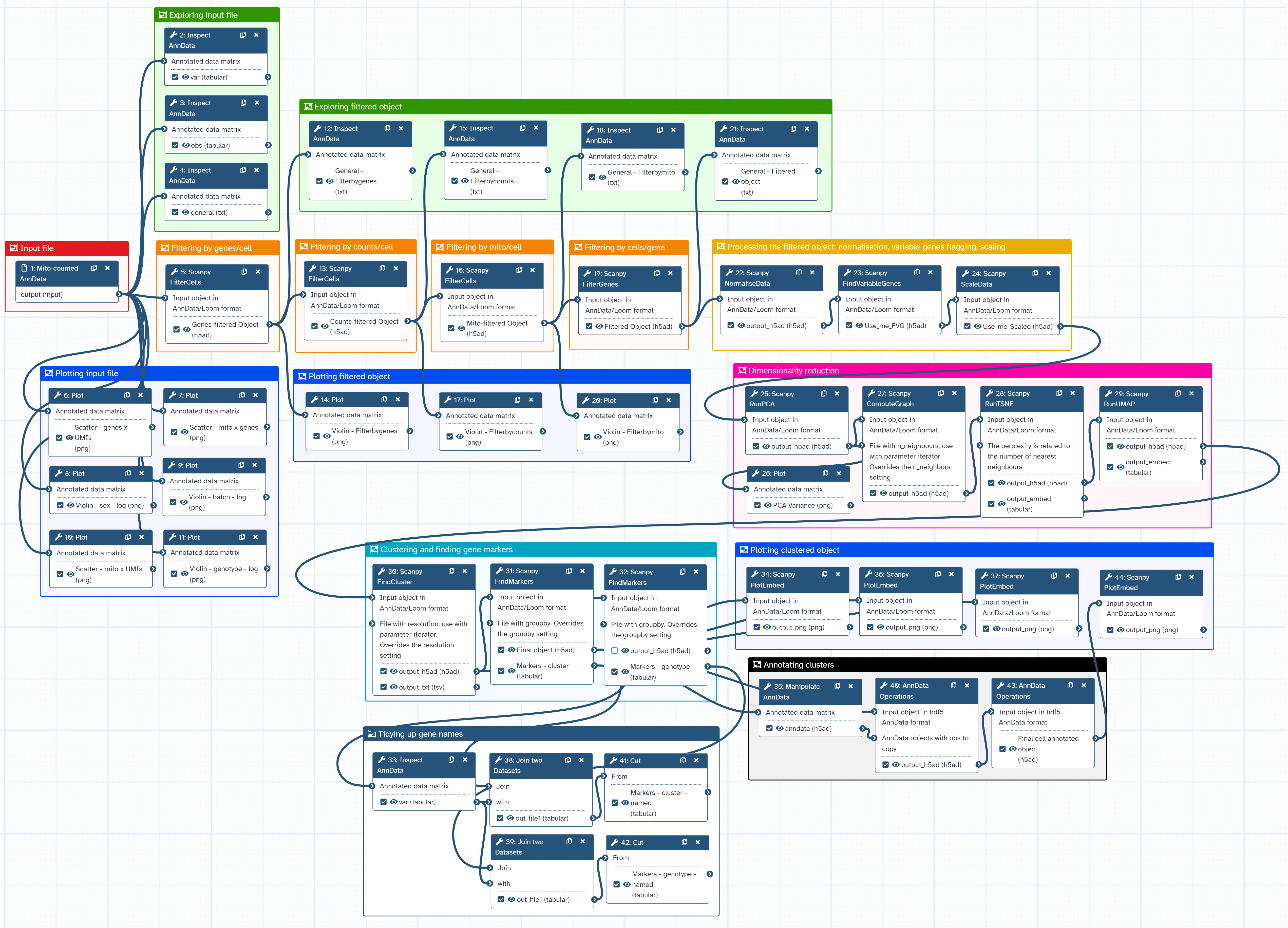

Supplement: giae107_Supplemental_Files [file giae107_supplemental_files.zip › FigS3_FPE-min.png]

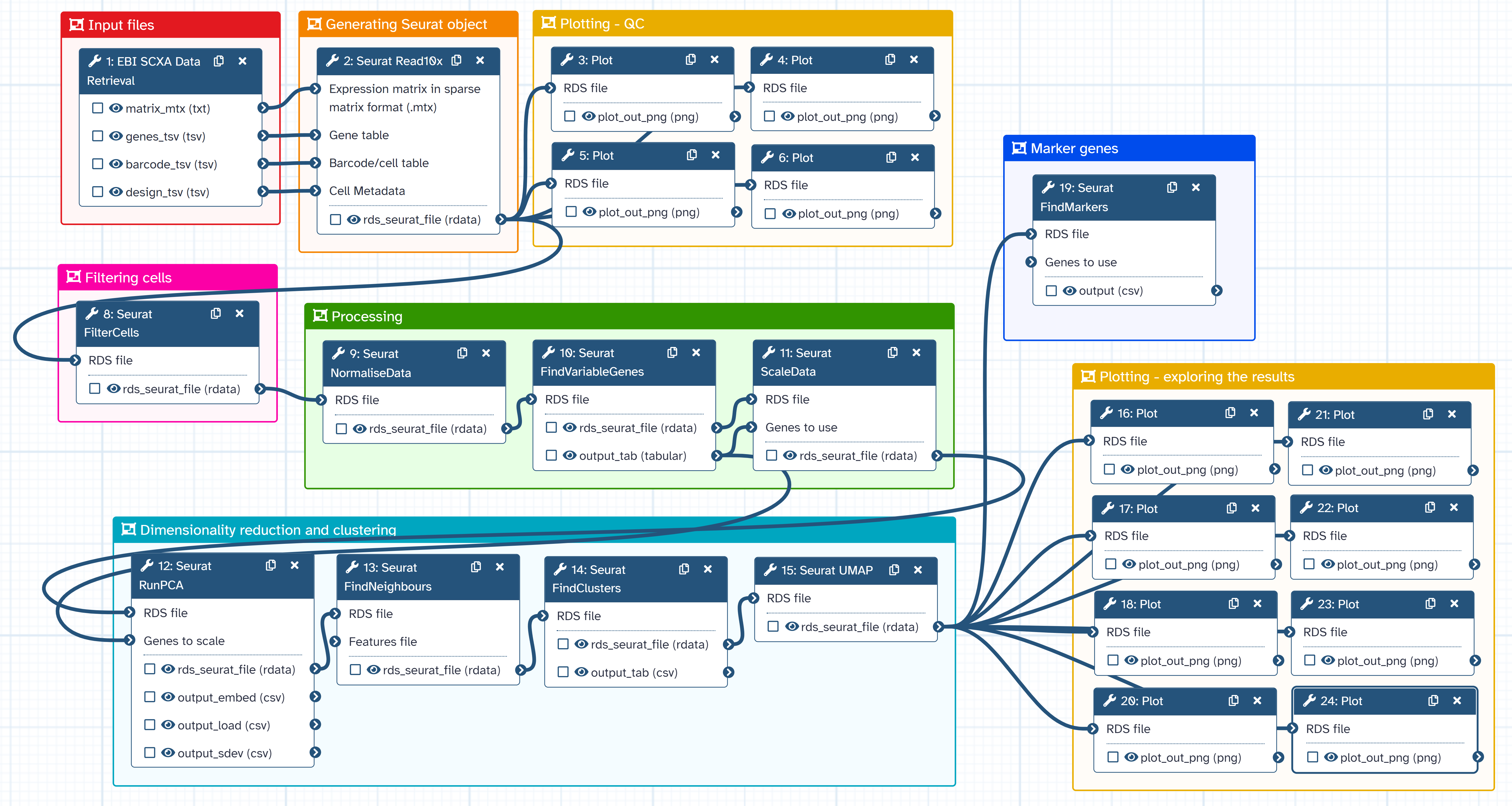

Supplement: giae107_Supplemental_Files [file giae107_supplemental_files.zip › FigS4_seurat_FPE-min.png]

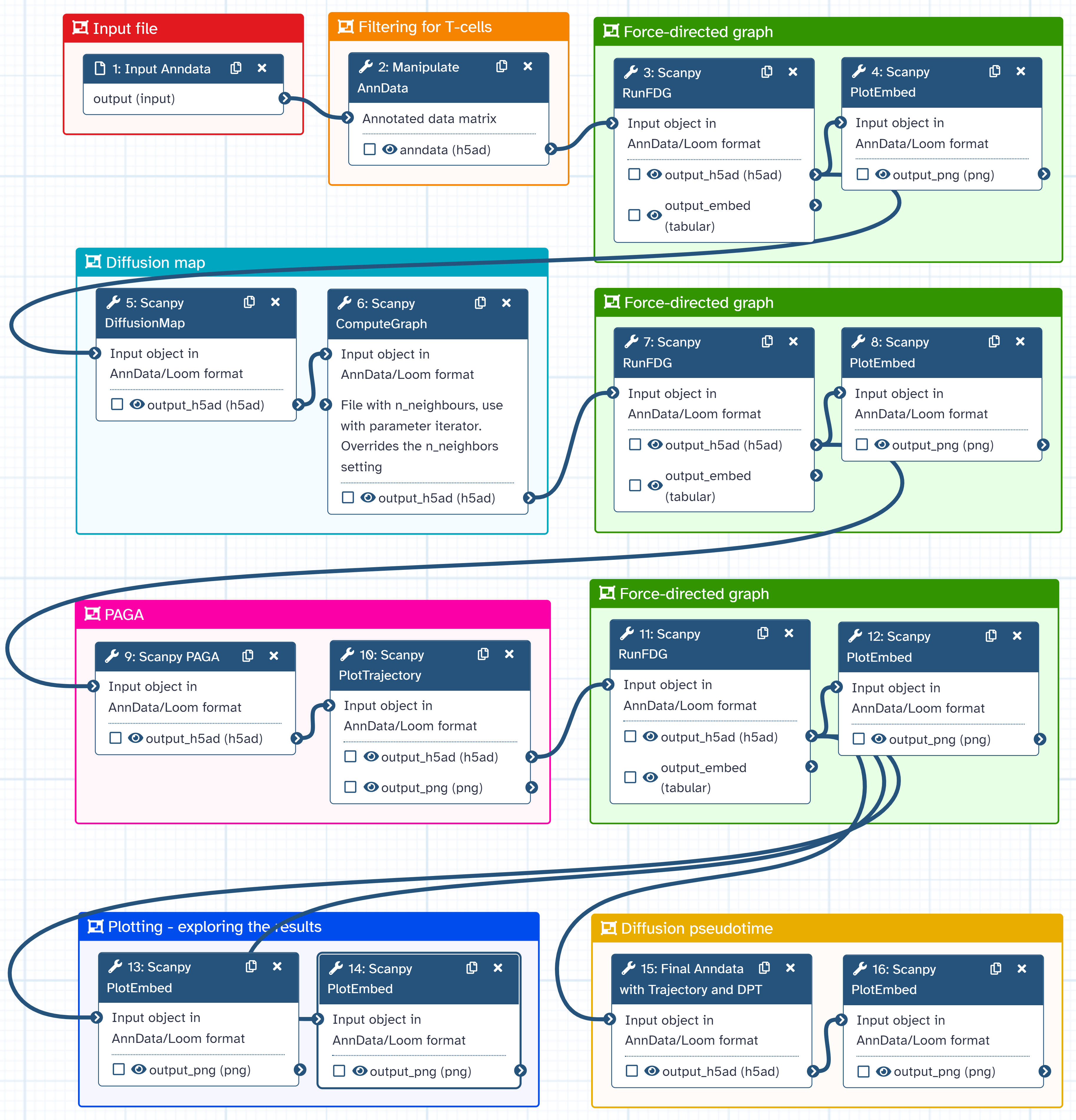

Supplement: giae107_Supplemental_Files [file giae107_supplemental_files.zip › FigS5_trajectories_scanpy-min.png]

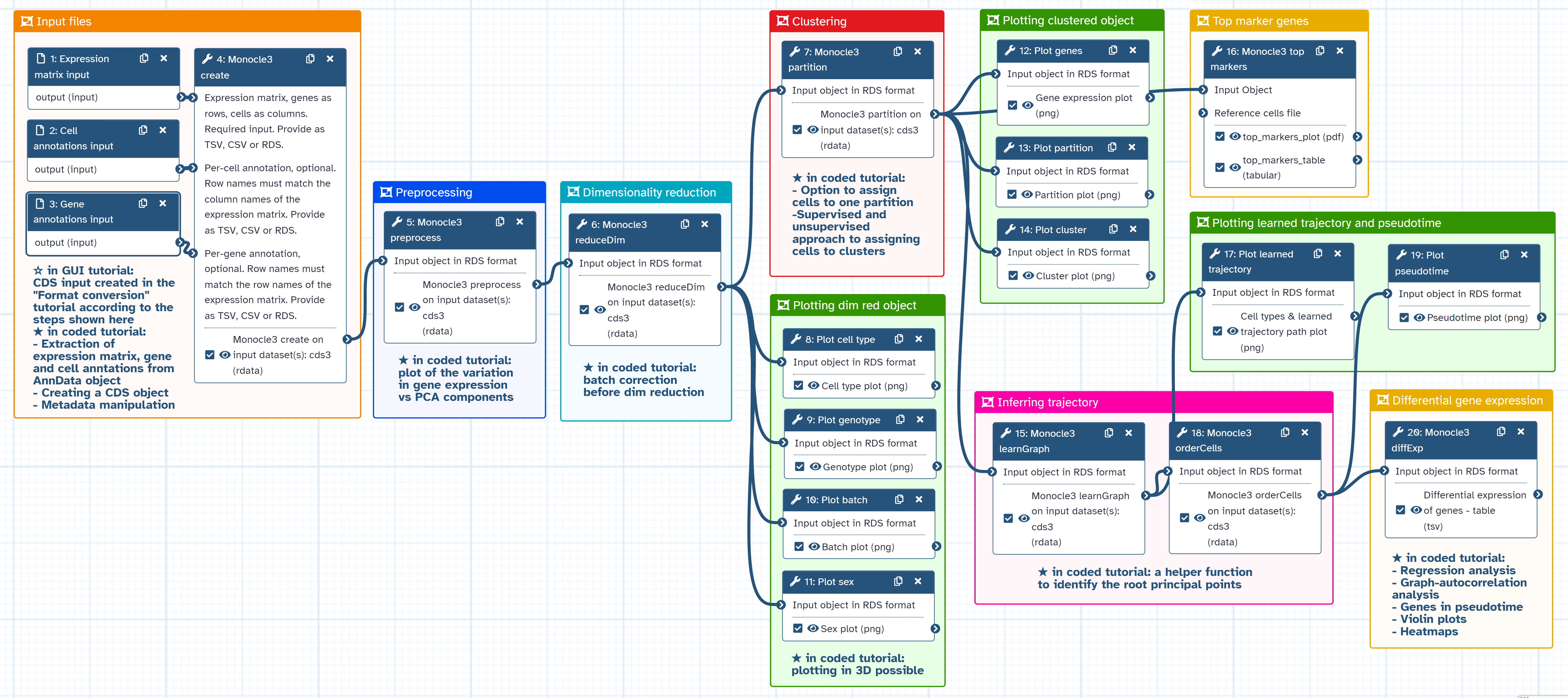

Supplement: giae107_Supplemental_Files [file giae107_supplemental_files.zip › FigS6_monocle-min.png]
