# Supplementary material for: Galaxy as a gateway to bioinformatics: Multi-Interface Galaxy Hands-on Training Suite (MIGHTS) for scRNA-seq
Source: Gigascience. 2025 Jan 8;14:giae107. doi: 10.1093/gigascience/giae107 (PMC11707610; doi:10.1093/gigascience/giae107)
Supplement: giae107_GIGA-D-24-00327_Original_Submission [file giae107_giga-d-24-00327_original_submission.pdf]

GigaScience

Galaxy as a Gateway to Bioinformatics: Multi-Interface Galaxy Hands-on Training Suite (MIGHTS) for scRNA-seq

--Manuscript Draft--

**Manuscript Number:**	GIGA-D-24-00327
**Full Title:**	Galaxy as a Gateway to Bioinformatics: Multi-Interface Galaxy Hands-on Training Suite (MIGHTS) for scRNA-seq
**Article Type:**	Technical Note
**Funding Information:**	Horizon 2020 (824087) — Ms. Julia Jakiela<br>Hobart and William Smith Colleges — Ms. Camila Goclowski<br>Engineering and Physical Sciences Research Council — Mr. Morgan Howells
**Abstract:**	Background. Bioinformatics is fundamental to biomedical sciences, but its mastery presents a steep learning curve for bench biologists and clinicians. Learning to code while analyzing data is difficult. The curve may be flattened by separating the two aspects and providing intermediate steps for budding bioinformaticians. Single-cell analysis is in great demand from biologists and biomedical scientists, as evidenced by the proliferation of training events, materials, and collaborative global efforts like the Human Cell Atlas. However, iterative analyses and un-standardized pipelines have made effective single-cell training a moving target. Findings. To address these challenges, we present a Multi-Interface Galaxy Hands-on Training Suite (MIGHTS) for scRNA-seq analysis, which offers parallel analytical methods using a graphical interface (buttons) or code. With clear, interoperable materials, MIGHTS facilitates smooth transitions between environments. Bridging the biologist-programmer gap, MIGHTS emphasizes interdisciplinary communication for effective learning at all levels. Real-world data analysis in MIGHTS promotes critical thinking and best practices, while FAIR data principles ensure validation of results. MIGHTS is freely available, hosted on the Galaxy Training Network, and leverages Galaxy interfaces for analyses in both settings. Given the ongoing popularity of Python-based (Scanpy) and R-based (Seurat, Monocle) scRNA-seq analyses, MIGHTS enables analyses using both. Conclusions. MIGHTS consists of 11 tutorials including recordings, slide-decks, and interactive visualizations, with a proven track record of sustainability via regular updates and community collaborations. Parallel pathways in MIGHTS enable concurrent training of scientists at any programming level, addressing the heterogeneous needs of novice bioinformaticians.
**Corresponding Author:**	Wendi Bacon, PhD<br>The Open University<br>Milton Keynes, UNITED KINGDOM
**Corresponding Author Secondary Information:**	
**Corresponding Author's Institution:**	The Open University
**Corresponding Author's Secondary Institution:**	
**First Author:**	Camila Goclowski
**First Author Secondary Information:**	
**Order of Authors:**	Camila Goclowski<br>Julia Jakiela<br>Tyler Collins<br>Saskia Hiltemann<br>Morgan Howells

Powered by Editorial Manager® and ProduXion Manager® from Aries Systems Corporation

	Marisa Loach
	Jonathan Manning
	Pablo Moreno
	Alex Ostrovsky
	Helena Rasche
	Mehmet Tekman
	Graeme Tyson
	Pavankumar Videm
	Wendi Bacon
**Order of Authors Secondary Information:**	
**Additional Information:**	
**Question**	**Response**
Are you submitting this manuscript to a special series or article collection?	No
**Experimental design and statistics**<br><br>Full details of the experimental design and statistical methods used should be given in the Methods section, as detailed in our Minimum Standards Reporting Checklist. Information essential to interpreting the data presented should be made available in the figure legends.<br><br>Have you included all the information requested in your manuscript?	Yes
**Resources**<br><br>A description of all resources used, including antibodies, cell lines, animals and software tools, with enough information to allow them to be uniquely identified, should be included in the Methods section. Authors are strongly encouraged to cite Research Resource Identifiers (RRIDs) for antibodies, model organisms and tools, where possible.<br><br>Have you included the information requested as detailed in our Minimum Standards Reporting Checklist?	Yes

**Availability of data and materials**<br><br>All datasets and code on which the conclusions of the paper rely must be either included in your submission or deposited in publicly available repositories (where available and ethically appropriate), referencing such data using a unique identifier in the references and in the "Availability of Data and Materials" section of your manuscript.<br><br>Have you have met the above requirement as detailed in our Minimum Standards Reporting Checklist?	Yes

Powered by Editorial Manager® and ProduXion Manager® from Aries Systems Corporation

Click here to access/download;LaTeX - Main Document (TeX file);main.tex

```
This is pdfTeX, Version 3.141592653-2.6-1.40.25 (TeX Live 2023) (preloaded format=pdflatex 2024.3.8)  13 AUG 2024 16:37
entering extended mode
 restricted \write18 enabled.
 %&-line parsing enabled.
**main.tex
(./main.tex
LaTeX2e <2023-11-01> patch level 1
L3 programming layer <2024-02-20>
(./oup-contemporary.cls
Document Class: oup-contemporary 2024/08/12, v1.2
(c:/texlive/2023/texmf-dist/tex/latex/base/article.cls
Document Class: article 2023/05/17 v1.4n Standard LaTeX document class
(c:/texlive/2023/texmf-dist/tex/latex/base/size10.clo
File: size10.clo 2023/05/17 v1.4n Standard LaTeX file (size option)
)
\c@part=\count188
\c@section=\count189
\c@subsection=\count190
\c@subsubsection=\count191
\c@paragraph=\count192
\c@subparagraph=\count193
\c@figure=\count194
\c@table=\count195
\abovecaptionskip=\skip48
\belowcaptionskip=\skip49
\bibindent=\dimen140
) (c:/texlive/2023/texmf-dist/tex/latex/base/inputenc.sty
Package: inputenc 2021/02/14 v1.3d Input encoding file
\inpenc@prehook=\toks17
\inpenc@posthook=\toks18
) (c:/texlive/2023/texmf-dist/tex/latex/base/fontenc.sty
Package: fontenc 2021/04/29 v2.0v Standard LaTeX package
) (c:/texlive/2023/texmf-dist/tex/generic/iftex/ifpdf.sty
Package: ifpdf 2019/10/25 v3.4 ifpdf legacy package. Use iftex instead.
(c:/texlive/2023/texmf-dist/tex/generic/iftex/iftex.sty
Package: iftex 2022/02/03 v1.0f TeX engine tests
)) (c:/texlive/2023/texmf-dist/tex/latex/microtype/microtype.sty
Package: microtype 2023/03/13 v3.1a Micro-typographical refinements (RS)
(c:/texlive/2023/texmf-dist/tex/latex/graphics/keyval.sty
Package: keyval 2022/05/29 v1.15 key=value parser (DPC)
\KV@toks@=\toks19
) (c:/texlive/2023/texmf-dist/tex/latex/etoolbox/etoolbox.sty
Package: etoolbox 2020/10/05 v2.5k e-TeX tools for LaTeX (JAW)
\etb@tempcnta=\count196
)
\MT@toks=\toks20
\MT@tempbox=\box51
\MT@count=\count197
LaTeX Info: Redefining \noprotrusionifhmode on input line 1059.
LaTeX Info: Redefining \leftprotrusion on input line 1060.
\MT@prot@toks=\toks21
LaTeX Info: Redefining \rightprotrusion on input line 1078.
LaTeX Info: Redefining \textls on input line 1368.
```

```
\MT@outer@kern=\dimen141
LaTeX Info: Redefining \textmicrotypecontext on input line 1988.
\MT@listname@count=\count198
(c:/texlive/2023/texmf-dist/tex/latex/microtype/microtype-pdftex.def
File: microtype-pdftex.def 2023/03/13 v3.1a Definitions specific to
pdftex (RS)

LaTeX Info: Redefining \lsstyle on input line 902.
LaTeX Info: Redefining \lslig on input line 902.
\MT@outer@space=\skip50
)
Package microtype Info: Loading configuration file microtype.cfg.
(c:/texlive/2023/texmf-dist/tex/latex/microtype/microtype.cfg
File: microtype.cfg 2023/03/13 v3.1a microtype main configuration file
(RS)
)) (c:/texlive/2023/texmf-dist/tex/latex/euler/euler.sty
Package: euler 1995/03/05 v2.5
Package: `euler' v2.5 <1995/03/05> (FJ and FMi)
LaTeX Font Info:    Redeclaring symbol font `letters' on input line 35.
LaTeX Font Info:    Encoding `OML' has changed to `U' for symbol font
(Font)              `letters' in the math version `normal' on input line
35.
LaTeX Font Info:    Overwriting symbol font `letters' in version `normal'
(Font)                  OML/cmm/m/it --> U/eur/m/n on input line 35.
LaTeX Font Info:    Encoding `OML' has changed to `U' for symbol font
(Font)              `letters' in the math version `bold' on input line
35.
LaTeX Font Info:    Overwriting symbol font `letters' in version `bold'
(Font)                  OML/cmm/b/it --> U/eur/m/n on input line 35.
LaTeX Font Info:    Overwriting symbol font `letters' in version `bold'
(Font)                  U/eur/m/n --> U/eur/b/n on input line 36.
LaTeX Font Info:    Redeclaring math symbol \Gamma on input line 47.
LaTeX Font Info:    Redeclaring math symbol \Delta on input line 48.
LaTeX Font Info:    Redeclaring math symbol \Theta on input line 49.
LaTeX Font Info:    Redeclaring math symbol \Lambda on input line 50.
LaTeX Font Info:    Redeclaring math symbol \Xi on input line 51.
LaTeX Font Info:    Redeclaring math symbol \Pi on input line 52.
LaTeX Font Info:    Redeclaring math symbol \Sigma on input line 53.
LaTeX Font Info:    Redeclaring math symbol \Upsilon on input line 54.
LaTeX Font Info:    Redeclaring math symbol \Phi on input line 55.
LaTeX Font Info:    Redeclaring math symbol \Psi on input line 56.
LaTeX Font Info:    Redeclaring math symbol \Omega on input line 57.
\symEulerFraktur=\mathgroup4
LaTeX Font Info:    Overwriting symbol font `EulerFraktur' in version
`bold'
(Font)                  U/euf/m/n --> U/euf/b/n on input line 63.
LaTeX Info: Redefining \oldstylenums on input line 85.
\symEulerScript=\mathgroup5
LaTeX Font Info:    Overwriting symbol font `EulerScript' in version
`bold'
(Font)                  U/eus/m/n --> U/eus/b/n on input line 93.
LaTeX Font Info:    Redeclaring math symbol \aleph on input line 97.
LaTeX Font Info:    Redeclaring math symbol \Re on input line 98.
LaTeX Font Info:    Redeclaring math symbol \Im on input line 99.
```

```
LaTeX Font Info:    Redeclaring math delimiter \vert on input line 101.
LaTeX Font Info:    Redeclaring math delimiter \backslash on input line
103.
LaTeX Font Info:    Redeclaring math symbol \neg on input line 106.
LaTeX Font Info:    Redeclaring math symbol \wedge on input line 108.
LaTeX Font Info:    Redeclaring math symbol \vee on input line 110.
LaTeX Font Info:    Redeclaring math symbol \setminus on input line 112.
LaTeX Font Info:    Redeclaring math symbol \sim on input line 113.
LaTeX Font Info:    Redeclaring math symbol \mid on input line 114.
LaTeX Font Info:    Redeclaring math delimiter \arrowvert on input line
116.
LaTeX Font Info:    Redeclaring math symbol \mathsection on input line
117.
\symEulerExtension=\mathgroup6
LaTeX Font Info:    Redeclaring math symbol \coprod on input line 125.
LaTeX Font Info:    Redeclaring math symbol \prod on input line 125.
LaTeX Font Info:    Redeclaring math symbol \sum on input line 125.
LaTeX Font Info:    Redeclaring math symbol \intop on input line 130.
LaTeX Font Info:    Redeclaring math symbol \ointop on input line 131.
LaTeX Font Info:    Redeclaring math symbol \braceld on input line 132.
LaTeX Font Info:    Redeclaring math symbol \bracerd on input line 133.
LaTeX Font Info:    Redeclaring math symbol \bracelu on input line 134.
LaTeX Font Info:    Redeclaring math symbol \braceru on input line 135.
LaTeX Font Info:    Redeclaring math symbol \infty on input line 136.
LaTeX Font Info:    Redeclaring math symbol \nearrow on input line 153.
LaTeX Font Info:    Redeclaring math symbol \searrow on input line 154.
LaTeX Font Info:    Redeclaring math symbol \nwarrow on input line 155.
LaTeX Font Info:    Redeclaring math symbol \swarrow on input line 156.
LaTeX Font Info:    Redeclaring math symbol \Leftrightarrow on input line
157.
LaTeX Font Info:    Redeclaring math symbol \Leftarrow on input line 158.
LaTeX Font Info:    Redeclaring math symbol \Rightarrow on input line
159.
LaTeX Font Info:    Redeclaring math symbol \leftrightarrow on input line
160.
LaTeX Font Info:    Redeclaring math symbol \leftarrow on input line 161.
LaTeX Font Info:    Redeclaring math symbol \rightarrow on input line
163.
LaTeX Font Info:    Redeclaring math delimiter \uparrow on input line
166.
LaTeX Font Info:    Redeclaring math delimiter \downarrow on input line
168.
LaTeX Font Info:    Redeclaring math delimiter \updownarrow on input line
170.
LaTeX Font Info:    Redeclaring math delimiter \Uparrow on input line
172.
LaTeX Font Info:    Redeclaring math delimiter \Downarrow on input line
174.
LaTeX Font Info:    Redeclaring math delimiter \Updownarrow on input line
176.
LaTeX Font Info:    Redeclaring math symbol \leftharpoonup on input line
177.
LaTeX Font Info:    Redeclaring math symbol \leftharpoondown on input
line 178.
```

```
LaTeX Font Info:    Redeclaring math symbol \rightharpoonup on input line
179.
LaTeX Font Info:    Redeclaring math symbol \rightharpoondown on input
line 180
.
LaTeX Font Info:    Redeclaring math delimiter \lbrace on input line 182.
LaTeX Font Info:    Redeclaring math delimiter \rbrace on input line 184.
\symcmmigroup=\mathgroup7
LaTeX Font Info:    Overwriting symbol font `cmmigroup' in version `bold'
(Font)                  OML/cmm/m/it --> OML/cmm/b/it on input line 200.
LaTeX Font Info:    Redeclaring math accent \vec on input line 201.
LaTeX Font Info:    Redeclaring math symbol \triangleleft on input line
202.
LaTeX Font Info:    Redeclaring math symbol \triangleright on input line
203.
LaTeX Font Info:    Redeclaring math symbol \star on input line 204.
LaTeX Font Info:    Redeclaring math symbol \lhook on input line 205.
LaTeX Font Info:    Redeclaring math symbol \rhook on input line 206.
LaTeX Font Info:    Redeclaring math symbol \flat on input line 207.
LaTeX Font Info:    Redeclaring math symbol \natural on input line 208.
LaTeX Font Info:    Redeclaring math symbol \sharp on input line 209.
LaTeX Font Info:    Redeclaring math symbol \smile on input line 210.
LaTeX Font Info:    Redeclaring math symbol \frown on input line 211.
LaTeX Font Info:    Redeclaring math accent \grave on input line 245.
LaTeX Font Info:    Redeclaring math accent \acute on input line 246.
LaTeX Font Info:    Redeclaring math accent \tilde on input line 247.
LaTeX Font Info:    Redeclaring math accent \ddot on input line 248.
LaTeX Font Info:    Redeclaring math accent \check on input line 249.
LaTeX Font Info:    Redeclaring math accent \breve on input line 250.
LaTeX Font Info:    Redeclaring math accent \bar on input line 251.
LaTeX Font Info:    Redeclaring math accent \dot on input line 252.
LaTeX Font Info:    Redeclaring math accent \hat on input line 254.
) (c:/texlive/2023/texmf-dist/tex/latex/merriweather/merriweather.sty
Package: merriweather 2022/09/20 (Bob Tennent) Supports
Merriweather(Sans) font
s for all LaTeX engines.
(c:/texlive/2023/texmf-dist/tex/generic/iftex/ifxetex.sty
Package: ifxetex 2019/10/25 v0.7 ifxetex legacy package. Use iftex
instead.
) (c:/texlive/2023/texmf-dist/tex/generic/iftex/ifluatex.sty
Package: ifluatex 2019/10/25 v1.5 ifluatex legacy package. Use iftex
instead.
) (c:/texlive/2023/texmf-dist/tex/latex/base/textcomp.sty
Package: textcomp 2020/02/02 v2.0n Standard LaTeX package
) (c:/texlive/2023/texmf-dist/tex/latex/xkeyval/xkeyval.sty
Package: xkeyval 2022/06/16 v2.9 package option processing (HA)
(c:/texlive/2023/texmf-dist/tex/generic/xkeyval/xkeyval.tex
(c:/texlive/2023/te
xmf-dist/tex/generic/xkeyval/xkvutils.tex
\XKV@toks=\toks22
\XKV@tempa@toks=\toks23
)
\XKV@depth=\count199
```

```
File: xkeyval.tex 2014/12/03 v2.7a key=value parser (HA)
)) (c:/texlive/2023/texmf-dist/tex/latex/base/fontenc.sty
Package: fontenc 2021/04/29 v2.0v Standard LaTeX package
) (c:/texlive/2023/texmf-dist/tex/latex/fontaxes/fontaxes.sty
Package: fontaxes 2020/07/21 v1.0e Font selection axes
LaTeX Info: Redefining \upshape on input line 29.
LaTeX Info: Redefining \itshape on input line 31.
LaTeX Info: Redefining \slshape on input line 33.
LaTeX Info: Redefining \swshape on input line 35.
LaTeX Info: Redefining \scshape on input line 37.
LaTeX Info: Redefining \sscshape on input line 39.
LaTeX Info: Redefining \ulcshape on input line 41.
LaTeX Info: Redefining \textsw on input line 47.
LaTeX Info: Redefining \textssc on input line 48.
LaTeX Info: Redefining \textulc on input line 49.
)) (c:/texlive/2023/texmf-dist/tex/latex/mathastext/mathastext.sty
Package: mathastext 2023/12/29 v1.3zb Use the text font in math mode
(JFB)

Package mathastext Info: Starting the math mode configuration.
\mst@exists@muskip=\muskip16
\mst@forall@muskip=\muskip17
\mst@prime@muskip=\muskip18
\mst@do@nonletters=\toks24
\mst@do@easynonletters=\toks25
\mst@do@az=\toks26
\mst@do@AZ=\toks27
\symmtoperatorfont=\mathgroup8
\symmtletterfont=\mathgroup9
(   mathastext:   ) ! and ?
(   mathastext:   ) punctuation: , . : ; and \colon
LaTeX Info: Redefining \relbar on input line 894.
LaTeX Info: Redefining \rightarrowfill on input line 897.
LaTeX Info: Redefining \leftarrowfill on input line 902.
(   mathastext:   ) + and =
LaTeX Info: Redefining \Relbar on input line 993.
(   mathastext:   ) adding = ; and + to \nfss@catcodes
(   mathastext:   ) parentheses ( ) [ ] and slash /
(   mathastext:   ) alldelims: < > \backslash \setminus | \vert \mid \{
\}
LaTeX Font Info:    Redeclaring math delimiter \backslash on input line
1039.
LaTeX Font Info:    Redeclaring math symbol \setminus on input line 1051.
LaTeX Info: Redefining \models on input line 1060.
(   mathastext:   ) \# \mathdollar \% \&
(   mathastext:   ) \imath and \jmath
LaTeX Font Info:    Overwriting math alphabet `\Mathnormalbold' in
version `nor
mal'
(Font)                  T1/Merriwthr-OsF/b/it --> T1/Merriwthr-OsF/b/it
on inpu
t line 2516.
LaTeX Font Info:    Overwriting math alphabet `\Mathnormalbold' in
version `bol
```

```
d'
(Font)              T1/Merriwthr-OsF/b/it --> T1/Merriwthr-OsF/b/it
on inpu
t line 2516.
LaTeX Font Info:    Overwriting symbol font `mtletterfont' in version
`normal'
(Font)              T1/Merriwthr-OsF/m/it --> T1/Merriwthr-OsF/m/it
on inpu
t line 2516.
LaTeX Font Info:    Overwriting symbol font `mtletterfont' in version
`bold'
(Font)              T1/Merriwthr-OsF/m/it --> T1/Merriwthr-OsF/b/it
on inpu
t line 2516.
LaTeX Font Info:    Overwriting symbol font `mtoperatorfont' in version
`normal
'
(Font)              T1/Merriwthr-OsF/m/n --> T1/Merriwthr-OsF/m/n on
input
line 2516.
LaTeX Font Info:    Overwriting symbol font `mtoperatorfont' in version
`bold'
(Font)              T1/Merriwthr-OsF/m/n --> T1/Merriwthr-OsF/b/n on
input
line 2516.
LaTeX Font Info:    Overwriting math alphabet `\Mathbf' in version
`normal'
(Font)              T1/Merriwthr-OsF/b/n --> T1/Merriwthr-OsF/b/n on
input
line 2516.
LaTeX Font Info:    Overwriting math alphabet `\Mathbf' in version `bold'
(Font)              T1/Merriwthr-OsF/b/n --> T1/Merriwthr-OsF/b/n on
input
line 2516.
LaTeX Font Info:    Overwriting math alphabet `\Mathit' in version
`normal'
(Font)              T1/Merriwthr-OsF/m/it --> T1/Merriwthr-OsF/m/it
on inpu
t line 2516.
LaTeX Font Info:    Overwriting math alphabet `\Mathit' in version `bold'
(Font)              T1/Merriwthr-OsF/m/it --> T1/Merriwthr-OsF/b/it
on inpu
t line 2516.
LaTeX Font Info:    Overwriting math alphabet `\Mathsf' in version
`normal'
(Font)              T1/MerriwthrSans-OsF/m/n --> T1/MerriwthrSans-
OsF/m/n o
n input line 2516.
LaTeX Font Info:    Overwriting math alphabet `\Mathsf' in version `bold'
(Font)              T1/MerriwthrSans-OsF/m/n --> T1/MerriwthrSans-
OsF/b/n o
n input line 2516.
LaTeX Font Info:    Overwriting math alphabet `\Mathtt' in version
`normal'
```

```
(Font)                  T1/lmtt/m/n --> T1/lmtt/m/n on input line 2516.
LaTeX Font Info:    Overwriting math alphabet `\Mathtt' in version `bold'
(Font)                  T1/lmtt/m/n --> T1/lmtt/b/n on input line 2516.
(   mathastext:   ) Latin letters in the `normal', resp. `bold',
(   mathastext:   ) math versions are now set up to use the fonts
(   mathastext:   ) T1/Merriwthr-OsF/m/it, resp. T1/Merriwthr-OsF/b/it.
(   mathastext:   ) Other characters (digits, ...) and \log-like names
will be
(   mathastext:   ) typeset with the n shape.
(   mathastext:   ) \hbar
(   mathastext:   ) minus as endash
(   mathastext:   ) The italic option is in effect.
(   mathastext:   ) \HUGE has been (re)-defined.
(   mathastext:   ) mathastext has declared larger sizes for subscripts.
(   mathastext:   ) To keep LaTeX defaults, use option
`defaultmathsizes'.

Package mathastext Info: Loading is complete.  You can now use
\Mathastext to
(mathastext)             modify the normal and bold math versions.  Use
it
(mathastext)             with optional argument or use \MTDeclareVersion
to
(mathastext)             declare additional math versions.
) (c:/texlive/2023/texmf-dist/tex/latex/relsize/relsize.sty
Package: relsize 2013/03/29 ver 4.1
) (c:/texlive/2023/texmf-dist/tex/latex/ragged2e/ragged2e.sty
Package: ragged2e 2023/06/22 v3.6 ragged2e Package
\CenteringLeftskip=\skip51
\RaggedLeftLeftskip=\skip52
\RaggedRightLeftskip=\skip53
\CenteringRightskip=\skip54
\RaggedLeftRightskip=\skip55
\RaggedRightRightskip=\skip56
\CenteringParfillskip=\skip57
\RaggedLeftParfillskip=\skip58
\RaggedRightParfillskip=\skip59
\JustifyingParfillskip=\skip60
\CenteringParindent=\skip61
\RaggedLeftParindent=\skip62
\RaggedRightParindent=\skip63
\JustifyingParindent=\skip64
) (c:/texlive/2023/texmf-dist/tex/latex/xcolor/xcolor.sty
Package: xcolor 2023/11/15 v3.01 LaTeX color extensions (UK)
(c:/texlive/2023/texmf-dist/tex/latex/graphics-cfg/color.cfg
File: color.cfg 2016/01/02 v1.6 sample color configuration
)
Package xcolor Info: Driver file: pdftex.def on input line 274.
(c:/texlive/2023/texmf-dist/tex/latex/graphics-def/pdftex.def
File: pdftex.def 2022/09/22 v1.2b Graphics/color driver for pdftex
) (c:/texlive/2023/texmf-dist/tex/latex/graphics/mathcolor.ltx)
Package xcolor Info: Model `cmy' substituted by `cmy0' on input line
1350.
Package xcolor Info: Model `hsb' substituted by `rgb' on input line 1354.
```

```
Package xcolor Info: Model `RGB' extended on input line 1366.
Package xcolor Info: Model `HTML' substituted by `rgb' on input line
1368.
Package xcolor Info: Model `Hsb' substituted by `hsb' on input line 1369.
Package xcolor Info: Model `tHsb' substituted by `hsb' on input line
1370.
Package xcolor Info: Model `HSB' substituted by `hsb' on input line 1371.
Package xcolor Info: Model `Gray' substituted by `gray' on input line
1372.
Package xcolor Info: Model `wave' substituted by `hsb' on input line
1373.
) (c:/texlive/2023/texmf-dist/tex/latex/colortbl/colortbl.sty
Package: colortbl 2024/02/20 v1.0g Color table columns (DPC)
(c:/texlive/2023/texmf-dist/tex/latex/tools/array.sty
Package: array 2023/10/16 v2.5g Tabular extension package (FMi)
\col@sep=\dimen142
\ar@mcellbox=\box52
\extrarowheight=\dimen143
\NC@list=\toks28
\extratabsurround=\skip65
\backup@length=\skip66
\ar@cellbox=\box53
)
\everycr=\toks29
\minrowclearance=\skip67
\rownum=\count266
) (c:/texlive/2023/texmf-dist/tex/latex/graphics/graphicx.sty
Package: graphicx 2021/09/16 v1.2d Enhanced LaTeX Graphics (DPC,SPQR)
(c:/texlive/2023/texmf-dist/tex/latex/graphics/graphics.sty
Package: graphics 2022/03/10 v1.4e Standard LaTeX Graphics (DPC,SPQR)
(c:/texlive/2023/texmf-dist/tex/latex/graphics/trig.sty
Package: trig 2021/08/11 v1.11 sin cos tan (DPC)
) (c:/texlive/2023/texmf-dist/tex/latex/graphics-cfg/graphics.cfg
File: graphics.cfg 2016/06/04 v1.11 sample graphics configuration
)
Package graphics Info: Driver file: pdftex.def on input line 107.
)
\Gin@req@height=\dimen144
\Gin@req@width=\dimen145
) (c:/texlive/2023/texmf-dist/tex/latex/xpatch/xpatch.sty
(c:/texlive/2023/texm
f-dist/tex/latex/l3kernel/expl3.sty
Package: expl3 2024-02-20 L3 programming layer (loader)
(c:/texlive/2023/texmf-dist/tex/latex/l3backend/l3backend-pdftex.def
File: l3backend-pdftex.def 2024-02-20 L3 backend support: PDF output
(pdfTeX)
\l__color_backend_stack_int=\count267
\l__pdf_internal_box=\box54
))
Package: xpatch 2020/03/25 v0.3a Extending etoolbox patching commands
(c:/texlive/2023/texmf-dist/tex/latex/l3packages/xparse/xparse.sty
Package: xparse 2024-02-18 L3 Experimental document command parser
)) (c:/texlive/2023/texmf-dist/tex/latex/environ/environ.sty
Package: environ 2014/05/04 v0.3 A new way to define environments
```

```
(c:/texlive/2023/texmf-dist/tex/latex/trimspaces/trimspaces.sty
Package: trimspaces 2009/09/17 v1.1 Trim spaces around a token list
)
\@envbody=\toks30
) (c:/texlive/2023/texmf-dist/tex/latex/lastpage/lastpage.sty
Package: lastpage 2023/10/14 v2.0e lastpage: 2.09 or 2e? (HMM)
(c:/texlive/2023/texmf-dist/tex/latex/lastpage/lastpage2e.sty
Package: lastpage2e 2023/10/14 v2.0e Decide which 2e lastpage version to
use (H
MM)
(c:/texlive/2023/texmf-dist/tex/latex/lastpage/lastpagemodern.sty
Package: lastpagemodern 2023-10-14 v2.0e Refers to last page's name (HMM;
JPG)
\c@lastpagecount=\count268
)
)) (c:/texlive/2023/texmf-dist/tex/latex/graphics/rotating.sty
Package: rotating 2016/08/11 v2.16d rotated objects in LaTeX
(c:/texlive/2023/texmf-dist/tex/latex/base/ifthen.sty
Package: ifthen 2022/04/13 v1.1d Standard LaTeX ifthen package (DPC)
)
\c@r@tfl@t=\count269
\rotFPtop=\skip68
\rotFPbot=\skip69
\rot@float@box=\box55
\rot@mess@toks=\toks31
) (c:/texlive/2023/texmf-dist/tex/latex/graphics/lscape.sty
Package: lscape 2020/05/28 v3.02 Landscape Pages (DPC)
) (c:/texlive/2023/texmf-dist/tex/latex/tools/afterpage.sty
Package: afterpage 2023/07/04 v1.08 After-Page Package (DPC)
\AP@output=\toks32
\AP@partial=\box56
\AP@footins=\box57
) (c:/texlive/2023/texmf-dist/tex/latex/textpos/textpos.sty
Package: textpos 2022/07/23 v1.10.1
Package textpos Info: choosing support for LaTeX3 on input line 60.
\TP@textbox=\box58
\TP@holdbox=\box59
\TPHorizModule=\dimen146
\TPVertModule=\dimen147
\TP@margin=\dimen148
\TP@absmargin=\dimen149
Grid set 16 x 16 = 37.34424pt x 52.81541pt
\TPboxrulesize=\dimen150
\TP@ox=\dimen151
\TP@oy=\dimen152
\TP@tbargs=\toks33
TextBlockOrigin set to 0pt x 0pt
) (c:/texlive/2023/texmf-dist/tex/latex/url/url.sty
\Urlmuskip=\muskip19
Package: url 2013/09/16  ver 3.4  Verb mode for urls, etc.
) (c:/texlive/2023/texmf-dist/tex/latex/newfloat/newfloat.sty
Package: newfloat 2023/10/01 v1.2 Defining new floating environments (AR)
Package newfloat Info: `rotating' package detected.
) (c:/texlive/2023/texmf-dist/tex/latex/mdframed/mdframed.sty
```

```
Package: mdframed 2013/07/01 1.9b: mdframed
(c:/texlive/2023/texmf-dist/tex/latex/kvoptions/kvoptions.sty
Package: kvoptions 2022-06-15 v3.15 Key value format for package options
(HO)
(c:/texlive/2023/texmf-dist/tex/generic/ltxcmds/ltxcmds.sty
Package: ltxcmds 2023-12-04 v1.26 LaTeX kernel commands for general use
(HO)
) (c:/texlive/2023/texmf-dist/tex/latex/kvsetkeys/kvsetkeys.sty
Package: kvsetkeys 2022-10-05 v1.19 Key value parser (HO)
)) (c:/texlive/2023/texmf-dist/tex/latex/zref/zref-abspage.sty
Package: zref-abspage 2023-09-14 v2.35 Module abspage for zref (HO)
(c:/texlive/2023/texmf-dist/tex/latex/zref/zref-base.sty
Package: zref-base 2023-09-14 v2.35 Module base for zref (HO)
(c:/texlive/2023/texmf-dist/tex/generic/infwarerr/infwarerr.sty
Package: infwarerr 2019/12/03 v1.5 Providing info/warning/error messages
(HO)
) (c:/texlive/2023/texmf-dist/tex/generic/kvdefinekeys/kvdefinekeys.sty
Package: kvdefinekeys 2019-12-19 v1.6 Define keys (HO)
) (c:/texlive/2023/texmf-dist/tex/generic/pdftexcmds/pdftexcmds.sty
Package: pdftexcmds 2020-06-27 v0.33 Utility functions of pdfTeX for
LuaTeX (HO
)
Package pdftexcmds Info: \pdf@primitive is available.
Package pdftexcmds Info: \pdf@ifprimitive is available.
Package pdftexcmds Info: \pdfdraftmode found.
) (c:/texlive/2023/texmf-dist/tex/generic/etexcmds/etexcmds.sty
Package: etexcmds 2019/12/15 v1.7 Avoid name clashes with e-TeX commands
(HO)
) (c:/texlive/2023/texmf-dist/tex/latex/auxhook/auxhook.sty
Package: auxhook 2019-12-17 v1.6 Hooks for auxiliary files (HO)
)
Package zref Info: New property list: main on input line 767.
Package zref Info: New property: default on input line 768.
Package zref Info: New property: page on input line 769.
)
\c@abspage=\count270
Package zref Info: New property: abspage on input line 67.
) (c:/texlive/2023/texmf-dist/tex/latex/needspace/needspace.sty
Package: needspace 2010/09/12 v1.3d reserve vertical space
)
\mdf@templength=\skip70
\c@mdf@globalstyle@cnt=\count271
\mdf@skipabove@length=\skip71
\mdf@skipbelow@length=\skip72
\mdf@leftmargin@length=\skip73
\mdf@rightmargin@length=\skip74
\mdf@innerleftmargin@length=\skip75
\mdf@innerrightmargin@length=\skip76
\mdf@innertopmargin@length=\skip77
\mdf@innerbottommargin@length=\skip78
\mdf@splittopskip@length=\skip79
\mdf@splitbottomskip@length=\skip80
\mdf@outermargin@length=\skip81
\mdf@innermargin@length=\skip82
```

```
\mdf@linewidth@length=\skip83
\mdf@innerlinewidth@length=\skip84
\mdf@middlelinewidth@length=\skip85
\mdf@outerlinewidth@length=\skip86
\mdf@roundcorner@length=\skip87
\mdf@footenotedistance@length=\skip88
\mdf@userdefinedwidth@length=\skip89
\mdf@needspace@length=\skip90
\mdf@frametitleaboveskip@length=\skip91
\mdf@frametitlebelowskip@length=\skip92
\mdf@frametitlerulewidth@length=\skip93
\mdf@frametitleleftmargin@length=\skip94
\mdf@frametitlerightmargin@length=\skip95
\mdf@shadowsize@length=\skip96
\mdf@extratopheight@length=\skip97
\mdf@subtitleabovelinewidth@length=\skip98
\mdf@subtitlebelowlinewidth@length=\skip99
\mdf@subtitleaboveskip@length=\skip100
\mdf@subtitlebelowskip@length=\skip101
\mdf@subtitleinneraboveskip@length=\skip102
\mdf@subtitleinnerbelowskip@length=\skip103
\mdf@subsubtitleabovelinewidth@length=\skip104
\mdf@subsubtitlebelowlinewidth@length=\skip105
\mdf@subsubtitleaboveskip@length=\skip106
\mdf@subsubtitlebelowskip@length=\skip107
\mdf@subsubtitleinneraboveskip@length=\skip108
\mdf@subsubtitleinnerbelowskip@length=\skip109
(c:/texlive/2023/texmf-dist/tex/latex/mdframed/md-frame-0.mdf
File: md-frame-0.mdf 2013/07/01\ 1.9b: md-frame-0
)
\mdf@frametitlebox=\box60
\mdf@footnotebox=\box61
\mdf@splitbox@one=\box62
\mdf@splitbox@two=\box63
\mdf@splitbox@save=\box64
\mdfsplitboxwidth=\skip110
\mdfsplitboxtotalwidth=\skip111
\mdfsplitboxheight=\skip112
\mdfsplitboxdepth=\skip113
\mdfsplitboxtotalheight=\skip114
\mdfframetitleboxwidth=\skip115
\mdfframetitleboxtotalwidth=\skip116
\mdfframetitleboxheight=\skip117
\mdfframetitleboxdepth=\skip118
\mdfframetitleboxtotalheight=\skip119
\mdffootnoteboxwidth=\skip120
\mdffootnoteboxtotalwidth=\skip121
\mdffootnoteboxheight=\skip122
\mdffootnoteboxdepth=\skip123
\mdffootnoteboxtotalheight=\skip124
\mdftotallinewidth=\skip125
\mdfboundingboxwidth=\skip126
\mdfboundingboxtotalwidth=\skip127
\mdfboundingboxheight=\skip128
```

```
\mdfboundingboxdepth=\skip129
\mdfboundingboxtotalheight=\skip130
\mdf@freevspace@length=\skip131
\mdf@horizontalwidthofbox@length=\skip132
\mdf@verticalmarginwhole@length=\skip133
\mdf@horizontalspaceofbox=\skip134
\mdfsubtitleheight=\skip135
\mdfsubsubtitleheight=\skip136
\c@mdfcountframes=\count272

****** mdframed patching \endmdf@trivlist

****** -- success******

\mdf@envdepth=\count273
\c@mdf@env@i=\count274
\c@mdf@env@ii=\count275
\c@mdf@zref@counter=\count276
Package zref Info: New property: mdf@pagevalue on input line 895.
) (c:/texlive/2023/texmf-dist/tex/latex/titlesec/titlesec.sty
Package: titlesec 2023/10/27 v2.16 Sectioning titles
\ttl@box=\box65
\beforetitleunit=\skip137
\aftertitleunit=\skip138
\ttl@plus=\dimen153
\ttl@minus=\dimen154
\ttl@toksa=\toks34
\titlewidth=\dimen155
\titlewidthlast=\dimen156
\titlewidthfirst=\dimen157
) (c:/texlive/2023/texmf-dist/tex/latex/koma-script/scrextend.sty
Package: scrextend 2023/07/07 v3.41 KOMA-Script package (extend other
classes w
ith features of KOMA-Script classes)
(c:/texlive/2023/texmf-dist/tex/latex/koma-script/scrkbase.sty
Package: scrkbase 2023/07/07 v3.41 KOMA-Script package (KOMA-Script-
dependent b
asics and keyval usage)
(c:/texlive/2023/texmf-dist/tex/latex/koma-script/scrbase.sty
Package: scrbase 2023/07/07 v3.41 KOMA-Script package (KOMA-Script-
independent
basics and keyval usage)
(c:/texlive/2023/texmf-dist/tex/latex/koma-script/scrlfile.sty
Package: scrlfile 2023/07/07 v3.41 KOMA-Script package (file load hooks)
(c:/texlive/2023/texmf-dist/tex/latex/koma-script/scrlfile-hook.sty
Package: scrlfile-hook 2023/07/07 v3.41 KOMA-Script package (using LaTeX
hooks)

(c:/texlive/2023/texmf-dist/tex/latex/koma-script/scrlogo.sty
Package: scrlogo 2023/07/07 v3.41 KOMA-Script package (logo)
)))
Applying: [2021/05/01] Usage of raw or classic option list on input line
252.
```

```
Already applied: [0000/00/00] Usage of raw or classic option list on
input line
 368.
))
Package scrextend Info: unexpected definition of `\@makefnmark'.
(scrextend)             Trying to patch it on input line 1762.
Package scrextend Info: patch seems to be successfull on input line 1762.
)

LaTeX Font Warning: Font shape `T1/cmr/m/n' in size <7.5> not available
(Font)              size <7> substituted on input line 69.

(c:/texlive/2023/texmf-dist/tex/latex/tools/calc.sty
Package: calc 2023/07/08 v4.3 Infix arithmetic (KKT,FJ)
\calc@Acount=\count277
\calc@Bcount=\count278
\calc@Adimen=\dimen158
\calc@Bdimen=\dimen159
\calc@Askip=\skip139
\calc@Bskip=\skip140
LaTeX Info: Redefining \setlength on input line 80.
LaTeX Info: Redefining \addtolength on input line 81.
\calc@Ccount=\count279
\calc@Cskip=\skip141
) (c:/texlive/2023/texmf-dist/tex/latex/geometry/geometry.sty
Package: geometry 2020/01/02 v5.9 Page Geometry
(c:/texlive/2023/texmf-dist/tex/generic/iftex/ifvtex.sty
Package: ifvtex 2019/10/25 v1.7 ifvtex legacy package. Use iftex instead.
)
\Gm@cnth=\count280
\Gm@cntv=\count281
\c@Gm@tempcnt=\count282
\Gm@bindingoffset=\dimen160
\Gm@wd@mp=\dimen161
\Gm@odd@mp=\dimen162
\Gm@even@mp=\dimen163
\Gm@layoutwidth=\dimen164
\Gm@layoutheight=\dimen165
\Gm@layouthoffset=\dimen166
\Gm@layoutvoffset=\dimen167
\Gm@dimlist=\toks35
) (c:/texlive/2023/texmf-dist/tex/latex/preprint/authblk.sty
Package: authblk 2001/02/27 1.3 (PWD)
\affilsep=\skip142
\@affilsep=\skip143
\c@Maxaffil=\count283
\c@authors=\count284
\c@affil=\count285
) (c:/texlive/2023/texmf-dist/tex/latex/footmisc/footmisc.sty
Package: footmisc 2023/07/05 v6.0f a miscellany of footnote facilities
\FN@temptoken=\toks36
\footnotemargin=\dimen168
\@outputbox@depth=\dimen169
```

```
Package footmisc Info: Declaring symbol style bringhurst on input line
696.
Package footmisc Info: Declaring symbol style chicago on input line 704.
Package footmisc Info: Declaring symbol style wiley on input line 713.
Package footmisc Info: Declaring symbol style lamport-robust on input
line 724.

Package footmisc Info: Declaring symbol style lamport* on input line 744.
Package footmisc Info: Declaring symbol style lamport*-robust on input
line 765
.
) (c:/texlive/2023/texmf-dist/tex/latex/fancyhdr/fancyhdr.sty
Package: fancyhdr 2022/11/09 v4.1 Extensive control of page headers and
footers

\f@nch@headwidth=\skip144
\f@nch@O@elh=\skip145
\f@nch@O@erh=\skip146
\f@nch@O@olh=\skip147
\f@nch@O@orh=\skip148
\f@nch@O@elf=\skip149
\f@nch@O@erf=\skip150
\f@nch@O@olf=\skip151
\f@nch@O@orf=\skip152
) (c:/texlive/2023/texmf-dist/tex/generic/alphalph/alphalph.sty
Package: alphalph 2019/12/09 v2.6 Convert numbers to letters (HO)
(c:/texlive/2023/texmf-dist/tex/generic/intcalc/intcalc.sty
Package: intcalc 2019/12/15 v1.3 Expandable calculations with integers
(HO)
))
\c@authorfn=\count286
(c:/texlive/2023/texmf-dist/tex/latex/abstract/abstract.sty
Package: abstract 2009/06/08 v1.2a configurable abstracts
\abstitleskip=\skip153
\absleftindent=\skip154
\absrightindent=\skip155
\absparindent=\skip156
\absparsep=\skip157
)
Package newfloat Info: New float `keypoints' with options
`placement=t!,name=kp
t' on input line 291.
\c@keypoints=\count287
\newfloat@ftype=\count288
Package newfloat Info: float type `keypoints'=8 on input line 291.
(c:/texlive/2023/texmf-dist/tex/latex/enumitem/enumitem.sty
Package: enumitem 2019/06/20 v3.9 Customized lists
\labelindent=\skip158
\enit@outerparindent=\dimen170
\enit@toks=\toks37
\enit@inbox=\box66
\enit@count@id=\count289
\enitdp@description=\count290
) (c:/texlive/2023/texmf-dist/tex/latex/quoting/quoting.sty
```

```
Package: quoting 2014/01/28 v0.1c Consolidated environment for displayed
text
\quo@toppartop=\skip159
) (c:/texlive/2023/texmf-dist/tex/latex/sttools/stfloats.sty
Package: stfloats 2017/03/27 v3.3 Improve float mechanism and
baselineskip sett
ings
\@dblbotnum=\count291
\c@dblbotnumber=\count292
) (c:/texlive/2023/texmf-dist/tex/latex/booktabs/booktabs.sty
Package: booktabs 2020/01/12 v1.61803398 Publication quality tables
\heavyrulewidth=\dimen171
\lightrulewidth=\dimen172
\cmidrulewidth=\dimen173
\belowrulesep=\dimen174
\belowbottomsep=\dimen175
\aboverulesep=\dimen176
\abovetopsep=\dimen177
\cmidrulesep=\dimen178
\cmidrulekern=\dimen179
\defaultaddspace=\dimen180
\@cmidla=\count293
\@cmidlb=\count294
\@aboverulesep=\dimen181
\@belowrulesep=\dimen182
\@thisruleclass=\count295
\@lastruleclass=\count296
\@thisrulewidth=\dimen183
) (c:/texlive/2023/texmf-dist/tex/latex/tools/tabularx.sty
Package: tabularx 2023/07/08 v2.11c `tabularx' package (DPC)
\TX@col@width=\dimen184
\TX@old@table=\dimen185
\TX@old@col=\dimen186
\TX@target=\dimen187
\TX@delta=\dimen188
\TX@cols=\count297
\TX@ftn=\toks38
)
\enitdp@tablenotes=\count298
(c:/texlive/2023/texmf-dist/tex/latex/caption/caption.sty
Package: caption 2023/08/05 v3.6o Customizing captions (AR)
(c:/texlive/2023/texmf-dist/tex/latex/caption/caption3.sty
Package: caption3 2023/07/31 v2.4d caption3 kernel (AR)
\caption@tempdima=\dimen189
\captionmargin=\dimen190
\caption@leftmargin=\dimen191
\caption@rightmargin=\dimen192
\caption@width=\dimen193
\caption@indent=\dimen194
\caption@parindent=\dimen195
\caption@hangindent=\dimen196
Package caption Info: Standard document class detected.
)
\c@caption@flags=\count299
```

```
\c@continuedfloat=\count300
Package caption Info: rotating package is loaded.
Package caption Info: scrextend package is loaded.
\caption@addmargin@hsize=\dimen197
\caption@addmargin@linewidth=\dimen198
) (c:/texlive/2023/texmf-dist/tex/latex/natbib/natbib.sty
Package: natbib 2010/09/13 8.31b (PWD, AO)
\bibhang=\skip160
\bibsep=\skip161
LaTeX Info: Redefining \cite on input line 694.
\c@NAT@ctr=\count301
)) (c:/texlive/2023/texmf-dist/tex/latex/siunitx/siunitx.sty
Package: siunitx 2024-02-15 v3.3.12 A comprehensive (SI) units package
\l__siunitx_number_uncert_offset_int=\count302
\l__siunitx_number_exponent_fixed_int=\count303
\l__siunitx_number_min_decimal_int=\count304
\l__siunitx_number_min_integer_int=\count305
\l__siunitx_number_round_precision_int=\count306
\l__siunitx_number_lower_threshold_int=\count307
\l__siunitx_number_upper_threshold_int=\count308
\l__siunitx_number_group_first_int=\count309
\l__siunitx_number_group_size_int=\count310
\l__siunitx_number_group_minimum_int=\count311
\l__siunitx_angle_tmp_dim=\dimen199
\l__siunitx_angle_marker_box=\box67
\l__siunitx_angle_unit_box=\box68
\l__siunitx_compound_count_int=\count312
(c:/texlive/2023/texmf-dist/tex/latex/translations/translations.sty
Package: translations 2022/02/05 v1.12 internationalization of LaTeX2e
packages
 (CN)
) (c:/texlive/2023/texmf-dist/tex/latex/amsmath/amstext.sty
Package: amstext 2021/08/26 v2.01 AMS text
(c:/texlive/2023/texmf-dist/tex/latex/amsmath/amsgen.sty
File: amsgen.sty 1999/11/30 v2.0 generic functions
\@emptytoks=\toks39
\ex@=\dimen256
))
\l__siunitx_table_tmp_box=\box69
\l__siunitx_table_tmp_dim=\dimen257
\l__siunitx_table_column_width_dim=\dimen258
\l__siunitx_table_integer_box=\box70
\l__siunitx_table_decimal_box=\box71
\l__siunitx_table_uncert_box=\box72
\l__siunitx_table_before_box=\box73
\l__siunitx_table_after_box=\box74
\l__siunitx_table_before_dim=\dimen259
\l__siunitx_table_carry_dim=\dimen260
\l__siunitx_unit_tmp_int=\count313
\l__siunitx_unit_position_int=\count314
\l__siunitx_unit_total_int=\count315
) (c:/texlive/2023/texmf-dist/tex/latex/float/float.sty
Package: float 2001/11/08 v1.3d Float enhancements (AL)
\c@float@type=\count316
```

```
\float@exts=\toks40
\float@box=\box75
\@float@everytoks=\toks41
\@floatcapt=\box76
) (c:/texlive/2023/texmf-dist/tex/latex/adjustbox/adjustbox.sty
Package: adjustbox 2022/10/17 v1.3a Adjusting TeX boxes (trim, clip, ...)
(c:/texlive/2023/texmf-dist/tex/latex/adjustbox/adjcalc.sty
Package: adjcalc 2012/05/16 v1.1 Provides advanced setlength with
multiple back
-ends (calc, etex, pgfmath)
) (c:/texlive/2023/texmf-dist/tex/latex/adjustbox/trimclip.sty
Package: trimclip 2020/08/19 v1.2 Trim and clip general TeX material
(c:/texlive/2023/texmf-dist/tex/latex/collectbox/collectbox.sty
Package: collectbox 2022/10/17 v0.4c Collect macro arguments as boxes
\collectedbox=\box77
)
\tc@llx=\dimen261
\tc@lly=\dimen262
\tc@urx=\dimen263
\tc@ury=\dimen264
Package trimclip Info: Using driver 'tc-pdftex.def'.
(c:/texlive/2023/texmf-dist/tex/latex/adjustbox/tc-pdftex.def
File: tc-pdftex.def 2019/01/04 v2.2 Clipping driver for pdftex
))
\adjbox@Width=\dimen265
\adjbox@Height=\dimen266
\adjbox@Depth=\dimen267
\adjbox@Totalheight=\dimen268
\adjbox@pwidth=\dimen269
\adjbox@pheight=\dimen270
\adjbox@pdepth=\dimen271
\adjbox@ptotalheight=\dimen272
(c:/texlive/2023/texmf-dist/tex/latex/ifoddpage/ifoddpage.sty
Package: ifoddpage 2022/10/18 v1.2 Conditionals for odd/even page
detection
\c@checkoddpage=\count317
) (c:/texlive/2023/texmf-dist/tex/latex/varwidth/varwidth.sty
Package: varwidth 2009/03/30 ver 0.92;  Variable-width minipages
\@vwid@box=\box78
\sift@deathcycles=\count318
\@vwid@loff=\dimen273
\@vwid@roff=\dimen274
)) (c:/texlive/2023/texmf-dist/tex/latex/hyperref/hyperref.sty
Package: hyperref 2024-01-20 v7.01h Hypertext links for LaTeX
(c:/texlive/2023/texmf-dist/tex/generic/pdfescape/pdfescape.sty
Package: pdfescape 2019/12/09 v1.15 Implements pdfTeX's escape features
(HO)
) (c:/texlive/2023/texmf-dist/tex/latex/hycolor/hycolor.sty
Package: hycolor 2020-01-27 v1.10 Color options for hyperref/bookmark
(HO)
) (c:/texlive/2023/texmf-dist/tex/latex/hyperref/nameref.sty
Package: nameref 2023-11-26 v2.56 Cross-referencing by name of section
(c:/texlive/2023/texmf-dist/tex/latex/refcount/refcount.sty
```

```
Package: refcount 2019/12/15 v3.6 Data extraction from label references
(HO)
) (c:/texlive/2023/texmf-
dist/tex/generic/gettitlestring/gettitlestring.sty
Package: gettitlestring 2019/12/15 v1.6 Cleanup title references (HO)
)
\c@section@level=\count319
)
\@linkdim=\dimen275
\Hy@linkcounter=\count320
\Hy@pagecounter=\count321
(c:/texlive/2023/texmf-dist/tex/latex/hyperref/pd1enc.def
File: pd1enc.def 2024-01-20 v7.01h Hyperref: PDFDocEncoding definition
(HO)
Now handling font encoding PD1 ...
... no UTF-8 mapping file for font encoding PD1
)
\Hy@SavedSpaceFactor=\count322
(c:/texlive/2023/texmf-dist/tex/latex/hyperref/puenc.def
File: puenc.def 2024-01-20 v7.01h Hyperref: PDF Unicode definition (HO)
Now handling font encoding PU ...
... no UTF-8 mapping file for font encoding PU
)
Package hyperref Info: Option `colorlinks' set `true' on input line 4062.
Package hyperref Info: Hyper figures OFF on input line 4179.
Package hyperref Info: Link nesting OFF on input line 4184.
Package hyperref Info: Hyper index ON on input line 4187.
Package hyperref Info: Plain pages OFF on input line 4194.
Package hyperref Info: Backreferencing OFF on input line 4199.
Package hyperref Info: Implicit mode ON; LaTeX internals redefined.
Package hyperref Info: Bookmarks ON on input line 4446.
\c@Hy@tempcnt=\count323
LaTeX Info: Redefining \url on input line 4784.
\XeTeXLinkMargin=\dimen276
(c:/texlive/2023/texmf-dist/tex/generic/bitset/bitset.sty
Package: bitset 2019/12/09 v1.3 Handle bit-vector datatype (HO)
(c:/texlive/2023/texmf-dist/tex/generic/bigintcalc/bigintcalc.sty
Package: bigintcalc 2019/12/15 v1.5 Expandable calculations on big
integers (HO
)
))
\Fld@menulength=\count324
\Field@Width=\dimen277
\Fld@charsize=\dimen278
Package hyperref Info: Hyper figures OFF on input line 6063.
Package hyperref Info: Link nesting OFF on input line 6068.
Package hyperref Info: Hyper index ON on input line 6071.
Package hyperref Info: backreferencing OFF on input line 6078.
Package hyperref Info: Link coloring ON on input line 6081.
Package hyperref Info: Link coloring with OCG OFF on input line 6088.
Package hyperref Info: PDF/A mode OFF on input line 6093.
(c:/texlive/2023/texmf-dist/tex/latex/base/atbegshi-ltx.sty
Package: atbegshi-ltx 2021/01/10 v1.0c Emulation of the original atbegshi
package with kernel methods
```

```
)
\Hy@abspage=\count325
\c@Item=\count326
\c@Hfootnote=\count327
)
Package hyperref Info: Driver (autodetected): hpdftex.
(c:/texlive/2023/texmf-dist/tex/latex/hyperref/hpdftex.def
File: hpdftex.def 2024-01-20 v7.01h Hyperref driver for pdfTeX
(c:/texlive/2023/texmf-dist/tex/latex/base/atveryend-ltx.sty
Package: atveryend-ltx 2020/08/19 v1.0a Emulation of the original
atveryend pac
kage
with kernel methods
)
\HyAnn@Count=\count328
\Fld@listcount=\count329
\c@bookmark@seq@number=\count330
(c:/texlive/2023/texmf-dist/tex/latex/rerunfilecheck/rerunfilecheck.sty
Package: rerunfilecheck 2022-07-10 v1.10 Rerun checks for auxiliary files
(HO)
(c:/texlive/2023/texmf-dist/tex/generic/uniquecounter/uniquecounter.sty
Package: uniquecounter 2019/12/15 v1.4 Provide unlimited unique counter
(HO)
)
Package uniquecounter Info: New unique counter `rerunfilecheck' on input
line 2
85.
)
\Hy@SectionHShift=\skip162
)
Package translations Info: No language package found. I am going to use
`englis
h' as default language. on input line 71.
LaTeX Font Info:    Trying to load font information for T1+Merriwthr-OsF
on inp
ut line 71.
(c:/texlive/2023/texmf-dist/tex/latex/merriweather/T1Merriwthr-OsF.fd
File: T1Merriwthr-OsF.fd 2020/08/30 (autoinst) Font definitions for
T1/Merriwth
r-OsF.
)
LaTeX Font Info:    Font shape `T1/Merriwthr-OsF/m/n' will be
(Font)              scaled to size 7.5pt on input line 71.
(./main.aux)
\openout1 = `main.aux'.

LaTeX Font Info:    Checking defaults for OML/cmm/m/it on input line 71.
LaTeX Font Info:    ... okay on input line 71.
LaTeX Font Info:    Checking defaults for OMS/cmsy/m/n on input line 71.
LaTeX Font Info:    ... okay on input line 71.
LaTeX Font Info:    Checking defaults for OT1/cmr/m/n on input line 71.
LaTeX Font Info:    ... okay on input line 71.
LaTeX Font Info:    Checking defaults for T1/cmr/m/n on input line 71.
LaTeX Font Info:    ... okay on input line 71.
```

```
LaTeX Font Info:    Checking defaults for TS1/cmr/m/n on input line 71.
LaTeX Font Info:    ... okay on input line 71.
LaTeX Font Info:    Checking defaults for OMX/cmex/m/n on input line 71.
LaTeX Font Info:    ... okay on input line 71.
LaTeX Font Info:    Checking defaults for U/cmr/m/n on input line 71.
LaTeX Font Info:    ... okay on input line 71.
LaTeX Font Info:    Checking defaults for PD1/pdf/m/n on input line 71.
LaTeX Font Info:    ... okay on input line 71.
LaTeX Font Info:    Checking defaults for PU/pdf/m/n on input line 71.
LaTeX Font Info:    ... okay on input line 71.
LaTeX Info: Redefining \microtypecontext on input line 71.
Package microtype Info: Applying patch `item' on input line 71.
Package microtype Info: Applying patch `toc' on input line 71.
Package microtype Info: Applying patch `eqnum' on input line 71.
Package microtype Info: Applying patch `footnote' on input line 71.
Package microtype Info: Applying patch `verbatim' on input line 71.
Package microtype Info: Generating PDF output.
Package microtype Info: Character protrusion enabled (level 2).
Package microtype Info: Using default protrusion set `alltext'.
Package microtype Info: Automatic font expansion enabled (level 2),
(microtype)             stretch: 20, shrink: 20, step: 1, non-selected.
Package microtype Info: Using default expansion set `alltext-nott'.
LaTeX Info: Redefining \showhyphens on input line 71.
Package microtype Info: No adjustment of tracking.
Package microtype Info: No adjustment of interword spacing.
Package microtype Info: No adjustment of character kerning.
Package microtype Info: Loading generic protrusion settings for font
family
(microtype)             `Merriwthr-OsF' (encoding: T1).
(microtype)             For optimal results, create family-specific
settings.
(microtype)             See the microtype manual for details.
LaTeX Font Info:    Redeclaring symbol font `operators' on input line 71.
LaTeX Font Info:    Encoding `OT1' has changed to `T1' for symbol font
(Font)              `operators' in the math version `normal' on input
line 71.
LaTeX Font Info:    Overwriting symbol font `operators' in version
`normal'
(Font)                  OT1/cmr/m/n --> T1/Merriwthr-OsF/m/up on input
line 71.

LaTeX Font Info:    Encoding `OT1' has changed to `T1' for symbol font
(Font)              `operators' in the math version `bold' on input line
71.
LaTeX Font Info:    Overwriting symbol font `operators' in version `bold'
(Font)                  OT1/cmr/bx/n --> T1/Merriwthr-OsF/m/up on input
line 71
.
LaTeX Font Info:    Overwriting symbol font `operators' in version `bold'
(Font)                  T1/Merriwthr-OsF/m/up --> T1/Merriwthr-OsF/b/up
on inpu
t line 71.
LaTeX Font Info:    Redeclaring math alphabet \mathbf on input line 71.
```

```
LaTeX Font Info:    Overwriting math alphabet `\mathbf' in version
`normal'
(Font)                  OT1/cmr/bx/n --> T1/Merriwthr-OsF/b/up on input
line 71
.
LaTeX Font Info:    Overwriting math alphabet `\mathbf' in version `bold'
(Font)                  OT1/cmr/bx/n --> T1/Merriwthr-OsF/b/up on input
line 71
.
LaTeX Font Info:    Redeclaring math alphabet \mathsf on input line 71.
LaTeX Font Info:    Overwriting math alphabet `\mathsf' in version
`normal'
(Font)                  OT1/cmss/m/n --> T1/MerriwthrSans-OsF/m/up on
input lin
e 71.
LaTeX Font Info:    Overwriting math alphabet `\mathsf' in version `bold'
(Font)                  OT1/cmss/bx/n --> T1/MerriwthrSans-OsF/m/up on
input li
ne 71.
LaTeX Font Info:    Redeclaring math alphabet \mathit on input line 71.
LaTeX Font Info:    Overwriting math alphabet `\mathit' in version
`normal'
(Font)                  OT1/cmr/m/it --> T1/Merriwthr-OsF/m/it on input
line 71
.
LaTeX Font Info:    Overwriting math alphabet `\mathit' in version `bold'
(Font)                  OT1/cmr/bx/it --> T1/Merriwthr-OsF/m/it on input
line 7
1.
LaTeX Font Info:    Redeclaring math alphabet \mathtt on input line 71.
LaTeX Font Info:    Overwriting math alphabet `\mathtt' in version
`normal'
(Font)                  OT1/cmtt/m/n --> T1/lmtt/m/up on input line 71.
LaTeX Font Info:    Overwriting math alphabet `\mathtt' in version `bold'
(Font)                  OT1/cmtt/m/n --> T1/lmtt/m/up on input line 71.
LaTeX Font Info:    Overwriting math alphabet `\mathsf' in version `bold'
(Font)                  T1/MerriwthrSans-OsF/m/up --> T1/MerriwthrSans-
OsF/b/up
 on input line 71.
LaTeX Font Info:    Overwriting math alphabet `\mathit' in version `bold'
(Font)                  T1/Merriwthr-OsF/m/it --> T1/Merriwthr-OsF/b/it
on inpu
t line 71.
\c@mv@tabular=\count331
\c@mv@boldtabular=\count332
(c:/texlive/2023/texmf-dist/tex/context/base/mkii/supp-pdf.mkii
[Loading MPS to PDF converter (version 2006.09.02).]
\scratchcounter=\count333
\scratchdimen=\dimen279
\scratchbox=\box79
\nofMPsegments=\count334
\nofMParguments=\count335
\everyMPshowfont=\toks42
\MPscratchCnt=\count336
```

```
\MPscratchDim=\dimen280
\MPnumerator=\count337
\makeMPintoPDFobject=\count338
\everyMPtoPDFconversion=\toks43
) (c:/texlive/2023/texmf-dist/tex/latex/epstopdf-pkg/epstopdf-base.sty
Package: epstopdf-base 2020-01-24 v2.11 Base part for package epstopdf
Package epstopdf-base Info: Redefining graphics rule for `.eps' on input
line 4
85.
(c:/texlive/2023/texmf-dist/tex/latex/latexconfig/epstopdf-sys.cfg
File: epstopdf-sys.cfg 2010/07/13 v1.3 Configuration of (r)epstopdf for
TeX Liv
e
))
Package newfloat Info: `float' package detected.
*geometry* driver: auto-detecting
*geometry* detected driver: pdftex
*geometry* verbose mode - [ preamble ] result:
* driver: pdftex
* paper: a4paper
* layout: <same size as paper>
* layoutoffset:(h,v)=(0.0pt,0.0pt)
* modes: includefoot twoside
* h-part:(L,W,R)=(54.64pt, 488.22787pt, 54.64pt)
* v-part:(T,H,B)=(66.0pt, 745.04684pt, 34.0pt)
* \paperwidth=597.50787pt
* \paperheight=845.04684pt
* \textwidth=488.22787pt
* \textheight=715.04684pt
* \oddsidemargin=-17.62999pt
* \evensidemargin=-17.62999pt
* \topmargin=-47.76999pt
* \headheight=17.5pt
* \headsep=24.0pt
* \topskip=10.0pt
* \footskip=30.0pt
* \marginparwidth=48.0pt
* \marginparsep=10.0pt
* \columnsep=18.0pt
* \skip\footins=22.0pt plus 2.0pt
* \hoffset=0.0pt
* \voffset=0.0pt
* \mag=1000
* \@twocolumntrue
* \@twosidetrue
* \@mparswitchtrue
* \@reversemarginfalse
* (1in=72.27pt=25.4mm, 1cm=28.453pt)

Package caption Info: Begin \AtBeginDocument code.
Package caption Info: float package is loaded.
Package caption Info: hyperref package is loaded.
Package caption Info: End \AtBeginDocument code.
```

```
(c:/texlive/2023/texmf-dist/tex/latex/translations/translations-basic-
dictionar
y-english.trsl
File: translations-basic-dictionary-english.trsl (english translation
file `tra
nslations-basic-dictionary')
)
Package translations Info: loading dictionary `translations-basic-
dictionary' f
or `english'. on input line 71.
Package hyperref Info: Link coloring ON on input line 71.
(./main.out) (./main.out)
\@outlinefile=\write3
\openout3 = `main.out'.

\@gscitedetails=\box80
\@gscitedetailsheight=\skip163
\@gsheadbox=\box81
\@gsheadboxheight=\skip164
LaTeX Font Info:    Font shape `T1/Merriwthr-OsF/b/n' will be
(Font)              scaled to size 6.5pt on input line 71.
LaTeX Font Info:    Calculating math sizes for size <7.5> on input line
71.

LaTeX Font Warning: Font shape `T1/Merriwthr-OsF/m/up' undefined
(Font)              using `T1/Merriwthr-OsF/m/n' instead on input line
71.

LaTeX Font Info:    Font shape `T1/Merriwthr-OsF/m/up' will be
(Font)              scaled to size 6.24973pt on input line 71.
LaTeX Font Info:    Font shape `T1/Merriwthr-OsF/m/up' will be
(Font)              scaled to size 5.24997pt on input line 71.
LaTeX Font Info:    Trying to load font information for U+eur on input
line 71.

(c:/texlive/2023/texmf-dist/tex/latex/amsfonts/ueur.fd
File: ueur.fd 2013/01/14 v3.01 Euler Roman
) (c:/texlive/2023/texmf-dist/tex/latex/microtype/mt-eur.cfg
File: mt-eur.cfg 2006/07/31 v1.1 microtype config. file: AMS Euler Roman
(RS)
)

LaTeX Font Warning: Font shape `OMS/cmsy/m/n' in size <7.5> not available
(Font)              size <7> substituted on input line 71.

LaTeX Font Info:    External font `cmex10' loaded for size
(Font)              <7.5> on input line 71.
LaTeX Font Info:    External font `cmex10' loaded for size
(Font)              <6.24973> on input line 71.
LaTeX Font Info:    External font `cmex10' loaded for size
(Font)              <5.24997> on input line 71.
LaTeX Font Info:    Trying to load font information for U+euf on input
line 71.
```

```
(c:/texlive/2023/texmf-dist/tex/latex/amsfonts/ueuf.fd
File: ueuf.fd 2013/01/14 v3.01 Euler Fraktur
) (c:/texlive/2023/texmf-dist/tex/latex/microtype/mt-euf.cfg
File: mt-euf.cfg 2006/07/03 v1.1 microtype config. file: AMS Euler
Fraktur (RS)

)
LaTeX Font Info:    Trying to load font information for U+eus on input
line 71.

(c:/texlive/2023/texmf-dist/tex/latex/amsfonts/ueus.fd
File: ueus.fd 2013/01/14 v3.01 Euler Script
) (c:/texlive/2023/texmf-dist/tex/latex/microtype/mt-eus.cfg
File: mt-eus.cfg 2006/07/28 v1.2 microtype config. file: AMS Euler Script
(RS)
)
LaTeX Font Info:    Trying to load font information for U+euex on input
line 71
.
(c:/texlive/2023/texmf-dist/tex/latex/amsfonts/ueuex.fd
File: ueuex.fd 2013/01/14 v3.01 Euler extra symbols
)

LaTeX Font Warning: Font shape `OML/cmm/m/it' in size <7.5> not available
(Font)              size <7> substituted on input line 71.

LaTeX Font Info:    Font shape `T1/Merriwthr-OsF/m/n' will be
(Font)              scaled to size 6.24973pt on input line 71.
LaTeX Font Info:    Font shape `T1/Merriwthr-OsF/m/n' will be
(Font)              scaled to size 5.24997pt on input line 71.
LaTeX Font Info:    Font shape `T1/Merriwthr-OsF/m/it' will be
(Font)              scaled to size 7.5pt on input line 71.
LaTeX Font Info:    Font shape `T1/Merriwthr-OsF/m/it' will be
(Font)              scaled to size 6.24973pt on input line 71.
LaTeX Font Info:    Font shape `T1/Merriwthr-OsF/m/it' will be
(Font)              scaled to size 5.24997pt on input line 71.
LaTeX Font Info:    Font shape `T1/Merriwthr-OsF/m/n' will be
(Font)              scaled to size 8.0pt on input line 71.
LaTeX Font Info:    Font shape `T1/Merriwthr-OsF/m/it' will be
(Font)              scaled to size 8.0pt on input line 71.
LaTeX Font Info:    Font shape `T1/Merriwthr-OsF/b/it' will be
(Font)              scaled to size 8.0pt on input line 71.
TextBlockOrigin set to 4pc+6.64pt x 4pc+6pt
<oup.pdf, id=125, 597.50829pt x 845.0471pt>
File: oup.pdf Graphic file (type pdf)
<use oup.pdf>
Package pdftex.def Info: oup.pdf  used on input line 81.
(pdftex.def)             Requested size: 41.03665pt x 58.038pt.
<gigascience-logo.pdf, id=126, 99.37125pt x 33.12375pt>
File: gigascience-logo.pdf Graphic file (type pdf)
<use gigascience-logo.pdf>
Package pdftex.def Info: gigascience-logo.pdf  used on input line 81.
(pdftex.def)             Requested size: 126.00902pt x 42.0pt.
```

```
Overfull \hbox (54.64pt too wide) in paragraph at lines 81--81
[][]
 []

LaTeX Font Info:    Font shape `T1/Merriwthr-OsF/m/n' will be
(Font)              scaled to size 14.0pt on input line 81.
LaTeX Font Info:    Font shape `T1/Merriwthr-OsF/m/n' will be
(Font)              scaled to size 8.99997pt on input line 81.
LaTeX Font Info:    Calculating math sizes for size <14> on input line
81.
LaTeX Font Info:    Font shape `T1/Merriwthr-OsF/m/up' will be
(Font)              scaled to size 14.0pt on input line 81.
LaTeX Font Info:    Font shape `T1/Merriwthr-OsF/m/up' will be
(Font)              scaled to size 11.66617pt on input line 81.
LaTeX Font Info:    Font shape `T1/Merriwthr-OsF/m/up' will be
(Font)              scaled to size 9.79996pt on input line 81.
LaTeX Font Info:    External font `cmex10' loaded for size
(Font)              <14> on input line 81.
LaTeX Font Info:    External font `cmex10' loaded for size
(Font)              <11.66617> on input line 81.
LaTeX Font Info:    External font `cmex10' loaded for size
(Font)              <9.79996> on input line 81.
LaTeX Font Info:    Font shape `T1/Merriwthr-OsF/m/n' will be
(Font)              scaled to size 11.66617pt on input line 81.
LaTeX Font Info:    Font shape `T1/Merriwthr-OsF/m/n' will be
(Font)              scaled to size 9.79996pt on input line 81.
LaTeX Font Info:    Font shape `T1/Merriwthr-OsF/m/it' will be
(Font)              scaled to size 14.0pt on input line 81.
LaTeX Font Info:    Font shape `T1/Merriwthr-OsF/m/it' will be
(Font)              scaled to size 11.66617pt on input line 81.
LaTeX Font Info:    Font shape `T1/Merriwthr-OsF/m/it' will be
(Font)              scaled to size 9.79996pt on input line 81.
LaTeX Font Info:    Font shape `T1/Merriwthr-OsF/b/n' will be
(Font)              scaled to size 18.0pt on input line 81.
LaTeX Font Info:    Font shape `T1/Merriwthr-OsF/m/n' will be
(Font)              scaled to size 13.0pt on input line 81.
LaTeX Font Info:    Calculating math sizes for size <13> on input line
81.
LaTeX Font Info:    Font shape `T1/Merriwthr-OsF/m/up' will be
(Font)              scaled to size 13.0pt on input line 81.
LaTeX Font Info:    Font shape `T1/Merriwthr-OsF/m/up' will be
(Font)              scaled to size 10.83287pt on input line 81.
LaTeX Font Info:    Font shape `T1/Merriwthr-OsF/m/up' will be
(Font)              scaled to size 9.09996pt on input line 81.

LaTeX Font Warning: Font shape `OMS/cmsy/m/n' in size <13> not available
(Font)              size <12> substituted on input line 81.

LaTeX Font Info:    External font `cmex10' loaded for size
(Font)              <13> on input line 81.
LaTeX Font Info:    External font `cmex10' loaded for size
(Font)              <10.83287> on input line 81.
LaTeX Font Info:    External font `cmex10' loaded for size
(Font)              <9.09996> on input line 81.
```

```
LaTeX Font Warning: Font shape `OML/cmm/m/it' in size <13> not available
(Font)              size <12> substituted on input line 81.

LaTeX Font Info:    Font shape `T1/Merriwthr-OsF/m/n' will be
(Font)              scaled to size 10.83287pt on input line 81.
LaTeX Font Info:    Font shape `T1/Merriwthr-OsF/m/n' will be
(Font)              scaled to size 9.09996pt on input line 81.
LaTeX Font Info:    Font shape `T1/Merriwthr-OsF/m/it' will be
(Font)              scaled to size 13.0pt on input line 81.
LaTeX Font Info:    Font shape `T1/Merriwthr-OsF/m/it' will be
(Font)              scaled to size 10.83287pt on input line 81.
LaTeX Font Info:    Font shape `T1/Merriwthr-OsF/m/it' will be
(Font)              scaled to size 9.09996pt on input line 81.
LaTeX Font Info:    Trying to load font information for TS1+Merriwthr-OsF
on in
put line 81.
(c:/texlive/2023/texmf-dist/tex/latex/merriweather/TS1Merriwthr-OsF.fd
File: TS1Merriwthr-OsF.fd 2020/08/30 (autoinst) Font definitions for
TS1/Merriw
thr-OsF.
)
LaTeX Font Info:    Font shape `TS1/Merriwthr-OsF/m/n' will be
(Font)              scaled to size 10.83287pt on input line 81.
Package microtype Info: Loading generic protrusion settings for font
family
(microtype)             `Merriwthr-OsF' (encoding: TS1).
(microtype)             For optimal results, create family-specific
settings.
(microtype)             See the microtype manual for details.
LaTeX Font Info:    Font shape `T1/Merriwthr-OsF/m/n' will be
(Font)              scaled to size 9.0pt on input line 81.
LaTeX Font Info:    Font shape `T1/Merriwthr-OsF/m/up' will be
(Font)              scaled to size 9.0pt on input line 81.
LaTeX Font Info:    Font shape `T1/Merriwthr-OsF/m/up' will be
(Font)              scaled to size 7.0pt on input line 81.
LaTeX Font Info:    Font shape `T1/Merriwthr-OsF/m/up' will be
(Font)              scaled to size 5.0pt on input line 81.
LaTeX Font Info:    External font `cmex10' loaded for size
(Font)              <9> on input line 81.
LaTeX Font Info:    External font `cmex10' loaded for size
(Font)              <7> on input line 81.
LaTeX Font Info:    External font `cmex10' loaded for size
(Font)              <5> on input line 81.
LaTeX Font Info:    Font shape `T1/Merriwthr-OsF/m/n' will be
(Font)              scaled to size 7.0pt on input line 81.
LaTeX Font Info:    Font shape `T1/Merriwthr-OsF/m/n' will be
(Font)              scaled to size 5.0pt on input line 81.
LaTeX Font Info:    Font shape `T1/Merriwthr-OsF/m/it' will be
(Font)              scaled to size 9.0pt on input line 81.
LaTeX Font Info:    Font shape `T1/Merriwthr-OsF/m/it' will be
(Font)              scaled to size 7.0pt on input line 81.
LaTeX Font Info:    Font shape `T1/Merriwthr-OsF/m/it' will be
(Font)              scaled to size 5.0pt on input line 81.
```

```
LaTeX Font Info:    Font shape `T1/Merriwthr-OsF/m/n' will be
(Font)              scaled to size 6.5pt on input line 81.
LaTeX Font Info:    Calculating math sizes for size <6.5> on input line
81.
LaTeX Font Info:    Font shape `T1/Merriwthr-OsF/m/up' will be
(Font)              scaled to size 6.5pt on input line 81.
LaTeX Font Info:    Font shape `T1/Merriwthr-OsF/m/up' will be
(Font)              scaled to size 5.41643pt on input line 81.
LaTeX Font Info:    Font shape `T1/Merriwthr-OsF/m/up' will be
(Font)              scaled to size 4.54997pt on input line 81.

LaTeX Font Warning: Font shape `OMS/cmsy/m/n' in size <6.5> not available
(Font)              size <6> substituted on input line 81.


LaTeX Font Warning: Font shape `OMS/cmsy/m/n' in size <5.41643> not
available
(Font)              size <5> substituted on input line 81.


LaTeX Font Warning: Font shape `OMS/cmsy/m/n' in size <4.54997> not
available
(Font)              size <5> substituted on input line 81.

LaTeX Font Info:    External font `cmex10' loaded for size
(Font)              <6.5> on input line 81.
LaTeX Font Info:    External font `cmex10' loaded for size
(Font)              <5.41643> on input line 81.
LaTeX Font Info:    External font `cmex10' loaded for size
(Font)              <4.54997> on input line 81.

LaTeX Font Warning: Font shape `OML/cmm/m/it' in size <6.5> not available
(Font)              size <6> substituted on input line 81.


LaTeX Font Warning: Font shape `OML/cmm/m/it' in size <5.41643> not
available
(Font)              size <5> substituted on input line 81.


LaTeX Font Warning: Font shape `OML/cmm/m/it' in size <4.54997> not
available
(Font)              size <5> substituted on input line 81.

LaTeX Font Info:    Font shape `T1/Merriwthr-OsF/m/n' will be
(Font)              scaled to size 5.41643pt on input line 81.
LaTeX Font Info:    Font shape `T1/Merriwthr-OsF/m/n' will be
(Font)              scaled to size 4.54997pt on input line 81.
LaTeX Font Info:    Font shape `T1/Merriwthr-OsF/m/it' will be
(Font)              scaled to size 6.5pt on input line 81.
LaTeX Font Info:    Font shape `T1/Merriwthr-OsF/m/it' will be
(Font)              scaled to size 5.41643pt on input line 81.
LaTeX Font Info:    Font shape `T1/Merriwthr-OsF/m/it' will be
(Font)              scaled to size 4.54997pt on input line 81.
```

```
LaTeX Font Info:    Font shape `TS1/Merriwthr-OsF/m/n' will be
(Font)              scaled to size 5.41643pt on input line 81.

Overfull \hbox (54.64pt too wide) in paragraph at lines 81--81
[][][]
 []

LaTeX Font Info:    Font shape `T1/Merriwthr-OsF/b/n' will be
(Font)              scaled to size 10.0pt on input line 81.
LaTeX Font Info:    Font shape `T1/Merriwthr-OsF/b/n' will be
(Font)              scaled to size 8.0pt on input line 81.

Overfull \hbox (54.64pt too wide) in paragraph at lines 81--81
[][][]
 []


LaTeX Warning: Optional argument of \twocolumn too tall on page 1.


Overfull \vbox (18.45389pt too high) has occurred while \output is active
[]


LaTeX Warning: Text page 1 contains only floats.


Overfull \vbox (18.45389pt too high) has occurred while \output is active
[]

LaTeX Font Info:    Font shape `T1/Merriwthr-OsF/m/n' will be
(Font)              scaled to size 7.8pt on input line 81.
LaTeX Font Info:    Font shape `T1/Merriwthr-OsF/b/n' will be
(Font)              scaled to size 7.8pt on input line 81.
[1{c:/texlive/2023/texmf-
var/fonts/map/pdftex/updmap/pdftex.map}{c:/texlive/202
3/texmf-
dist/fonts/enc/dvips/merriweather/merriwthr_posqbl.enc}{c:/texlive/2023
/texmf-dist/fonts/enc/dvips/merriweather/merriwthr_owzwzj.enc}


 <./oup.pdf> <./gigascience-logo.pdf>]
LaTeX Font Info:    Font shape `T1/Merriwthr-OsF/b/n' will be
(Font)              scaled to size 8.5pt on input line 83.
LaTeX Font Info:    Font shape `T1/Merriwthr-OsF/b/n' will be
(Font)              scaled to size 7.5pt on input line 84.

Package natbib Warning: Citation `Attwood2019-lb' on page 2 undefined on
input
line 84.


Package natbib Warning: Citation `Goodman2002-jb' on page 2 undefined on
input
```

```
line 84.


Package natbib Warning: Citation `Mitra2022-va' on page 2 undefined on
input li
ne 84.


Package natbib Warning: Citation `Singh2024-nn' on page 2 undefined on
input li
ne 84.


Package natbib Warning: Citation `Dhiman2024-hf' on page 2 undefined on
input l
ine 84.


Package natbib Warning: Citation `Wright2019-vg' on page 2 undefined on
input l
ine 84.


Package natbib Warning: Citation `Dabholkar_2021' on page 2 undefined on
input
line 84.


Package natbib Warning: Citation `Ras2021-kq' on page 2 undefined on
input line
 84.


Package natbib Warning: Citation `Chasapi2020-yz' on page 2 undefined on
input
line 84.


Package natbib Warning: Citation `Erxleben-Eggenhofer2023-kc' on page 2
undefin
ed on input line 84.


Package natbib Warning: Citation `Forero2016-vl' on page 2 undefined on
input l
ine 84.


Package natbib Warning: Citation `Mendy2014-li' on page 2 undefined on
input li
ne 84.
```

Package natbib Warning: Citation `Perez-Wohlfeil2018-ob' on page 2 undefined on
 input line 84.

Package natbib Warning: Citation `Ras2021-kq' on page 2 undefined on input line
 84.

Package natbib Warning: Citation `Attwood2019-lb' on page 2 undefined on input
line 85.

Package natbib Warning: Citation `Wilson_Sayres2018-cp' on page 2 undefined on
input line 85.

Package natbib Warning: Citation `Williams2019-fz' on page 2 undefined on input
 line 85.

Package natbib Warning: Citation `Katara2013-py' on page 2 undefined on input l
ine 85.

Package natbib Warning: Citation `Levine2014-bp' on page 2 undefined on input l
ine 85.

Package natbib Warning: Citation `Hwang2018-nl' on page 2 undefined on input li
ne 85.

Package natbib Warning: Citation `Wilson_Sayres2018-cp' on page 2 undefined on
input line 85.

Package natbib Warning: Citation `Navlakha2011-hf' on page 2 undefined on input
 line 85.

Package natbib Warning: Citation `Carey2018-cb' on page 2 undefined on input li
ne 86.

Package natbib Warning: Citation `Via2013-kg' on page 2 undefined on input line
86.

Package natbib Warning: Citation `Carey2018-cb' on page 2 undefined on input li
ne 86.

Package natbib Warning: Citation `Dudley2009-rd' on page 2 undefined on input l
ine 86.

Package natbib Warning: Citation `Jazayeri2015-qh' on page 2 undefined on input
line 86.

Package natbib Warning: Citation `Perkins1986-jw' on page 2 undefined on input
line 86.

Package natbib Warning: Citation `Shafto1986-oi' on page 2 undefined on input l
ine 86.

Package natbib Warning: Citation `Johnston2022-nd' on page 2 undefined on input
line 86.

Package natbib Warning: Citation `Hiltemann2023-mn' on page 2 undefined on inpu
t line 87.

Package natbib Warning: Citation `Rasche2022-ww' on page 2 undefined on input l
ine 87.

Package natbib Warning: Citation `Rasche2022-ww' on page 2 undefined on input l
ine 87.

Package natbib Warning: Citation `Hiltemann2023-mn' on page 2 undefined on inpu
t line 87.

Package natbib Warning: Citation `Bacon2022-jk' on page 2 undefined on input li
ne 87.

Package natbib Warning: Citation `Moreno2021-bm' on page 2 undefined on input l
ine 88.

LaTeX Font Info: Font shape `T1/Merriwthr-OsF/b/sl' in size <7.5> not availa
ble
(Font)   Font shape `T1/Merriwthr-OsF/b/it' tried instead on input l
ine 90.
LaTeX Font Info: Font shape `T1/Merriwthr-OsF/b/it' will be
(Font)   scaled to size 7.5pt on input line 90.
LaTeX Font Info: Font shape `T1/Merriwthr-OsF/b/n' will be
(Font)   scaled to size 7.0pt on input line 93.

Package natbib Warning: Citation `single-cell-scrna-case_alevin' on page 2 unde
fined on input line 99.

Package natbib Warning: Citation `single-cell-alevin-commandline' on page 2 und
efined on input line 99.

Package natbib Warning: Citation `single-cell-scrna-case_alevin-combine-dataset
s' on page 2 undefined on input line 101.

Package natbib Warning: Citation `single-cell-scrna-case_basic-pipeline' on pag
e 2 undefined on input line 103.

Package natbib Warning: Citation `single-cell-scrna-case-jupyter_basic-pipeline
' on page 2 undefined on input line 103.

Package natbib Warning: Citation `single-cell-scrna-case_FilterPlotandExplore_S
euratTools' on page 2 undefined on input line 105.

Package natbib Warning: Citation `single-cell-scrna-case_FilterPlotandExploreRS

```
tudio' on page 2 undefined on input line 105.


Package natbib Warning: Citation `single-cell-scrna-case_trajectories' on
page
2 undefined on input line 108.


Package natbib Warning: Citation `single-cell-scrna-case_JUPYTER-
trajectories'
on page 2 undefined on input line 108.


Package natbib Warning: Citation `single-cell-scrna-case_monocle3-
trajectories'
 on page 2 undefined on input line 110.


Package natbib Warning: Citation `single-cell-scrna-case_monocle3-
rstudio' on p
age 2 undefined on input line 110.


Package natbib Warning: Citation `Moreno2021-bm' on page 2 undefined on
input l
ine 115.

<Fig1.png, id=202, 534.798pt x 456.0237pt>
File: Fig1.png Graphic file (type png)
<use Fig1.png>
Package pdftex.def Info: Fig1.png  used on input line 118.
(pdftex.def)             Requested size: 235.11394pt x 200.48404pt.
LaTeX Font Info:    Font shape `T1/Merriwthr-OsF/m/n' will be
(Font)              scaled to size 6.0pt on input line 119.
LaTeX Font Info:    Font shape `T1/Merriwthr-OsF/b/n' will be
(Font)              scaled to size 6.0pt on input line 119.

Package natbib Warning: Citation `Gruning2017-yy' on page 2 undefined on
input
line 121.


Package natbib Warning: Citation `Baumer_Udwin_2015' on page 2 undefined
on inp
ut line 121.


Package natbib Warning: Citation `Kluyver2016jupyter' on page 2 undefined
on in
put line 121.

<Fig2.jpg, id=207, 681.747pt x 471.56175pt>
File: Fig2.jpg Graphic file (type jpg)
<use Fig2.jpg>
```

```
Package pdftex.def Info: Fig2.jpg  used on input line 126.
(pdftex.def)             Requested size: 235.11394pt x 162.6242pt.

Underfull \vbox (badness 10000) has occurred while \output is active []

LaTeX Font Info:    Font shape `T1/Merriwthr-OsF/m/it' will be
(Font)              scaled to size 7.8pt on input line 129.
[2 <./Fig1.png>]

Package natbib Warning: Citation `Satija2015-ne' on page 3 undefined on
input l
ine 131.


Package natbib Warning: Citation `Hao2024-tv' on page 3 undefined on
input line
 131.


Package natbib Warning: Citation `Hao2021-fj' on page 3 undefined on
input line
 131.


Package natbib Warning: Citation `Stuart2019-jr' on page 3 undefined on
input l
ine 131.


Package natbib Warning: Citation `Butler2018-tn' on page 3 undefined on
input l
ine 131.


Package natbib Warning: Citation `Cao2019-th' on page 3 undefined on
input line
 131.


Package natbib Warning: Citation `Wolf2018-kd' on page 3 undefined on
input lin
e 131.

<Fig3.jpg, id=222, 1043.69925pt x 517.935pt>
File: Fig3.jpg Graphic file (type jpg)
<use Fig3.jpg>
Package pdftex.def Info: Fig3.jpg  used on input line 134.
(pdftex.def)             Requested size: 235.11394pt x 116.67259pt.

Underfull \vbox (badness 10000) has occurred while \output is active []


Package natbib Warning: Citation `Patro2017-qg' on page 3 undefined on
input li
```

```
ne 146.


Package natbib Warning: Citation `Srivastava2019-wi' on page 3 undefined
on inp
ut line 146.


Package natbib Warning: Citation `Lun2019-id' on page 3 undefined on
input line
 148.


Package natbib Warning: Citation `Griffiths2018-px' on page 3 undefined
on inpu
t line 148.


Package natbib Warning: Citation `Lun2019-id' on page 3 undefined on
input line
 148.


Package natbib Warning: Citation `Moreno2022-zx' on page 3 undefined on
input l
ine 150.


Package natbib Warning: Citation `Love2020-km' on page 3 undefined on
input lin
e 152.


Package natbib Warning: Citation `Durinck2005-qy' on page 3 undefined on
input
line 154.


Package natbib Warning: Citation `Durinck2009-ue' on page 3 undefined on
input
line 154.


Package natbib Warning: Citation `Wolf2018-kd' on page 3 undefined on
input lin
e 158.


Package natbib Warning: Citation `igraph' on page 3 undefined on input
line 160
.
```

Package natbib Warning: Citation `Blondel2008-hz' on page 3 undefined on input
line 162.

Package natbib Warning: Citation `McKinney2010-es' on page 3 undefined on input
 line 164.

Package natbib Warning: Citation `Bates2000-ir' on page 3 undefined on input li
ne 168.

Package natbib Warning: Citation `dplyr' on page 3 undefined on input line 170.

Package natbib Warning: Citation `Wolf2018-kd' on page 3 undefined on input lin
e 172.

Package natbib Warning: Citation `Razavi2022-xx' on page 3 undefined on input l
ine 174.

Package natbib Warning: Citation `igraph' on page 3 undefined on input line 176
.

Package natbib Warning: Citation `Blondel2008-hz' on page 3 undefined on input
line 178.

Package natbib Warning: Citation `Harris2020-iz' on page 3 undefined on input l
ine 180.

Package natbib Warning: Citation `Hunter:2007' on page 3 undefined on input lin
e 182.

Package natbib Warning: Citation `Cao2019-th' on page 3 undefined on input line
 184.

Package natbib Warning: Citation `Virshup2021-rg' on page 3 undefined on input
line 186.

Package natbib Warning: Citation `Garnier2021-ov' on page 3 undefined on input
line 188.

Package natbib Warning: Citation `magrittr' on page 3 undefined on input line 1
90.

Package natbib Warning: Citation `rcpp' on page 3 undefined on input line
192.

Package natbib Warning: Citation `Durinck2005-qy' on page 3 undefined on input
line 194.

Package natbib Warning: Citation `Bacon2018-qy' on page 3 undefined on input li
ne 201.

Package natbib Warning: Citation `He2022-zp' on page 3 undefined on input line
202.

<Fig4.png, id=238, 5575.83125pt x 5761.525pt>
File: Fig4.png Graphic file (type png)
<use Fig4.png>
Package pdftex.def Info: Fig4.png used on input line 212.
(pdftex.def) Requested size: 235.11394pt x 242.90549pt.
[3 <./Fig2.jpg> <./Fig3.jpg>]
Underfull \vbox (badness 1019) has occurred while \output is active []

Package natbib Warning: Citation `vanDerMaaten2008' on page 4 undefined on inpu
t line 222.

Package natbib Warning: Citation `McInnes2018-ew' on page 4 undefined on input
line 222.

Package natbib Warning: Citation `Megill2021-sm' on page 4 undefined on
input l
ine 226.

<Fig5.png, id=248, 3827.29875pt x 2757.55219pt>
File: Fig5.png Graphic file (type png)
<use Fig5.png>
Package pdftex.def Info: Fig5.png used on input line 229.
(pdftex.def)   Requested size: 235.11394pt x 169.35913pt.
[4 <./Fig4.png>]

Package natbib Warning: Citation `Hafemeister2019-bg' on page 5 undefined
on in
put line 245.

Package natbib Warning: Citation `Tractenberg2019-bx' on page 5 undefined
on in
put line 245.

<Fig6.png, id=255, 3330.4425pt x 1775.433pt>
File: Fig6.png Graphic file (type png)
<use Fig6.png>
Package pdftex.def Info: Fig6.png used on input line 248.
(pdftex.def)   Requested size: 235.11394pt x 125.32246pt.

Package natbib Warning: Citation `Ko2020-lg' on page 5 undefined on input
line
256.

<Fig7.png, id=256, 2751.5297pt x 2864.45157pt>
File: Fig7.png Graphic file (type png)
<use Fig7.png>
Package pdftex.def Info: Fig7.png used on input line 261.
(pdftex.def)   Requested size: 235.11394pt x 244.72084pt.

Underfull \vbox (badness 10000) has occurred while \output is active []

[5 <./Fig5.png> <./Fig6.png> <./Fig7.png>]
<Fig8.png, id=268, 4372.335pt x 1945.2675pt>
File: Fig8.png Graphic file (type png)
<use Fig8.png>
Package pdftex.def Info: Fig8.png used on input line 282.
(pdftex.def)   Requested size: 235.11394pt x 104.60062pt.
<Fig9.png, id=270, 534.798pt x 456.0237pt>
File: Fig9.png Graphic file (type png)
<use Fig9.png>
Package pdftex.def Info: Fig9.png used on input line 294.
(pdftex.def)   Requested size: 235.11394pt x 200.48404pt.

Package natbib Warning: Citation `Hiltemann2023-mn' on page 6 undefined
on inpu
t line 297.

```
Package natbib Warning: Citation `Rasche2022-ww' on page 6 undefined on
input l
ine 297.


Package natbib Warning: Citation `Tractenberg2019-bx' on page 6 undefined
on in
put line 299.

<Fig10.jpg, id=272, 3021.48825pt x 1421.91225pt>
File: Fig10.jpg Graphic file (type jpg)
<use Fig10.jpg>
Package pdftex.def Info: Fig10.jpg  used on input line 302.
(pdftex.def)             Requested size: 235.11394pt x 110.63098pt.

Package natbib Warning: Citation `Hiltemann2023-mn' on page 6 undefined
on inpu
t line 305.


Package natbib Warning: Citation `Rasche2022-ww' on page 6 undefined on
input l
ine 305.


LaTeX Warning: `h' float specifier changed to `ht'.


Package natbib Warning: Citation `Cwtscwts2019-oz' on page 6 undefined on
input
 line 309.


Package natbib Warning: Citation `Papatheodorou2020-pg' on page 6
undefined on
input line 309.

<Fig11.jpg, id=274, 1155.71774pt x 594.42075pt>
File: Fig11.jpg Graphic file (type jpg)
<use Fig11.jpg>
Package pdftex.def Info: Fig11.jpg  used on input line 312.
(pdftex.def)             Requested size: 235.11394pt x 120.9228pt.

Underfull \hbox (badness 10000) in paragraph at lines 309--316
[]\T1/Merriwthr-OsF/m/n/7.5 (+20) MIGHTS ad-di-tion-ally pi-lots mul-ti-
ple im-
port strate-
 []

[6 <./Fig8.png> <./Fig9.png>]

Package natbib Warning: Citation `R' on page 7 undefined on input line
318.
```

```
Package natbib Warning: Citation `Goclowski_2024' on page 7 undefined on
input
line 318.


Package natbib Warning: Citation `Goecks2010-pa' on page 7 undefined on
input l
ine 320.


Package natbib Warning: Citation `Ouwerkerk2024-hv' on page 7 undefined
on inpu
t line 323.


Underfull \vbox (badness 3158) has occurred while \output is active []


Package natbib Warning: Citation
`Aron_Chauke_Ras_Panji_Johnston_Mulder_2021' o
n page 7 undefined on input line 326.

<Fig12.jpg, id=286, 510.9489pt x 403.9893pt>
File: Fig12.jpg Graphic file (type jpg)
<use Fig12.jpg>
Package pdftex.def Info: Fig12.jpg  used on input line 329.
(pdftex.def)             Requested size: 235.11394pt x 185.89897pt.

LaTeX Warning: `h' float specifier changed to `ht'.


Package natbib Warning: Citation `Dudley2009-rd' on page 7 undefined on
input l
ine 362.


Package natbib Warning: Citation `Perkins1986-jw' on page 7 undefined on
input
line 362.


Package natbib Warning: Citation `Cokelaer_Cohen-Boulakia_Lemoine_2023'
on page
 7 undefined on input line 362.


Package natbib Warning: Citation `Yang2017-kh' on page 7 undefined on
input lin
e 362.
```

```
Package natbib Warning: Citation `Bacon2018-qy' on page 7 undefined on
input li
ne 364.

[7 <./Fig10.jpg> <./Fig11.jpg> <./Fig12.jpg>]

Package natbib Warning: Citation `Bartlett2016-yy' on page 8 undefined on
input
 line 370.


Package natbib Warning: Citation `Garmire2021-mc' on page 8 undefined on
input
line 370.

LaTeX Font Info:    Font shape `TS1/Merriwthr-OsF/m/n' will be
(Font)              scaled to size 7.5pt on input line 383.

Underfull \hbox (badness 10000) in paragraph at lines 391--392
\T1/Merriwthr-OsF/m/n/7.5 (+20) All the tu-to-ri-als are avail-able at
ded-i-ca
ted Sin-
 []


Underfull \hbox (badness 10000) in paragraph at lines 391--392
\T1/Merriwthr-OsF/m/n/7.5 (+20) gle Cell sub-page of Galaxy Train-ing
Net-work
 []


Underfull \hbox (badness 10000) in paragraph at lines 391--392
\T1/Merriwthr-OsF/m/n/7.5 (+20) (GTN) at
[][]https://training.galaxyproject.org
/training-
 []


Underfull \hbox (badness 3240) in paragraph at lines 395--396
[]\T1/Merriwthr-OsF/m/n/7.5 (+20) The tu-to-ri-als com-prise many dif-
fer-ent t
ools that can be
 []


Underfull \hbox (badness 3930) in paragraph at lines 395--396
[][]\T1/Merriwthr-OsF/m/n/7.5 (+20) https://usegalaxy.org[][] and oth-
ers). The
 tool wrap-pers with
 []


Underfull \hbox (badness 10000) in paragraph at lines 395--396
```

```
\T1/Merriwthr-OsF/m/n/7.5 (+20) de-tailed in-for-ma-tion are stored at
the Gala
xy Tool-Shed
 []


Underfull \vbox (badness 1163) has occurred while \output is active []

[8]
No file main.bbl.
! Undefined control sequence.
l.444 \printbibliography

The control sequence at the end of the top line
of your error message was never \def'ed. If you have
misspelled it (e.g., `\hobx'), type `I' and the correct
spelling (e.g., `I\hbox'). Otherwise just continue,
and I'll forget about whatever was undefined.


Package natbib Warning: There were undefined citations.

[9

]
enddocument/afterlastpage: lastpage setting LastPage.
(./main.aux)
 ***********
LaTeX2e <2023-11-01> patch level 1
L3 programming layer <2020/03/25>
 ***********

LaTeX Font Warning: Size substitutions with differences
(Font)              up to 1.0pt have occurred.


LaTeX Font Warning: Some font shapes were not available, defaults
substituted.

Package rerunfilecheck Info: File `main.out' has not changed.
(rerunfilecheck)             Checksum:
009EE9759221F1FA84D1B2E6D31D4E2D;5835.
 )
Here is how much of TeX's memory you used:
 24960 strings out of 474121
 484258 string characters out of 5747949
 1973190 words of memory out of 5000000
 46220 multiletter control sequences out of 15000+600000
 1794660 words of font info for 568 fonts, out of 8000000 for 9000
 1141 hyphenation exceptions out of 8191
 123i,13n,131p,1931b,961s stack positions out of
10000i,1000n,20000p,200000b,200000s
<c:/texlive/2023/texmf-dist/fonts/type1/sorkin/merriweather/Merriwthr-
Bold.pf
```

b><c:/texlive/2023/texmf-dist/fonts/type1/sorkin/merriweather/Merriwthr-BoldIta
lic.pfb><c:/texlive/2023/texmf-dist/fonts/type1/sorkin/merriweather/Merriwthr-I
talic.pfb><c:/texlive/2023/texmf-dist/fonts/type1/sorkin/merriweather/Merriwthr
-Regular.pfb><c:/texlive/2023/texmf-dist/fonts/type1/public/lm/lmtt8.pfb>
Output written on main.pdf (9 pages, 12497291 bytes).
PDF statistics:
 350 PDF objects out of 1000 (max. 8388607)
 294 compressed objects within 3 object streams
 56 named destinations out of 1000 (max. 500000)
 194871 words of extra memory for PDF output out of 221844 (max. 10000000)

Manuscript

Click here to access/download;Manuscript;MIGHTS_GigaScience

GigaScience, 2023, 1–11

doi: xx.xxxx/xxxx
Manuscript in Preparation
Technical Note

TECHNICAL NOTE

Galaxy as a Gateway to Bioinformatics: Multi-Interface Galaxy Hands-on Training Suite (MIGHTS) for scRNA-seq

Camila Goclowski[1,†], Julia Jakiela[2,†], Tyler Collins[3], Saskia Hiltemann[4], Morgan Howells[5], Marisa Loach[6], Jonathan Manning[7], Pablo Moreno[8], Alex Ostrovsky[3], Helena Rasche[4], Mehmet Tekman[9], Graeme Tyson[10], Pavankumar Videm[11] and Wendi Bacon[6,*]

[1]University of Utah; Salt Lake City, Utah, United States of America and [2]University of Edinburgh School of Chemistry; Edinburgh, United Kingdom and [3]John Hopkins Medical Institution; Baltimore, Maryland, United States of America and [4]Erasmus Medical Center; Rotterdam, Zuid-Holland, Netherlands and [5]The Open University School of Computing & Communications; Milton Keynes, Cambridgeshire, United Kingdom and [6]The Open University School of Life, Health & Chemical Sciences; Milton Keynes, Cambridgeshire, United Kingdom and [7]EMBL-EBI; Hinxton, England and [8]Astrazeneca (2022 - Present) & EMBL-EBI (pre-2022); Hinxton, England and [9]University of Freiburg Division of Pharmacology and Toxicology; Freiburg im Breisgau, Baden-Württemberg, Germany and [10]The Open University; Milton Keynes, Cambridgeshire, United Kingdom and [11]University of Freiburg Department of Computer Science; Freiburg im Breisgau, Baden-Württemberg, Germany

[*]wendi.bacon@open.ac.uk
[†]Contributed equally.
[‡]*First and middle authors have been listed in the alphabetical order. Both first authors can put their name first on their CV.*

Abstract

Background. Bioinformatics is fundamental to biomedical sciences, but its mastery presents a steep learning curve for bench biologists and clinicians. Learning to code while analyzing data is difficult. The curve may be flattened by separating the two aspects and providing intermediate steps for budding bioinformaticians. Single-cell analysis is in great demand from biologists and biomedical scientists, as evidenced by the proliferation of training events, materials, and collaborative global efforts like the Human Cell Atlas. However, iterative analyses and un-standardized pipelines have made effective single-cell training a moving target. **Findings**. To address these challenges, we present a Multi-Interface Galaxy Hands-on Training Suite (MIGHTS) for scRNA-seq analysis, which offers parallel analytical methods using a graphical interface (buttons) or code. With clear, interoperable materials, MIGHTS facilitates smooth transitions between environments. Bridging the biologist-programmer gap, MIGHTS emphasizes interdisciplinary communication for effective learning at all levels. Real-world data analysis in MIGHTS promotes critical thinking and best practices, while FAIR data principles ensure validation of results. MIGHTS is freely available, hosted on the Galaxy Training Network, and leverages Galaxy interfaces for analyses in both settings. Given the ongoing popularity of Python-based (Scanpy) and R-based (Seurat, Monocle) scRNA-seq analyses, MIGHTS enables analyses using both. **Conclusions**. MIGHTS consists of 11 tutorials including recordings, slide-decks, and interactive visualizations, with a proven track record of sustainability via regular updates and community collaborations. Parallel pathways in MIGHTS enable concurrent training of scientists at any programming level, addressing the heterogeneous needs of novice bioinformaticians.

Key words: Training; STEM Education; Galaxy project; single-cell RNA-seq analysis; scRNA-seq; Bioinformatics; Reproducibility; Sustainability

Findings

Introduction

Although bioinformatics is critical to basic biological and applied biomedical research, there remains a shortage of scientists with bioinformatics expertise [[1]]. As access to computationally driven domains of biology continue to grow, bioinformatics plays an important role in biological discoveries [[2], [3], [4], [5]]. Thinking computationally about biological processes has been shown to produce more accurate models [[6]] and enhance problem solving [[7]]. But, bioinformatics requires many, often expensive, resources, such as computational infrastructure, maintenance, and training [[8]]. Financial barriers can limit access to training and research [[9], [10], [11], [12], [13]]. As such, many bioinformaticians rarely receive formal training in the field [[8]] and teaching bioinformatics is notably difficult. Integrating bioinformatics into undergraduate curriculums may address the current gap [[1], [14]]. Bioinformatics has been introduced in high schools, where it was shown to improve awareness, engagement, and self-efficacy of students: leading to increased interest in STEM careers [[15]]. Pharmaceutical companies need biomedical analysts [[16]], most employers in the life sciences prefer some competency in software analyses [[17]], and the use of bioinformatic analyses to characterize novel cell types and lineages [[18]] has surged. In response, institutes are beginning to teach foundational computing skills to biologists [[14], [19]]. Materials that focus on problem-solving, interactivity, and cooperative learning have demonstrated enhanced learning outcomes [[20]] and bioinformatics has effectively been taught by emphasizing interdisciplinary problem-solving [[21]]. To standardize training, a list of "rules" were identified to teach scientists to program: beginning with the end in mind, taking small steps forward, and focusing on individual tasks [[20]]. The 'end in mind' requires domain-specific understanding (i.e. identifying cell types via marker genes) while the individual tasks require programming skills (R, Python, troubleshooting, etc.). This duality forces participants to learn and apply two new skill-sets simultaneously [[22], [23], [24]]. The need to embed computing into science is not novel [[25]], but blending skills across disciplines is not without challenge [[26]]. The Galaxy Training Network (GTN) boasts tutorials for analysis across a range of fields, all publicly available and accessible by URL [[27]]. The GTN provides free training infrastructure to fast-track trainees via live courses in which trainers are available to monitor and assist participants [[28]]. This supports all, but especially low-resource institutions', engagement with bioinformatic training and has additionally been tested for native Spanish speakers [[28]]. Integrating these free resources into undergraduate curriculums has been successful [[27]], as training materials include interactive features based on research-backed pedagogies. Separation of learning components has previously been suggested as an effective method [[29]], but raises the question: how can coding and complex bioinformatic analyses be isolated from one another? Here, we directly address the need to separate the two for training. Leveraging the Galaxy Graphical User Interface (GUI) and the GTN, we present MIGHTS: a scRNA-seq tutorial suite enabling a smooth transition from data analysis in a button-based, user-friendly environment [[30]] to a more advanced, flexible coding environment. MIGHTS offers multiple routes of scRNA-seq analysis: allowing a button-based or coding-based version of the same, commonly published workflows. MIGHTS offers opportunities for a heterogeneous student population ranging from programming-friendly to programming-fearful, expanding access to critical skills required for effective bioinformatic analyses, biomedical, and life science research.

Methods

Multi-Environment

MIGHTS consists of 11 tutorials: six button-based (BB) and five in a programming environment (PE) (Table 1). The Galaxy GUI features "click-to-run" buttons which execute programming functions [[30]]. Users select and set parameters from dropdown lists and input boxes (Figure 1A and 1C). Each tool includes help text to guide users and describe the flexibility of the tool's function.

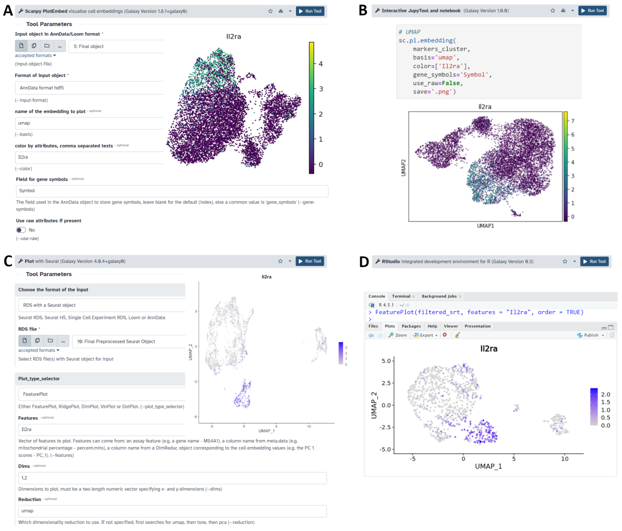

Figure 1. Performing the same step (plotting a marker gene: Il2ra on UMAP embedding) using different methods. A) Button-based Scanpy PlotEmbed tool. B) Running Scanpy code in Jupyter notebook. C) Button-based Plot with Seurat tool. D) Running Seurat code in RStudio.

Galaxy's interactive programming environments [[42]] are where the PE tutorials take place. Tutorials may be downloaded as Jupyter or RMarkdown notebooks [[43], [44]], or users can copy, paste, and run each executable code-containing cell from the PE text (Figure 1B and 1D). Jupyter and RMarkdown notebooks may be exported at the conclusion of each coded tutorial for easy reference or repetition.

Multi-Level

MIGHTS caters to three learning pathways: BB to PE, straight to PE, and PE with BB (Figure 2).

Compiled on: August 12, 2024.
Draft manuscript prepared by the author.

Table 1. Linked and cited tutorials featured in MIGHTS (n =11).

Analysis	Button-based (BB)	Programming Environment (PE)
Pre-processing	Generating a single-cell matrix using Alevin [[31]]	Generating a single-cell matrix using Alevin and combining datasets (bash + R) [[32]]
	Combining single-cell datasets after pre-processing [[33]]	
Plotting and Interpretation	Filter, plot and explore single-cell RNA-seq data with Scanpy [[34]]	Filter, plot and explore single-cell RNA-seq data with Scanpy (Python) [[35]]
	Filter, plot, and explore single-cell RNA-seq data with Seurat [[36]]	Filter, plot, and explore single-cell RNA-seq data with Seurat (R) [[37]]
Trajectories	Inferring single-cell trajectories with Scanpy [[38]]	Inferring single-cell trajectories with Scanpy (Python) [[39]]
	Inferring single-cell trajectories with Monocle3 [[40]]	Inferring single-cell trajectories with Monocle3 (R) [[41]]

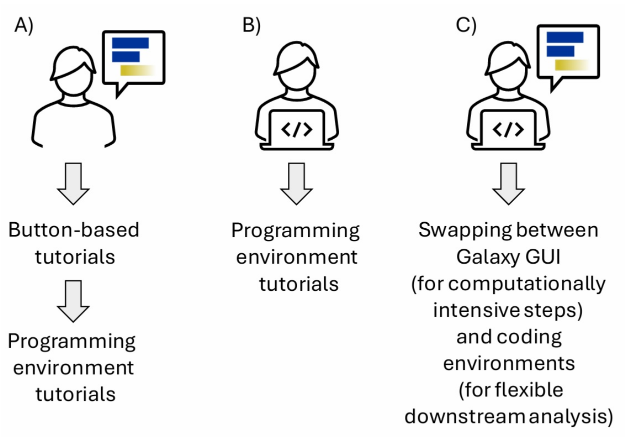

Figure 2. Representation of three possible user journeys using MIGHTS. A) A beginner starting from button-based (BB) tutorials who can then move to programming environment (PE). B) An experienced programmer who can start the analysis directly from the PE, skipping introductory BB tutorials. C) A skilled user who can optimize analyses by swapping between Galaxy GUI to perform computationally intensive steps, and a programming environment for more flexible analyses.

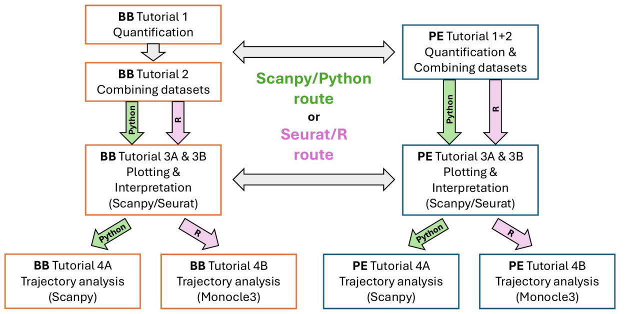

Figure 3. A diagram of the connections of tutorials. It highlights that the languages and packages used in BB and PE tutorials are consistent and allow moving between them easily.

In the first case, BB tutorials guide beginners through the key steps of scRNA-seq analysis, becoming familiar with the methods and learning to interpret results. Then, users repeat the analysis in the PE, focusing on programming skills, while becoming familiar with the languages and libraries commonly used in scRNA-seq analysis (Figure 2A). If a user has experience programming and wants a more flexible analysis, they may begin with the PE tutorials, learning methods with more advanced functionality (Figure 2B). Alternatively, experienced bioinformaticians may utilize Galaxy's Interactive Environments to learn new analyses or run computationally demanding steps that they are unable to run locally (Figure 2C).

Multi-Language

scRNA-seq analysis is commonly performed in both R-based (Seurat [[45], [46], [47], [48], [49]]; Monocle [[50]]) and Python-based (Scanpy [[51]]) environments. Therefore, parallel analyses were created across BB and PE and across programming languages–demonstrating multiple methods of analysis and data validation (Figure 3). Users may conduct a typical, full scRNA-seq analysis workflow in R or Python in addition to on a GUI or in a PE.

Research Relevant Skills

MIGHTS demonstrates the use of many frequently used data types and packages for scRNA-seq analyses (Table 2), preparing users with research relevant skills. It also improves users' employability and helps to reach scientists in various research groups, no matter the method they predominantly use.

Tutorials

Each tutorial begins with data import. The data used in MIGHTS comes from a published study by Bacon *et al.* 2018 [[72]], describing a mouse model of fetal growth restriction that is publicly available from the EMBL-EBI ArrayExpress under accession number E-MTAB-6945 and can also be browsed from Single Cell Expression Atlas. We continue working with the same data throughout the series to demonstrate analyses using different methods and tools. Our tutorials use real, uncurated data, which has simply been subsampled to enhance computational efficiency. The source data is the same, but each analysis inputs a different data file. The tutorials are designed to be completed in order, but they may be performed in any order–if a user wishes to learn how to cluster cells using Scanpy, for example, they may select the dedicated tutorial and start with the provided, pre-processed file. MIGHTS' full workflow consists of three sequential analyses aligning with standard scRNA-seq pipelines [[73]] and allowing users to compare results across methods.

Generating a single-cell matrix using Alevin and Combining Datasets

The first two tutorials demonstrate the transformation of a FASTA sequencing file into a count matrix (Figure 4). The BB tutorial describes principles of transcriptome quantification, while the PE tutorial introduces users to the many means of installing required packages. Users generate a transcript-to-gene map with FASTQ files, a GTF file, and a reference FASTA transcriptome. A Salmon index of the transcriptome is created, and a cell-by-gene count matrix is built using Alevin. The BB tutorial combines these two steps using one Galaxy tool. The BB tutorial demonstrates basic quality control checks including a description of the barcode rank plot "knee detection". The PE tutorial identifies empty droplets, adds cell and gene level metadata, and flags empty droplets based on transcript count. Droplet annotation is corrected for false discovery and the matrix is filtered before combining the datasets manually. Users save and export files while converting formats to

Table 2. MIGHTS tutorials with used packages and datatypes.

Analysis	Environment | Tutorial (Language)	Packages	Data Types
**Pre-processing**	BB | Generating a single-cell matrix using Alevin<br>BB | Combining single-cell datasets after pre-processing<br>PE | Generating a single-cell matrix using Alevin and combining datasets (bash + R)	Salmon[[52]] with Alevin [[53]]<br>dropletUtils [[54], [55]] (emptyDrops [[54]])<br>atlas-gene-annotation-manipulation [[56]]<br>tximeta [[57]] (PE)<br>biomaRt [[58], [59]] (PE)	FASTQ<br>FASTA<br>GTF<br>SingleCellExperiment Object<br>SummarizedExperiment (PE)<br>AnnData
**Plotting & Interpretation**	BB | Filter, plot and explore single-cell RNA-seq data (Scanpy)<br>PE | Filter, plot and explore single-cell RNA-seq data (Scanpy, Python)	Scanpy [[51]]<br>igraph [[60]] (PE)<br>louvain [[61]] (PE)<br>pandas [[62]] (PE)	AnnData
	BB | Filter, plot and explore single-cell RNA-seq data (Seurat)<br>PE | Filter, plot, and explore single-cell RNA-seq data (Seurat, R)	Seurat [34, 35, 36, 37, 38]<br>Matrix [[63]] (PE)<br>dplyr [[64]] (PE)	AnnData (for conversion to Seurat)<br>Seurat Object
**Trajectories**	BB | Inferring single-cell trajectories (Scanpy)<br>PE | Inferring single-cell trajectories (Scanpy, Python)	Scanpy [[51]]<br>fa2 [[65]] (PE)<br>igraph [[60]] (PE)<br>louvain [[61]] (PE)<br>numpy [[66]] (PE)<br>matplotlib [[67]] (PE)	AnnData
	BB | Inferring single-cell trajectories (Monocle3)<br>PE | Inferring single-cell trajectories (Monocle3, R)	Monocle [[50]]<br>anndata [[68]] (PE)<br>viridislite [[69]] (PE)<br>magrittr [[70]] (PE)<br>Rcpp [[71]] (PE)<br>biomaRt [[58]] (PE)	Cell Data Set<br>AnnData (for conversion to Cell Data Set in PE)

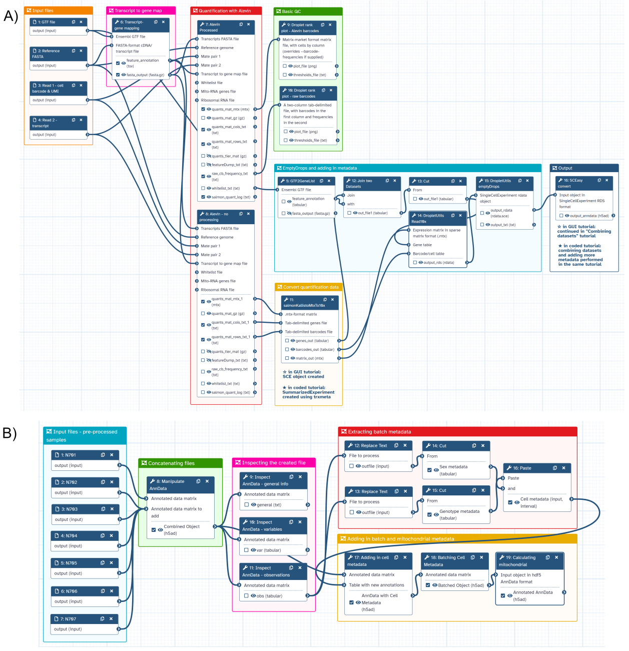

Figure 4. Galaxy generated workflows of the pre-processing tutorials. A) Workflow for tutorial "Generating a single-cell matrix using Alevin." Solid stars denote steps specific to the PE tutorial while unfilled stars represent BB specific ones. B) Workflow for tutorial "Combining single-cell datasets after pre-processing." All steps featured in the BB tutorial are combined with A's workflow for the PE.

SCE so they are compatible with downstream analyses. The BB tutorial incorporates metadata straight from a GTF file using a tool to extract gene names and IDs and to flag mitochondrial transcripts. The generated gene information is assigned to the matrix, which can then be transposed to be compatible with tools built for 10x Genomics software. EmptyDrops is then used to remove empty droplets. Much of the remaining suite emphasizes the use of AnnData compatible packages. To prepare users, tutorials conclude with one final format conversion from SCE to AnnData with the SCEasy tool. Once each of the objects have been converted, the BB user concatenates them with a Galaxy tool. The BB tutorial sets the user and their objects up for the next tutorial by adding a number of useful metrics to help visualize the data in the coming tutorial(s). Workflows for each tutorial topic are shown below in Figure 4.

Filter, Plot, and Explore with Scanpy

These tutorials filter and analyze the pre-processed matrix (Figure 5). PE users leverage Python via Jupyter Notebook.

The PE tutorial imports a raw AnnData file and demonstrates storage as a pandas dataframe, while users iteratively visualize data with violin and scatter plots to determine filtering thresholds. Users filter the data to remove technical artifacts and poor quality cells. The PE uses Boolean indexing rather than Scanpy's built-in functions. Users remove transcripts no longer expressed in more than three cells and are prompted to compare different thresholds for the filtering of genes.

Log normalization aligns gene expression along a normal distribution. The PE tutorial includes a description of how normalization works and what other methods exist. Variable genes are flagged for use in more computationally demanding steps. Scaling the data ensures all genes have equal variance and a zero mean, creating a matrix which is compatible with subsequent analyses.

Users reduce the dimensionality of the matrix to allow visualization and interpretation. Principal component analysis (PCA) is performed to calculate the most descriptive principal components (PCs). Users plot PCs against the standard variation they describe, visualizing how PCs relate to variance. The PCs are used to compute a k-nearest neighbors graph, storing a representation of connections between and across cells. Final dimensionality reductions are performed with t-distributed Stochastic Neighbor Embedding (tSNE) [[74]] and Uniform Manifold Approximation and Projection (UMAP) [[75]] - both methods reducing the data down to two dimensions for visualization.

Scanpy's clustering function(s) assign each cell to a cluster based on transcriptome similarities. The tutorials describe clustering algorithms and prompt users to experiment with different resolutions, adjusting so the assigned clusters visually represent what is understood to be biologically accurate. Scanpy's rank_genes_groups identifies the most representative transcripts for each cluster and genotype and PE users transform the output into a data frame.

Users visualize all three dimensionality reductions, different clustering resolutions, and the expression of marker genes. A table of marker genes per cell type from the literature is provided so that the user may inspect their expression patterns and map them to the correct cluster(s). Users label each cluster with a cell type, and each plot is saved into the history or notebook to be exported for downloading. BB users are additionally introduced to the CELLxGENE [[76]] tool: an interactive environment on Galaxy for visualizing and exploring scRNA-seq data. Workflow is shown below in Figure 5.

Filter, Plot, and Explore with Seurat

These tutorials closely resemble the workflows of the preceding Scanpy ones, this time making use of the R package, Seurat. The workflows teach users the basics of scRNA-seq data analysis includ-

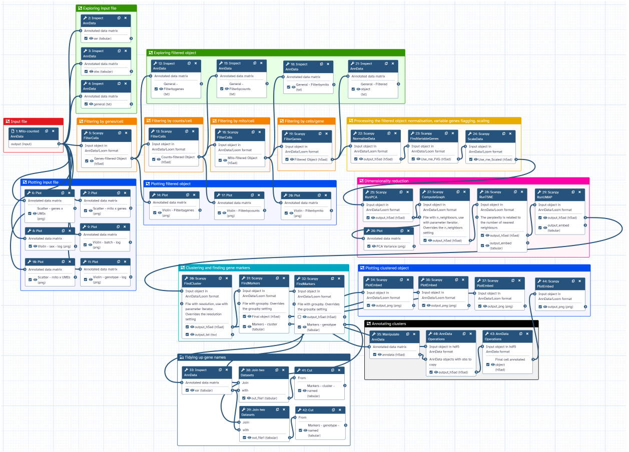

Figure 5. Workflows of plotting & interpretation tutorials: Filter, Plot, and Explore with Scanpy. Features creation of single-cell objects, normalizing data, identifying variable genes, performing dimensionality reduction, identifying clusters, finding marker genes, and interpreting plots.

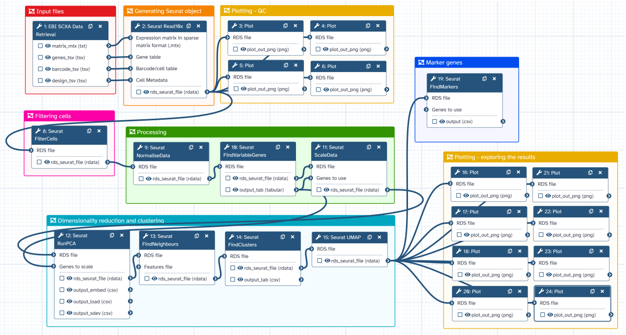

Figure 6. Workflow of Filter, Plot, and Explore tutorial with Seurat. Features generation of a Seurat object, quality control plots, filtering cells, processing, dimensionality reduction, clustering, finding marker genes and creating many plots to analyze the results.

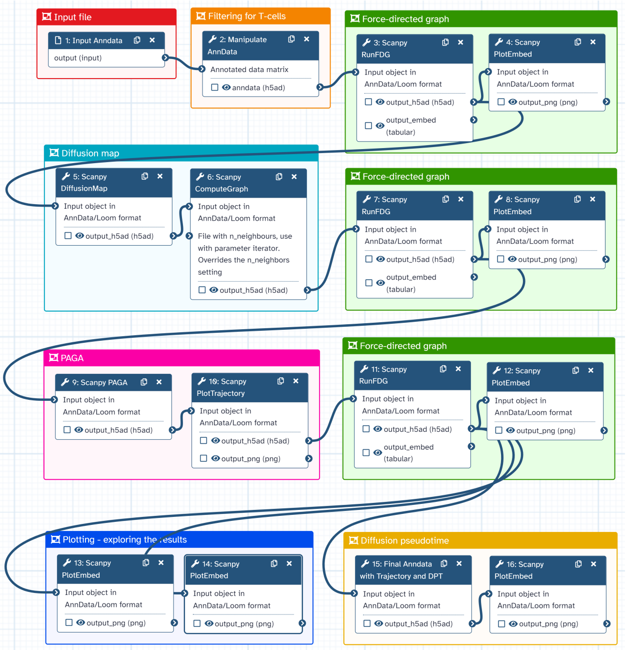

Figure 7. Workflow of inferring trajectories with Scanpy tutorial. Features methods such as force-directed graphs, diffusion maps and PAGA used to infer the cells trajectory in pseudotime.

ing typical preprocessing, basic visualization, and exploration.

Users import raw counts in both the PE and BB pathways. PE users transition to Galaxy's Interactive RStudio environment, where they are shown how to set up an environment, and given an explanation of how and why packages must be loaded prior to use, as well as how to use Galaxy's gx_get() function. Users manually change the column names of the experimental design data such that Seurat may properly read it.

Users generate a Seurat object: BB users with Seurat's Read10X function, and PE users by manually applying barcode and feature labels to the matrix for input to Seurat's CreateSeuratObject function. Each method is accompanied by descriptions of the alternatives for creating the same Seurat object.

Users apply cell level metadata to their objects. PE users add percent of gene expression (per cell) mapping to the mitochondrial genome—a useful parameter for quality control. Tools are being updated to enable BB users to do the same.

Users produce and interpret quality control plots to choose filtering thresholds: assessing potential confounds in the data and gaining an understanding of how different variables drive it. The purpose and theory behind commonly used filtering parameters are described so that users may bring the same (or different) strategies to their own analyses. PE users are additionally shown how to preview the number of cells which would be included based on their choice of filtering parameters.

Both users subset their Seurat object—removing cells outside the chosen threshold(s). PE users additionally remove genes that are now expressed at such low frequencies that they will not contribute biological insight.

Next, users process their filtered object. In the BB, processing of the data includes sequentially normalizing the data, identifying variable features, and scaling. In a more recent update to Seurat's workflow, they introduced the SCTransform function [[77] [78]], which combinatorially conducts the three aforementioned steps in a manner optimized for downstream analyses. SCTransform is used in the PE tutorial while the BB tutorial follows a similar workflow to the one originally published by Seurat. Both users subsequently cover dimensionality reduction via PCA, deciding on the number of PCs to use, finding neighbors, identifying clusters, and UMAP before guided visualization and exploration of the data.

Inferring single-cell trajectories with Scanpy

Trajectory inferences (TI), or pseudotime analysis, provides an alternative means of grouping cells, although not all TI algorithms are the best fit for all datasets. These parallel tutorials conduct a typical TI pipeline using Galaxy buttons or in a Python coded environment.

Tutorials are significantly based on Scanpy documentation, beginning by importing an annotated AnnData object into Galaxy. Users filter the data, retaining a single cell type. The PE tutorial demonstrates installation of modules before transferring their h5ad data to their Jupyter Notebook with the Galaxy-Jupyter cross-talk feature.

Users calculate force directed graphs (FDGs): representing the data more appropriately for TI than the previously generated tSNE or UMAP visualizations [[79]]. Optionally, they may create diffusion maps: which can be used in place of PCs to re-compute nearest neighbors visualized in the FDGs.

Both BB and PE users order cells in pseudotime using Scanpy's diffusion approach, which accepts root cluster assignment, indicating to the algorithm which population of cells the trajectory begins with. Users visualize inferred trajectories colored by pseudotime, save, and export their data, plots, and notebook. Users are encouraged to consider other changes across the identified trajectories beyond the scope of the tutorial.

Inferring single-cell trajectories with Monocle3

Similarly to the aforementioned, the Monocle3 tutorials teaches users to conduct trajectory inference (Figure 8).These tutorials demonstrate the variability that may arise when trajectories are inferred by different algorithms–this time using the algorithms employed by Monocle3. PE users can implement RStudio or Jupyter Notebook through Galaxy's Interactive Environments.

PE users are shown the installation of necessary libraries and modules, they import a filtered AnnData object, and familiarize themselves with the data's structure. They extract the expression matrix, cell, and gene metadata, and prepare them for generation of a Cell Data Set (CDS) object–Monocle's preferred data type–with format and column name changes, as well as transposition. BB users may import a CDS file ready for downstream analysis in Monocle or the precursor files to create a CDS manually.

PE users use the BioMart database to retrieve gene symbols and associated gene IDs. Although not necessary to complete the tutorial's workflow, this ability is of use to users analyzing their own data.

Users preprocess with Monocle3 beginning with dimensionality reduction. PCA is the method used in these tutorials although Latent Semantic Indexing (LSI), UMAP, and tSNE options are also available. PE users visualize each PC in relation to gene variance: to identify how many PCs are needed to capture appropriate variability. Users are provided with visualizations of the output data given different choices in PC.

BB users plot the data in a PCA space, visualizing the effects of various experimental design variables. PE users may optionally correct for batch effects and enjoy customizable plots for a more tailored analysis prior to final dimensionality reduction.

Users cluster the data and the tutorial describes the difference between clusters and partitions. The PE tutorial additionally demonstrates manual partitioning of cells, important for reliable trajectory inference.

The PE tutorial demonstrates three combined means of assigning cell types to the clusters–a supervised, unsupervised, and automated method. Users then infer trajectories relying on Monocle's trajectory graph. Once cells have been ordered in pseudotime starting from the root cell, users visualize the cells coloured by pseudotime. BB users end here, comparing the results of the Monocle3 derived trajectory with the Scanpy algorithm's.

PE users are presented with more options for differential expression analysis, visualizing results, identifying a visualization method suited for them, and exporting plots, data, and their Python, or RStudio, notebook. Workflow is demonstrated below in Figure 8.

Discussion

We present MIGHTS, a Multi-Interface Galaxy Hands-on Training Suite, where users may embark on three possible learning trajectories: (1) first learning to analyze scRNA-seq data with buttons in a GUI and subsequently performing the same, more flexible analysis in a programming environment, (2) learning to run the code behind commonly published scRNA-seq analyses, or (3) supplement their pre-existing analyses and skills with Galaxy tools.

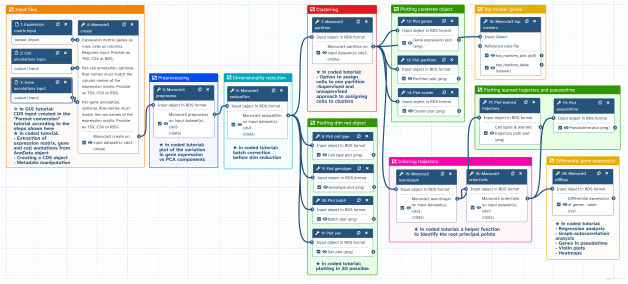

Figure 8. Workflow of Monocle3 inferring trajectories tutorial. Features data type changes for package compatibility, Monocle-specific preprocessing, and trajectory inference on a CellDataSet (CDS) object, followed by differential gene expression.

MIGHTS performs analysis from raw reads, guiding users through filtering, normalization, dimensionality reduction, quality assessment, and biological interpretation. The suite demonstrates clustering, annotation, and trajectory analysis for a well rounded scRNA-seq skill set. Each analysis is demonstrated using methods based on different packages, libraries, and programming languages with the hope that MIGHTS will prepare users to conduct their own more complex analyses.

Training Features

Users may start at any step by importing pre-processed input files, using output files from the preceding tutorial, or their own data. Regardless, the analyses will be replicated across languages, methods and starting points (Figure 9) allowing users to follow the trajectory best suited for their skill level. Each tutorial builds on the preceding, with no behind-the-scenes data formatting or annotation required between tutorials. With visual examples and examinations of varying data types, live training courses found that trainees who performed tutorials during the day could successfully apply the analyses to their own data in the evenings [[27], [28]].

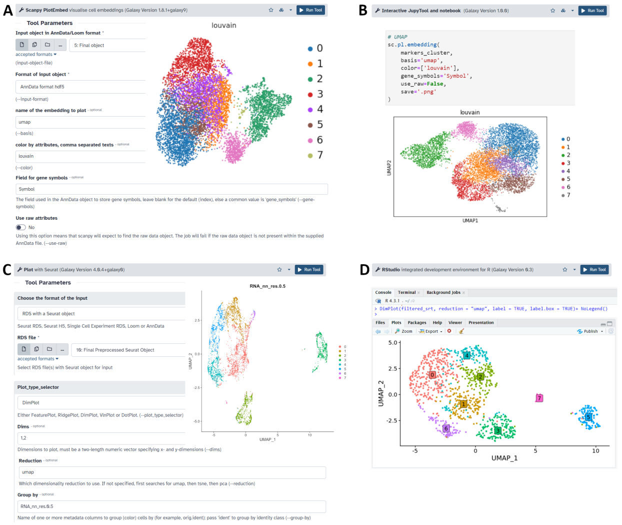

Figure 9. The final 'cluster plot' as an output of plotting & interpretation tutorials across four paths: BB tutorial with Scanpy, PE (Jupyter notebook) with Scanpy, BB tutorial with Seurat, and PE (RStudio) tutorial with Seurat.

Learning how to set parameters has long been a difficulty in bioinformatics training [[78]]. By highlighting parameters that are adjusted often, users learn to prioritize what would otherwise be extensive lists of decision making. These 'Decision-Time' features enable training for individuals and groups: with the option to vary parameter values and compare results (Figure 10). Testing of this feature has shown that, broadly, results remain the same regardless of parameter choice, demonstrating the relevance of robust, iterative analyses and data validation [[27], [28]].

To facilitate effective comprehension and a self-led learning environment, tutorials are interspersed with question boxes and collapsible solutions, allowing users to test their understanding of the material while they learn.

MIGHTS additionally pilots multiple import strategies–ensuring reliability for live training events. This includes direct import from Zenodo [[80]], import tools linked to data atlases [[81]], and import from "input" and "answer-key" Galaxy histories–which led to the development of a new feature within

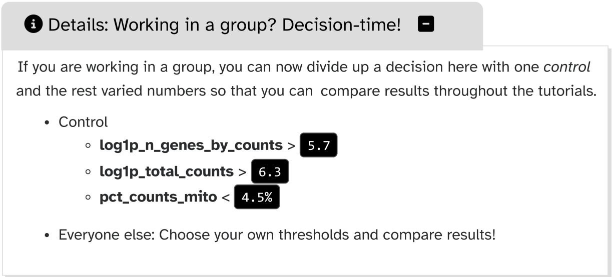

Figure 10. Exemplary 'Decision-Time' feature box in tutorial 'Filter, plot and explore single-cell RNA-seq data (Scanpy)".

the GTN to signpost the option as supporting material (Figure 11). "Answer-key" histories show datasets along every step of

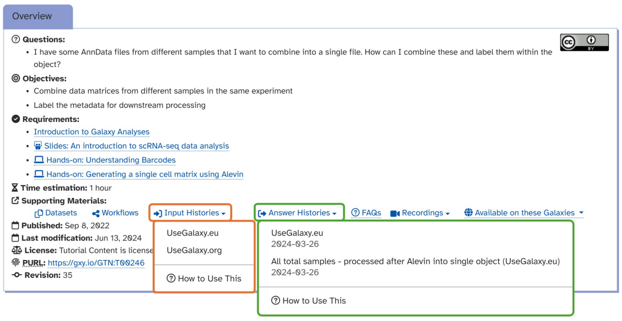

Figure 11. An overview box found at the beginning of the BB tutorial 'Combining single cell datasets after pre-processing'. It showcases a header feature which allows for a quick access to the input histories (orange frame) and answer histories (green frame).

the analyses, providing a final contingency for delivering live training and protecting users from frustration. Tutorials are additionally accompanied by slide decks (which can act as a general introduction to the topic), as well as recordings of the step-by-step analysis performed by an instructor.

Learn to Code in a Beginner Friendly Way

To ensure full understanding, each tutorial provides detailed explanations of biological and computational concepts including simplified troubleshooting and multiple interactive elements. By showing alternative methods to perform a single analysis, users become familiar with the most common programming languages in the life sciences: Python and R, as well as command language Bash. PE users additionally begin to learn the syntax and use of R [[82]]–providing them with well rounded examples of how to analyze scRNA-seq data, or how they may begin to leverage it (and Galaxy) as a means to learn new programming skills [[83]]. These PE tutorials introduce users to relevant packages, functions, and data types used in today's published bioinformatic analyses (Table 2).

The transition from Galaxy-button tutorials into the coded environment is facilitated by interactive tools such as RStudio or Jupyter Notebook, such that all the analysis can be done within Galaxy as opposed to on local instances. Importantly, there is no need for any software installation–all tutorials provide everything needed to complete them, including example datasets, slides, videos, workflows, and public Galaxy servers where the analysis can be performed. Internet access is the only additional necessary resource [[84]]. This approach specifically facilitates accessible bioinformatics analyses by eliminating installation issues, reducing the time needed to set one's environment, and increasing the computing capacity for novice users.

FAIR Data Usage

MIGHTS tutorials were created on an interface with employed findable, accessible, interoperable, and reusable (FAIR) data usage [[85]]. The FAIR principles can and should be applied in all life science domains where large amounts of data are produced. FAIR data management is particularly important in scRNA-seq analysis which looks at large expression matrices. Unfortunately, it is often the case that published datasets come with missing, or incomplete, metadata–rendering the dataset less useful than it would be with complete annotation(s). However, by completing MIGHTS tutorials, the users become equipped with the skills helpful in formatting those demanding datasets.

Sustainability

An important feature characterizing MIGHTS is its sustainability. As previously reported, the evolving nature of bioinformatics requires a sustainable bridge between the fields of biology and informatics [[86]]. Therefore, collaboration between developers and domain experts is crucial. The GTN emphasizes that users be included in this collaboration: whereby users have the opportunity to report issues and request additional resources. This facilitates involvement of developers who are aware of user needs. All issues may be reported back to tutorial developers: demonstrating the sustainability of Galaxy's Circle of Life (Figure 12). Tutorials on the GTN

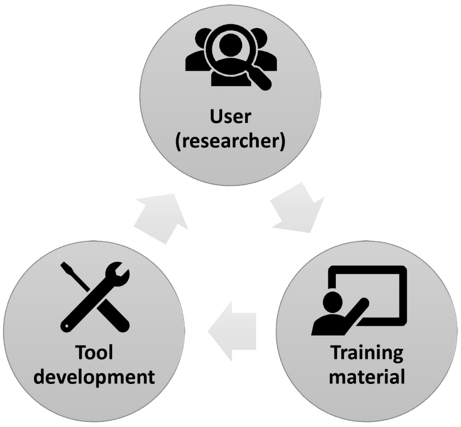

Figure 12. Galaxy Circle of Life demonstrating the interdisciplinary, multi-level sustainability practiced by the GTN. Users report changes they wish to see made in the training material, prompting new tool development and updates which can be sustainably utilized and tested by researchers.

are, at minimum, updated annually in advance of the Galaxy Community Conference (GCC). The Galaxy Circle of Life functions such that tutorials will continue to meet evolving user needs–largely thanks to the commitment of the growing Galaxy Single-Cell & Spatial Omics Community. Notably, MIGHTS tutorials have been updated, on average, 7 times a year since their respective publication (Table 3). The number of revisions demonstrates continued sustainability of tutorials featured in the suite.

Addressing Modern Challenged in Bioinformatics

MIGHTS addresses many broad challenges of bioinformatics training, emphasizing that effective bioinformatics involves understanding key principles and gaining experience [[22]] with real world data, problem-solving [[24]], reproducibility [[87]], and validation [[88]].

One challenge in bioinformatics is the application of analyses from training courses to real, messy, lab-generated data. Uniquely, MIGHTS uses raw, un-annotated data from a published analysis:

Table 3. Number of revisions made to each tutorial featured in MIGHTS as of August 2024.

Tutorial Topic	BB tutorials		PE tutorials	
	Months since tutorial Publication	Number of Revisions	Months since Publication	Number of Revisions
Generating a single-cell matrix using Alevin	41	16	8	2
Combining single-cell datasets after pre-processing	23	16		
Filter plot explore single-cell rna seq data with Scanpy	40	18	11	9
Filter Plot and explore single-cell RNA-seq data with Seurat	4	3	10	8
Inferring single-cell trajectories with Scanpy	8	5	40	15
Inferring single-cell trajectories with Monocle3	22	19	15	10

Bacon *et al.* 2018 [[72]], and guides users through reformatting and annotating.

Reproducibility, a keystone of quality bioinformatic analyses, is ensured by MIGHTS thanks to the inclusion of published workflows and key histories for each dataset (Figure 11). Workflows are available on the Galaxy servers, providing a stable way to perform a particular analysis in an identical environment. By linking the tool versions to the tutorials themselves, it is possible to submit new input files, adjust parameter thresholds, and wait for an output. This is particularly helpful for analyzing multiple samples that require the same pipeline, allowing reproducible results, minimizing time spent running code, and eliminating the need for complex coding skills.

To address another challenge of the field, this MIGHTS emphasizes the importance of validating one's results: to determine whether results reflect an actual biological process or artifacts of the pipeline. Using tools based in various programming languages and using different algorithms, allows users to feel confident that their results are uncovering true biological insights no matter the analysis method used (Figure 9). MIGHTS can act as a guide on how to validate results. The suite may additionally be used directly by experienced users to check whether their results are consistent via an alternative method without learning another programming language.

Because bioinformatics combines numerous STEM fields, it faces interdisciplinary and inter-generational challenges [[89]]. Software developers often do not understand the underlying biology and biologists do not know how analytical algorithms work [[90]]. MIGHTS aims to fill this gap by introducing step-by-step analyses, while simultaneously demonstrating the biological interpretation of results and how they were uncovered. Coded tutorials give additional opportunities to become familiar with the algorithms behind analyses. The suite can act as a resource to educate and inspire future generations of bioinformaticians: ones who are able to speak across disciplines, effectively identifying areas for improvement, and building flexible, long term solutions.

Limitations and Further Steps

The main limitation of the Galaxy GUI tutorials is that the analysis is limited to the packages and functions that have been wrapped into tools. As such, some analysis steps might be limited in the BB tutorials. However, users have the opportunity to submit 'tool requests': an ongoing effort to mitigate this limitation.

Additionally, tool versions must be compatible with one another. To mitigate this limitation, tools are regularly tested and updated to allow for analysis using the most recent versions. Problems with tools can be reported on Galaxy forums, where experts and developers respond quickly to issues.

The main limitations of the PE tutorials are limited resources allocated to Interactive Environments, and inconsistencies between the notebooks on different public Galaxy servers (.eu vs .org vs .au). However, the educational purpose of the coded-tutorials is to familiarize users with coding environments, so downsampled data provides the same benefits and enables most analyses to be done within the resource limit. Even so, should a user need or want more resources allocated, they can request that from the Galaxy admins.

There are ongoing efforts to expand the functionality of MIGHTS to enable more bespoke analyses of datasets, in response to community needs.

Availability of Source Code and Requirements

- Project name: Multi-Interface Galaxy Hands-on Training Suite for scRNA-seq
- Project home page: https://github.com/galaxyproject/training-material/tree/main/topics/single-cell/tutorials
- Operating system(s): web-based, platform independent
- Programming languages: R, Python, Bash
- License: MIT

Availability of Supporting Data and Materials

All the tutorials are available at dedicated Single Cell subpage of Galaxy Training Network (GTN) at https://training.galaxyproject.org/training-material/topics/single-cell.

The used experimental data comes from a published study by Bacon *et al.* 2018 [41], that is publicly available from the EMBL-EBI ArrayExpress under accession number E-MTAB-6945 and can also be browsed from Single Cell Expression Atlas. The input datasets used in tutorials are stored at Zenodo and all generated data files are available in the shared Galaxy histories, included in each tutorial.

The tutorials comprise many different tools that can be freely used at the Galaxy public servers (https://usegalaxy.eu, https://usegalaxy.org and others). The tool wrappers with detailed information are stored at the Galaxy ToolShed (https://toolshed.g2.bx.psu.edu).

Declarations

Abbreviations

BB: button-based CDS: Cell Data Set DPT: Diffusion Pseudotime EMBL-EBI: European Molecular Biology Laboratory - European Bioinformatics Institute FAIR: Findability, Accessibility, Interoperability, Reusability FDG: Force-directed Graph GCC: Galaxy Community Conference GTF: Gene Transfer Format GTN: Galaxy Training Network GUI: Graphical User Interface LSI: Latent Semantic Indexing MIGHTS: Multi-Interface Galaxy Hands-on Training Suite for scRNA-seq PAGA: Partition-Based Graph Abstraction PC: Principal Component PCA: Principal Component Analysis PE: programming environment QC: Quality Control SCE: SingleCellExperiment scRNA-seq: single cell RNA sequencing SE: SummarizedExperiment STEM: Science, Technology, Engineering and Mathematics TI: Trajectory Inference tSNE: t-distributed Stochastic Neighbor Embedding UMAP: Uniform Manifold Approximation and Projection

Ethics Approval and consent to participate

Not applicable.

Consent for Publication

Not applicable.

Competing Interests

The author(s) declare that they have no competing interests.

Funding

Internships were funded in part by the Engineering & Physical Sciences Research Council (UK) as well as Hobart and William Smith Colleges (Geneva, NY, USA). Additionally, part of the materials were created thanks to the Third Training Open Call issued by EOSC-Life which has received funding from the European Union's Horizon 2020 programm under grant agreement number 824087.

Authors' Contributions

Conceptualization: Wendi Bacon; **Data curation**: Wendi Bacon, Helena Rasche; **Formal analysis**: Wendi Bacon, Camila Goclowski, Morgan Howells, Julia Jakiela, Marisa Loach, Jonathan Manning; **Funding acquisition**: Wendi Bacon; **Investigation**: Wendi Bacon, Camila Goclowski, Morgan Howells, Julia Jakiela, Marisa Loach, Jonathan Manning; **Methodology**: Wendi Bacon, Camila Goclowski, Morgan Howells, Julia Jakiela, Marisa Loach, Jonathan Manning; **Project administration**: Wendi Bacon; **Software**: Tyler Collins, Saskia Hiltemann, Pablo Moreno, Alex Ostrovsky, Helena Rasche, Mehmet Tekman, Pavankumar Videm; **Resources**: Tyler Collins, Saskia Hiltemann, Alex Ostrovsky, Helena Rasche, Mehmet Tekman, Pavankumar Videm, Wendi Bacon; **Supervision**: Wendi Bacon; **Validation**: Wendi Bacon, Marisa Loach, Morgan Howells, Julia Jakiela, Camila Goclowski, Jonathan Manning, Mehmet Tekman, Graeme Tyson, Pavankumar Videm; **Visualization** (tutorials): Wendi Bacon, Camila Goclowski, Morgan Howells, Julia Jakiela, Marisa Loach, Jonathan Manning; **Visualization** (paper): Julia Jakiela; **Writing-original draft**: Camila Goclowski, Julia Jakiela; **Writing-review & editing**: Wendi Bacon, Camila Goclowski, Julia Jakiela, Marisa Loach, Pavankumar Videm *Both first authors may state their name first on CV.*

Acknowledgments

The authors extend their gratitude to the Galaxy community for supporting the testing of workflows as well as the development of tools. We thank Gareth Price for support testing on the Australian Galaxy instance and training course participants for testing tutorials in live user settings.

References

1. Attwood TK, Blackford S, Brazas MD, Davies A, Schneider MV. A global perspective on evolving bioinformatics and data science training needs. Brief Bioinform 2019 Mar;20(2):398–404.
2. Goodman N. Biological data becomes computer literate: new advances in bioinformatics. Curr Opin Biotechnol 2002 Feb;13(1):68–71.
3. Mitra D, Mitra D, Sabri Bensaad M, Sinha S, Pant K, Pant M, et al. Evolution of bioinformatics and its impact on modern bio-science in the twenty-first century: Special attention to pharmacology, plant science and drug discovery. Comput Toxicol 2022 Nov;24(100248):100248.
4. Singh S, Pandey AK, Prajapati VK. From genome to clinic: The power of translational bioinformatics in improving human health. Adv Protein Chem Struct Biol 2024 Feb;139:1–25.
5. Dhiman K, Dhiman H. Unveiling the world of bioinformatics. In: Advances in Bioinformatics and Biomedical Engineering IGI Global; 2024.p. 181–200.
6. Wright AM, Schwartz RS, Oaks JR, Newman CE, Flanagan SP. The why, when, and how of computing in biology classrooms. F1000Res 2019 Nov;8:1854.
7. Dabholkar S, Computational Thinking in Biology: Part 2; 2021. `https://bridges.wp.txstate.edu/2021/07/09/computational-thinking-in-biology-part-2/`.
8. Ras V, Carvajal-López P, Gopalasingam P, Matimba A, Chauke PA, Mulder N, et al. Challenges and considerations for delivering bioinformatics training in LMICs: Perspectives from pan-African and Latin American bioinformatics networks. Front Educ 2021 Jul;6.
9. Chasapi A, Promponas VJ, Ouzounis CA. The bioinformatics wealth of nations. Bioinformatics 2020 May;36(9):2963–2965.
10. Erxleben-Eggenhofer A, Batut B, Müller T. FAIR and scalable education. Proc Conf Res Data Infrastr 2023 Sep;1.
11. Forero DA, Wonkam A, Wang W, Laissue P, López-Correa C, Fernández-López JC, et al. Current needs for human and medical genomics research infrastructure in low and middle income countries: Table 1. J Med Genet 2016 Jul;53(7):438–440.
12. Mendy M, Caboux E, Sylla BS, Dillner J, Chinquee J, Wild C, et al. Infrastructure and facilities for human biobanking in low- and middle-income countries: a situation analysis. Pathobiology 2014;81(5-6):252–260.
13. Pérez-Wohlfeil E, Torreno O, Bellis LJ, Fernandes PL, Leskosek B, Trelles O. Training bioinformaticians in High Performance Computing. Heliyon 2018 Dec;4(12):e01057.
14. Wilson Sayres MA, Hauser C, Sierk M, Robic S, Rosenwald AG, Smith TM, et al. Bioinformatics core competencies for undergraduate life sciences education. PLoS One 2018 Jun;13(6):e0196878.
15. Williams JJ, Drew JC, Galindo-Gonzalez S, Robic S, Dinsdale E, Morgan WR, et al. Barriers to integration of bioinformatics into undergraduate life sciences education: A national study of US life sciences faculty uncover significant barriers to integrating bioinformatics into undergraduate instruction. PLoS One 2019 Nov;14(11):e0224288.
16. Katara P. Role of bioinformatics and pharmacogenomics in drug discovery and development process. Netw Model Anal Health Inform Bioinform 2013 Dec;2(4):225–230.
17. Levine AG. An explosion of bioinformatics careers. Science 2014 Jun;344(6189):1303–1306.
18. Hwang B, Lee JH, Bang D. Single-cell RNA sequencing technologies and bioinformatics pipelines. Exp Mol Med 2018 Aug;50(8):1–14.
19. Navlakha S, Bar-Joseph Z. Algorithms in nature: the convergence of systems biology and computational thinking. Mol Syst Biol 2011 Nov;7(1):546.
20. Carey MA, Papin JA. Ten simple rules for biologists learning to program. PLoS Comput Biol 2018 Jan;14(1):e1005871.
21. Via A, Blicher T, Bongcam-Rudloff E, Brazas MD, Brooksbank C, Budd A, et al. Best practices in bioinformatics training for life scientists. Brief Bioinform 2013 Sep;14(5):528–537.
22. Dudley JT, Butte AJ. A quick guide for developing effective bioinformatics programming skills. PLoS Comput Biol 2009 Dec;5(12):e1000589.
23. Jazayeri M. Combining mastery learning with project-based learning in a first programming course: An experience report. In: 2015 IEEE/ACM 37th IEEE International Conference on Software Engineering IEEE; 2015. .
24. Perkins DN, Hancock C, Hobbs R, Martin F, Simmons R. Conditions of learning in novice programmers. J Educ Comput Res 1986 Feb;2(1):37–55.
25. Shafto SA. Programming for learning in mathematics and science. SIGCSE Bull 1986 Feb;18(1):296–302.
26. Johnston IG, Slater M, Cazier JB. Interdisciplinary and transferable concepts in bioinformatics education: Observations and approaches from a UK MSc course. Front Educ 2022 Feb;7.
27. Hiltemann S, Rasche H, Gladman S, Hotz HR, Larivière D, Blankenberg D, et al. Galaxy Training: A powerful framework for teaching! PLoS Comput Biol 2023 Jan;19(1):e1010752.
28. Rasche H, Hyde C, Davis J, Gladman S, Coraor N, Bretaudeau A, et al. Training Infrastructure as a Service. Gigascience 2022 Dec;12.
29. Bacon W, Holinski A, Pujol M, Wilmott M, Morgan SL, European Molecular Biology Laboratory-European Bioinformatics Institute Training Team. Ten simple rules for leveraging virtual interaction to build higher-level learning into bioinformat-

ics short courses. PLoS Comput Biol 2022 Jul;18(7):e1010220.
30. Moreno P, Huang N, Manning JR, Mohammed S, Solovyev A, Polanski K, et al. User-friendly, scalable tools and workflows for single-cell RNA-seq analysis. Nat Methods 2021 Apr;18(4):327–328.
31. Bacon W, Manning J, Generating a single cell matrix using Alevin (Galaxy Training Materials);. https://training.galaxyproject.org/training-material/topics/single-cell/tutorials/alevin-commandline/tutorial.html, [Online; accessed Sun Aug 11 2024].
32. Jakiela J, Bacon W, Generating a single cell matrix using Alevin and combining datasets (bash + R) (Galaxy Training Materials);. https://training.galaxyproject.org/training-material/topics/single-cell/tutorials/alevin-commandline/tutorial.html, [Online; accessed Sun Aug 11 2024].
33. Bacon W, Manning J, Combining single cell datasets after pre-processing (Galaxy Training Materials);. https://training.galaxyproject.org/training-material/topics/single-cell/tutorials/alevin-commandline/tutorial.html, [Online; accessed Sun Aug 11 2024].
34. Bacon W, Filter, plot and explore single-cell RNA-seq data with Scanpy (Galaxy Training Materials);. https://training.galaxyproject.org/training-material/topics/single-cell/tutorials/scrna-case_basic-pipeline/tutorial.html, [Online; accessed Sun Aug 11 2024].
35. Howells M, Bacon W, Filter, plot and explore single-cell RNA-seq data with Scanpy (Python) (Galaxy Training Materials);. https://training.galaxyproject.org/training-material/topics/single-cell/tutorials/scrna-case-jupyter_basic-pipeline/tutorial.html, [Online; accessed Sun Aug 11 2024].
36. Goclowski C, Moreno P, Filter, plot, and explore single cell RNA-seq data with Seurat (Galaxy Training Materials);. https://training.galaxyproject.org/training-material/topics/single-cell/tutorials/scrna-case_FilterPlotandExplore_SeuratTools/tutorial.html, [Online; accessed Sun Aug 11 2024].
37. Goclowski C, Filter, plot, and explore single cell RNA-seq data with Seurat (R) (Galaxy Training Materials);. https://training.galaxyproject.org/training-material/topics/single-cell/tutorials/scrna-case_FilterPlotandExploreRStudio/tutorial.html, [Online; accessed Sun Aug 11 2024].
38. Loach M, Bacon W, Jakiela J, Tekman M, Inferring single cell trajectories with Scanpy (Galaxy Training Materials);. https://training.galaxyproject.org/training-material/topics/single-cell/tutorials/scrna-case_trajectories/tutorial.html, [Online; accessed Sun Aug 11 2024].
39. Bacon W, Jakiela J, Tekman M, Inferring single cell trajectories with Scanpy (Python) (Galaxy Training Materials);. https://training.galaxyproject.org/training-material/topics/single-cell/tutorials/scrna-case_JUPYTER-trajectories/tutorial.html, [Online; accessed Sun Aug 11 2024].
40. Jakiela J, Inferring single cell trajectories with Monocle3 (Galaxy Training Materials);. https://training.galaxyproject.org/training-material/topics/single-cell/tutorials/scrna-case_monocle3-trajectories/tutorial.html, [Online; accessed Sun Aug 11 2024].
41. Jakiela J, Inferring single cell trajectories with Monocle3 (R) (Galaxy Training Materials);. https://training.galaxyproject.org/training-material/topics/single-cell/tutorials/scrna-case_monocle3-rstudio/tutorial.html, [Online; accessed Sun Aug 11 2024].
42. Grüning BA, Rasche E, Rebolledo-Jaramillo B, Eberhard C, Houwaart T, Chilton J, et al. Jupyter and Galaxy: Easing entry barriers into complex data analyses for biomedical researchers. PLoS Comput Biol 2017 May;13(5):e1005425.
43. Baumer B, Udwin D. R Markdown. WIREs Computational Statistics 2015;7(3):167–177.
44. Kluyver T, Ragan-Kelley B, Pérez F, Granger B, Bussonnier M, Frederic J, et al. Jupyter Notebooks – a publishing format for reproducible computational workflows. In: Loizides F, Schmidt B, editors. Positioning and Power in Academic Publishing: Players, Agents and Agendas IOS Press; 2016. p. 87–90.
45. Satija R, Farrell JA, Gennert D, Schier AF, Regev A. Spatial reconstruction of single-cell gene expression data. Nat Biotechnol 2015 May;33(5):495–502.
46. Hao Y, Stuart T, Kowalski MH, Choudhary S, Hoffman P, Hartman A, et al. Dictionary learning for integrative, multimodal and scalable single-cell analysis. Nat Biotechnol 2024 Feb;42(2):293–304.
47. Hao Y, Hao S, Andersen-Nissen E, Mauck WM 3rd, Zheng S, Butler A, et al. Integrated analysis of multimodal single-cell data. Cell 2021 Jun;184(13):3573–3587.e29.
48. Stuart T, Butler A, Hoffman P, Hafemeister C, Papalexi E, Mauck WM 3rd, et al. Comprehensive integration of single-cell data. Cell 2019 Jun;177(7):1888–1902.e21.
49. Butler A, Hoffman P, Smibert P, Papalexi E, Satija R. Integrating single-cell transcriptomic data across different conditions, technologies, and species. Nat Biotechnol 2018 May;36(5):411–420.
50. Cao J, Spielmann M, Qiu X, Huang X, Ibrahim DM, Hill AJ, et al. The single-cell transcriptional landscape of mammalian organogenesis. Nature 2019 Feb;566(7745):496–502.
51. Wolf FA, Angerer P, Theis FJ. SCANPY: large-scale single-cell gene expression data analysis. Genome Biol 2018 Dec;19(1).
52. Patro R, Duggal G, Love MI, Irizarry RA, Kingsford C. Salmon provides fast and bias-aware quantification of transcript expression. Nat Methods 2017 Apr;14(4):417–419.
53. Srivastava A, Malik L, Smith T, Sudbery I, Patro R. Alevin efficiently estimates accurate gene abundances from dscRNA-seq data. Genome Biol 2019 Mar;20(1):65.
54. Lun ATL, Riesenfeld S, Andrews T, Dao TP, Gomes T, participants in the 1st Human Cell Atlas Jamboree, et al. EmptyDrops: distinguishing cells from empty droplets in droplet-based single-cell RNA sequencing data. Genome Biol 2019 Mar;20(1):63.
55. Griffiths JA, Richard AC, Bach K, Lun ATL, Marioni JC. Detection and removal of barcode swapping in single-cell RNA-seq data. Nat Commun 2018 Jul;9(1):2667.
56. Moreno P, Fexova S, George N, Manning JR, Miao Z, Mohammed S, et al. Expression Atlas update: gene and protein expression in multiple species. Nucleic Acids Res 2022 Jan;50(D1):D129–D140.
57. Love MI, Soneson C, Hickey PF, Johnson LK, Pierce NT, Shepherd L, et al. Tximeta: Reference sequence checksums for provenance identification in RNA-seq. PLoS Comput Biol 2020 Feb;16(2):e1007664.
58. Durinck S, Moreau Y, Kasprzyk A, Davis S, De Moor B, Brazma A, et al. BioMart and Bioconductor: a powerful link between biological databases and microarray data analysis. Bioinformatics 2005 Aug;21(16):3439–3440.
59. Durinck S, Spellman PT, Birney E, Huber W. Mapping identifiers for the integration of genomic datasets with the R/Bioconductor package biomaRt. Nat Protoc 2009 Jul;4(8):1184–1191.
60. Csardi G, Nepusz T. The igraph software package for complex network research. InterJournal 2006;Complex Systems:1695. https://igraph.org.
61. Blondel VD, Guillaume JL, Lambiotte R, Lefebvre E. Fast unfolding of communities in large networks. J Stat Mech 2008 Oct;2008(10):P10008.
62. McKinney W. Data Structures for Statistical Computing in

Python. In: Proceedings of the 9th Python in Science Conference SciPy; 2010. .
63. Bates D, Maechler M, Jagan M, Matrix: Sparse and dense matrix classes and methods. The R Foundation; 2000. Title of the publication associated with this dataset: CRAN: Contributed Packages.
64. Wickham H, François R, Henry L, Müller K, Vaughan D. dplyr: A Grammar of Data Manipulation; 2023, https://dplyr.tidyverse.org, r package version 1.1.4, https://github.com/tidyverse/dplyr.
65. Razavi K, Luthra M, Koldehofe B, Muhlhauser M, Wang L. FA2: Fast, accurate autoscaling for serving deep learning inference with SLA guarantees. In: 2022 IEEE 28th Real-Time and Embedded Technology and Applications Symposium (RTAS) IEEE; 2022. .
66. Harris CR, Millman KJ, van der Walt SJ, Gommers R, Virtanen P, Cournapeau D, et al. Array programming with NumPy. Nature 2020 Sep;585(7825):357–362.
67. Hunter JD. Matplotlib: A 2D graphics environment. Computing in Science & Engineering 2007;9(3):90–95.
68. Virshup I, Rybakov S, Theis FJ, Angerer P, Wolf FA. anndata: Annotated data; 2021.
69. Garnier S, Ross N, Camargo AP, Rudis B, Woo K, sjmgarnier/viridisLite: viridisLite 0.4.0 (pre-CRAN release). Zenodo; 2021.
70. Bache SM, Wickham H. magrittr: A Forward-Pipe Operator for R; 2022, https://magrittr.tidyverse.org, https://github.com/tidyverse/magrittr.
71. Eddelbuettel D, Francois R, Allaire J, Ushey K, Kou Q, Russell N, et al. Rcpp: Seamless R and C++ Integration; 2024, https://CRAN.R-project.org/package=Rcpp, r package version 1.0.13.
72. Bacon WA, Hamilton RS, Yu Z, Kieckbusch J, Hawkes D, Krzak AM, et al. Single-cell analysis identifies thymic maturation delay in growth-restricted neonatal mice. Front Immunol 2018 Nov;9:2523.
73. He J, Lin L, Chen J. Practical bioinformatics pipelines for single-cell RNA-seq data analysis. Biophys Rep 2022 Jun;8(3):158–169.
74. van der Maaten L, Hinton G. Visualizing Data using t-SNE. Journal of Machine Learning Research 2008;9:2579–2605. http://www.jmlr.org/papers/v9/vandermaaten08a.html.
75. McInnes L, Healy J, Saul N, Großberger L. UMAP: Uniform Manifold Approximation and Projection. J Open Source Softw 2018 Sep;3(29):861.
76. Megill C, Martin B, Weaver C, Bell S, Prins L, Badajoz S, et al. Cellxgene: A performant, scalable exploration platform for high dimensional sparse matrices; 2021.
77. Hafemeister C, Satija R. Normalization and variance stabilization of single-cell RNA-seq data using regularized negative binomial regression. Genome Biol 2019 Dec;20(1):296.
78. Tractenberg RE, Lindvall JM, Attwood TK, Via A. The Mastery Rubric for Bioinformatics: A tool to support design and evaluation of career-spanning education and training. PLoS One 2019 Nov;14(11):e0225256.
79. Ko ME, Williams CM, Fread KI, Goggin SM, Rustagi RS, Fragiadakis GK, et al. FLOW-MAP: a graph-based, force-directed layout algorithm for trajectory mapping in single-cell time course datasets. Nat Protoc 2020 Feb;15(2):398–420.
80. Cwts:cwts NA, corporate-body RTD:Directorate-General for Research, Innovation, Elsevier:Elsevier NA, Esade:esade NA, null agent Lisbon Council:Lisbon Council. Zenodo: Open science monitor case study. Publications Office of the European Union; 2019.
81. Papatheodorou I, Moreno P, Manning J, Fuentes AMP, George N, Fexova S, et al. Expression Atlas update: from tissues to single cells. Nucleic Acids Res 2020 Jan;48(D1):D77–D83.
82. R Core Team. R: A Language and Environment for Statistical Computing. R Foundation for Statistical Computing, Vienna, Austria; 2021, https://www.R-project.org/.
83. Goclowski CL, From GTN Intern to Tutorial Author to Bioinformatician; 2024. https://gxy.io/GTN:N00087.
84. Goecks J, Nekrutenko A, Taylor J, Galaxy Team. Galaxy: a comprehensive approach for supporting accessible, reproducible, and transparent computational research in the life sciences. Genome Biol 2010 Aug;11(8):R86.
85. Ouwerkerk J, Rasche H, Spalding JD, Hiltemann S, Stubbs AP. FAIR data retrieval for sensitive clinical research data in Galaxy. Gigascience 2024 Jan;13.
86. Aron S, Chauke PA, Ras V, Panji S, Johnston K, Mulder N. The Development of a Sustainable Bioinformatics Training Environment Within the H3Africa Bioinformatics Network (H3ABioNet). Frontiers in Education 2021;6. https://www.frontiersin.org/journals/education/articles/10.3389/feduc.2021.725702.
87. Cokelaer T, Cohen-Boulakia S, Lemoine F. Reprohackathons: promoting reproducibility in bioinformatics through training. Bioinformatics 2023 Jun;39(Supplement$_1$) : $i11$–$i20$.
88. Yang A, Troup M, Ho JWK. Scalability and validation of big data bioinformatics software. Comput Struct Biotechnol J 2017 Jul;15:379–386.
89. Bartlett A, Lewis J, Williams ML. Generations of interdisciplinarity in bioinformatics. New Genet Soc 2016 Apr;35(2):186–209.
90. Garmire DG, Zhu X, Mantravadi A, Huang Q, Yunits B, Liu Y, et al. GranatumX: A community-engaging, modularized, and flexible webtool for single-cell data analysis. Genomics Proteomics Bioinformatics 2021 Jun;19(3):452–460.
