# Supplementary material for: Galaxy as a gateway to bioinformatics: Multi-Interface Galaxy Hands-on Training Suite (MIGHTS) for scRNA-seq
Source: Gigascience. 2025 Jan 8;14:giae107. doi: 10.1093/gigascience/giae107 (PMC11707610; doi:10.1093/gigascience/giae107)
Supplement: giae107_GIGA-D-24-00327_Revision_1 [file giae107_giga-d-24-00327_revision_1.pdf]

GigaScience

Galaxy as a Gateway to Bioinformatics: Multi-Interface Galaxy Hands-on Training Suite (MIGHTS) for scRNA-seq

--Manuscript Draft--

**Manuscript Number:**	GIGA-D-24-00327R1
**Full Title:**	Galaxy as a Gateway to Bioinformatics: Multi-Interface Galaxy Hands-on Training Suite (MIGHTS) for scRNA-seq
**Article Type:**	Technical Note
**Funding Information:**	Horizon 2020 (824087) — Ms. Julia Jakiela<br>Hobart and William Smith Colleges — Ms. Camila Goclowski<br>Engineering and Physical Sciences Research Council — Mr. Morgan Howells
**Abstract:**	Background. Bioinformatics is fundamental to biomedical sciences, but its mastery presents a steep learning curve for bench biologists and clinicians. Learning to code while analyzing data is difficult. The curve may be flattened by separating the two aspects and providing intermediate steps for budding bioinformaticians. Single-cell analysis is in great demand from biologists and biomedical scientists, as evidenced by the proliferation of training events, materials, and collaborative global efforts like the Human Cell Atlas. However, iterative analyses and un-standardized pipelines have made effective single-cell training a moving target. Findings. To address these challenges, we present a Multi-Interface Galaxy Hands-on Training Suite (MIGHTS) for scRNA-seq analysis, which offers parallel analytical methods using a graphical interface (buttons) or code. With clear, interoperable materials, MIGHTS facilitates smooth transitions between environments. Bridging the biologist-programmer gap, MIGHTS emphasizes interdisciplinary communication for effective learning at all levels. Real-world data analysis in MIGHTS promotes critical thinking and best practices, while FAIR data principles ensure validation of results. MIGHTS is freely available, hosted on the Galaxy Training Network, and leverages Galaxy interfaces for analyses in both settings. Given the ongoing popularity of Python-based (Scanpy) and R-based (Seurat, Monocle) scRNA-seq analyses, MIGHTS enables analyses using both. Conclusions. MIGHTS consists of 11 tutorials including recordings, slide-decks, and interactive visualizations, with a proven track record of sustainability via regular updates and community collaborations. Parallel pathways in MIGHTS enable concurrent training of scientists at any programming level, addressing the heterogeneous needs of novice bioinformaticians.
**Corresponding Author:**	Wendi Bacon, PhD<br>The Open University<br>Milton Keynes, UNITED KINGDOM
**Corresponding Author Secondary Information:**	
**Corresponding Author's Institution:**	The Open University
**Corresponding Author's Secondary Institution:**	
**First Author:**	Camila Goclowski
**First Author Secondary Information:**	
**Order of Authors:**	Camila Goclowski<br>Julia Jakiela<br>Tyler Collins<br>Saskia Hiltemann<br>Morgan Howells

Powered by Editorial Manager® and ProduXion Manager® from Aries Systems Corporation

	Marisa Loach
	Jonathan Manning
	Pablo Moreno
	Alex Ostrovsky
	Helena Rasche
	Mehmet Tekman
	Graeme Tyson
	Pavankumar Videm
	Wendi Bacon
**Order of Authors Secondary Information:**	
**Response to Reviewers:**	Reviewer 1:<br><br>1.“While the manuscript lays emphasis on unstandardized and iterative analysis, the authors could draw a strong rationale on lack of reinstantiation methods which is certainly the need. The tools like Scanpy/Seurat and other ecosystem tools, there is a latency and they work differently.”<br><br>●In the discussion, reinstantiation has been emphasized, in connection to reproducibility, as an end goal accomplishment of the MIGHTS tutorial suite.<br><br>2.“Training-the-trainer is a very important entity as next generation sequencing (NGS) tools advances. A word or two on that would be a nice addition.”<br><br>●In the discussion, added “As sequencing strategies and tools advance, it is important that the field of bioinformatics “trains the trainer” in response to continued growth”.<br><br>3.“While programming based and button based entities are well taken, there could be a ‘programming a button’ section if not swapping between them. This will allow naive bioinformaticists to embrace programming. The authors may want to mention this.”<br>○In the discussion, added a nod to the existing “How to wrap a galaxy tool” section of GTN Training Material: “If users wish to embrace programming such that they are looking to wrap their own tools and create training material based on them, resources and opportunities to do so exist on GTN pages dedicated to development in Galaxy [https://training.galaxyproject.org/training-material/topics/dev/] and contributing to the Galaxy Training Material [https://training.galaxyproject.org/training-material/topics/contributing/]”<br><br>4.“There could be an alternative annotation tool, viz. Annotationhub instead of Biosmart as well.”<br><br>●We thank the reviewers for this comment, however we cannot include every available tool in Galaxy, so unfortunately this is beyond the scope of the project. The authors agree that expanding annotation tool availability and programming environments would be beneficial. The Galaxy Training Network is committed to continued enhancement of tools to meet evolving user needs.<br><br>5.“Likewise vscode could be more inviting for some instead of jupyter IDE. The end user may be given a note in README file”<br>●Because this is training material, rather than traditional coding documentation, there is no README file associated with the training. VSCode is not traditionally used for training, as the Jupyter IDE includes both the coding environment and the space to run it. For this reason, we have not mentioned text editing software. However, after completing the MIGHTS tutorials, the users should feel comfortable enough in the programming environment to work in another IDE if they find it more inviting.<br><br>Reviewer 2:<br><br>We have addressed each of the following grammatical/spelling errors:

6.As access to computationally driven domains of biology continue[s] to grow
7.blending skills across disciplines is not without challenge[s]
8."MIGHTS demonstrates the use of many frequently used data types and packages for scRNA-seq analyses (Table 2), preparing users with research[-]relevant skills."
9."Workflows for each tutorial topic are shown -below- in Figure 4."
○"Workflow is demonstrated -below- in Figure 8" (remove the belows)

We have addressed the following figure and table corrections:
10.“Figure 1 is too small to read, and it would be interesting to compare the BB method and the PE method to get the same images.”
○Figure 1 has been edited to convey key concepts/steps in the tutorial. Relevant information from the original figure was converted to text from a screenshot so as to more uniformly present the arguments. The figure caption has also been altered to more directly compare BB and PE methods.
11.“Table 1 and 2 are redundant, use only table 2.”
○Tables 1 and 2 have been collapsed such that they do not present redundant information.
12.“Figure 4: Too small to see the stars.”.
○Figure 4 has been replaced with a more legible workflow diagram describing only the necessary details of the tutorials’ workflows.
13.“Figure 4,5,6,7: Add the significance of colored boxes in the legend. (too small to read the box titles). Overall, these figures are hard to read and are difficult to link with the text. Maybe in the text about tutorials, mention which step corresponds to which box color, or move these figures to supplemental material with more detailed legends.”
○Figures 4,5,6,7,8 have been replaced with more legible workflow diagrams rather than images of the extracted Galaxy workflows. The updated diagrams visualize the high-level information and the tools used in each step of the analysis. The coloring of the boxes has been unified, for example the input files are now all shown in orange, output files in purple, plotting in cyan, marker genes in bright yellow. Galaxy-generated workflow images were included in the Supplementary Data to provide exhaustive details, such as all the output files generated by each tool and their formats.
14.“Figure 9: what does each letter correspond to? It looks like it is showing the same information than figure 1.”
○Figure 1 is intended to draw attention to the similarities in arguments across all four modes when plotting the expression of a gene of interest. Whereas Figure 9 demonstrates the consistency of clustering results observed across all four methods. The letters in the Figure 9 were removed since the image itself is self-explanatory, without the need to include the four panels A-D.
15."’MIGHTS demonstrates the use of many frequently used data types and packages for scRNA-seq analyses (Table 2), preparing users with research relevant skills.’ : Discuss a bit more which skills are deemed "research-relevant". I agree that both the biological skills and coding skills are important, but in that sentence I am not sure why it's linked to the datatypes and packages.”
○Discussion of research relevant skills has been expanded to specify the demonstrated connection between learning to code and enhanced critical thinking: “MIGHTS demonstrates the use of many frequently used data types and packages for scRNA-seq analyses (Table 1), preparing users with research-relevant skills. Broadly applicable use of programming functions, algorithms, and troubleshooting lends itself to increased creative and critical thinking [42, 43].”

Reviewer 3:

16.“Intro: Give some information about the type of information these tutorial provide in the end: is it the growth rate for each cell type in the fetus?”
○Description of the sample dataset and biological insights explored by the suite have been introduced earlier than in the original draft–emphasizing the kind of information and analysis skills the suite provides.
17.“Overall, add a little bit more high-level information about what each tutorial does, for people who are not already familiar with scRNA-seq.”
○All figures have been altered such that they are more legible and concise. Workflow figures for the tutorials have been edited to include pertinent information regarding the steps and accomplishments of the tutorial(s). Additionally, higher-level descriptions of the tutorial outcomes are described in each of their respective text introductions.
18.“The Single cell subpage contains more than the MIGHTS material, are they all

	supported the same way by the community with the same revision rate than described in table 3?"<br>○All the material on the Single Cell subpage is maintained by the community to provide updated and well-functioning tutorials. Since the MIGHTS tutorials are designed as a suite, their average revision rate is different from standalone tutorials: "These tutorials are similarly monitored and revised, although the rate of growth specifically for single-cell tutorials is noteworthy."<br>19. "In the Discussion: Do you have advice to give to people who want to develop similar material in their field?"<br>○Broader discussion of the topics available on the Galaxy Training Network have been added such that readers may be directed to explore, and even contribute to, the growth of these resources across many applications of bioinformatics. Also, the resources to develop both training material as well as wrapping the tools have been mentioned: "If users wish to embrace programming such that they are looking to wrap their own tools and create training material based on them, resources and opportunities to do so exist on GTN pages dedicated to development in Galaxy [https://training.galaxyproject.org/training-material/topics/dev/] and contributing to the Galaxy Training Material [https://training.galaxyproject.org/training-material/topics/contributing/]"<br>20."It would be nice to have separate learning paths for BB and PE, so that users who want to focus on developing one set of skill find them more easily."<br>○Distinct learning pathways for BB and PE users have been additionally emphasized in the text and in reference to Figure 2.<br>○Two distinct learning pathways have been created - one for BB, the other for PE and referenced in the Discussion: "To facilitate choosing the right starting point depending on the users' experience or skills to develop, single-cell-oriented Learning Pathways were introduced. "Applying single-cell RNA-seq analysis" [51] and "Applying single-cell RNA-seq analysis in Coding Environments" [52] pathways are based on BB and PE tutorials respectively and can be used for a smooth transition from button-based tutorials to programming environment (Figure 2A) or a direct start in the coding environment (Figure 2B)."<br>21."It would be nice to be able to find this set of tutorials by searching MIGHTS in the GTN."<br>○We have added a tag to each tutorial to allow for this searching, and noted this in the Discussion: "Additionally, to allow for easy searching for any of the described tutorials, each tutorial is has a tag and hence the user can simply enter "MIGHTS" in the GTN search box to get a list of the relevant materials."
**Additional Information:**	
**Question**	**Response**
Are you submitting this manuscript to a special series or article collection?	No
**Experimental design and statistics**<br><br>Full details of the experimental design and statistical methods used should be given in the Methods section, as detailed in our [Minimum Standards Reporting Checklist](). Information essential to interpreting the data presented should be made available in the figure legends.<br><br>Have you included all the information requested in your manuscript?	Yes
**Resources**	Yes

Powered by Editorial Manager® and ProduXion Manager® from Aries Systems Corporation

A description of all resources used, including antibodies, cell lines, animals and software tools, with enough information to allow them to be uniquely identified, should be included in the Methods section. Authors are strongly encouraged to cite Research Resource Identifiers (RRIDs) for antibodies, model organisms and tools, where possible.<br><br>Have you included the information requested as detailed in our Minimum Standards Reporting Checklist?	
**Availability of data and materials**<br><br>All datasets and code on which the conclusions of the paper rely must be either included in your submission or deposited in publicly available repositories (where available and ethically appropriate), referencing such data using a unique identifier in the references and in the "Availability of Data and Materials" section of your manuscript.<br><br>Have you have met the above requirement as detailed in our Minimum Standards Reporting Checklist?	Yes

Powered by Editorial Manager® and ProduXion Manager® from Aries Systems Corporation

Manuscript Click here to access/download;Manuscript;MIGHTS V2 FINAL.docx

TITLE:

Galaxy as a Gateway to Bioinformatics: Multi-Interface Galaxy Hands-on Training Suite (MIGHTS) for scRNA-seq

AUTHORS:

[Co-first Author*] Camila L Goclowski; University of Utah; Salt Lake City, Utah, United States of America; cgoclowski@genetics.utah.edu

[Co-first Author*] Julia Jakiela; University of Edinburgh School of Chemistry; Edinburgh, United Kingdom; j.jakiela@sms.ed.ac.uk

Tyler Collins; John Hopkins Medical Institution; Baltimore, Maryland, United States of America; tcolli32@jhmi.edu

Saskia Hiltemann; Erasmus Medical Center; Rotterdam, Zuid-Holland, Netherlands; saskiahiltemann@gmail.com

Morgan Howells; The Open University School of Computing & Communications; Milton Keynes, Buckinghamshire, United Kingdom; morganhowells314@gmail.com

Marisa Loach; The Open University School of Life, Health & Chemical Sciences; Milton Keynes, Buckinghamshire, United Kingdom; marisa.loach@open.ac.uk

Jonathan Manning; EMBL-EBI; Hinxton, England; jmanning@ebi.ac.uk

Pablo Moreno; Astrazeneca (2022 - Present) & EMBL-EBI (pre-2022); Hinxton, England; pablo.moreno@astrazeneca.com

Alex Ostrovsky; John Hopkins University; Baltimore, Maryland, United States of America; a.ostrovsky@me.com

Helena Rasche; Erasmus Medical Center; Rotterdam, Zuid-Holland, Netherlands; helena.rasche@gmail.com

Mehmet Tekman; University of Freiburg Division of Pharmacology and Toxicology; Freiburg im Breisgau, Baden-Württemberg, Germany; mtekman89@gmail.com

Graeme Tyson; The Open University; Milton Keynes, Buckinghamshire, United Kingdom; graema.tyson@open.ac.uk

Pavankumar Videm; University of Freiburg Department of Computer Science; Freiburg im Breisgau, Baden-Württemberg, Germany; videmp@informatik.uni-freiburg.de

[Corresponding Author] Wendi Bacon; The Open University School of Life, Health & Chemical Sciences; Milton Keynes, Buckinghamshire, United Kingdom, MK7 6BJ; wendi.bacon@open.ac.uk

First and middle authors have been listed in the alphabetical order. Both first authors contributed equally to this publication and as such: should put their name first on respective CVs and receive equal credit for contributions made.

ORCID iDs:
Camila Goclowski [0009-0002-1327-0424]; Julia Jakiela [0009-0001-2017-8805]; Tyler Collins [0000-0002-3026-9049]; Saskia Hiltemann [0000-0003-3803-468X]; Morgan Howells [0009-0008-9422-6380]; Marisa Loach [0000-0001-6979-6930]; Jonathan Manning [0000-0002-3483-8456]; Pablo Moreno [0000-0002-9856-1679]; Alex Ostrovsky [0000-0002-7901-7109]; Helena Rasche [0000-0001-9760-8992]; Mehmet Tekman [0000-0002-4181-2676]; Graeme Tyson [0000-0002-1748-2806]; Pavankumar Videm [0000-0002-5192-126X]; Wendi Bacon [0000-0002-8170-8806];

ABSTRACT

Background. Bioinformatics is fundamental to biomedical sciences, but its mastery presents a steep learning curve for bench biologists and clinicians. Learning to code while analyzing data is difficult. The curve may be flattened by separating the two aspects and providing intermediate steps for budding bioinformaticians. Single-cell analysis is in great demand from biologists and biomedical scientists, as evidenced by the proliferation of training events, materials, and collaborative global efforts like the Human Cell Atlas. However, iterative analyses lacking reinstantiation coupled with unstandardized pipelines have made effective single-cell training a moving target. **Findings**. To address these challenges, we present a Multi-Interface Galaxy Hands-on Training Suite (MIGHTS) for scRNA-seq analysis, which offers parallel analytical methods using a graphical interface (buttons) or code. With clear, interoperable materials, MIGHTS facilitates smooth transitions between environments. Bridging the biologist-programmer gap, MIGHTS emphasizes interdisciplinary communication for effective learning at all levels. Real-world data analysis in MIGHTS promotes critical thinking and best practices, while FAIR data principles ensure validation of results. MIGHTS is freely available, hosted on the Galaxy Training Network, and leverages Galaxy interfaces for analyses in both settings. Given the ongoing popularity of Python-based (Scanpy) and R-based (Seurat, Monocle) scRNA-seq analyses, MIGHTS enables analyses using both. **Conclusions**. MIGHTS consists of 11 tutorials including recordings, slide-decks, and interactive visualizations, with a proven track record of sustainability via regular updates and community collaborations. Parallel pathways in MIGHTS enable concurrent training of scientists at any programming level, addressing the heterogeneous needs of novice bioinformaticians.

KEY WORDS

Training; STEM Education; Galaxy project; single-cell RNA-seq analysis; scRNA-seq; Bioinformatics; Reproducibility; Sustainability

INTRODUCTION

Although bioinformatics is critical to basic biological and applied biomedical research, there remains a shortage of scientists with bioinformatics expertise [1]. As computationally driven domains of biology continue to grow, bioinformatics play an important role in groundbreaking discoveries [2, 3, 4, 5]. Thinking computationally about biological processes has been shown to produce more accurate models [6] and enhance problem solving [7]. However, it is important to note that bioinformatics often requires many, expensive resources, such as computational infrastructure, maintenance, and training [8]. Financial barriers can limit

access to training and research [9, 10, 11, 12, 13]. As such, many bioinformaticians rarely receive formal training in the field [8] and teaching bioinformatics is notably difficult.

Integrating bioinformatics into undergraduate curriculums may address this gap [1, 14]. Bioinformatics has been introduced in high schools, where it was shown to improve awareness, engagement, and self-efficacy of students: leading to increased interest in STEM careers [15]. Pharmaceutical companies need biomedical analysts [16], most employers in the life sciences prefer some competency in software analyses [17], and the use of bioinformatic analyses to characterize novel cell types and lineages [18] has surged. In response, institutes are beginning to teach foundational computing skills to biologists [14, 19].

Materials that focus on problem-solving, interactivity, and cooperative learning have demonstrated enhanced learning outcomes [20] and bioinformatics has effectively been taught by emphasizing interdisciplinary problem-solving [21]. To standardize training, a list of "rules" were identified to teach scientists to program: beginning with the end in mind, taking small steps forward, and focusing on individual tasks [20]. The 'end in mind' requires domain-specific understanding (i.e. identifying cell types via marker genes) while the individual tasks require programming skills (R, Python, troubleshooting, etc.). This duality forces participants to learn and apply two new skill-sets simultaneously [22, 23, 24]. The need to embed computing into science is not novel [25], but blending skills across disciplines is not without challenges [26].

The Galaxy Training Network (GTN) boasts tutorials for analysis across a range of fields, all publicly available and accessible via URL [27]. The GTN provides free training infrastructure to fast-track trainees via live courses in which trainers are available to monitor and assist participants [28]. This supports all, but especially low-resource institutions', engagement with bioinformatic training and has additionally been tested for native Spanish speakers [28]. Integrating these free resources into undergraduate curriculums has been successful [27], as training materials include interactive features based on research-backed pedagogies. Separation of learning components has previously been suggested as an effective method [29], but raises the question: how can coding and complex bioinformatic analyses be isolated from one another?

Here, we directly address the need to separate the two for training. Leveraging the Galaxy Graphical User Interface (GUI) and the GTN, we present MIGHTS: a scRNA-seq tutorial suite enabling a smooth transition from data analysis in a button-based, user-friendly environment [30] to a more advanced, flexible programming environment. Using a sample dataset, MIGHTs guides users through the steps necessary to accomplish commonly published scRNA-seq analyses and visualizations: including generating a matrix, combining datasets, filtering, plotting and general exploration of the data, as well as trajectory inference of a dataset known to demonstrate a developmental spectrum. The sample dataset available for use with the suite, reveals a delay in thymic maturation in growth restricted neonatal mice [31]. MIGHTS offers multiple routes of scRNA-seq analysis: allowing a button-based or coding-based version of the same, commonly published workflows. MIGHTS offers opportunities for a heterogeneous student population ranging from programming-friendly to programming-fearful, expanding access to critical skills required for effective bioinformatic analyses, biomedical, and life science research.

METHODS

Multi-Environment

MIGHTS consists of 11 tutorials: six button-based (BB) and five in a programming environment (PE) (Table 1). The Galaxy GUI features “click-to-run” buttons which execute programming functions [30]. Users select and set parameters from dropdown lists and input boxes (Figure 1, Left Column: Button-based). Each tool includes help text to guide users and describe the flexibility of the tool’s function.

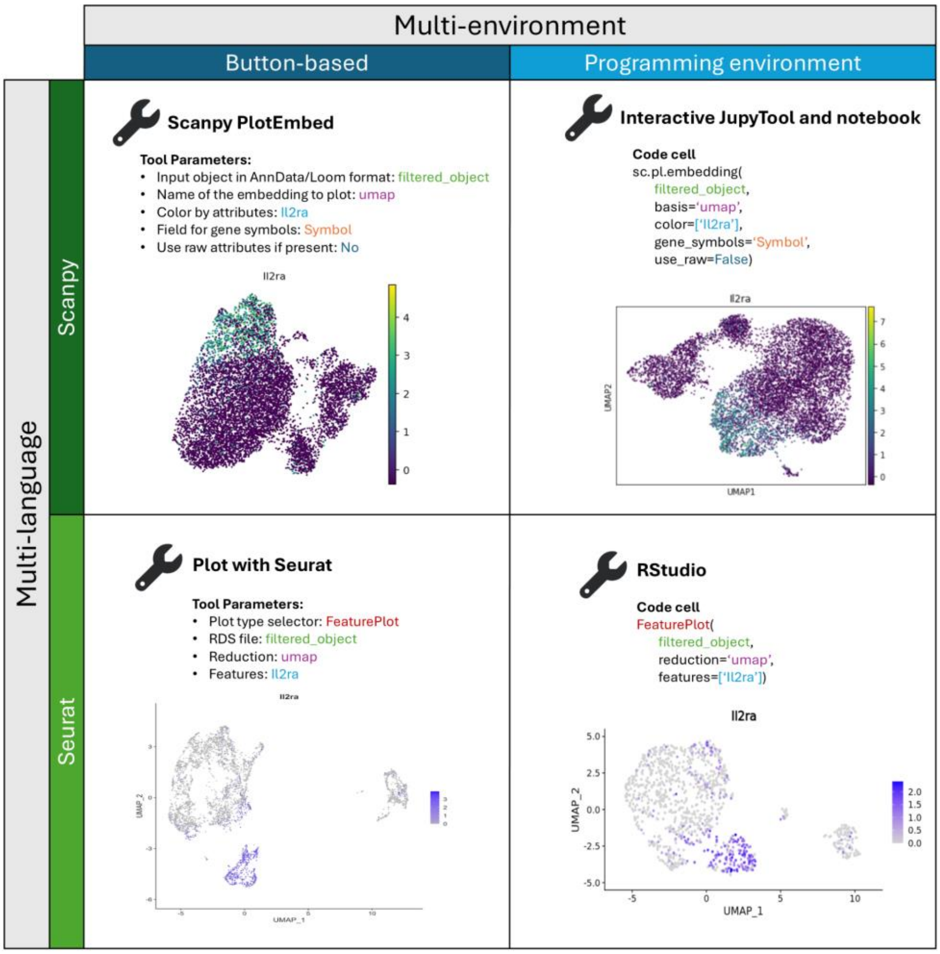

Figure 1. Performing the same step (plotting a marker gene: Il2ra on UMAP embedding) using Galaxy GUI and programming environment as well as Python-based Scanpy and R-based Seurat packages. The resulting plots, although slightly different, represent the same biological information, no matter if BB or PE method was used.

Galaxy’s interactive programming environments [32] are where the PE tutorials take place. Tutorials may be downloaded as RMarkdown or Jupyter notebooks [33, 34], or users may copy, paste, and run each executable code-containing cell from the PE text (Figure 1, Right Column: Programming-environment). Jupyter and RMarkdown notebooks may be exported at the conclusion of each coded tutorial for easy reference or repetition.

Multi-Level

MIGHTS caters to three learning pathways: BB to PE, straight to PE, and PE with BB (Figure 2).

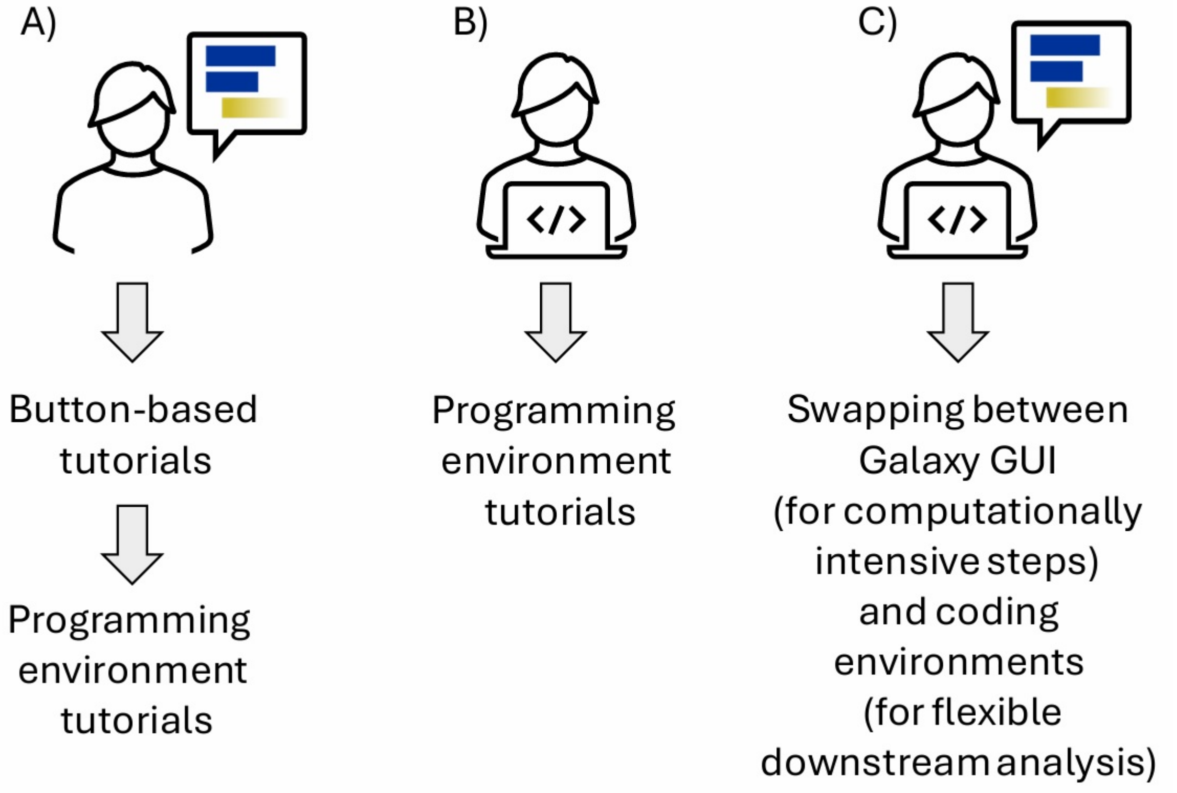

Figure 2. Representation of three possible user journeys using MIGHTS. A) A beginner starting from button-based (BB) tutorials who can then move to programming environment (PE). B) An experienced programmer who can start the analysis directly from the PE, skipping introductory BB tutorials. C) A skilled user who can optimize analyses by swapping between Galaxy GUI to perform computationally intensive steps, and a programming environment for more flexible analyses.

In the first case, BB tutorials guide beginners through the key steps of scRNA-seq analysis, becoming familiar with the methods and learning to interpret results. Then, users repeat the analysis in the PE, focusing on programming skills, while becoming familiar with the languages and libraries commonly used for scRNA-seq analysis (Figure 2A). If a user has experience programming and wants a more flexible analysis, they may begin with the PE tutorials, learning methods with more advanced functionality (Figure 2B). Alternatively, experienced bioinformaticians may utilize Galaxy's Interactive Environments to learn new analyses or run computationally demanding steps that they are unable to locally (Figure 2C).

Multi-Language

scRNA-seq analysis is commonly performed in both R-based (Seurat [35, 36, 37, 38, 39]; Monocle [40]) and Python-based (Scanpy [41]) environments. Therefore, parallel analyses were created across BB and PE and across programming languages–demonstrating multiple methods of analysis and data validation (Figure 3). Users may conduct a typical, full scRNA-seq analysis workflow in R or Python in addition to on a GUI or in a PE.

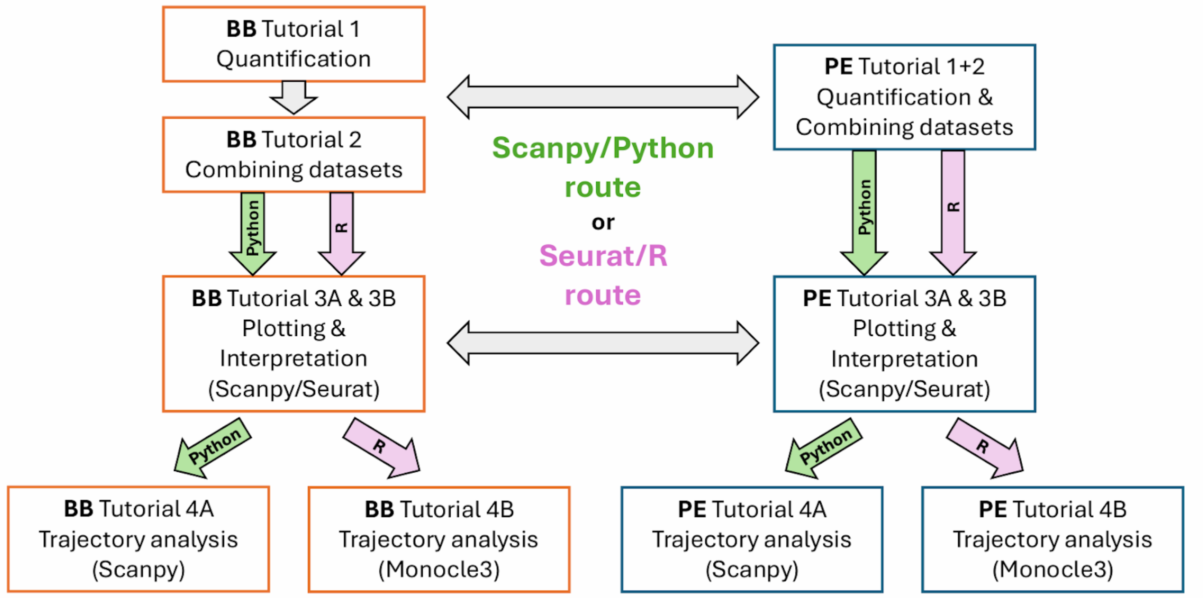

Figure 3. A diagram of the connections of tutorials. It highlights that the languages and packages used in BB and PE tutorials are consistent and allow moving between them easily.

Research-Relevant Skills

MIGHTS demonstrates the use of many frequently used data types and packages for scRNA-seq analyses (Table 1), preparing users with research-relevant skills. Broadly applicable use of programming functions, algorithms, and troubleshooting lends itself to increased creativity and critical thinking [42, 43]. This also improves users' employability and reaches scientists in various research groups, regardless of the method they prefer.

**Analysis**	**Environment | Tutorial (Language)**	**Packages**	**Data Types**
**Pre-processing**	BB | Generating a single-cell matrix using Alevin	Salmon[76] with Alevin [77]	FASTQ

	BB | Combining single-cell datasets after pre-processing	dropletUtils [78, 79] (emptyDrops [78])	FASTA
	PE | Generating a single-cell matrix using Alevin and combining datasets (bash + R)	atlas-gene-annotation-manipulation [80]	GTF
		tximeta [81] (PE)	SingleCellExperiment Object
		biomaRt [82, 83] (PE)	SummarizedExperiment (PE) AnnData
**Plotting & Interpretation**	BB | Filter, plot and explore single-cell RNA-seq data (Scanpy)	Scanpy [41]	AnnData
	PE | Filter, plot and explore single-cell RNA-seq data (Scanpy, Python)	igraph [84] (PE)	
		louvain [85] (PE)	
		pandas [87] (PE)	
	BB | Filter, plot and explore single-cell RNA-seq data (Seurat)	Seurat [35, 36, 37, 38, 39]	AnnData (for conversion to Seurat)
	PE | Filter, plot, and explore single-cell RNA-seq data (Seurat, R)	Matrix [87] (PE)	Seurat Object
		dplyr [88] (PE)	
**Trajectories**	BB | Inferring single-cell trajectories (Scanpy)	Scanpy [41]	AnnData
	PE | Inferring single-cell trajectories (Scanpy, Python)	fa2 [89] (PE)	
		igraph [84] (PE)	
		louvain [85] (PE)	
		numpy [90] (PE)	
		matplotlib [91] (PE)	
	BB | Inferring single-cell trajectories (Monocle3)	Monocle [40]	Cell Data Set
	PE | Inferring single-cell trajectories (Monocle3, R)	anndata [92] (PE)	AnnData (for conversion to Cell Data Set in PE)
		viridislite [93] (PE)	
		magrittr [94] (PE)	
		Rcpp [95] (PE)	
		biomaRt [82, 83] (PE)	

Table 1. MIGHTS tutorials with used packages and datatypes.

Tutorials

Each tutorial begins with data import. The data used in MIGHTS comes from a published study by Bacon *et al.* 2018 [31], describing a mouse model of fetal growth restriction that is publicly available from the EMBL-EBI ArrayExpress under accession number E-MTAB-6945 and may additionally be explored in the Single Cell Expression Atlas. Tutorials in MIGHTS work with the same data throughout to demonstrate analyses using different methods and tools. Tutorials use real, un-curated data, which has simply been subsampled to enhance computational efficiency. The source data is the same, but each analysis allows import of a unique data file to start. The tutorials are designed to be completed in order, but may be performed out of order–if a user wishes to learn how to cluster cells using Scanpy (RRID:SCR_018139), for example, they may select the dedicated tutorial and start with the provided, pre-processed file.

MIGHTS' full workflow consists of three sequential analyses aligning with standard scRNA-seq pipelines [44] and allowing users to compare results across methods.

Generating a single-cell matrix using Alevin and Combining Datasets

The first two tutorials demonstrate the transformation of a FASTA sequencing file into a count matrix (Figure 4, Fig S1, Fig S2). The BB tutorial describes principles of transcriptome quantification, while the PE tutorial introduces users to the many means of installing required packages. This tutorial will take users from aligned read counts to a Single-Cell Experiment (SCE) object which may be further analyzed and converted in Rstudio (RRID:SCR_000432) or Jupyter Notebook.

Users first generate a transcript-to-gene map using FASTQ files, a GTF file, and a reference FASTA transcriptome. A Salmon index of the transcriptome is created, and a cell-by-gene count matrix is built using Alevin. The BB tutorial combines these two steps using one Galaxy tool and demonstrates basic quality control checks including a description of the barcode rank plot "knee detection" method.

The PE tutorial identifies empty droplets, adds cell and gene level metadata, and flags empty droplets based on transcript count. Droplet annotation is corrected for false discovery and the matrix is filtered before combining the datasets manually. PE users save and export files while converting formats to SCE so they are compatible with downstream analyses.

The BB tutorial incorporates metadata straight from a GTF file using a tool to extract gene names and IDs and to label mitochondrial transcripts. The generated gene information is assigned to the matrix, which is subsequently transposed for compatibility with tools meant for 10x Genomics software. EmptyDrops is then used to remove empty droplets.

Half of the remaining suite emphasizes use of AnnData compatible packages. To prepare users, tutorials conclude with one final format conversion from SCE to AnnData with the SCEasy tool. Once each of the objects have been converted, the BB user concatenates them with a Galaxy tool. The BB tutorial sets the user and their objects up for the next tutorial by adding a number of useful metrics to help visualize the data in the coming tutorial(s). Workflows for each tutorial topic are shown in Figure 4.

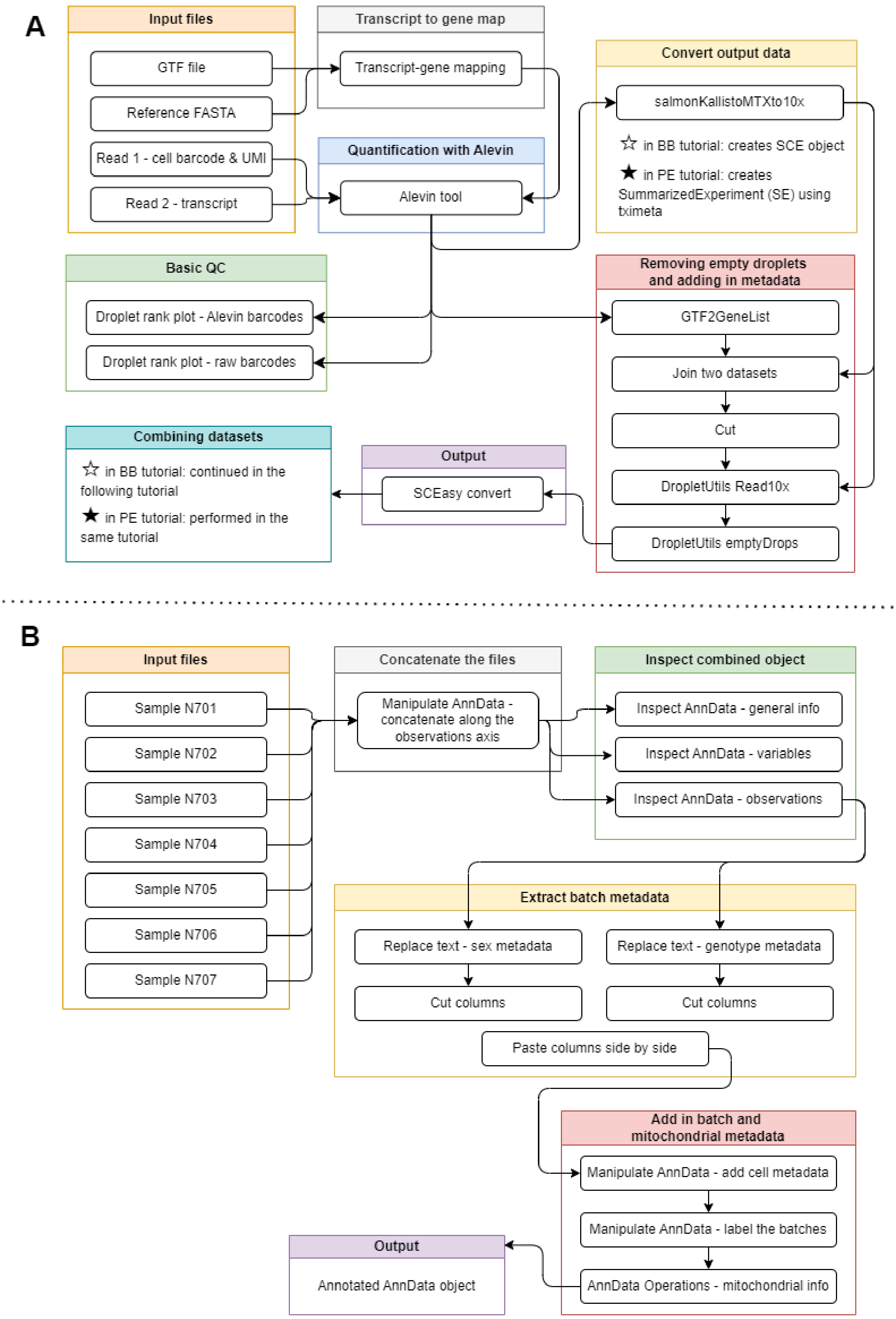
A
Input files
GTF file
Reference FASTA
Read 1 - cell barcode & UMI
Read 2 - transcript
Transcript to gene map
Transcript-gene mapping
Quantification with Alevin
Alevin tool
Convert output data
salmonKallistoMTXto10x
☆ in BB tutorial: creates SCE object
★ in PE tutorial: creates SummarizedExperiment (SE) using tximeta
Basic QC
Droplet rank plot - Alevin barcodes
Droplet rank plot - raw barcodes
Removing empty droplets and adding in metadata
GTF2GeneList
Join two datasets
Cut
DropletUtils Read10x
DropletUtils emptyDrops
Combining datasets
☆ in BB tutorial: continued in the following tutorial
★ in PE tutorial: performed in the same tutorial
Output
SCEasy convert
B
Input files
Sample N701
Sample N702
Sample N703
Sample N704
Sample N705
Sample N706
Sample N707
Concatenate the files
Manipulate AnnData - concatenate along the observations axis
Inspect combined object
Inspect AnnData - general info
Inspect AnnData - variables
Inspect AnnData - observations
Extract batch metadata
Replace text - sex metadata
Replace text - genotype metadata
Cut columns
Cut columns
Paste columns side by side
Add in batch and mitochondrial metadata
Manipulate AnnData - add cell metadata
Manipulate AnnData - label the batches
AnnData Operations - mitochondrial info
Output
Annotated AnnData object

Figure 4. A diagram of workflows in the pre-processing tutorials. A) Workflow for tutorial "Generating a single-cell matrix using Alevin." Solid stars denote steps specific to the PE tutorial while unfilled stars represent BB specific ones. B) Workflow for tutorial "Combining single-cell datasets after pre-processing." All steps featured in the BB tutorial are combined with A's workflow in the PE. A figure of extracted Galaxy workflow is available in the Supplementary Data, Figures S1, S2.

Filter, plot, and explore with Scanpy

These tutorials filter and analyze a pre-processed scRNA-seq matrix using Scanpy (Figure 5). PE users leverage Python via Jupyter Notebook to filter the data for noise, accomplish common visualizations, and differential expression analysis across clusters for the purpose of cell type labeling.

The PE tutorial imports a raw AnnData file and demonstrates storage as a pandas dataframe, while users iteratively visualize data with violin and scatter plots to determine filtering thresholds. Users filter the data to remove technical artifacts and poor quality cells. The PE alternatively uses Boolean indexing for this rather than Scanpy's built-in functions. Users remove transcripts no longer expressed in more than three cells and are prompted to compare different thresholds for the filtering of genes.

Log normalization aligns gene expression along a normal distribution. The PE tutorial includes a description of how normalization works and what other methods exist. Variable genes are flagged for use in more computationally demanding steps. Scaling the data ensures all genes have equal variance and a zero mean, creating a matrix which is compatible with downstream analyses.

Users next reduce the dimensionality of the matrix to allow visualization and interpretation. Principal component analysis (PCA) is performed to calculate the most descriptive principal components (PCs). Users plot PCs against the standard variation they describe, visualizing how PCs relate to variance in their data. The PCs are used to compute a k-nearest neighbors graph, storing a representation of connections between and across cells. Final dimensionality reductions are performed with t-distributed Stochastic Neighbor Embedding (tSNE) [45] and Uniform Manifold Approximation and Projection (UMAP) [46] - both methods reducing the data down to two dimensions for visualization.

Scanpy's clustering function(s) assign each cell to a cluster based on transcriptomic similarity. The tutorials describe clustering algorithms and prompt users to experiment with multiple clustering resolutions, adjusting such that the assigned clusters visually represent what is understood to be biologically accurate. Scanpy's rank_genes_groups identifies the most representative transcripts for each cluster and genotype and PE users transform the output into a data frame.

Users visualize all three dimensionality reductions, different clustering resolutions, and the expression of marker genes. A table of marker genes per cell type from the literature is provided so that the user may inspect their expression patterns and map them to the correct cluster(s). Users label each cluster with a cell type, and plots are saved to the Galaxy history or notebook to be exported. BB users are additionally introduced to the CELLxGENE

(RRID:SCR_021059) [47] tool: an interactive environment for visualizing and exploring scRNA-seq data. Workflow is shown in Figure 5 & Fig S3.

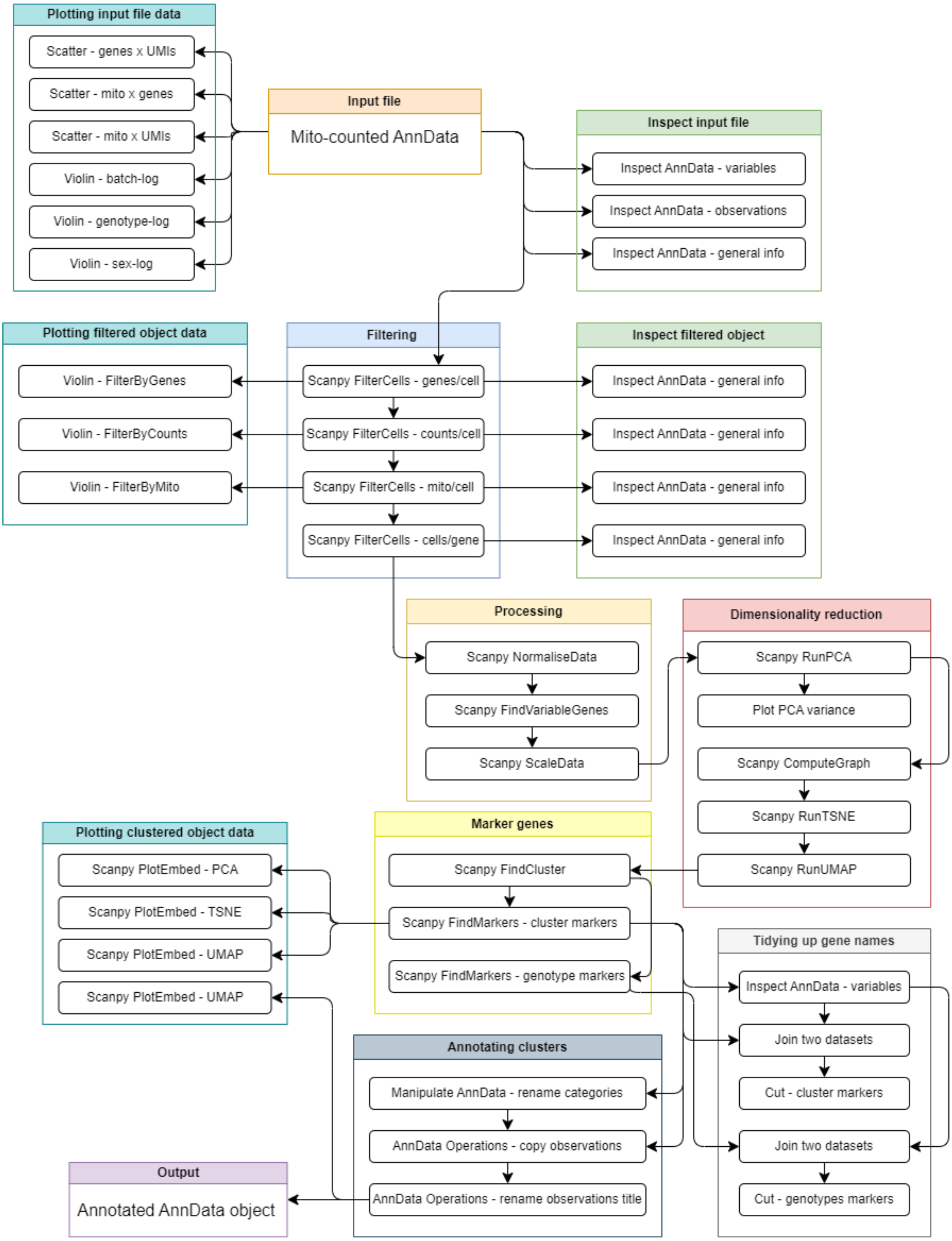

Figure 5. Workflows of plotting & interpretation tutorials: Filter, Plot, and Explore with Scanpy. Features creation of single-cell objects, normalizing data, identifying variable genes, performing dimensionality reduction, identifying clusters, finding marker genes, and interpreting plots. A figure of extracted Galaxy workflow is available in the Supplementary Data, Figure S3.

Filter, plot, and explore with Seurat

These tutorials closely resemble the workflows of the preceding Scanpy ones, this time making use of R package, Seurat. The workflows teach users the basics of scRNA-seq data analysis including typical preprocessing, basic visualization with FeaturePlots, DimPlots, and UMAPs, as well as an exploration of differentially expressed genes across clusters and experimental variables (Figure 6 & Fig S4).

Users import raw counts in both the PE and BB pathways. PE users transition to Galaxy's Interactive RStudio environment, where they are shown how to set up an environment, and given an explanation of how and why packages must be loaded prior to use, as well as how to use Galaxy's gx_get() function for data import. Users manually change the column names of the experimental design data for compatibility with Seurat.

Users next generate a Seurat object: BB users with Seurat's Read10X function, and PE users by manually applying barcode and feature labels to the matrix for input to Seurat's CreateSeuratObject function. Each method is accompanied by descriptions of the alternatives for creating the same Seurat object.

Users apply cell level metadata to their objects. PE users incorporate percent of gene expression (per cell) mapping to the mitochondrial genome–a commonly used parameter for quality control and filtering. Tools are currently being updated to enable BB users to do the same.

Users produce and interpret quality control plots to identify filtering thresholds: assessing potential confounds in the data and developing an understanding of how different variables drive this process. The purpose and theory behind commonly used filtering parameters are described so that users may bring the same (or different) strategies to their own analyses. PE users are additionally shown how to preview the number of cells which would be included based on their choice of filtering parameters.

Both users subset their Seurat object–removing cells outside the chosen threshold(s). PE users additionally remove genes which are now expressed at such low frequencies that they will not contribute biological insight.

Next, users process their filtered object. In the BB, processing of the data includes sequentially normalizing the data, identifying variable features, and scaling. In a more recent update to Seurat's workflow, the SCTransform function [48, 49] was introduced, which combinatorially conducts the three steps in a manner optimized for downstream analyses. SCTransform is used in the PE tutorial while the BB tutorial follows a similar workflow to the one originally published by Seurat. Both users subsequently cover dimensionality reduction via PCA, deciding on the number of PCs to use, finding neighbors, identifying clusters, and UMAP before guided visualization and exploration of the data.

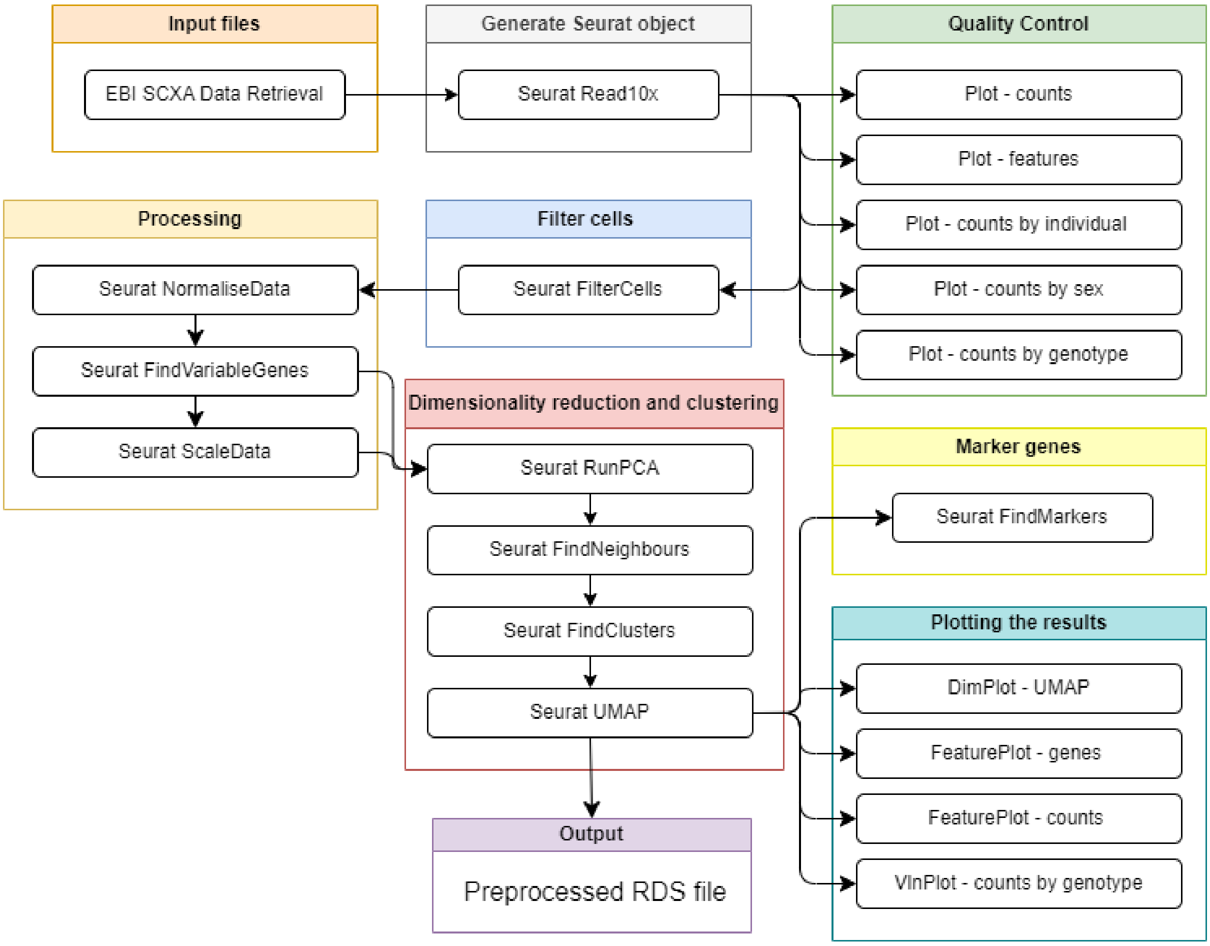

Figure 6. Workflow of Filter, Plot, and Explore tutorial with Seurat. Features generation of a Seurat object, quality control plots, filtering cells, processing, dimensionality reduction, clustering, finding marker genes and creating many plots to analyze the results. A figure of extracted Galaxy workflow is available in the Supplementary Data, Figure S4.

Inferring single-cell trajectories with Scanpy

Trajectory inferences (TI), or pseudotime analysis, provides an alternative means of grouping cells based on gradients of expression. It is worth noting that not all TI algorithms are fit for all datasets—these tutorials begin to explore the reasons why and guide users through the decision-making process. These parallel tutorials conduct typical TI pipeline using Galaxy buttons or in a Python coded environment to characterize transitions between cell states using Scanpy.

Tutorials are significantly based on Scanpy documentation, beginning with import of an annotated AnnData object into Galaxy. Users filter the data to retaining a single cell type. The PE tutorial additionally demonstrates installation of modules before transferring their h5ad data to their Jupyter Notebook with the Galaxy-Jupyter cross-talk feature.

Users calculate force directed graphs (FDGs): representing the data more appropriately for TI than the previously generated tSNE or UMAP visualizations [50]. Optionally, they may create diffusion maps: which can be used in place of PCs to re-compute the nearest neighbors visualized in the FDGs.

Both BB and PE users order cells in pseudotime using Scanpy's diffusion approach, which accepts root cluster assignment indicating to the algorithm which population of cells the trajectory begins with. Users visualize inferred trajectories colored by pseudotime, as well as save, and export their data, plots, and notebook. Users are encouraged to consider other changes across the identified trajectories beyond the scope of the tutorial.

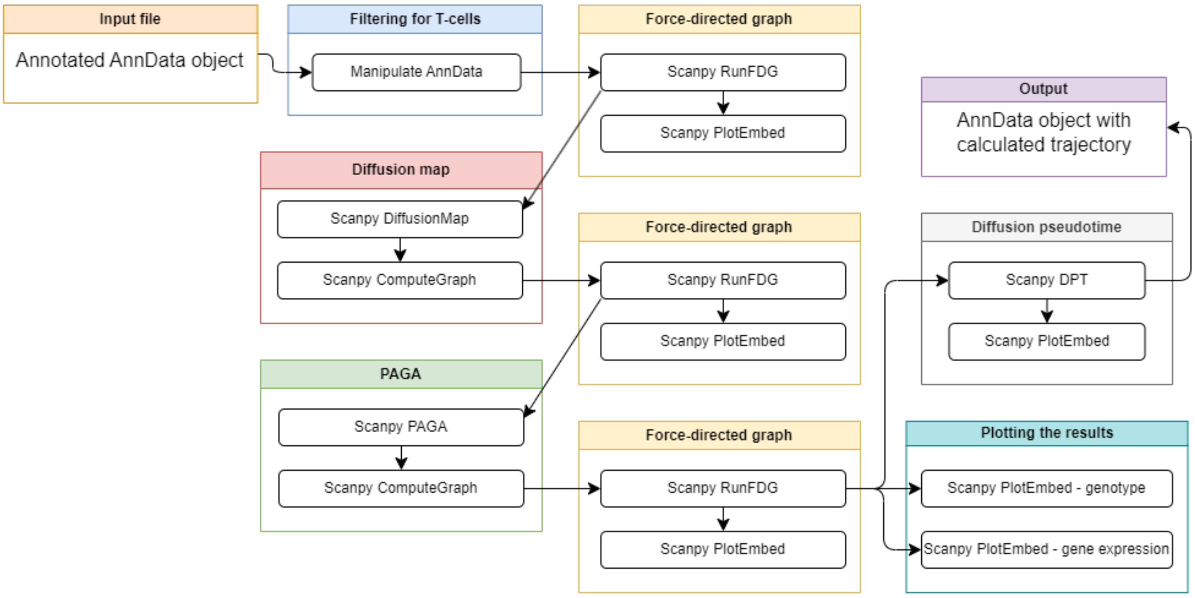

Figure 7. Workflow of inferring trajectories with Scanpy tutorial. Features methods such as force-directed graphs, diffusion maps and PAGA used to infer the cells trajectory in pseudotime. A figure of extracted Galaxy workflow is available in the Supplementary Data, Figure S5.

Inferring single-cell trajectories with Monocle3

Similarly to the aforementioned, the Monocle3 tutorials teach users to conduct trajectory inference (TI) (Figure 8 & Fig S6). These tutorials demonstrate the variability that may arise when trajectories are inferred by different algorithms–this time using the algorithms employed by Monocle3. PE users may implement RStudio or Jupyter Notebook through Galaxy's Interactive Environments. In collaboration with the Scanpy TI tutorial, users accomplish another TI method to additionally validate their results.

PE users are shown the installation of necessary libraries and modules, they import a filtered AnnData object, and familiarize themselves with the data's structure. They extract the expression matrix, cell, and gene metadata, and prepare them for generation of a Cell Data Set (CDS) object–Monocle's preferred data type–with format and column name changes, as well as transposition. BB users may import a CDS file ready for downstream analysis in Monocle or the precursor files to create a CDS manually.

PE users utilize the BioMart database to retrieve gene symbols and associated gene IDs. Although not necessary to complete the tutorial's workflow, this ability is of use to users analyzing their own data.

Users preprocess with Monocle3 beginning with dimensionality reduction. PCA is the method used in these tutorials, although Latent Semantic Indexing (LSI), UMAP, and tSNE

options are also available. PE users visualize each PC in relation to gene variance: to identify how many PCs are needed to capture appropriate variability. Users are provided with visualizations of the output data given different choices in PC.

BB users plot the data in a PCA space, visualizing the effects of various experimental design variables. PE users may optionally correct for batch effects and enjoy customizable plots for a more tailored analysis prior to final dimensionality reduction.

Users cluster the data using Monocle3 as the tutorial describes the differences between clusters and partitions. The PE tutorial additionally demonstrates manual partitioning of cells: an important step for reliable trajectory inference.

The PE tutorial demonstrates three combined means of assigning cell types to the clusters– a supervised, unsupervised, and automated method. Users next infer trajectories relying on Monocle's trajectory graph. Once cells have been ordered in pseudotime starting from the user-directed root cell, cells are visualized colored by pseudotime. BB users end here, comparing the results of the Monocle3 derived trajectory with the Scanpy algorithm's.

PE users are presented with more options for differential expression analysis, visualizing results, identifying the visualization method best suited for them, and exporting plots, data, and their Python, or RStudio, notebook. Workflow is demonstrated in Figure 8.

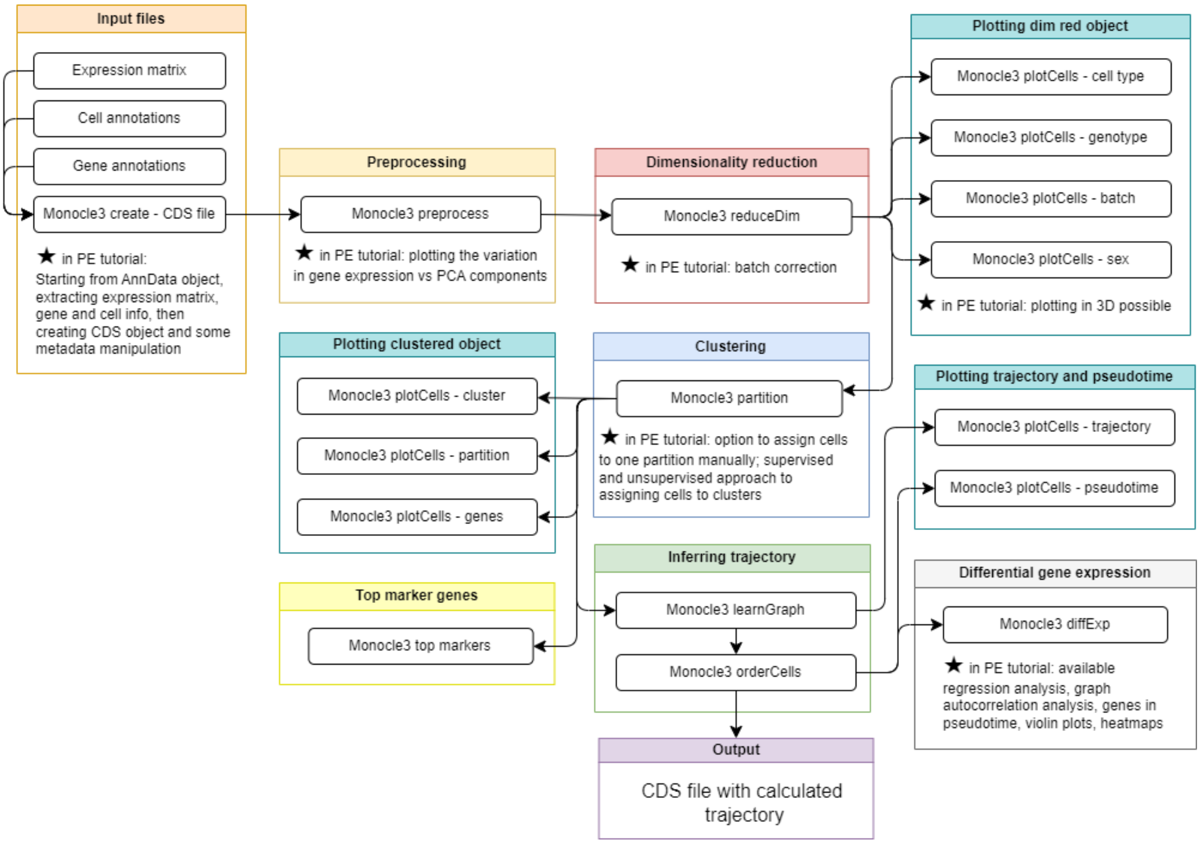

Figure 8. Workflow of Monocle3 inferring trajectories tutorial. Features data type changes for package compatibility, Monocle-specific preprocessing, and trajectory inference on a CellDataSet (CDS) object, followed by differential gene expression. A figure of extracted Galaxy workflow is available in the Supplementary Data, Figure S6.

DISCUSSION

We present MIGHTS, a Multi-Interface Galaxy Hands-on Training Suite, where users may embark on three possible learning trajectories: (1) first learning to analyze scRNA-seq data with buttons in a GUI and subsequently performing the same, more flexible analysis in a programming environment, (2) learning to run the code behind commonly published scRNA-seq analyses, or (3) supplement their pre-existing analyses and skills with Galaxy tools.

MIGHTS performs analysis from raw reads, guiding users through filtering, normalization, dimensionality reduction, data quality assessment, and biological interpretations. The suite demonstrates filtering, clustering, annotation, and trajectory inference for a well-rounded scRNA-seq skill set. Each analysis is demonstrated using methods based on different packages, libraries, and programming languages with the hope that MIGHTS will prepare users to conduct their own, more complex, analyses.

Training features

Users of MIGHTS may start at any step by importing pre-processed input files, using output files from the preceding tutorial, or their own data. Regardless, the analyses will be replicated across languages, methods, and starting points (Figure 9) allowing users to follow the trajectory best suited for their skill level and analysis goals. To facilitate choosing the correct starting point depending on experience and goals, single-cell-oriented Learning Pathways were introduced. “Applying single-cell RNA-seq analysis” [51] and “Applying single-cell RNA-seq analysis in Coding Environments” [52] pathways are based on BB and PE tutorials respectively and may be used to facilitate a smooth transition between button-based tutorials and a programming environment (Figure 2A) or a direct start in the programming environment (Figure 2B). Additionally, to allow for easy identification of the tutorials described here, each tutorial has been tagged and can be found by entering “MIGHTS” in the GTN search box to get access to the relevant materials.

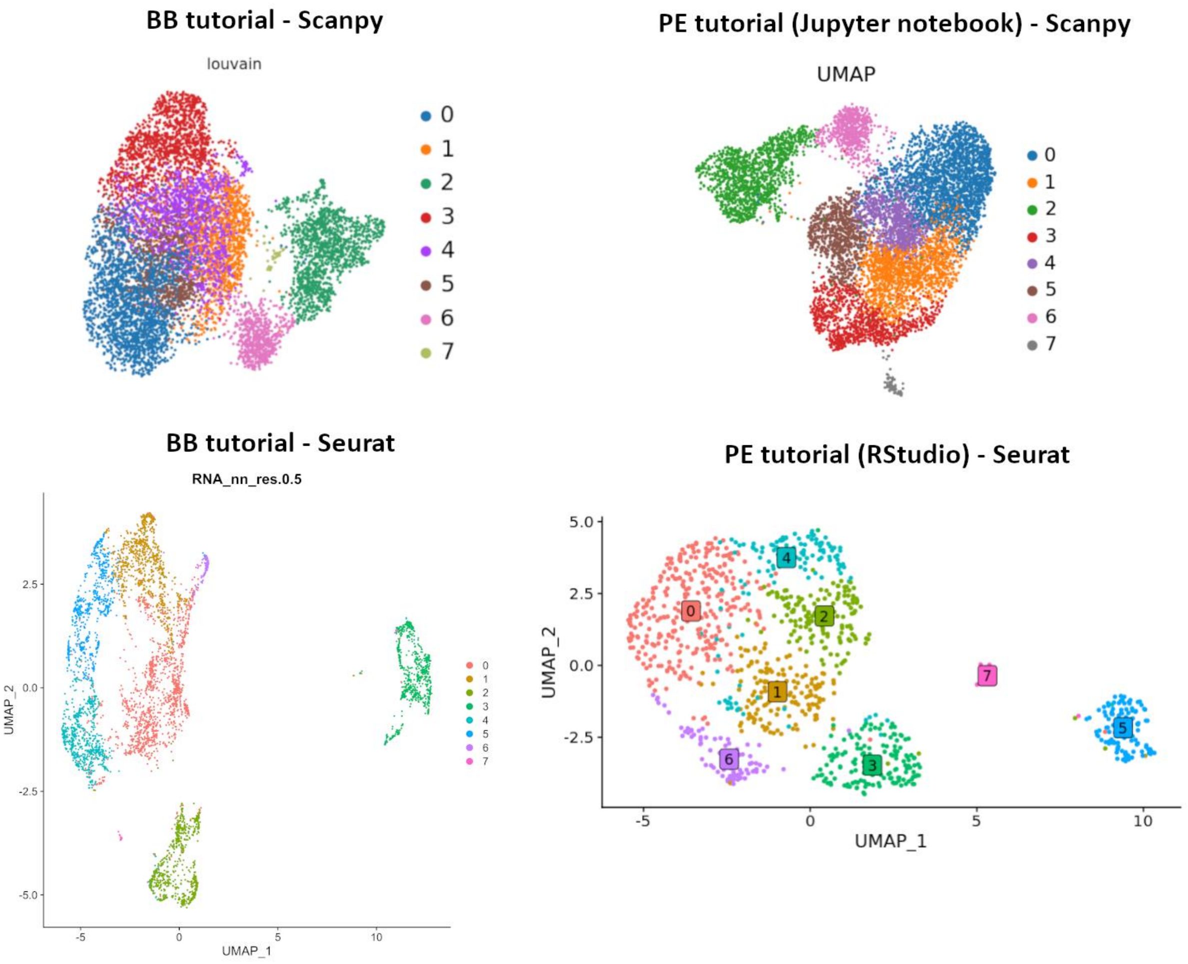

Figure 9. The final 'cluster plot' as an output of plotting & interpretation tutorials across four paths: BB tutorial with Scanpy, PE (Jupyter notebook) with Scanpy, BB tutorial with Seurat, and PE (RStudio) tutorial with Seurat. The numbers correspond to the identified clusters in the dataset. No matter which method (BB vs PE) or language (Scanpy vs Seurat) is used, the biological interpretation is consistent in identifying seven clusters.

Each tutorial builds on the preceding, with no behind-the-scenes data formatting or annotation required between tutorials. With visual examples and analysis of various data types, live training courses found that trainees who performed tutorials during the day could successfully apply the analyses to their own data in the evenings [27, 28].

Learning how to set parameters has long been a difficulty in bioinformatics training [53]. By highlighting parameters that are adjusted often, users learn to prioritize what would otherwise be a never-ending list of decision making. These 'Decision-Time' features enable training for individuals and groups: with the option to vary parameter values and compare results (Figure 10).

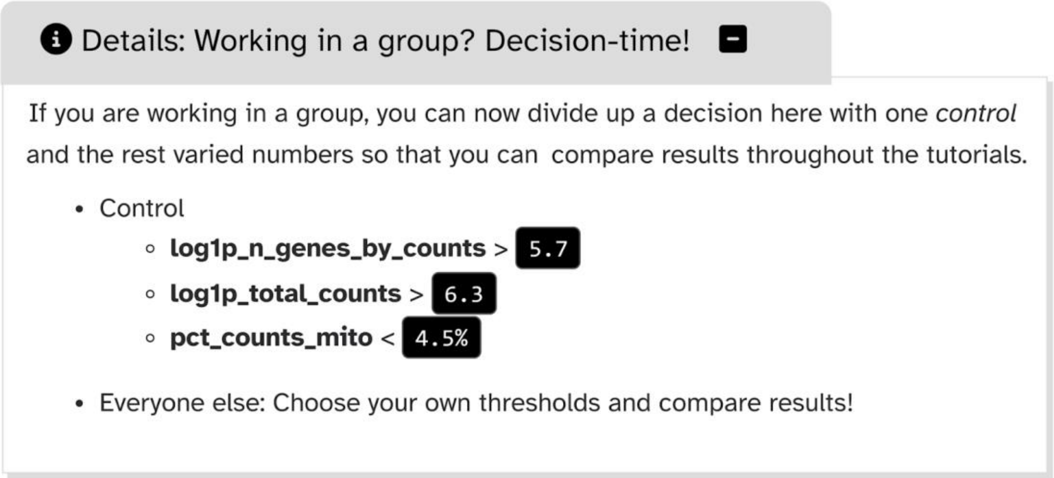

Figure 10. Exemplary 'Decision-Time' feature box in tutorial 'Filter, plot and explore single-cell RNA-seq data (Scanpy)".

Testing of this feature has shown that, broadly, results remain the same regardless of parameter choice, demonstrating the relevance of robust, iterative analyses and data validation [27, 28].

To facilitate effective comprehension and a self-led learning environment, tutorials are interspersed with question boxes and collapsible solutions, allowing users to solifidy their understanding of the material while they learn.

MIGHTS additionally pilots multiple import strategies–ensuring reliability for live training events. This includes direct import from Zenodo [54], import tools linked to data atlases [55], and import from "input" and "answer-key" Galaxy histories–which led to the development of a new feature within the GTN to signpost the option as supporting material (Figure 11).

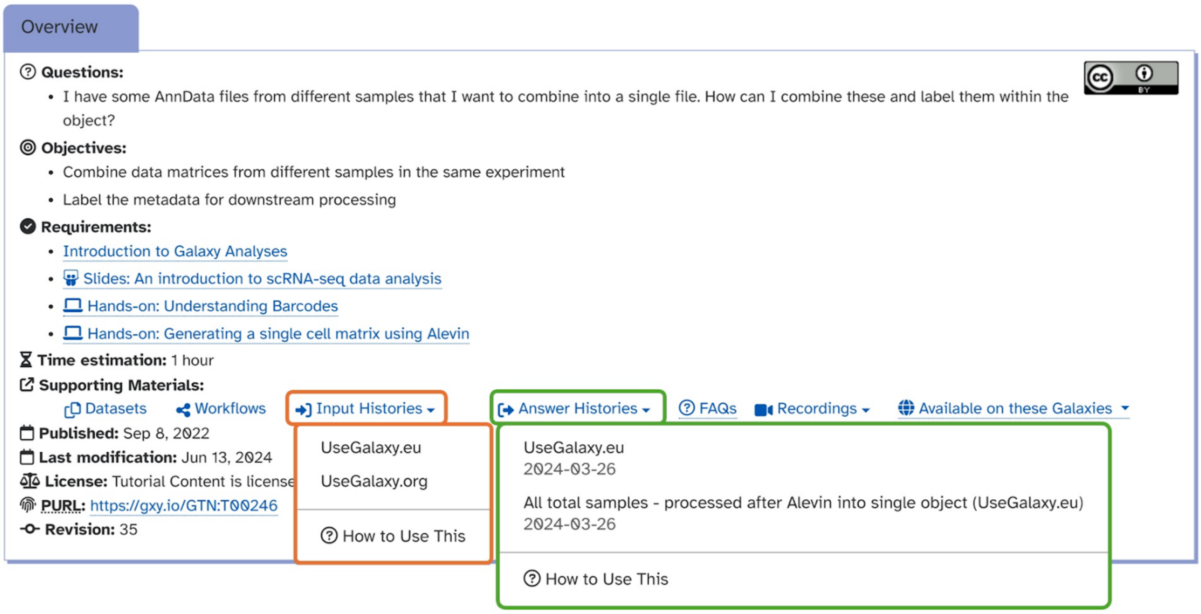

Figure 11. An overview box found at the beginning of the BB tutorial 'Combining single cell datasets after pre-processing'. It showcases a header feature which allows for a quick access to the input histories (orange frame) and answer histories (green frame).

“Answer-key” histories follow datasets along every step of analyses, providing a final contingency for delivering live training and protecting users from frustration. Tutorials are additionally accompanied by slide decks (which can act as a general introduction to the topic), as well as recordings of the step-by-step analysis performed by an instructor.

Learn to Code in a Beginner Friendly Way

As sequencing strategies and tools continue to advance, it is important that the field of bioinformatics “trains the trainer” in response to continued growth. To support comprehension, each tutorial provides detailed explanations of biological and computational concepts including simplified troubleshooting and multiple interactive elements. By showing alternative methods to perform a single analysis, users become familiar with the most common programming languages used in the life sciences: Python and R, as well as command language Bash. PE users additionally begin to learn the syntax and use of R [56] and other programming languages--providing them with well rounded examples of how to analyze scRNA-seq data, or how they may begin to leverage it (and Galaxy) as a means to learn new programming skills [57]. These PE tutorials introduce users to relevant packages, functions, and data types used in today’s published bioinformatic analyses (Table 1).

The transition from Galaxy-button tutorials into the coded environment is facilitated by interactive tools such as RStudio or Jupyter Notebook, such that all the analysis may be completed within Galaxy as opposed to on local instances. Importantly, there is no need for any software installation–all tutorials provide necessary tools to complete them, including example datasets, slides, videos, workflows, and public Galaxy servers where the analysis may be performed. Internet access is the only additional necessary resource [58]. This approach specifically facilitates accessible bioinformatics analyses by eliminating installation hang-ups, minimizing the time spent setting one’s environment, and increasing computing capacity for users.

Additionally, if users embrace programming such that they are looking to program their own button-based tools or create new training material, opportunities to do so exist on GTN pages dedicated to development in Galaxy [59] and contributing to the Galaxy Training Material [60].

FAIR data

MIGHTS tutorials were created on an interface with employed findable, accessible, interoperable, and reusable (FAIR) data usage [59]. The FAIR principles can and should be applied in all life science domains where large amounts of data are produced. FAIR data management is particularly important in scRNA-seq analysis which looks at large expression matrices. Unfortunately, it is often the case that published datasets come with missing, or incomplete, metadata–rendering the dataset less useful than it would be with complete annotation(s). By completing MIGHTS tutorials, users become equipped with the skills helpful in formatting such demanding datasets.

Sustainable

An important feature characterizing MIGHTS is its sustainability. As previously reported, the evolving nature of bioinformatics requires a sustainable bridge between the fields of biology and informatics [60]. Therefore, collaboration between developers and domain experts is critical. The GTN emphasizes that users be included in this collaboration: whereby users have the opportunity to report issues and request additional resources. This facilitates involvement of developers who are aware of user needs and users who are actively contributing to the improvement of materials. Any issues may be reported back to tutorial developers themselves, demonstrating the sustainability of Galaxy's Circle of Life (Figure 12).

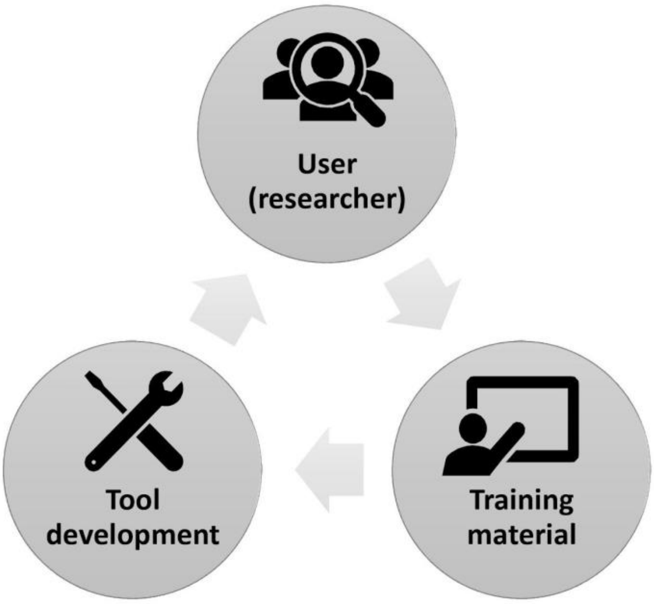

Figure 12. Galaxy Circle of Life demonstrating the interdisciplinary, multi-level sustainability practiced by the GTN. Users report changes they wish to see made in the training material, prompting new tool development and updates which can be sustainably utilized and tested by researchers.

Tutorials on the GTN are, at minimum, updated annually in preparation for the Galaxy Community Conference (GCC). The Galaxy Circle of Life functions such that tutorials will continue to meet evolving user needs–largely thanks to the commitment of the growing Galaxy Single-Cell & Spatial Omics Community. Notably, MIGHTS tutorials have been updated, on average, 7 times a year since their respective publication (Table 2).

Table 2. Number of revisions made to each tutorial featured in MIGHTS as of August 2024.

Tutorial Topic	BB tutorials		PE tutorials	
	Months since tutorial Publication	Number of Revisions	Months since tutorial Publication	Number of Revisions
Generating a single-cell matrix using Alevin	41	16	8	2

Combining single-cell datasets after pre-processing	23	16		
Filter plot explore single-cell rna seq data with Scanpy	40	18	11	9
Filter Plot and explore single-cell RNA-seq data with Seurat	4	3	10	8
Inferring single-cell trajectories with Scanpy	8	5	40	15
Inferring single-cell trajectories with Monocle3	22	19	15	10

The number of revisions demonstrates continued sustainability of tutorials featured in the suite.

Notably, the GTN offers tutorials on additional bioinformatic analyses in a variety of fields. These tutorials are similarly monitored and revised, although the rate of growth specifically for single-cell tutorials is worth noting.

Addressing Modern Challenges in Bioinformatics

MIGHTS addresses many broad challenges of bioinformatics training, emphasizing that effective bioinformatics involves understanding key principles and gaining experience [22] with real world data, problem-solving [24], reproducibility [61], and validation [62].

One challenge in bioinformatics is the application of analyses from training courses to real, messy, lab-generated data. Uniquely, MIGHTS uses raw, un-annotated data from a published analysis: Bacon *et al.* 2018 [31], and guides users through reformatting, annotating, and conducting biological analyses.

Reproducibility and reinstantiation, keystones of quality bioinformatic analyses, are ensured by MIGHTS thanks to published workflows and key histories for each dataset (Figure 11). Workflows are available on the Galaxy servers, providing a stable way to perform a particular analysis in an identical environment. By linking detailed tool versions to the tutorial workflows themselves, it is possible to submit new input files, adjust parameter thresholds, and wait for an output. This is particularly helpful for analyzing multiple samples that require the same pipeline, allowing reproducible results, minimizing time spent re-running code, and eliminating the need for complex coding skills to develop pipelines.

To address another challenge of the field, MIGHTS emphasizes the importance of validating one's results: to determine whether results reflect an actual biological process versus artifacts of the pipeline. Using tools based in various programming languages and multiple algorithms allows users to feel confident that their results are uncovering true biological insights no matter the analysis method used (Figure 9). MIGHTS can act as a guide on how to validate results. The suite may additionally be used directly by novice to intermediate bioinformaticians to check whether their results are consistent via alternative methods without needing to learn another programming language.

Because bioinformatics combines numerous STEM fields, it faces interdisciplinary and inter-generational challenges [63]. Software developers often do not understand the underlying biology their programs analyze and biologists often do not know how analytical algorithms function [65]. MIGHTS aims to fill this gap by introducing step-by-step analyses, while simultaneously demonstrating the biological interpretation of results and how they were uncovered. Coded tutorials provide additional opportunities to become familiar with the algorithms behind the analyses. The suite can act as a resource to educate and inspire future generations of bioinformaticians: ones who are able to speak across disciplines, effectively identifying areas for improvement, and building flexible, long term solutions.

Limitations and further steps

The main limitation of the Galaxy GUI tutorials is that the analyses are limited to the packages and functions which have been wrapped into tools. As such, some analysis steps might be limited in the BB tutorials. However, users have the opportunity to submit 'tool requests': an ongoing effort to mitigate this limitation.

Additionally, tool versions must be compatible with one another. To mitigate this limitation, tools are regularly tested and updated to allow for analysis using the most recent versions and ensuring outputs are compatible inputs for downstream steps. Issues with tools may be reported on Galaxy forums, where experts and developers respond quickly to issues.

The main limitations of the PE tutorials are limited resources allocated to Interactive Environments, and inconsistencies between the notebooks on different public Galaxy servers (.eu vs .org vs .au). However, the educational purpose of the coded-tutorials is to familiarize users with coding environments, so downsampled data provides the same benefits and enables most analyses to be done within the resource limit. Even so, should a user need or want more resources allocated, they can request that from the Galaxy admins.

There are ongoing efforts to expand the functionality of MIGHTS to enable more bespoke analyses of datasets, in response to community needs.

AVAILABILITY OF SOURCE CODE AND REQUIREMENTS

- Project name: Multi-Interface Galaxy Hands-on Training Suite for scRNA-seq
- Project home page: https://github.com/galaxyproject/training-material/tree/main/topics/single-cell/tutorials
- Operating system(s): web-based, platform independent
- Programming languages: R, Python, Bash
- License: MIT

Data Availability

All the tutorials are available at dedicated Single Cell subpage of Galaxy Training Network (GTN) [96].

The used experimental data comes from a published study by Bacon *et al.* 2018 [31], that is publicly available from the EMBL-EBI ArrayExpress under accession number E-MTAB-6945

and can also be browsed from Single Cell Expression Atlas. The input datasets used in tutorials are stored at Zenodo [97-102] and all generated data files are available in the shared Galaxy histories, included in each tutorial.

The tutorials comprise many different tools that can be freely used at the Galaxy public servers, such as Galaxy Europe [103], Galaxy US [104], Galaxy Australia [105] and others. The tool wrappers with detailed information are stored at the Galaxy ToolShed [106].

DECLARATIONS

Abbreviations

BB: button-based
CDS: Cell Data Set
DPT - Diffusion Pseudotime
EMBL-EBI: European Molecular Biology Laboratory - European Bioinformatics Institute
FAIR: Findability, Accessibility, Interoperability, Reusability
FDG: Force-directed Graph
GCC: Galaxy Community Conference
GTF: Gene Transfer Format
GTN: Galaxy Training Network
GUI: Graphical User Interface
LSI: Latent Semantic Indexing
MIGHTS: Multi-Interface Galaxy Hands-on Training Suite for scRNA-seq
PAGA: Partition-Based Graph Abstraction
PC: Principal Component
PCA: Principal Component Analysis
PE: programming environment
QC: Quality Control
SCE: SingleCellExperiment
scRNA-seq: single cell RNA sequencing
SE: SummarizedExperiment
STEM: Science, Technology, Engineering and Mathematics
TI: Trajectory Inference
tSNE: t-distributed Stochastic Neighbor Embedding
UMAP: Uniform Manifold Approximation and Projection

Ethics approval and consent to participate

Not applicable.

Consent for Publication

Not applicable.

Competing Interests

The author(s) declare that they have no competing interests.

Funding

Internships were funded in part by the Engineering & Physical Sciences Research Council (UK) as well as Hobart and William Smith Colleges (Geneva, NY, USA). Additionally, part of the materials were created thanks to the Third Training Open Call issued by EOSC-Life which has received funding from the European Union's Horizon 2020 programme under grant agreement number 824087.

Authors' Contributions

Conceptualization: Wendi Bacon; Data curation: Wendi Bacon, Helena Rasche; Formal analysis: Wendi Bacon, Camila Goclowski, Morgan Howells, Julia Jakiela, Marisa Loach, Jonathan Manning; Funding acquisition: Wendi Bacon; Investigation: Wendi Bacon, Camila Goclowski, Morgan Howells, Julia Jakiela, Marisa Loach, Jonathan Manning; Methodology: Wendi Bacon, Camila Goclowski, Morgan Howells, Julia Jakiela, Marisa Loach, Jonathan Manning; Project administration: Wendi Bacon; Software: Tyler Collins, Saskia Hiltemann, Pablo Moreno, Alex Ostrovsky, Helena Rasche, Mehmet Tekman, Pavankumar Videm; Resources: Tyler Collins, Saskia Hiltemann, Alex Ostrovsky, Helena Rasche, Mehmet Tekman, Pavankumar Videm, Wendi Bacon; Supervision: Wendi Bacon; Validation: Wendi Bacon, Marisa Loach, Morgan Howells, Julia Jakiela, Camila Goclowski, Jonathan Manning, Mehmet Tekman, Graeme Tyson, Pavankumar Videm; Visualization (tutorials): Wendi Bacon, Camila Goclowski, Morgan Howells, Julia Jakiela, Marisa Loach, Jonathan Manning; Visualization (manuscript): Julia Jakiela; Writing-original draft: Camila Goclowski, Julia Jakiela; Writing-review & editing: Wendi Bacon, Camila Goclowski, Julia Jakiela, Marisa Loach, Pavankumar Videm
Both first authors contributed equally and may state their name first on CV as well as should receive equal credit for contributions made.

ACKNOWLEDGEMENTS

The authors extend their gratitude to the Galaxy community for supporting the testing of workflows as well as the development of tools. We thank Gareth Price for support testing on the Australian Galaxy instance and training course participants for testing tutorials in live user settings.

Co-first authors CG and JJ contributed equally to this publication's curation and manuscript preparation. As such, they receive equal credit in the publication.

REFERENCES

[1] Attwood TK, Schneider MV, Brazas MD. A global perspective on evolving bioinformatics and data training needs. Briefing in Bioinformatics 2017 doi: doi.org/10.1093/bib/bbx100 (Attwood et al. 2019)

[2] Goodman N. Biological data becomes computer literate: new advances in bioinformatics. Current Opinion in Biotechnology 2002. https://doi.org/10.1016/S0958-1669(02)00287-2 (Goodman 2002)

[3] Mitra D, Bensaad MS, Sinha S, et al. Evolution of bioinformatics and its impact on modern bio-science in the twenty-first century: Special attention to pharmacology, plant science and drug discovery,
Computational Toxicology. 2022. doi: doi.org/10.1016/j.comtox.2022.100248

[4] Singh S, Pandey AK, and Prajapati VK. Chapter One - From genome to clinic: The power of translational bioinformatics in improving human health. Advances in Protein Chemistry and Structural Biology, Academic Press. 2024. doi: doi.org/10.1016/bs.apcsb.2023.11.010

[5] Dhiman K and Dhiman H. Unveiling the World of Bioinformatics. Applying Machine Learning Techniques to Bioinformatics: Few-Shot and Zero-Shot Methods, edited by Umesh Kumar Lilhore, et al. IGI Global. 2024. doi: doi.org/10.4018/979-8-3693-1822-5.ch010

[6] Wright AM, Schwartz JR, Newman CE. The why, when, and how of computing in biology classrooms. F1000Research 2019. https://doi.org/10.12688/f1000research.20873.2.

[7] Dabholkar S. Computational Thinking in Biology: Part 1. BRidging InterDisciplinary Gaps in Education Sciences. 2021. https://doi.org/10.1007/978-3-540-76639-1_4

[8] Ras V, Carvajal-Lopez P, Gopalasingam P, et al. Challenges and Considerations for Delivering Bioinformatics Training in LMICs: Perspectives From Pan-African and Latin American Bioinformatics Networks. Frontiers in Education 2021. https://doi.org/10.3389/feduc.2021.710971

[9] Chasapi A et al. The bioinformatics wealth of nations. Bioinformatics. 2020. https://doi.org/10.1093/bioinformatics/btaa132.

[10] Erxleben-Eggenhofer A et al. FAIR and Scalable Education The Galaxy Training Network (GTN) and a Training Infrastructure as a Service (TIaaS). Proceedings of the Conference on Research Data Infrastructure. 2023. https://doi.org/10.52825/cordi.v1i.422.

[11] Forero D et al. Current needs for human and medical genomics research infrastructure in low and middle income countries. Journal of Medical Genetics. 2016. https://doi.org/10.1136/jmedgenet-2015-103631.

[12] Mendy M et al. Infrastructure and Facilities for Human Biobanking in Low- and Middle-Income Countries: A Situation Analysis."Pathobiology. 2014. https://doi.org/10.1159/000362093.

[13] Pérez-Wohlfeil E, Torreno O, Bellis, L, et al. Training bioinformaticians in High Performance Computing. Heliyon. 2018. https://doi.org/10.1016/j.heliyon.2018.e01057.

[14] Wilson SMA, Hauser C, Sierk M, et al. Bioinformatics core competencies for undergraduate life sciences education. PLoS One. 2018. doi: 10.1371/journal.pone.0196878

[15] Williams JJ, Drew JC, Galindo-Gonzalez S, et al. Barriers to integration of bioinformatics into undergraduate life sciences education: A national study of US life sciences faculty

uncover significant barriers to integrating bioinformatics into undergraduate instruction. PLoS ONE 2019. https://doi.org/10.1371/journal.pone.0224288.

[16] Katara, P. Role of bioinformatics and pharmacogenomics in drug discovery and development process. Network Modeling Analysis Health Informatics and Bioinformatics. 2013. doi: doi.org/10.1007/s13721-013-0039-5

[17] Levine AG. An explosion of bioinformatics careers. Science. 2014; doi: 10.1126/science.344.6189.1303

[18] Hwang B, Lee JH, Bang D. Single-cell RNA sequencing technologies and bioinformatics pipelines. Experimental and Molecular Medicine. 2018. doi: doi.org/10.1038/s12276-018-0071-8

[19] Navlakha S, Bar-Joseph Z. Algorithms in nature: the convergence of systems biology and computational thinking. Molecular Systems Biology 2011. https://doi.org/10.1038/msb.2011.78

[20] Carey MA, Papin J. Ten simple rules for biologists learning to program. PLoS Computational Biology 2018. https://doi.org/10.1371/journal.pcbi.1005871.

[21] Via A, Blicher T, Bongcam-Rudloff E, et al. Best practices in bioinformatics training for life scientists. Briefings in Bioinformatics 2013. https://doi.org/10.1093/bib/bbt043

[22] Dudley J et al. A Quick Guide for Developing Effective Bioinformatics Programming Skills. PLoS Computational Biology. 2009. https://doi.org/10.1371/journal.pcbi.1000589.

[23] Jazayeri M et al. Combining Mastery Learning with Project-Based Learning in a First Programming Course: An Experience Report. 2015 IEEE/ACM 37th IEEE International Conference on Software Engineering. 2015. https://doi.org/10.1109/ICSE.2015.163.

[24] Perkins DN et al. Conditions of Learning in Novice Programmers. Journal of Educational Computing Research. 1986. https://doi.org/10.2190/GUJT-JCBJ-Q6QU-Q9PL.

[25] Shafto SA. Programming for learning in mathematics and science. Association for Computing Machinery 1986. https://doi.org/10.1145/953055.5635

[26] Johnston IG, Slater M, Cazier JB. Interdisciplinary and Transferable Concepts in Bioinformatics Education: Observations and Approaches From a UK MSc Course. Curriculum, INstruction, and Pedagogy: Sec. STEM Education. 2022. https://doi.org/10.3389/feduc.2022.826951

[27] Hiltemann S, Rasche H, Gladman S, et al. Galaxy Training: A powerful framework for teaching! PLoS Computational Biology. 2023. https://doi.org/10.1371/journal.pcbi.1010752

[28] Rasche H, Hyde C, Davis J, et al. Training Infrastructure as a Service. GigaScience 2023. https://doi.org/10.1093/gigascience/giad048

[29] Bacon W, Holinski A, Pujol M, et al. Ten simple rules for leveraging virtual interaction to build higher-level learning into bioinformatics short courses. PLoS Computational Biology 2022. https://doi.org/10.1371/journal.pcbi.1010220

[30] Moreno P, Huang N, Manning JR, et al. User-friendly, scalable tools and workflows for single-cell RNA-seq analysis. Nature Methods. 2021. doi: doi.org/10.1038/s41592-021-01102-w

[31] Bacon WA, Hamilton RS, Yu Z, et al. Single-Cell Analysis Identifies Thymic Maturation Delay in Growth-Restricted Neonatal Mice. Frontiers in Immunology. 2018. https://doi.org/10.3389/fimmu.2018.02523

[32] Gruning B, Rasche E, Rebolledo-Jaramillo B, et al. Jupyter and Galaxy: Easing entry barriers into complex data analyses for biomedical researchers. PLoS Computational Biology 2017. doi: doi.org/10.1371/journal.pcbi.1005425

[33] Baumer B, Udwin D. R markdown. Wiley Interdisciplinary Reviews: Computational Statistics. 2015. https://doi.org/10.1002/wics.1348

[34] Ragan-Kelley M, Perez F, Granger B, et al. The Juyter/iPython architecture: a unified view of computational research, from interactive exploration to communication and publication. American Geophysical Union. 2016. https://ui.adsabs.harvard.edu/abs/2014AGUFM.H44D..07R/abstract

[35] Satija R, Farrell JA, Gennert D, Schier AF, Regev A (2015). “Spatial reconstruction of single-cell gene expression data.” Nature Biotechnology, 33, 495-502. https://doi.org/10.1038/nbt.3192.

[36] Hao Y, Stuart T, Kowalski MH, et al. Dictionary learning for integrative, multimodal and scalable single-cell analysis. Nature Biotechnology. 2023. doi: doi.org/10.1038/s41587-023-01767-y.

[37] Hao Y, Hao S, Andersen-Nissen E, et al. (2021). “Integrated analysis of multimodal single-cell data.” Cell. 2021. doi: doi.org/10.1016/j.cell.2021.04.048

[38] Stuart T, Butler A, Hoffman P, et al. Comprehensive Integration of Single-Cell Data. Cell. 2019. doi: doi.org/10.1016/j.cell.2019.05.031

[39] Butler A, Hoffman P, Smibert P, et al. Integrating single-cell transcriptomic data across different conditions, technologies, and species. Nature Biotechnology. 2018. doi: doi.org/10.1038/nbt.4096.

[40] Cao, J. et. al. The single-cell transcriptional landscape of mammalian organogenesis. Nature. 2019. doi: doi.org/10.1038/s41586-019-0969-x

[41] Wolf FA, Angerer P, Theis FJ. SCANPY: large-scale single-cell gene expression data analysis. Genome Biology. 2018. https://doi.org/10.1186/s13059-017-1382-0

[42] Scherer R, Siddiq F, Sánchez-Scherer B. Some Evidence on the Cognitive Benefits of Learning to Code. Front Psychol. 2021 Sep 9;12:559424. doi: 10.3389/fpsyg.2021.559424.

[43] Shute VJ, Sun C, Asbell-Clarke J. Demystifying computational thinking. Educational Research Review. 2017. doi: doi.org/10.1016/j.edurev.2017.09.003.

[44] He J, Lihui L, Chen J. Practical bioinformatics pipeline for single-cell RNA-seq data analysis. Biophysics Reports. 2022. doi: 10.52601/bpr.2022.210041

[45] van der Maaten L, Hinton G. Visualizing Data using t-SNE. Journal of Machine Learning Research. 2008. 9(86):2579−2605, 2008.
https://www.jmlr.org/papers/volume9/vandermaaten08a/vandermaaten08a.pdf?fbcl

[46 McInnes L, Healy J, Saul N, et al. UMAP: Uniform Manifold Approximation and Projection. Journal of OPen Source Software. 2018. doi: doi.org/10.21105/joss.00861

[47] Megill C, Martin B, Weaver C, et al. cellxgene: a performant, scalable exploration platform for high dimensional sparse matrices. bioRxiv. 2021. doi: doi.org/10.1101/2021.04.05.438318

[48] Hafemeister C, Satija R. Normalization and variance stabilization of single-cell RNA-seq data using regularized negative binomial regression. Genome Biology. 2019. doi: doi.org/10.1186/s13059-019-1874-1.

[49] Choudhary S, Satija R. Comparison and evaluation of statistical error models for scRNA-seq. Genome Biology. 2022. doi: doi.org/10.1186/s13059-021-02584-9

[50] Ko ME, Williams CM, Fread KI, et al. FLOW-MAP: a graph based, force directed layout algorithm for trajectory mapping in single-cell time course datasets. Nature Protocols. 2020. doi: doi.org/10.1038/s41596-019-0246-3

[51] Galaxy Training. Applying single-cell RNA-seq analysis. https://gxy.io/GTN:P00020

[52] Galaxy Training. Applying single-cell RNA-seq analysis in Coding Environments. https://gxy.io/GTN:P00024

[53] Tractenberg RE, Lindvall JM, Attwood TK, et al. The Mastery Rubric for Bioinformatics: A tool to support design and evaluation of career-spanning education and training. 2019. doi: 10.1371/journal.pone.0225256

[54] Wareham J, Pujol Priego L, Zenodo – Open science monitor case study. European Commission, Directorate-General for Research and Innovation. 2019. https://data.europa.eu/doi/10.2777/298228

[55] Papatheodorou I, Moreno P, Manning J, et al. Expression Atlas update: from tissues to single cells. Nucleic Acids Research. 2020. https://doi.org/10.1093/nar/gkz947

[56] R Core Team (2021). R: A language and environment for statistical computing. R Foundation for Statistical Computing, Vienna, Austria. https://www.R-project.org/.

[57] Camila Goclowski. From GTN Intern to Tutorial Author to Bioinformatician. Galaxy Training. 2024. https://gxy.io/GTN:N00087

[58] Goecks J, Nekrutenko A, Taylor J. Galaxy: a comprehensive approach for supporting accessible, reproducible, and transparent computational research in the life sciences. Genome Biology. 2010. doi: doi.org/10.1186/gb-2010-11-8-r86.

[59] Ouwerkerk J, Rasche H, Spalding JD, et al. FAIR data retrieval for sensitive clinical research data in Galaxy. Gigascience. 2024 doi: 10.1093/gigascience/giad099

[60] Aron S, Chauke PA, Ras V, et al. The Development of a Sustainable Bioinformatics Training Environment within the H3Africa Bioinformatics Network. Frontiers in Education. 2021. doi: doi.org/10.3389/feduc.2021.725702

[61] Cokelaer T, Cohen-Boulakia S, Lemoine F. Reprohackathons: promoting reproducibility in bioinformatics through training. Bioinformatics. 2023. doi: doi.org/10.1093/bioinformatics/btad227

[62] Yang A, Troup M, Ho JWK. Scalability and Validation of BIg Data Bioinformatics Software. Computational and Structural Biotechnology Journal. 2017. doi: doi.org/10.1016/j.csbj.2017.07.002

[63] Bartlett A, Lewis J, Williams M. Generations of interdisciplinarity in bioinformatics. New Genetics and Society. 2016. doi: doi.org/10.1080/14636778.2016.1184965

[64] Garmire DG, Xun Z, Aravind M , et al. GranatumX: A Community-engaging, Modularized, and Flexible Webtool for Single-cell Data Analysis. Genomics, Proteomics & Bioinformatics. 2021. doi: doi.org/10.1016/j.gpb.2021.07.005

[65] Wendi Bacon, Jonathan Manning, Generating a single cell matrix using Alevin (Galaxy Training Materials). https://gxy.io/GTN:T00245

[66] Wendi Bacon, Jonathan Manning, Combining single cell datasets after pre-processing (Galaxy Training Materials). https://gxy.io/GTN:T00246

[67] Julia Jakiela, Wendi Bacon, Generating a single cell matrix using Alevin and combining datasets (bash + R) (Galaxy Training Materials). https://gxy.io/GTN:T00378

[68] Wendi Bacon, Filter, plot and explore single-cell RNA-seq data with Scanpy (Galaxy Training Materials). https://gxy.io/GTN:T00247

[69] Morgan Howells, Wendi Bacon, Filter, plot and explore single-cell RNA-seq data with Scanpy (Python) (Galaxy Training Materials). https://gxy.io/GTN:T00358

[70] Camila Goclowski, Pablo Moreno, Filter, plot, and explore single cell RNA-seq data with Seurat (Galaxy Training Materials). https://gxy.io/GTN:T00438

[71] Camila Goclowski, Filter, plot, and explore single cell RNA-seq data with Seurat (R) (Galaxy Training Materials). https://gxy.io/GTN:T00366

[72] Marisa Loach, Wendi Bacon, Julia Jakiela, Mehmet Tekman, Inferring single cell trajectories with Scanpy (Galaxy Training Materials). https://gxy.io/GTN:T00379

[73] Wendi Bacon, Julia Jakiela, Mehmet Tekman, Inferring single cell trajectories with Scanpy (Python) (Galaxy Training Materials). https://gxy.io/GTN:T00244

[74] Julia Jakiela, Inferring single cell trajectories with Monocle3 (Galaxy Training Materials). https://gxy.io/GTN:T00249

[75] Julia Jakiela, Inferring single cell trajectories with Monocle3 (R) (Galaxy Training Materials). https://gxy.io/GTN:T00336

[76] Patro R, Duggal G, Love M, et al. Salmon provides fast and bias-aware quantification of transcript expression. Nature Methods. 2017. doi: doi.org/10.1038/nmeth.4197

[77] Srivastava A, Malik L, Smith T, et al. Alevin efficiently estimates accurate gene abundances from dscRNA-seq data. Genome Biology. 2019. doi: doi.org/10.1186/s13059-019-1670-y

[78] Lun ATL, Riesenfeld S, Andrews T, et al. EmptyDrops: distinguishing cells from empty droplets in droplet-based single-cell RNA sequencing data. Genome Biology. 2019. doi:10.1186/s13059-019-1662-y

[79] Griffiths JA, Richard AC, Bach K, et al. Detection and removal of barcode swapping in single-cell RNA-seq data." Nature Communications. 2018. doi:10.1038/s41467-018-05083-x

[80] atlas-gene-annotation-manipulation. Github. https://github.com/ebi-gene-expression-group/atlas-gene-annotation-manipulation

[81] Love MI, Soneson C, Hickey PF, et al. Tximeta: Reference sequence checksums for provenance identification in RNA-seq. PLOS Computational Biology. 2020. doi:10.1371/journal.pcbi.1007664

[82] Durinck S, Moreau Y, Kasprzyk A, et al. BioMart and Bioconductor: a powerful link between biological databases and microarray data analysis. Bioinformatics. 2005. 21, 3439–3440. https://doi.org/10.1093/bioinformatics/bti525

[83] Durinck S, Spellman P, Birney E, et al. Mapping identifiers for the integration of genomic datasets with the R/Bioconductor package biomaRt. Nature Protocols. 2009. 4, 1184–1191. https://doi.org/10.1038/nprot.2009.97

[84] Csárdi G, Nepusz T, Traag V, et al. (2024). igraph: Network Analysis and Visualization in R. doi: 10.32614/CRAN.package.igraph.

[85] Blondel VD, Guillaume J, Lambiotte R, et al. Fast unfolding of communities in large networks. Journal of Statistical Mechanics: Theory and Experiment 2008. doi: 10.1088/1742-5468/2008/10/P10008

[86] McKinney, W., & others. (2010). Data structures for statistical computing in python. In Proceedings of the 9th Python in Science Conference (Vol. 445, pp. 51–56). http://conference.scipy.org.s3.amazonaws.com/proceedings/scipy2010/pdfs/mckinney.pdf

[87] Martin Maechler. Matrix: Sparse and Dense Matrix Classes and Methods. 2024. doi: 10.32614/CRAN.package.Matrix

[88] Wickham H, François R, Henry L, et al. (2023). dplyr: A Grammar of Data Manipulation. https://dplyr.tidyverse.org.

[89] Razavi K, Luthra M, Koldehofe B, et al. FA2: Fast, Accurate Autoscaling for Serving Deep Learning Inference with SLA Guarantees. 2022 IEEE 28th Real-Time and Embedded Technology and Applications Symposium (RTAS), Milano, Italy, 2022, pp. 146-159, doi: 10.1109/RTAS54340.2022.00020

[90] Harris, C.R., Millman, K.J., van der Walt, S.J. et al. Array programming with NumPy. Nature 585, 357–362 (2020). DOI: 10.1038/s41586-020-2649-2

[81] J. D. Hunter, "Matplotlib: A 2D Graphics Environment", Computing in Science & Engineering, vol. 9, no. 3, pp. 90-95, 2007. doi: 10.1109/MCSE.2007.55

[92] Virshup et al., (2024). anndata: Access and store annotated data matrices. Journal of Open Source Software, 9(101), 4371, https://doi.org/10.21105/joss.04371

[93] Garnier, Simon, Ross, Noam, Rudis, Robert, Camargo, Pedro A, Sciaini, Marco, Scherer, Cédric (2023). viridis(Lite) - Colorblind-Friendly Color Maps for R. doi:10.5281/zenodo.4678327, viridisLite package version 0.4.2, https://sjmgarnier.github.io/viridis/.

[94] Bache S, Wickham H (2022). magrittr: A Forward-Pipe Operator for R. https://magrittr.tidyverse.org, https://github.com/tidyverse/magrittr.

[95] Eddelbuettel D, Francois R, Allaire J, Ushey K, Kou Q, Russell N, Ucar I, Bates D, Chambers J (2024). Rcpp: Seamless R and C++ Integration. R package version 1.0.12, doi: 10.32614/CRAN.package.Rcpp.

[96] Single Cell subpage of Galaxy Training Network (GTN). 2024. https://training.galaxyproject.org/training-material/topics/single-cell. Accessed 20 Nov 2024

[97] Bacon, W. A. (2021). Pre-processing scRNA-seq data using Alevin in Galaxy [Data set]. Zenodo. https://doi.org/10.5281/zenodo.4574153

[98] Jakiela, J. (2024). Combining datasets after Alevin pre-processing - Galaxy Training Material [Data set]. Zenodo. https://doi.org/10.5281/zenodo.10852529

[99] Bacon, W. A. (2022). AnnData object for case study tutorials [Data set]. Zenodo. https://doi.org/10.5281/zenodo.7053673

[100] Bacon, W. A. (2021). Trajectories_Jupyter_Tutorial [Data set]. Zenodo. https://doi.org/10.5281/zenodo.7075718

[101] Jakiela, J. (2023). CDS input for Monocle3 tutorial - Galaxy Training Material [Data set]. Zenodo. https://doi.org/10.5281/zenodo.10397366

[102] Jakiela, J. (2022). Trajectory Analysis: Monocle3 in RStudio - Galaxy Training Material [Data set]. Zenodo. https://doi.org/10.5281/zenodo.7455590

[103] Galaxy Europe server. https://usegalaxy.eu . Accessed 20 Nov 2024

[104] Galaxy US server. https://usegalaxy.org . Accessed 20 Nov 2024

[105] Galaxy Australia server. https://usegalaxy.org.au . Accessed 20 Nov 2024

[106] Galaxy ToolShed. https://toolshed.g2.bx.psu.edu . Accessed 20 Nov 2024

Supplementary Data

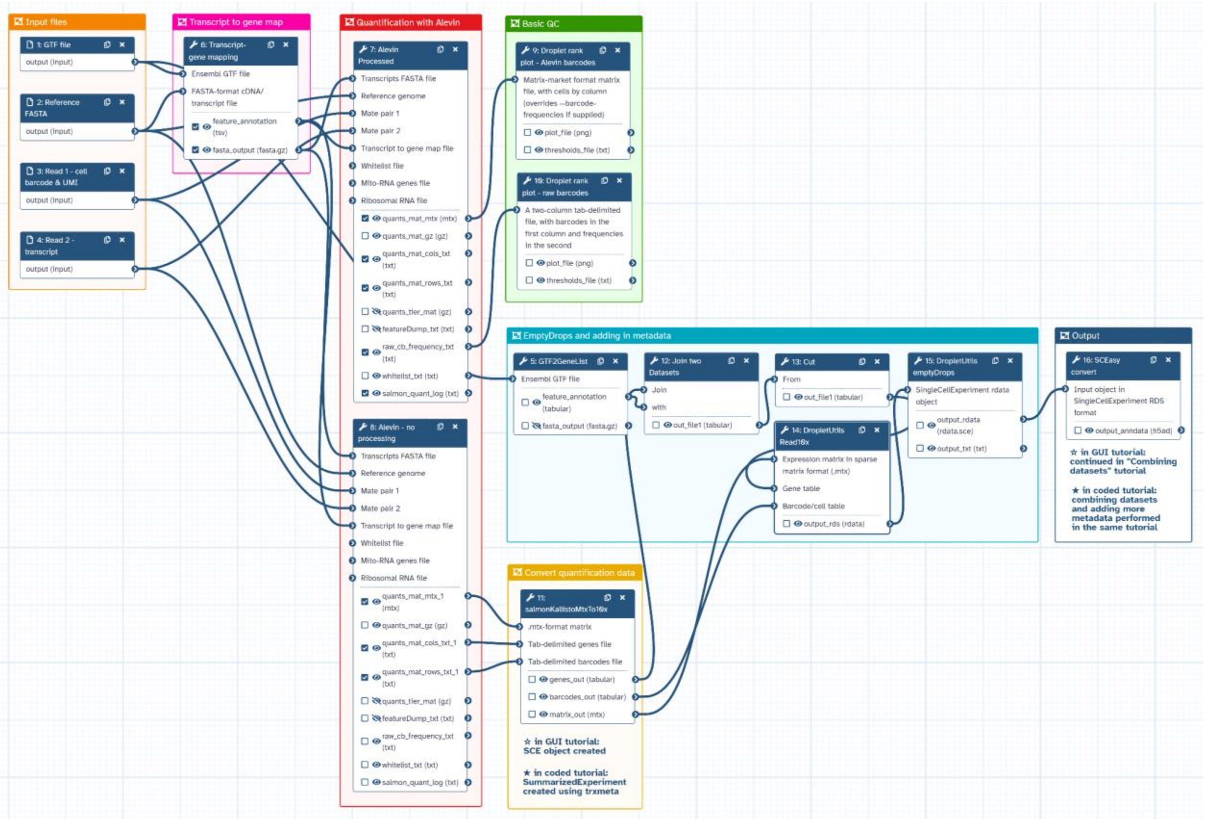

Fig S1. Galaxy workflow for tutorial “Generating a single-cell matrix using Alevin”. The tools used for the analysis are shown, together with their outputs and connectivities, as well as high-level descriptors of performed steps. Solid stars denote steps specific to the PE tutorial while unfilled stars represent BB specific ones.

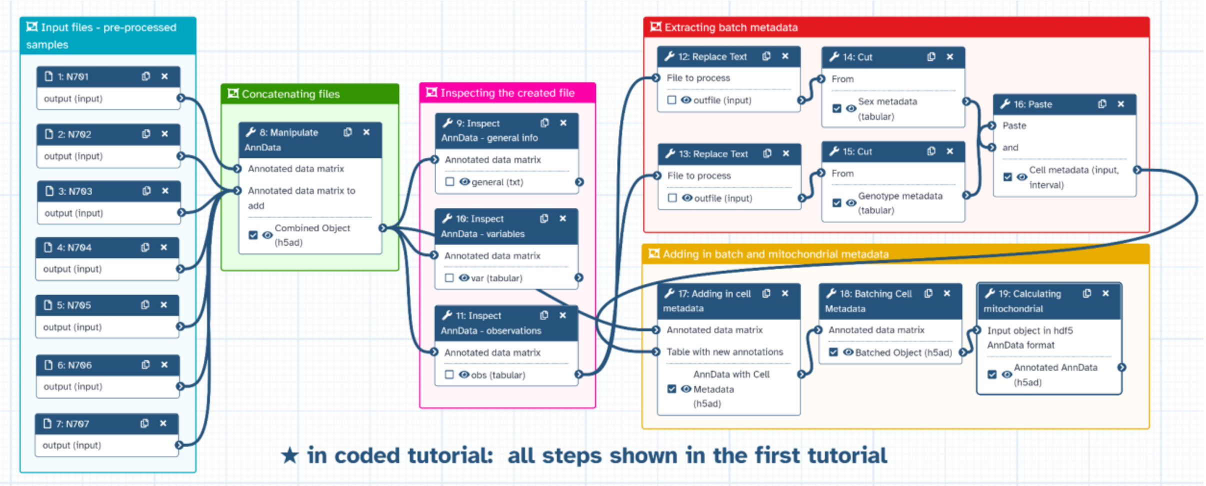

Fig S2. Galaxy workflow for tutorial “Combining single-cell datasets after pre-processing”. All tools used for the analysis are shown, as well as their connectivities and high-level descriptors of performed steps. Solid star denotes steps specific to the PE tutorial.

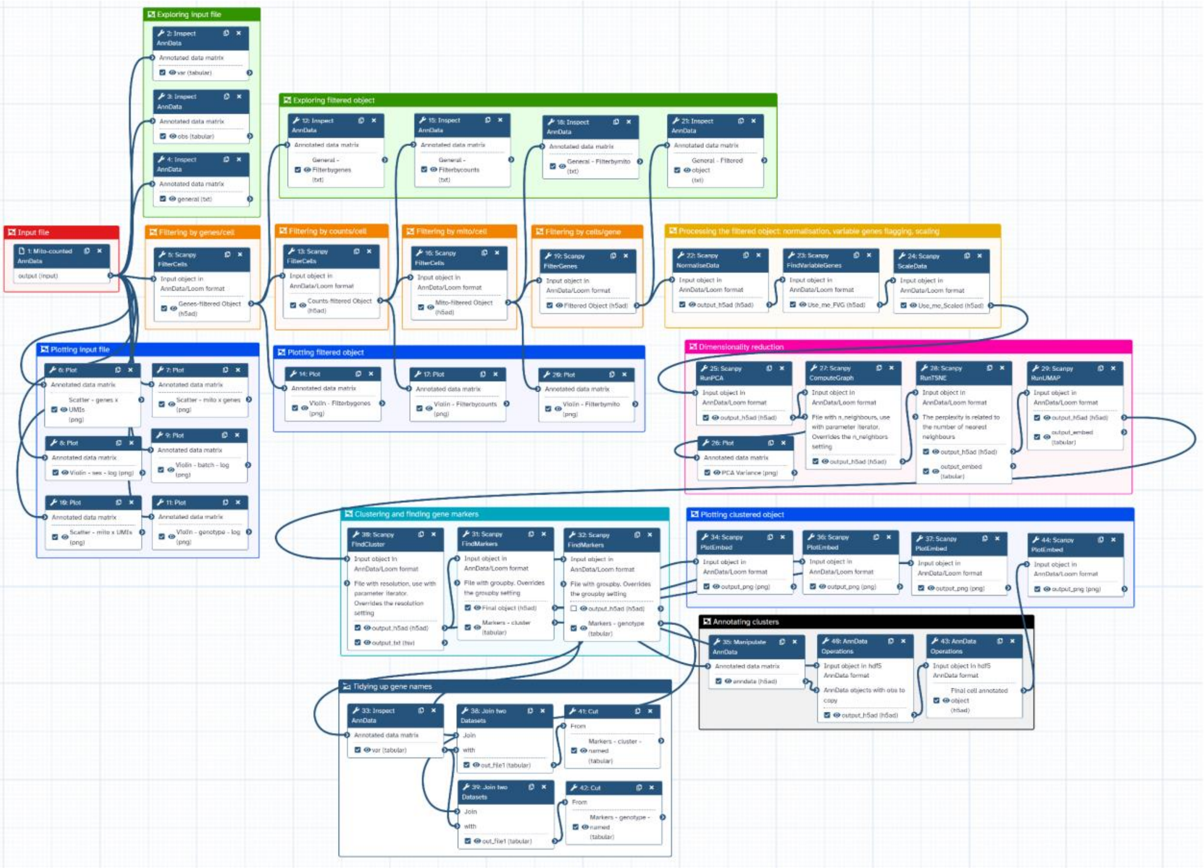

Fig S3. Galaxy workflow for tutorial "Filter, plot and explore single-cell RNA-seq data (Scanpy)". All tools used for the analysis are shown, as well as their connectivities and high-level descriptors of performed steps.

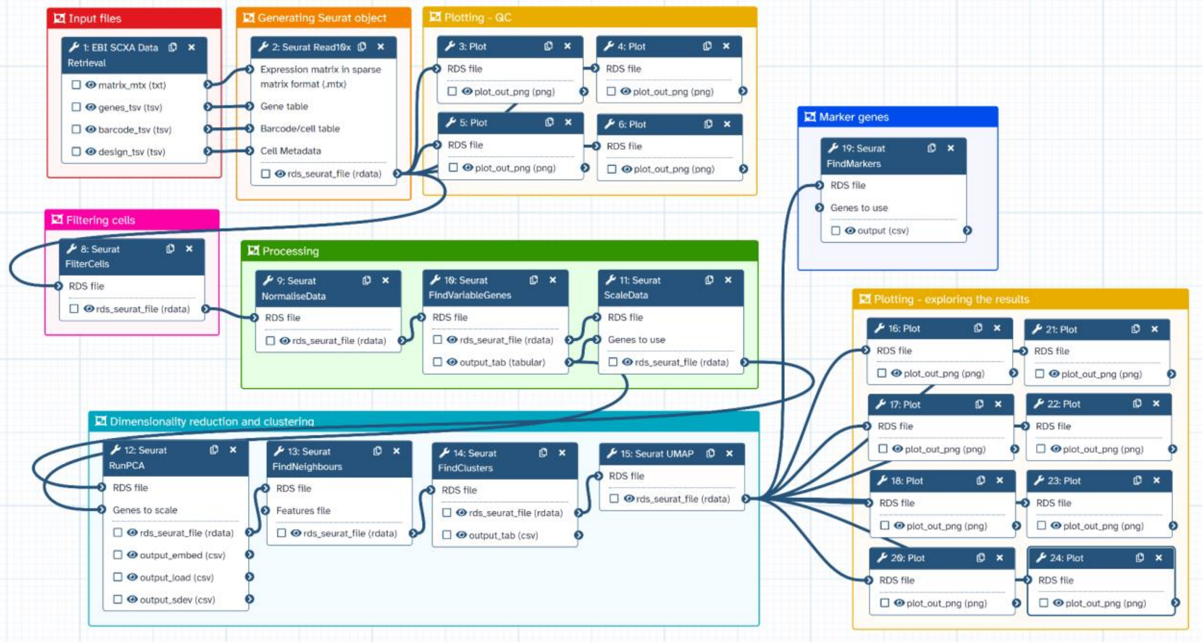

Fig S4. Galaxy workflow for tutorial "Filter, plot and explore single-cell RNA-seq data (Seurat)". All tools used for the analysis are shown, as well as their connectivities and high-level descriptors of performed steps.

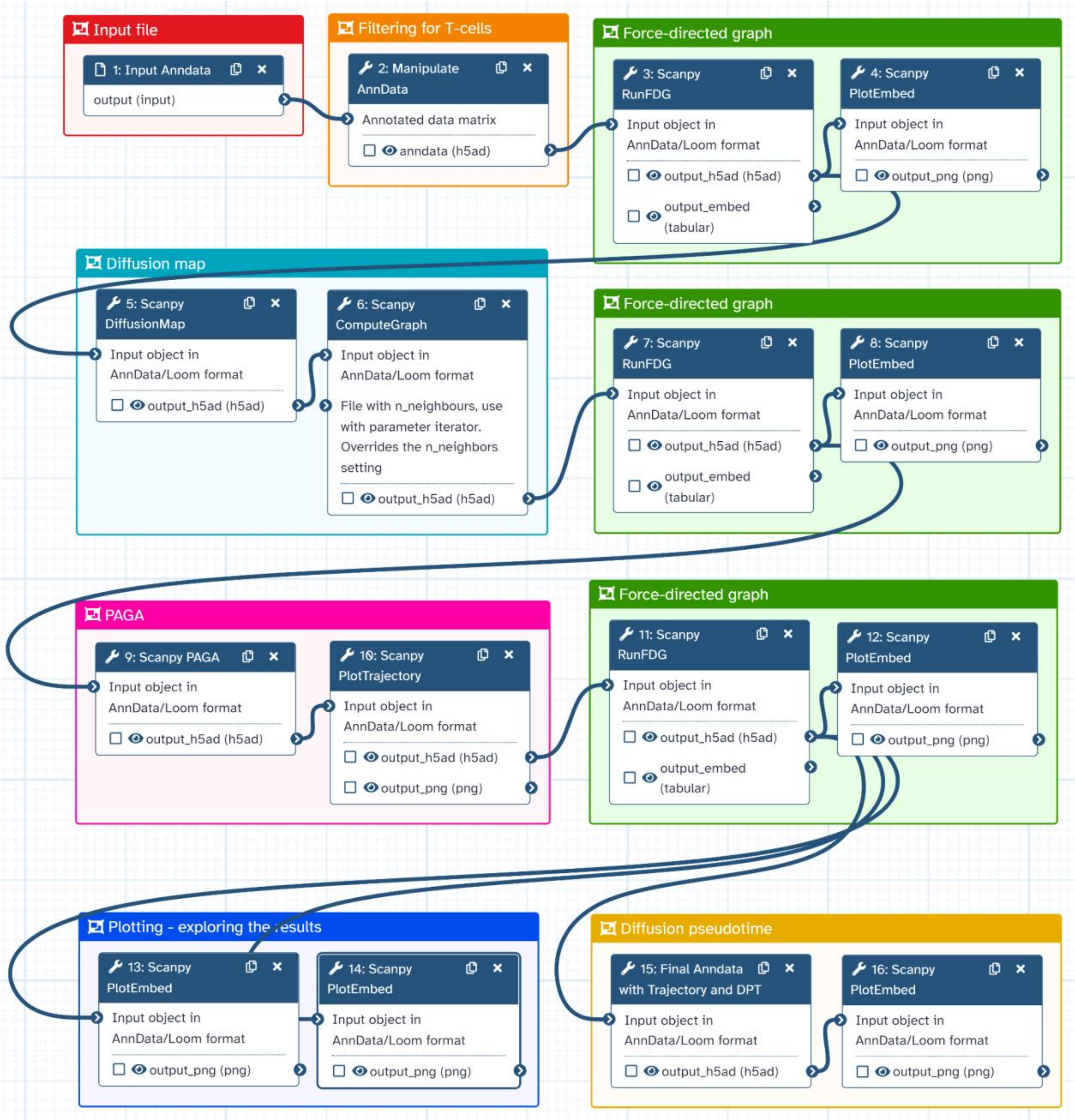

Fig S5. Galaxy workflow for tutorial “Inferring single-cell trajectories (Scanpy)”. All tools used for the analysis are shown, as well as their connectivities and high-level descriptors of performed steps.

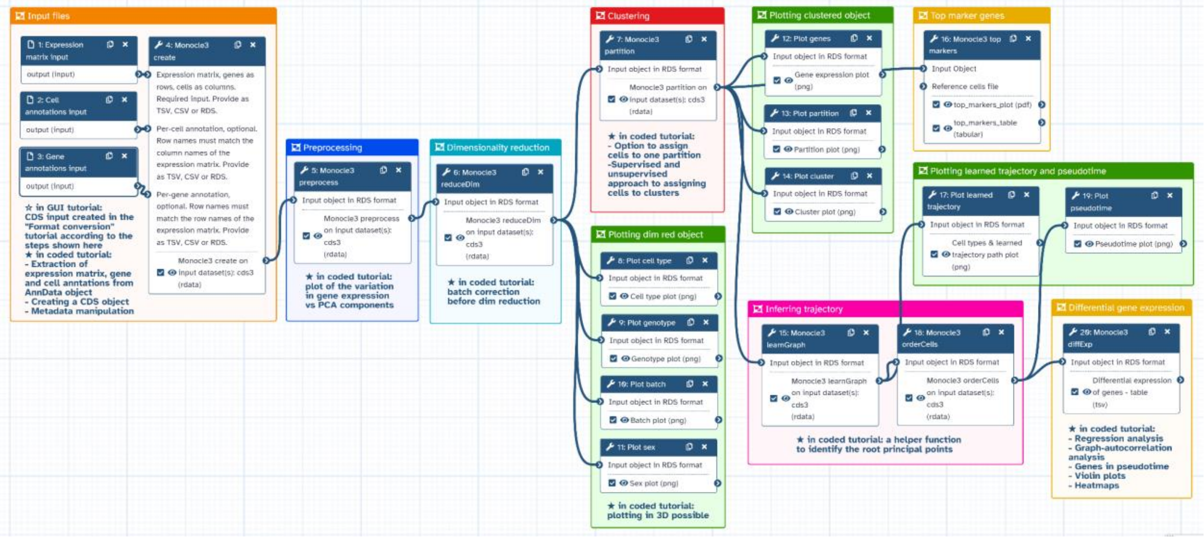

Fig S6. Galaxy workflow for tutorial “Inferring single-cell trajectories (Monocle3)”. All tools used for the analysis are shown, as well as their connectivities and high-level descriptors of performed steps.

Click here to access/download;Figure;Fig1_tools_fig_updated.png

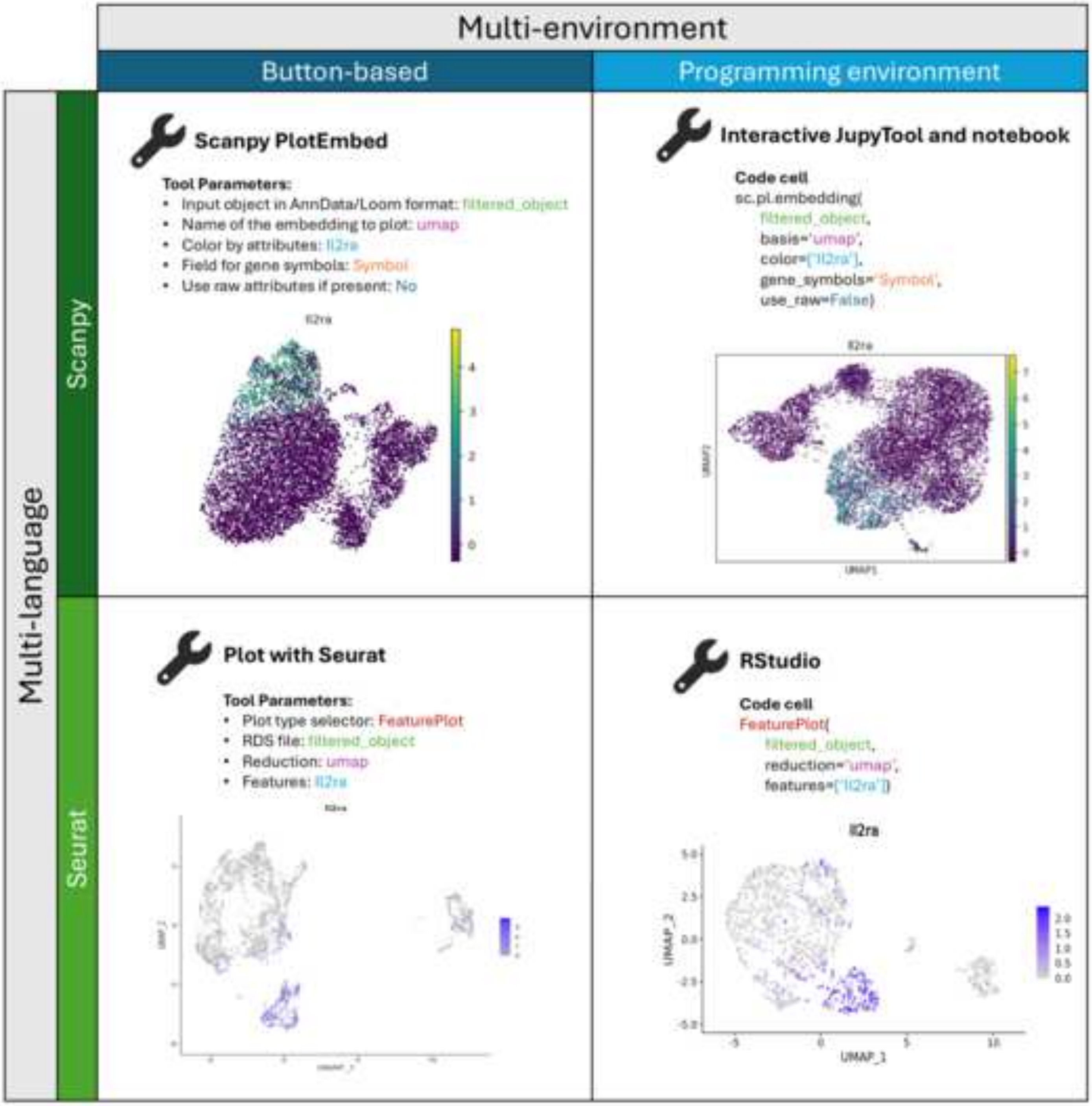

Click here to access/download;Figure;Fig2_user_journey.jpg

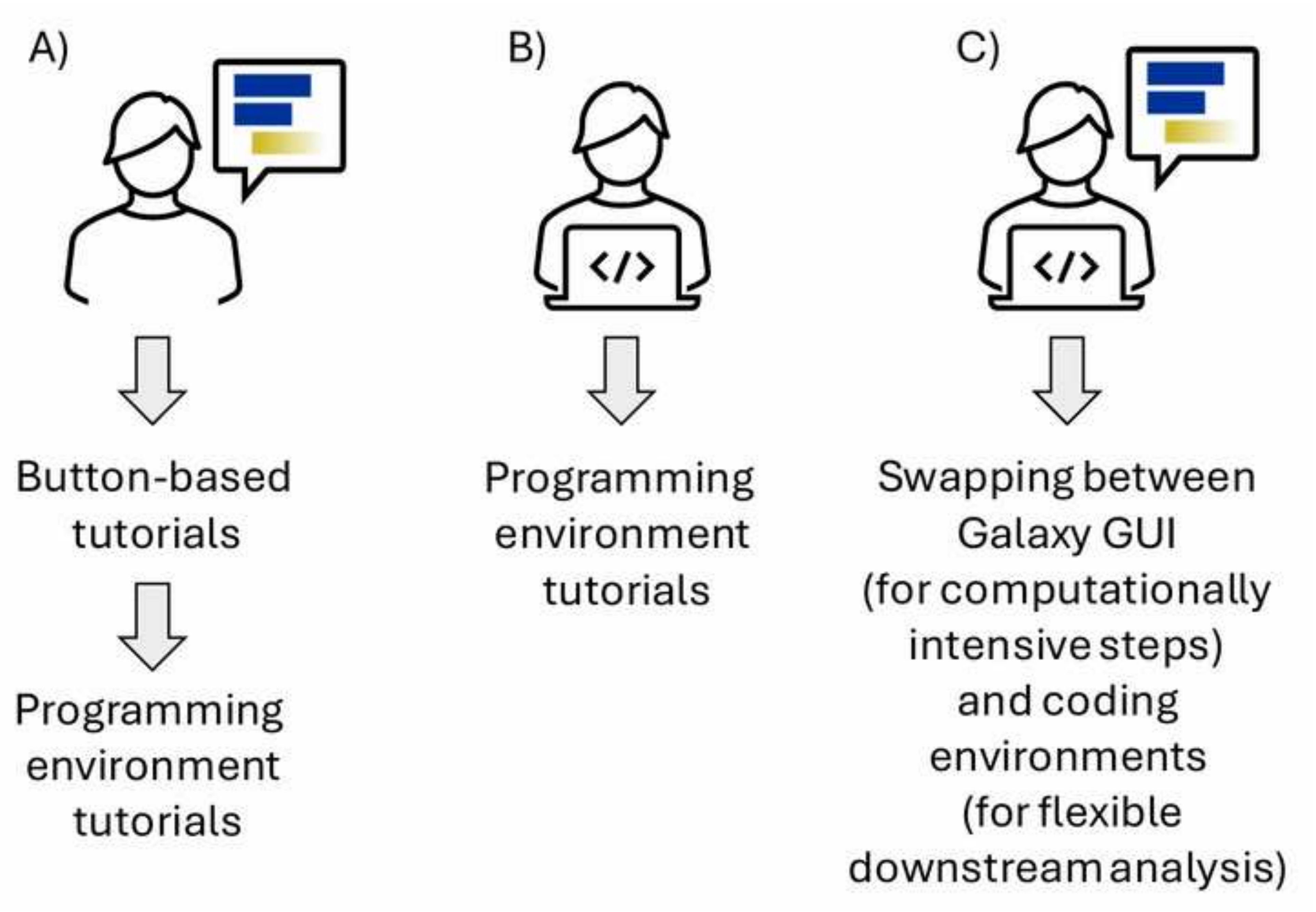

Click here to access/download;Figure;Fig3_connectivity2.jpg

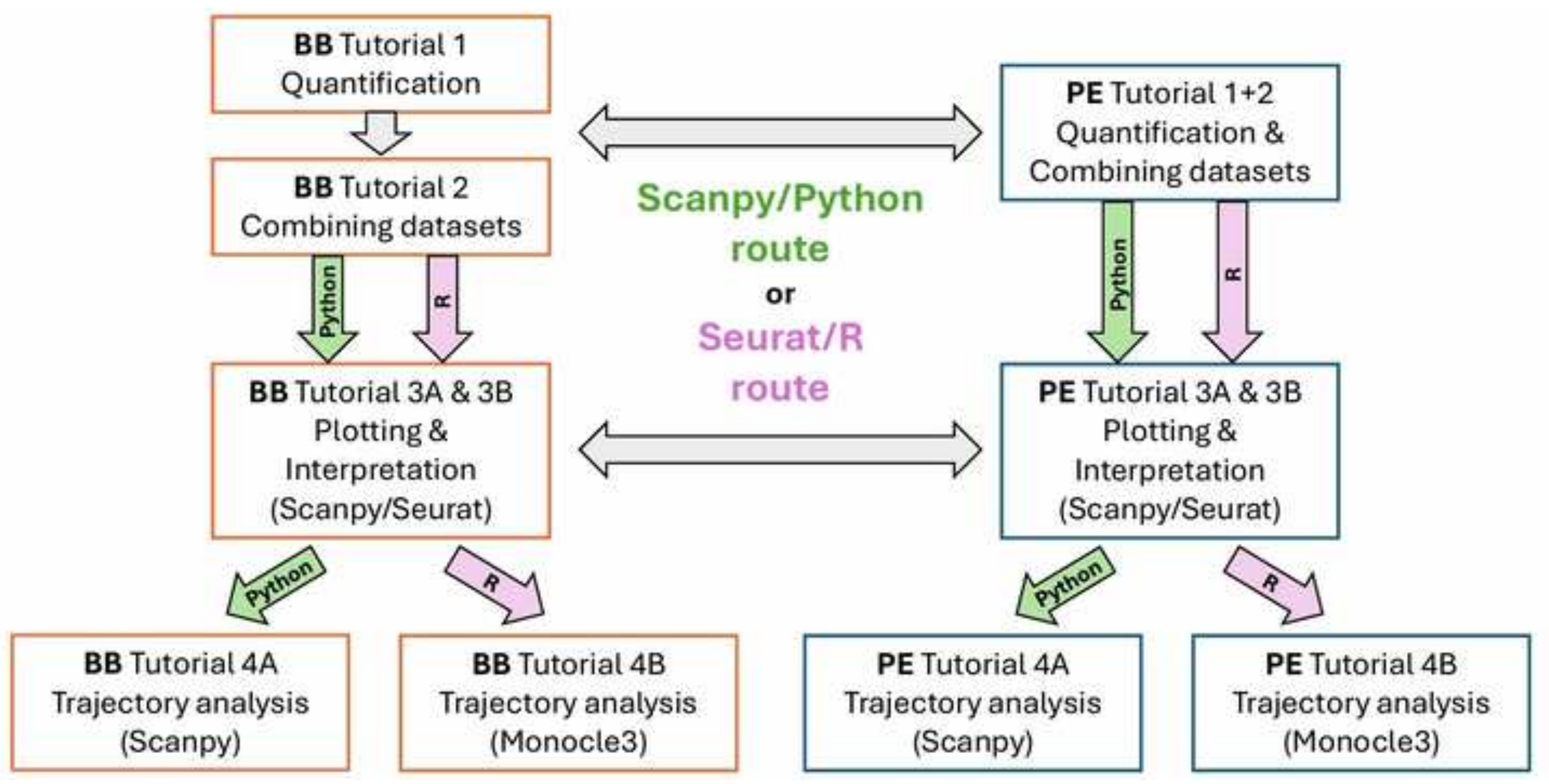

Click here to access/download;Figure;Fig4_Alevin_combining.png

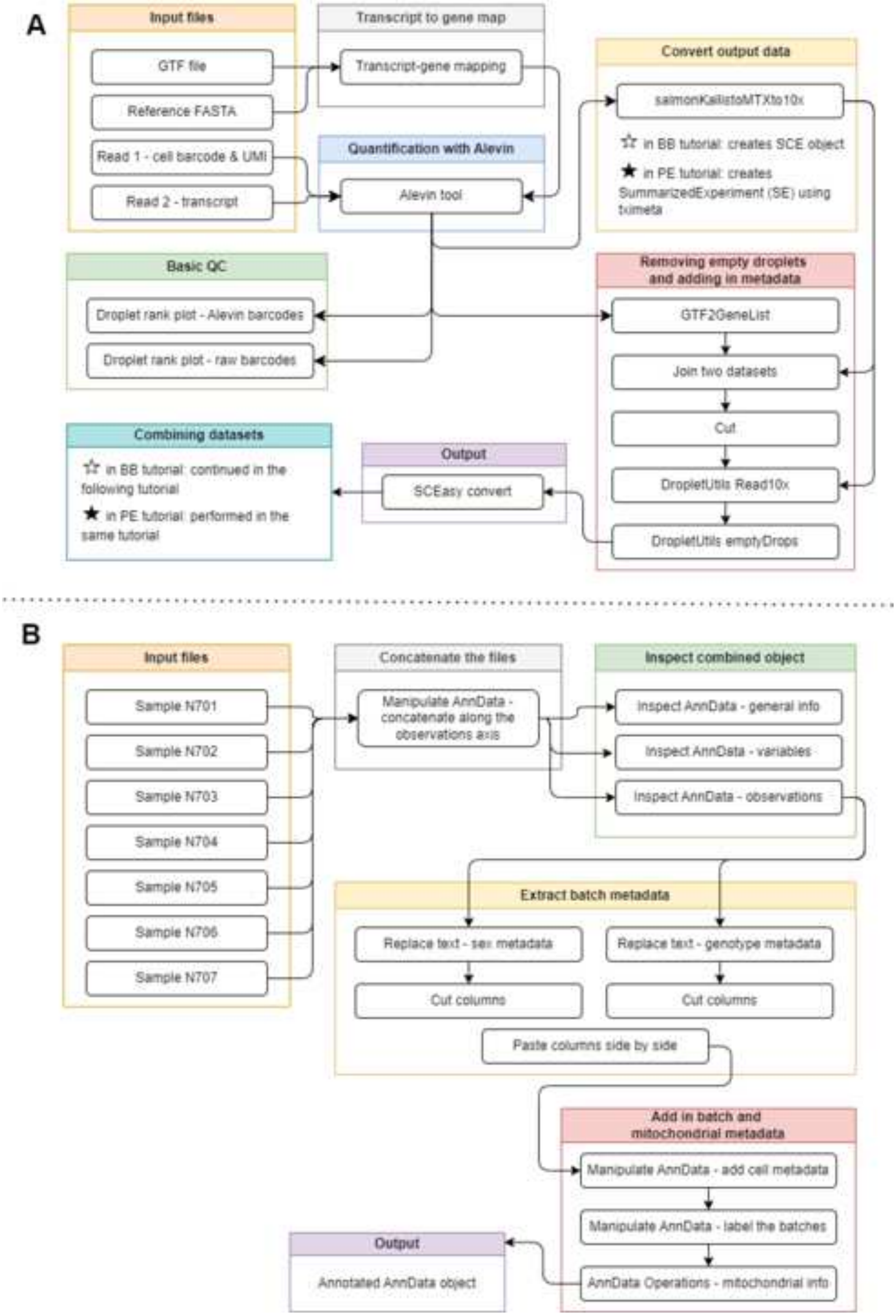

Click here to access/download;Figure;Fig5_FPE_scanpy.png

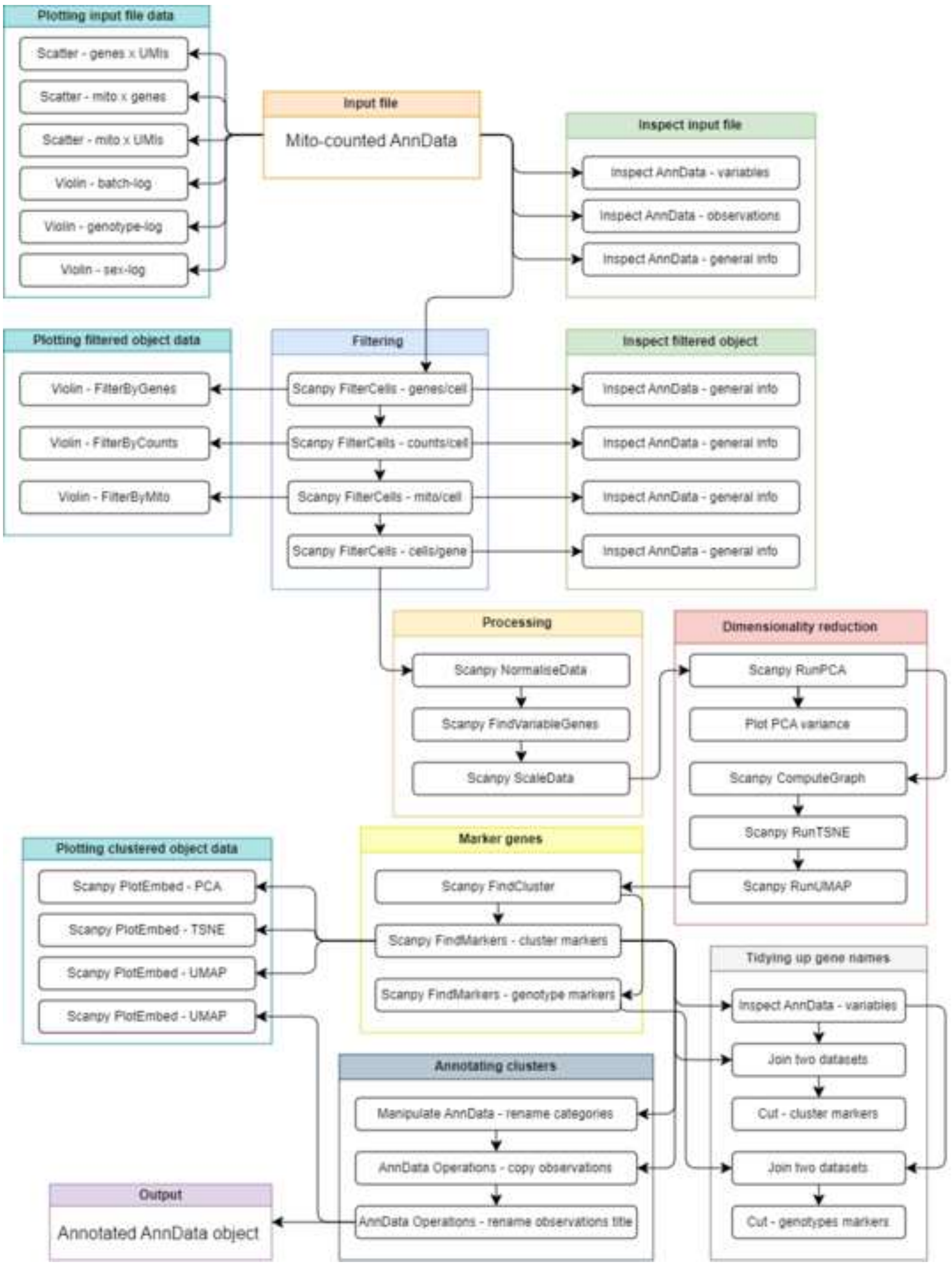

Click here to access/download;Figure;Fig6_FPE_Seurat_diagram.png

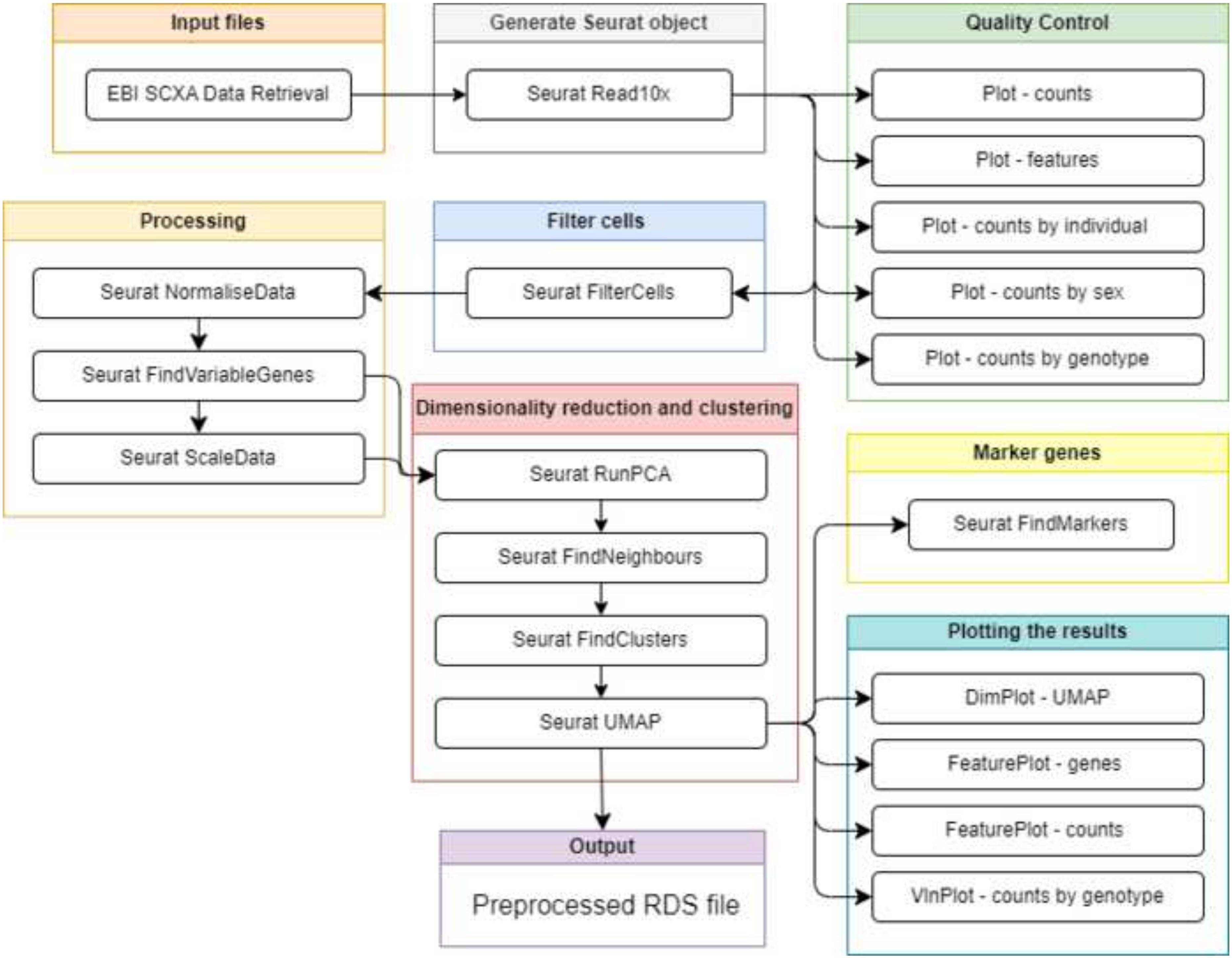

Click here to access/download;Figure;Fig7_Scanpy_trajectories.png

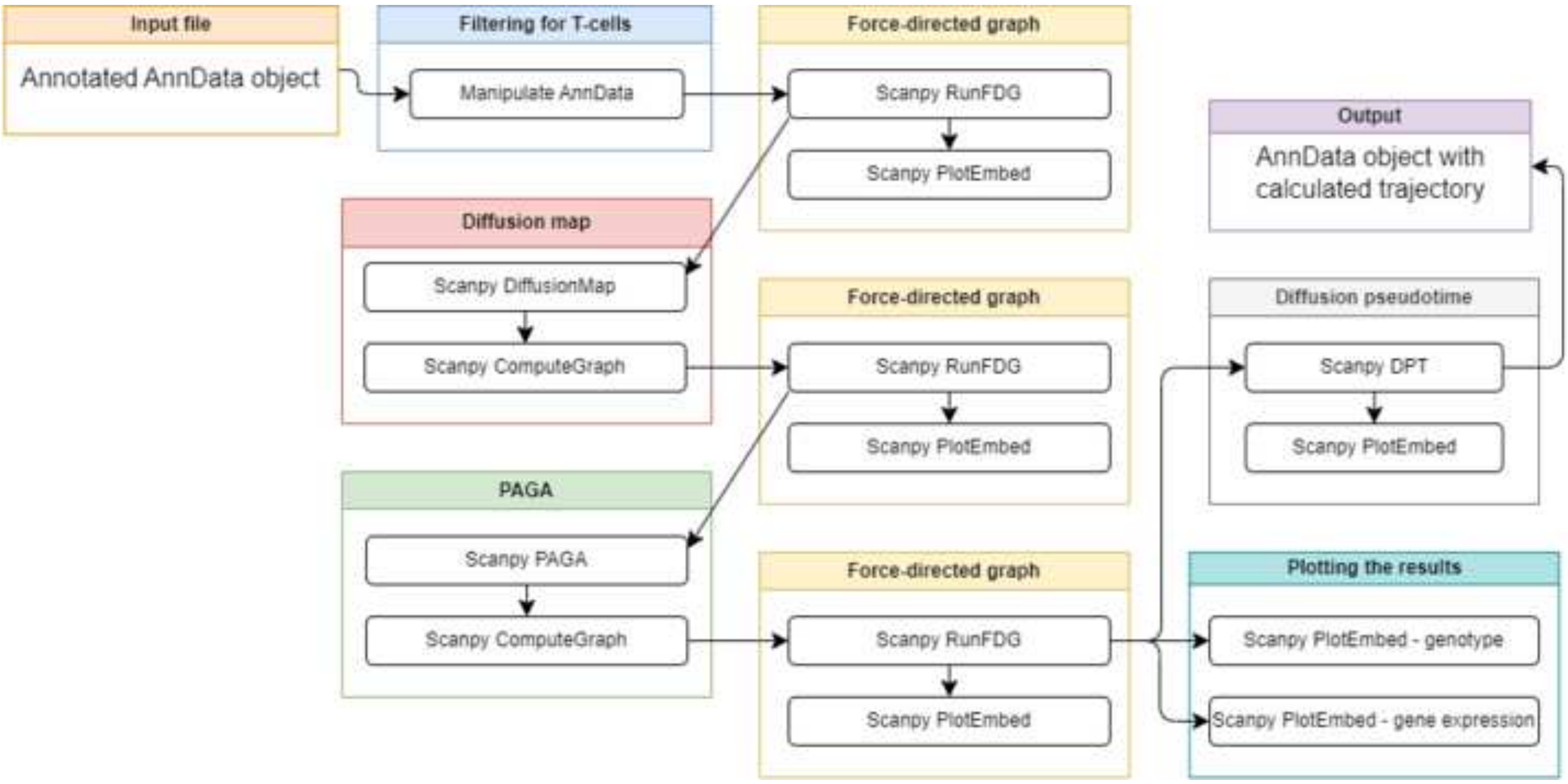

Click here to access/download;Figure;Fig8_Monocle3.png

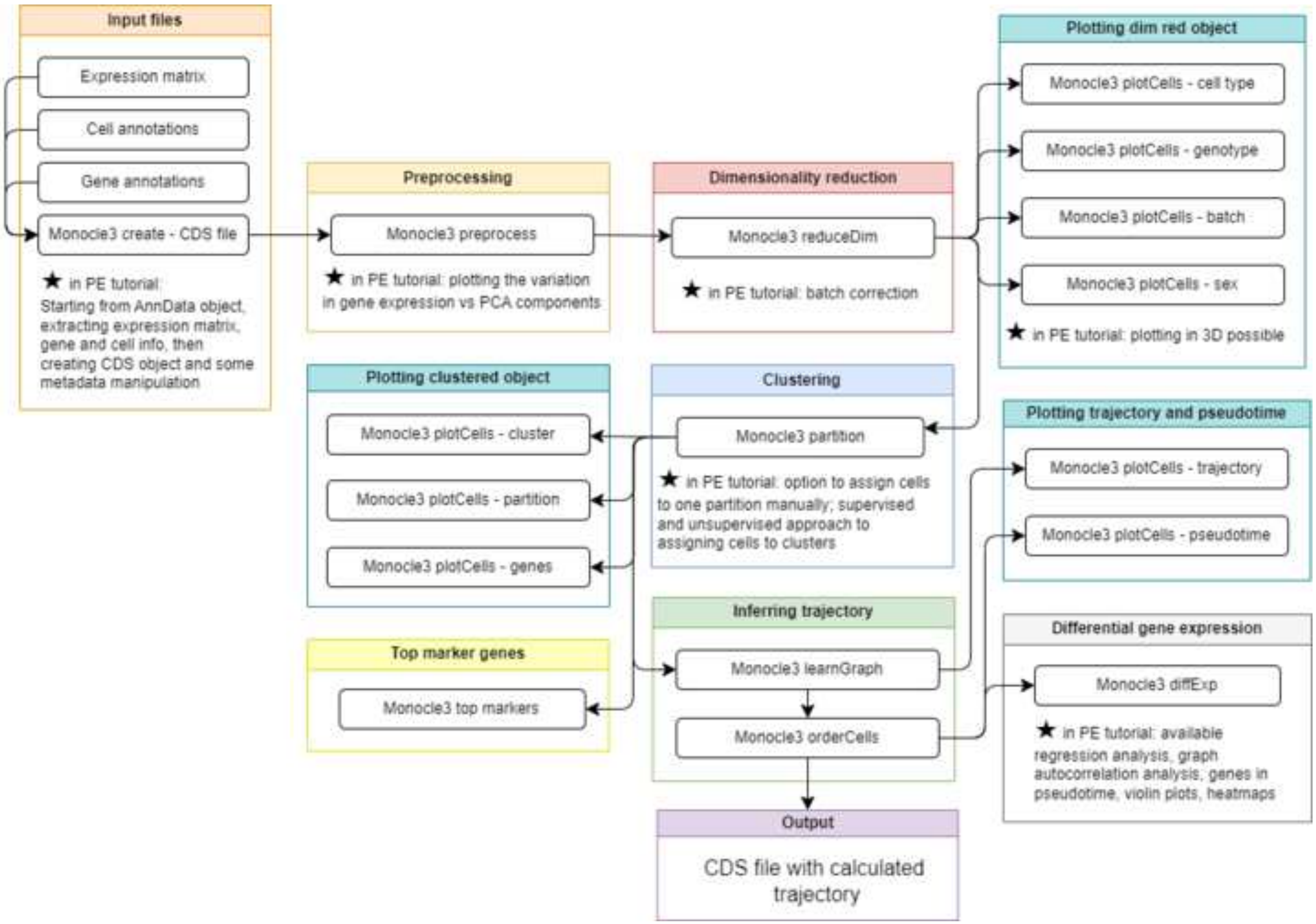

Click here to access/download;Figure;Fig9_outputs_numbers_2.png

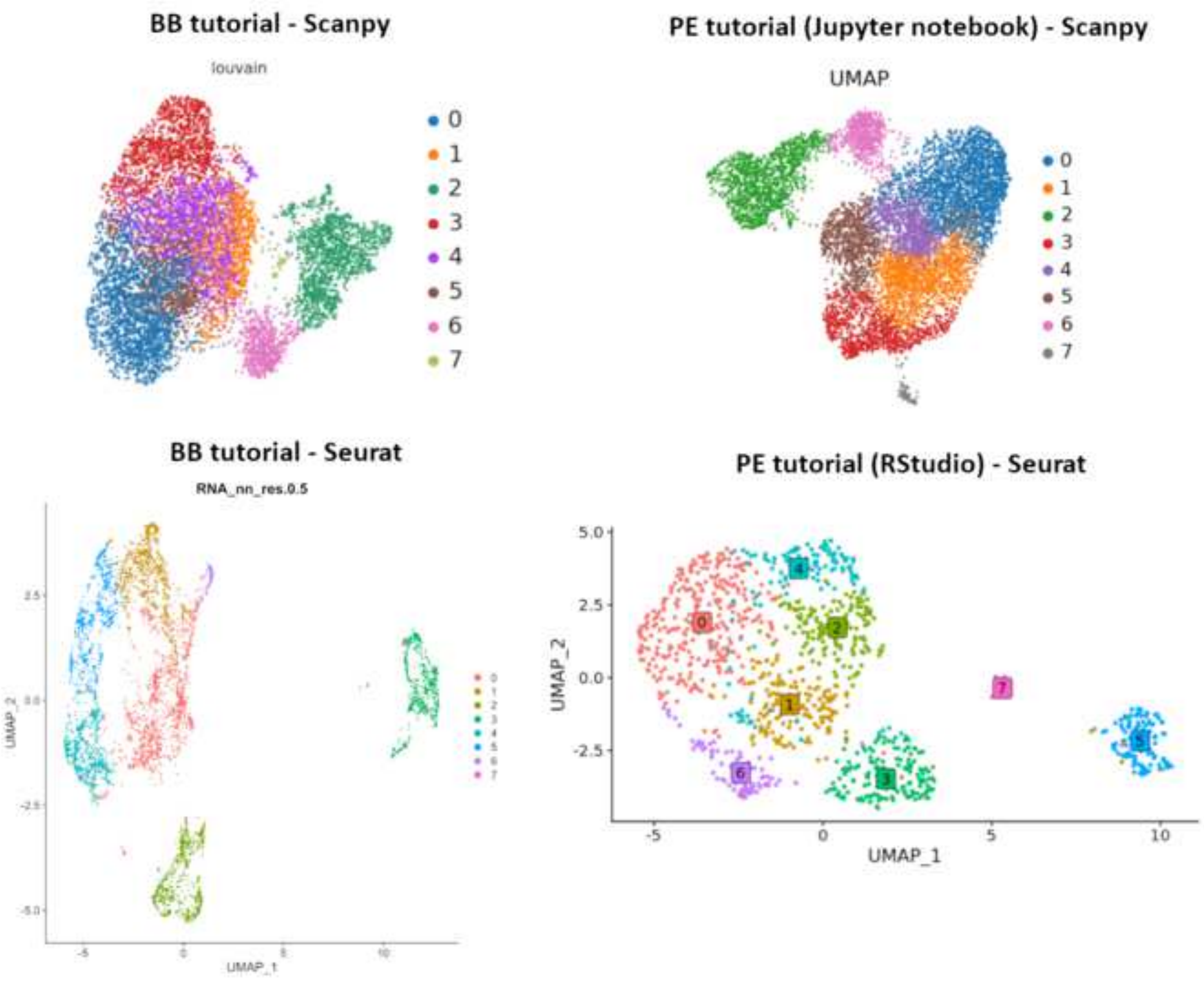

Click here to access/download;Figure;Fig10_decision_time.jpg

Details: Working in a group? Decision-time!

If you are working in a group, you can now divide up a decision here with one *control* and the rest varied numbers so that you can compare results throughout the tutorials.

- Control
  - **log1p_n_genes_by_counts** > `5.7`
  - **log1p_total_counts** > `6.3`
  - **pct_counts_mito** < `4.5%`
- Everyone else: Choose your own thresholds and compare results!

Click here to access/download;Figure;Fig11_header_feature.jpg

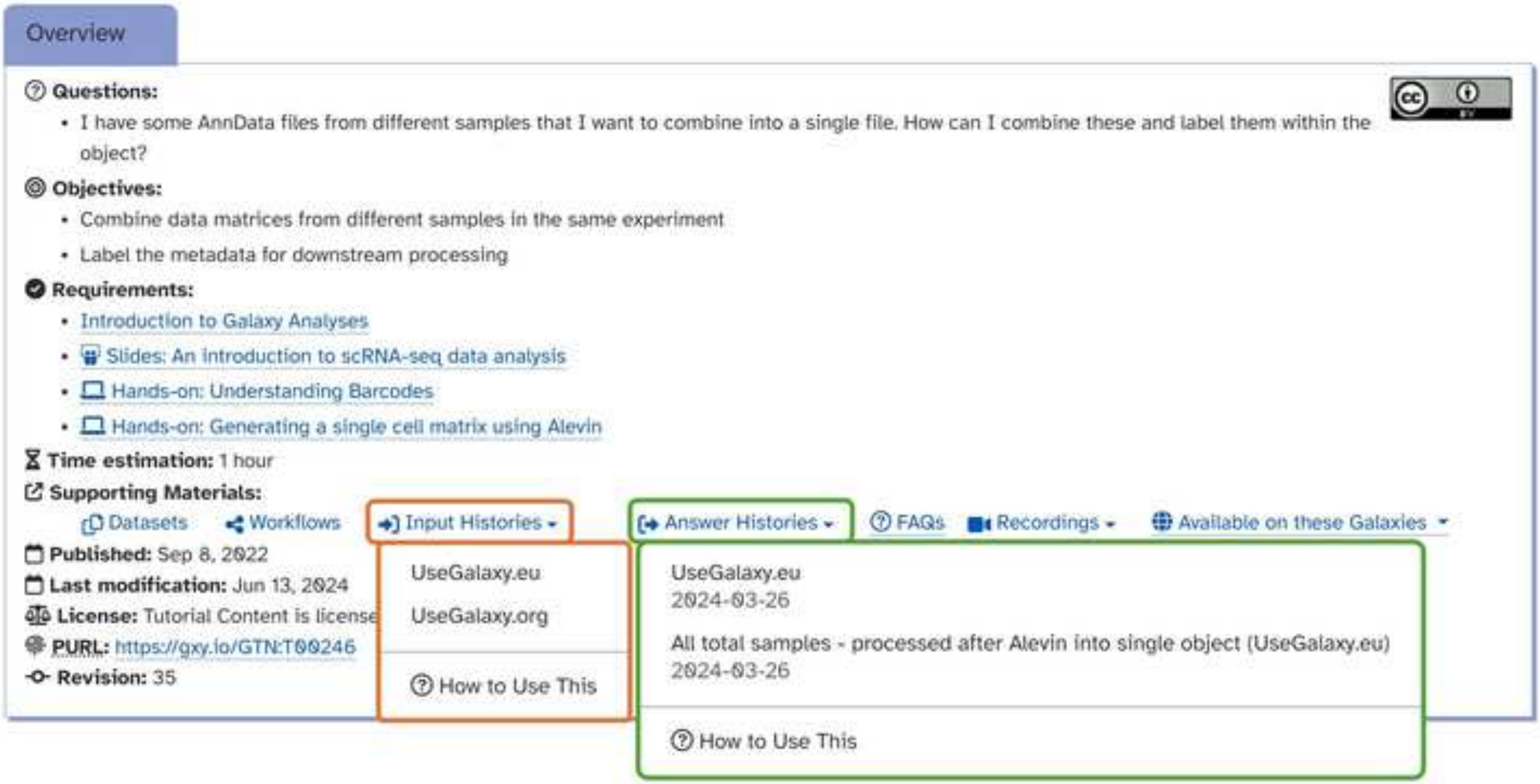

Click here to access/download;Figure;Fig12.jpg

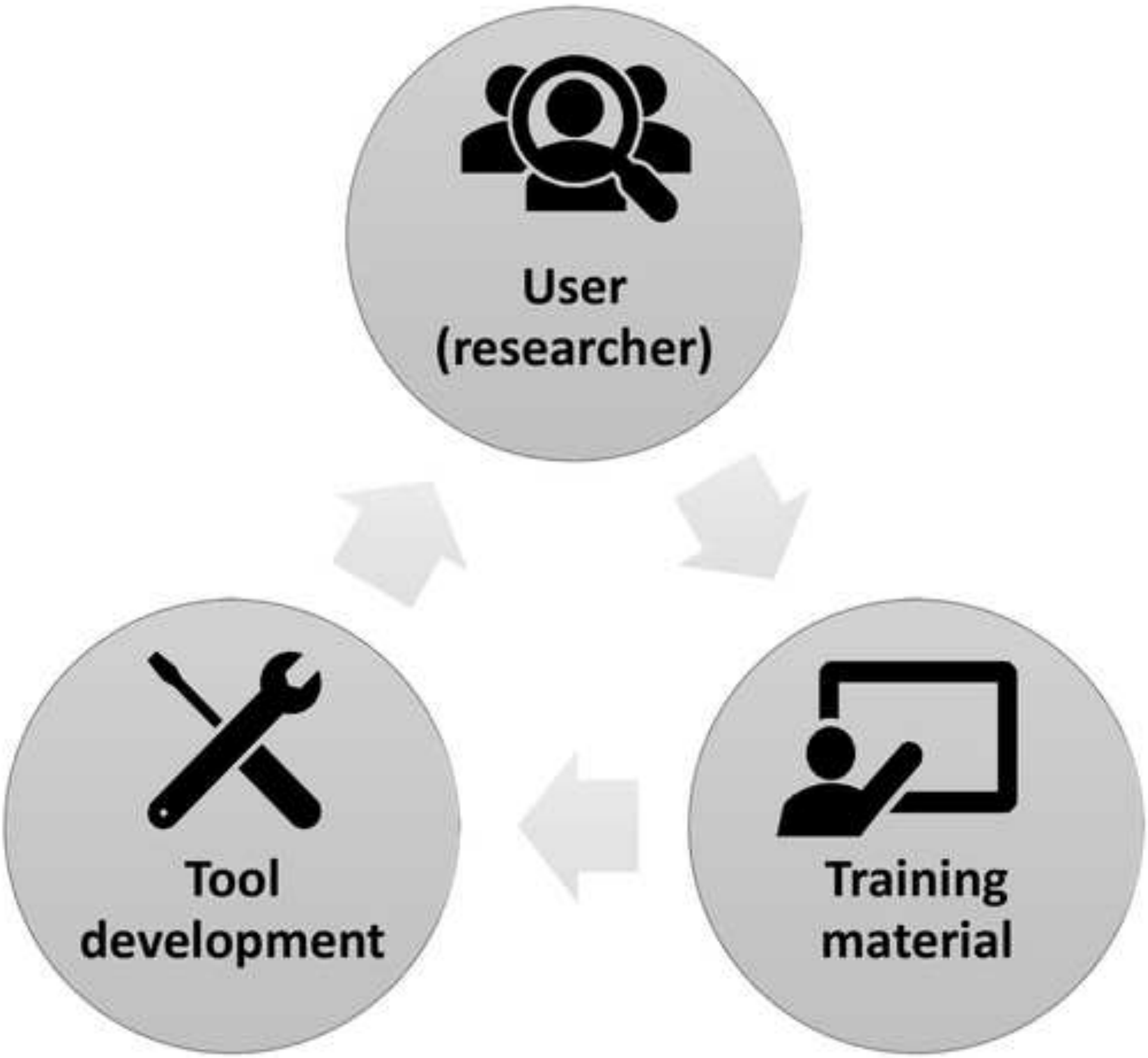

Click here to access/download
Supplementary Material
FigS1_Alevin_workflow-min.png

Click here to access/download
Supplementary Material
FigS2_combining_datasets-min.png

Click here to access/download
Supplementary Material
FigS3_FPE-min.png

Click here to access/download
Supplementary Material
FigS4_seurat_FPE-min.png

Click here to access/download
Supplementary Material
FigS5_trajectories_scanpy-min.png

Click here to access/download
Supplementary Material
FigS6_monocle-min.png

Click here to access/download;Personal Cover;Resubmission Cover Letter.docx

The Open University

Walton Hall
Milton Keynes
United Kingdom
MK7 6AA

open.ac.uk

Resubmission & Reviewer Response

28 Oct 2024

Dear *Gigascience* Editorial Board,

We are excited to re-submit an original technical note entitled “Galaxy as a Gateway to Bioinformatics: Multi-Interface Galaxy Hands-on Training Suite (MIGHTS) for scRNA-seq”

We first wish to thank the reviewers for their time and thoughtful comments on, and line edits of, our manuscript. Below is a summary of our edits in response to reviewers:

Reviewer 1:

1. “While the manuscript lays emphasis on unstandardized and iterative analysis, the authors could **draw a strong rationale on lack of reinstantiation methods which is certainly the need**. The tools like Scanpy/Seurat and other ecosystem tools, there is a latency and they work differently.”

    - In the discussion, reinstantiation has been emphasized, in connection to reproducibility, as an end goal accomplishment of the MIGHTS tutorial suite.

2. “Training-the-trainer is a very important entity as next generation sequencing (NGS) tools advances. A word or two on that would be a nice addition.”

    - In the discussion, added “As sequencing strategies and tools advance, it is important that the field of bioinformatics “trains the trainer” in response to continued growth”.

3. “While programming based and button based entities are well taken, there could be a ‘programming a button’ section if not swapping between them.

The Open University is incorporated by Royal Charter (RC 000391), an exempt charity in England & Wales, and a charity registered in Scotland (SC 038302). The Open University is authorised and regulated by the Financial Conduct Authority in relation to its secondary activity of credit broking.

This will allow naive bioinformaticists to embrace programming. The authors may want to mention this."

   - In the discussion, added a nod to the existing "How to wrap a galaxy tool" section of GTN Training Material: "If users wish to embrace programming such that they are looking to wrap their own tools and create training material based on them, resources and opportunities to do so exist on GTN pages dedicated to development in Galaxy [https://training.galaxyproject.org/training-material/topics/dev/] and contributing to the Galaxy Training Material [https://training.galaxyproject.org/training-material/topics/contributing/]"

4. "There could be an alternative annotation tool, viz. Annotationhub instead of Biosmart as well."

   - We thank the reviewers for this comment, however we cannot include every available tool in Galaxy, so unfortunately this is beyond the scope of the project. The authors agree that expanding annotation tool availability and programming environments would be beneficial. The Galaxy Training Network is committed to continued enhancement of tools to meet evolving user needs.

5. "Likewise vscode could be more inviting for some instead of jupyter IDE. The end user may be given a note in README file"
   - Because this is training material, rather than traditional coding documentation, there is no README file associated with the training. VSCode is not traditionally used for training, as the Jupyter IDE includes both the coding environment and the space to run it. For this reason, we have not mentioned text editing software. However, after completing the MIGHTS tutorials, the users should feel comfortable enough in the programming environment to work in another IDE if they find it more inviting.

Reviewer 2:

We have addressed each of the following grammatical/spelling errors:

6. As access to computationally driven domains of biology continue[s] to grow
7. blending skills across disciplines is not without challenge[s]

8. "MIGHTS demonstrates the use of many frequently used data types and packages for scRNA-seq analyses (Table 2), preparing users with research[-]relevant skills."
9. "Workflows for each tutorial topic are shown -below- in Figure 4."
    - "Workflow is demonstrated -below- in Figure 8" (remove the belows)

We have addressed the following figure and table corrections:

10. “Figure 1 is too small to read, and it would be interesting to compare the BB method and the PE method to get the same images.”
    - Figure 1 has been edited to convey key concepts/steps in the tutorial. Relevant information from the original figure was converted to text from a screenshot so as to more uniformly present the arguments. The figure caption has also been altered to more directly compare BB and PE methods.
11. “Table 1 and 2 are redundant, use only table 2.”
    - Tables 1 and 2 have been collapsed such that they do not present redundant information.
12. “Figure 4: Too small to see the stars.”.
    - Figure 4 has been replaced with a more legible workflow diagram describing only the necessary details of the tutorials’ workflows.
13. “Figure 4,5,6,7: Add the significance of colored boxes in the legend. (too small to read the box titles). Overall, these figures are hard to read and are difficult to link with the text. Maybe in the text about tutorials, mention which step corresponds to which box color, or move these figures to supplemental material with more detailed legends.”
    - Figures 4,5,6,7,8 have been replaced with more legible workflow diagrams rather than images of the extracted Galaxy workflows. The updated diagrams visualize the high-level information and the tools used in each step of the analysis. The coloring of the boxes has been unified, for example the input files are now all shown in orange, output files in purple, plotting in cyan, marker genes in bright yellow. Galaxy-generated workflow images were included in the Supplementary Data to provide exhaustive details, such as all the output files generated by each tool and their formats.
14. “Figure 9: what does each letter correspond to? It looks like it is showing the same information than figure 1.”
    - Figure 1 is intended to draw attention to the similarities in arguments across all four modes when plotting the expression of a gene of interest. Whereas Figure 9 demonstrates the consistency of clustering

results observed across all four methods. The letters in the Figure 9 were removed since the image itself is self-explanatory, without the need to include the four panels A-D.

15. "'MIGHTS demonstrates the use of many frequently used data types and packages for scRNA-seq analyses (Table 2), preparing users with research relevant skills.' : Discuss a bit more which skills are deemed "research-relevant". I agree that both the biological skills and coding skills are important, but in that sentence I am not sure why it's linked to the datatypes and packages."
    - Discussion of research relevant skills has been expanded to specify the demonstrated connection between learning to code and enhanced critical thinking: "MIGHTS demonstrates the use of many frequently used data types and packages for scRNA-seq analyses (Table 1), preparing users with research-relevant skills. Broadly applicable use of programming functions, algorithms, and troubleshooting lends itself to increased creative and critical thinking [42, 43]."

Reviewer 3:

16. "Intro: Give some information about the type of information these tutorial provide in the end: is it the growth rate for each cell type in the fetus?"
    - Description of the sample dataset and biological insights explored by the suite have been introduced earlier than in the original draft– emphasizing the kind of information and analysis skills the suite provides.
17. "Overall, add a little bit more high-level information about what each tutorial does, for people who are not already familiar with scRNA-seq."
    - All figures have been altered such that they are more legible and concise. Workflow figures for the tutorials have been edited to include pertinent information regarding the steps and accomplishments of the tutorial(s). Additionally, higher-level descriptions of the tutorial outcomes are described in each of their respective text introductions.
18. "The Single cell subpage contains more than the MIGHTS material, are they all supported the same way by the community with the same revision rate than described in table 3?"
    - All the material on the Single Cell subpage is maintained by the community to provide updated and well-functioning tutorials. Since the MIGHTS tutorials are designed as a suite, their average revision rate is different from standalone tutorials: "These tutorials are similarly

monitored and revised, although the rate of growth specifically for single-cell tutorials is noteworthy."

19. "In the Discussion: Do you have advice to give to people who want to develop similar material in their field?"
    - Broader discussion of the topics available on the Galaxy Training Network have been added such that readers may be directed to explore, and even contribute to, the growth of these resources across many applications of bioinformatics. Also, the resources to develop both training material as well as wrapping the tools have been mentioned: "If users wish to embrace programming such that they are looking to wrap their own tools and create training material based on them, resources and opportunities to do so exist on GTN pages dedicated to development in Galaxy [https://training.galaxyproject.org/training-material/topics/dev/] and contributing to the Galaxy Training Material [https://training.galaxyproject.org/training-material/topics/contributing/]"
20. "It would be nice to have separate learning paths for BB and PE, so that users who want to focus on developing one set of skill find them more easily."
    - Distinct learning pathways for BB and PE users have been additionally emphasized in the text and in reference to Figure 2.
    - Two distinct learning pathways have been created - one for BB, the other for PE and referenced in the Discussion: "To facilitate choosing the right starting point depending on the users' experience or skills to develop, single-cell-oriented Learning Pathways were introduced. "Applying single-cell RNA-seq analysis" [51] and "Applying single-cell RNA-seq analysis in Coding Environments" [52] pathways are based on BB and PE tutorials respectively and can be used for a smooth transition from button-based tutorials to programming environment (Figure 2A) or a direct start in the coding environment (Figure 2B)."
21. "It would be nice to be able to find this set of tutorials by searching MIGHTS in the GTN."
    - We have added a tag to each tutorial to allow for this searching, and noted this in the Discussion: "Additionally, to allow for easy searching for any of the described tutorials, each tutorial is has a tag and hence the user can simply enter "MIGHTS" in the GTN search box to get a list of the relevant materials."

Bioinformatic skills are highly sought after in the life sciences, yet the resources to learn them have yet to be optimized (Attwood et al., 2019). In this technical note we present a uniquely constructed suite of tutorials for single-cell RNA sequencing

analysis to bridge the gap from concepts to coding. The suite is unique in it's ability to also act as an introduction to coding bioinformatic analyses. The user tested workflows offering parallel tracks supporting programming fearful wet-lab scientists in becoming coding bioinformaticians.

As evidenced by the increased publication of scRNA-seq data globally (Lotfollahi et al., 2024), we believe that our tutorial suite should be published by *Gigascience* because it will appeal to the journal's target audience and support the growth of bioinformatics as a field.

We confirm that this work is original and has not been published elsewhere, nor is it currently under consideration for publication elsewhere.

We have no conflicts of interest to disclose, no AI-assisted technology used for manuscript drafting, and no other issues relating to journal policies.

All authors have approved the submission of this manuscript. Please address all correspondence regarding this manuscript to us at cgoclowski@genetics.utah.edu, j.jakiela@sms.ed.ac.uk, and wendi.bacon@open.ac.uk

Thank you for your consideration of this manuscript.

Sincerely,

Wendi Bacon, PhD; Camila Goclowski and Julia Jakiela (co-first authors)
