# Supplementary material for: Galaxy as a gateway to bioinformatics: Multi-Interface Galaxy Hands-on Training Suite (MIGHTS) for scRNA-seq
Source: Gigascience. 2025 Jan 8;14:giae107. doi: 10.1093/gigascience/giae107 (PMC11707610; doi:10.1093/gigascience/giae107)
Supplement: giae107_Response_to_Reviewer_Comments_Original_Submission [file giae107_response_to_reviewer_comments_original_submission.pdf]

# Author's Response To Reviewer Comments

Reviewer 1:

1. “While the manuscript lays emphasis on unstandardized and iterative analysis, the authors could draw a strong rationale on lack of reinstantiation methods which is certainly the need. The tools like Scanpy/Seurat and other ecosystem tools, there is a latency and they work differently.”

● In the discussion, reinstantiation has been emphasized, in connection to reproducibility, as an end goal accomplishment of the MIGHTS tutorial suite.

2. “Training-the-trainer is a very important entity as next generation sequencing (NGS) tools advances. A word or two on that would be a nice addition.”

● In the discussion, added “As sequencing strategies and tools advance, it is important that the field of bioinformatics “trains the trainer” in response to continued growth”.

3. “While programming based and button based entities are well taken, there could be a ‘programming a button’ section if not swapping between them. This will allow naive bioinformaticists to embrace programming. The authors may want to mention this.”
○ In the discussion, added a nod to the existing “How to wrap a galaxy tool” section of GTN Training Material: “If users wish to embrace programming such that they are looking to wrap their own tools and create training material based on them, resources and opportunities to do so exist on GTN pages dedicated to development in Galaxy [https://training.galaxyproject.org/training-material/topics/dev/] and contributing to the Galaxy Training Material [https://training.galaxyproject.org/training-material/topics/contributing/]”

4. “There could be an alternative annotation tool, viz. Annotationhub instead of Biosmart as well.”

● We thank the reviewers for this comment, however we cannot include every available tool in Galaxy, so unfortunately this is beyond the scope of the project. The authors agree that expanding annotation tool availability and programming environments would be beneficial. The Galaxy Training Network is committed to continued enhancement of tools to meet evolving user needs.

5. “Likewise vscode could be more inviting for some instead of jupyter IDE. The end user may be given a note in README file”
● Because this is training material, rather than traditional coding documentation, there is no README file associated with the training. VSCode is not traditionally used for training, as the Jupyter IDE includes both the coding environment and the space to run it. For this reason, we have not mentioned text editing software. However, after completing the MIGHTS tutorials, the users should feel comfortable enough in the programming environment to work in another IDE if they find it more inviting.

Reviewer 2:

We have addressed each of the following grammatical/spelling errors:
6. As access to computationally driven domains of biology continue[s] to grow
7. blending skills across disciplines is not without challenge[s]
8. "MIGHTS demonstrates the use of many frequently used data types and packages for scRNA-seq analyses (Table 2), preparing users with research[-]relevant skills."
9. "Workflows for each tutorial topic are shown -below- in Figure 4."
○ "Workflow is demonstrated -below- in Figure 8" (remove the belows)

We have addressed the following figure and table corrections:
10. “Figure 1 is too small to read, and it would be interesting to compare the BB method and the PE method to get the same images.”

○ Figure 1 has been edited to convey key concepts/steps in the tutorial. Relevant information from the original figure was converted to text from a screenshot so as to more uniformly present the arguments. The figure caption has also been altered to more directly compare BB and PE methods.
11. "Table 1 and 2 are redundant, use only table 2."
○ Tables 1 and 2 have been collapsed such that they do not present redundant information.
12. "Figure 4: Too small to see the stars.".
○ Figure 4 has been replaced with a more legible workflow diagram describing only the necessary details of the tutorials' workflows.
13. "Figure 4,5,6,7: Add the significance of colored boxes in the legend. (too small to read the box titles). Overall, these figures are hard to read and are difficult to link with the text. Maybe in the text about tutorials, mention which step corresponds to which box color, or move these figures to supplemental material with more detailed legends."
○ Figures 4,5,6,7,8 have been replaced with more legible workflow diagrams rather than images of the extracted Galaxy workflows. The updated diagrams visualize the high-level information and the tools used in each step of the analysis. The coloring of the boxes has been unified, for example the input files are now all shown in orange, output files in purple, plotting in cyan, marker genes in bright yellow. Galaxy-generated workflow images were included in the Supplementary Data to provide exhaustive details, such as all the output files generated by each tool and their formats.
14. "Figure 9: what does each letter correspond to? It looks like it is showing the same information than figure 1."
○ Figure 1 is intended to draw attention to the similarities in arguments across all four modes when plotting the expression of a gene of interest. Whereas Figure 9 demonstrates the consistency of clustering results observed across all four methods. The letters in the Figure 9 were removed since the image itself is self-explanatory, without the need to include the four panels A-D.
15. "'MIGHTS demonstrates the use of many frequently used data types and packages for scRNA-seq analyses (Table 2), preparing users with research relevant skills.' : Discuss a bit more which skills are deemed "research-relevant". I agree that both the biological skills and coding skills are important, but in that sentence I am not sure why it's linked to the datatypes and packages."
○ Discussion of research relevant skills has been expanded to specify the demonstrated connection between learning to code and enhanced critical thinking: "MIGHTS demonstrates the use of many frequently used data types and packages for scRNA-seq analyses (Table 1), preparing users with research-relevant skills. Broadly applicable use of programming functions, algorithms, and troubleshooting lends itself to increased creative and critical thinking [42, 43]."

Reviewer 3:

16. "Intro: Give some information about the type of information these tutorial provide in the end: is it the growth rate for each cell type in the fetus?"
○ Description of the sample dataset and biological insights explored by the suite have been introduced earlier than in the original draft–emphasizing the kind of information and analysis skills the suite provides.
17. "Overall, add a little bit more high-level information about what each tutorial does, for people who are not already familiar with scRNA-seq."
○ All figures have been altered such that they are more legible and concise. Workflow figures for the tutorials have been edited to include pertinent information regarding the steps and accomplishments of the tutorial(s). Additionally, higher-level descriptions of the tutorial outcomes are described in each of their respective text introductions.
18. "The Single cell subpage contains more than the MIGHTS material, are they all supported the same way by the community with the same revision rate than described in table 3?"
○ All the material on the Single Cell subpage is maintained by the community to provide updated and well-functioning tutorials. Since the MIGHTS tutorials are designed as a suite, their average revision rate is different from standalone tutorials: "These tutorials are similarly monitored and revised, although the rate of growth specifically for single-cell tutorials is noteworthy."
19. "In the Discussion: Do you have advice to give to people who want to develop similar material in their field?"
○ Broader discussion of the topics available on the Galaxy Training Network have been added such that readers may be directed to explore, and even contribute to, the growth of these resources across many applications of bioinformatics. Also, the resources to develop both training material as well as wrapping the

tools have been mentioned: “If users wish to embrace programming such that they are looking to wrap their own tools and create training material based on them, resources and opportunities to do so exist on GTN pages dedicated to development in Galaxy [https://training.galaxyproject.org/training-material/topics/dev/] and contributing to the Galaxy Training Material [https://training.galaxyproject.org/training-material/topics/contributing/]”

20. “It would be nice to have separate learning paths for BB and PE, so that users who want to focus on developing one set of skill find them more easily.”

○ Distinct learning pathways for BB and PE users have been additionally emphasized in the text and in reference to Figure 2.

○ Two distinct learning pathways have been created - one for BB, the other for PE and referenced in the Discussion: “To facilitate choosing the right starting point depending on the users’ experience or skills to develop, single-cell-oriented Learning Pathways were introduced. “Applying single-cell RNA-seq analysis” [51] and “Applying single-cell RNA-seq analysis in Coding Environments” [52] pathways are based on BB and PE tutorials respectively and can be used for a smooth transition from button-based tutorials to programming environment (Figure 2A) or a direct start in the coding environment (Figure 2B).”

21. “It would be nice to be able to find this set of tutorials by searching MIGHTS in the GTN.”

○ We have added a tag to each tutorial to allow for this searching, and noted this in the Discussion: “Additionally, to allow for easy searching for any of the described tutorials, each tutorial is has a tag and hence the user can simply enter “MIGHTS” in the GTN search box to get a list of the relevant materials.”
