# Supplementary material for: Galaxy as a gateway to bioinformatics: Multi-Interface Galaxy Hands-on Training Suite (MIGHTS) for scRNA-seq
Source: Gigascience. 2025 Jan 8;14:giae107. doi: 10.1093/gigascience/giae107 (PMC11707610; doi:10.1093/gigascience/giae107)
Supplement: giae107_Reviewer_1_Report_Original_Submission — Prashanth Suravajhala -- 9/25/2024 Reviewed [file giae107_reviewer_1_report_original_submission.pdf]

**Reviewer Report**

**Title: Galaxy as a Gateway to Bioinformatics: Multi-Interface Galaxy Hands-on Training Suite (MIGHTS) for scRNA-seq**

**Version: Original Submission** **Date:** 9/25/2024

**Reviewer name: Prashanth Suravajhala**

**Reviewer Comments to Author:**

Gist/Summary: The authors propose a Multi-Interface Galaxy Hands-on Training Suite (MIGHTS) for single cell RNA-seq ( ScRNA-seq) analysis which helps coders and as well beginners to adapt to it easily! The proposal comes up with a strong rationale mentioning the need for enhancing and developing unstandardized pipelines/iterative analysis. The authors describe the manuscript with pictorial methodology, workflows and ample datasets as an example that showcases tutorials, slide deck, video recordings, etc.. The manuscript is generally written very well and is a welcome article for the bioinformatics community in general.While the manuscript lays emphasis on unstandardized and iterative analysis, the authors could draw a strong rationale on lack of reinstantiation methods which is certainly the need. The tools like Scanpy/Seurat and other ecosystem tools, there is a latency and they work differently.Training-the-trainer is a very important entity as next generation sequencing (NGS) tools advances. A word or two on that would be a nice addition.While programming based and button based entities are well taken, there could be a "programming a button" section if not swapping between them. This will allow naive bioinformaticists to embrace programming. The authors may want to mention this.There could be an alternative annotation tool, viz. Annotationhub instead of Biosmart as well.Likewise vscode could be more inviting for some instead of jupyter IDE. The end user may be given a note in README fileScores on a scale of 0-5 with 5 being the bestLanguage: 4.75Novelty: 4Brevity: 4Scope and relevance: 4.5

**Level of Interest**

Please indicate how interesting you found the manuscript: Choose an item.

**Quality of Written English**

Please indicate the quality of language in the manuscript: Choose an item.

**Declaration of Competing Interests**

Please complete a declaration of competing interests, considering the following questions:

- Have you in the past five years received reimbursements, fees, funding, or salary from an organisation that may in any way gain or lose financially from the publication of this manuscript, either now or in the future?

- Do you hold any stocks or shares in an organisation that may in any way gain or lose financially from the publication of this manuscript, either now or in the future?
- Do you hold or are you currently applying for any patents relating to the content of the manuscript?
- Have you received reimbursements, fees, funding, or salary from an organization that holds or has applied for patents relating to the content of the manuscript?
- Do you have any other financial competing interests?
- Do you have any non-financial competing interests in relation to this paper?

If you can answer no to all of the above, write 'I declare that I have no competing interests' below. If your reply is yes to any, please give details below.

I have no competing interests!

I agree to the open peer review policy of the journal. I understand that my name will be included on my report to the authors and, if the manuscript is accepted for publication, my named report including any attachments I upload will be posted on the website along with the authors' responses. I agree for my report to be made available under an Open Access Creative Commons CC-BY license (http://creativecommons.org/licenses/by/4.0/). I understand that any comments which I do not wish to be included in my named report can be included as confidential comments to the editors, which will not be published.

Choose an item.

To further support our reviewers, we have joined with Publons, where you can gain additional credit to further highlight your hard work (see: https://publons.com/journal/530/gigascience). On publication of this paper, your review will be automatically added to Publons, you can then choose whether or not to claim your Publons credit. I understand this statement.

Yes Choose an item.
