# Supplementary material for: Galaxy as a gateway to bioinformatics: Multi-Interface Galaxy Hands-on Training Suite (MIGHTS) for scRNA-seq
Source: Gigascience. 2025 Jan 8;14:giae107. doi: 10.1093/gigascience/giae107 (PMC11707610; doi:10.1093/gigascience/giae107)
Supplement: giae107_Reviewer_2_Report_Original_Submission — Delphine Lariviere -- 9/29/2024 Reviewed [file giae107_reviewer_2_report_original_submission.pdf]

Reviewer Report

Title: Galaxy as a Gateway to Bioinformatics: Multi-Interface Galaxy Hands-on Training Suite (MIGHTS) for scRNA-seq

Version: Original Submission **Date:** 9/29/2024

Reviewer name: Delphine Lariviere

Reviewer Comments to Author:

The authors present a set of training materials in the Galaxy Training Network dedicated to scRNA-seq, They provide button based and programming based material to suit different level of skill and goals. This is a great work of making bioinformatic analyses accessible to everyone, regardless of learning trajectories.SpellingAs access to computationally driven domains of biology continue[s] to growblending skills across disciplines is not without challenge[s]"MIGHTS demonstrates the use of many frequently used data types and packages for scRNA-seq analyses (Table 2), preparing users with research[-]relevant skills.""Workflows for each tutorial topic are shown -below- in Figure 4.""Workflow is demonstrated -below- in Figure 8" (remove the belows)Figures and tables1. Figure 1 is too small to read, and it would be interesting to compare the BB method and the PE method to get the same images.2. Table 1 and 2 are redundant, use only table 2.3. Figure 4: Too small to see the stars.4. Figure 4,5,6,7: Add the significance of colored boxes in the legend. (too small to read the box titles). Overall, these figures are hard to read and are difficult to link with the text. Maybe in the text about tutorials, mention which step corresponds to which box color, or move these figure to supplemental material with more detailed legends.5. Figure 9: what does each letter correspond to? It looks like it is showing the same information than figure 1.Text1. "MIGHTS demonstrates the use of many frequently used data types and packages for scRNA-seq analyses (Table 2), preparing users with research relevant skills. " : Discuss a bit more which skills are deemed "research-relevant". I agree that both the biological skills and coding skills are important, but in that sentence I am not sure why it's linked to the datatypes and packages.2. Tutorials:- Intro: Give some information about the type of information these tutorial provide in the end: is it the growth rate for each cell type in the fetus? What are the main steps that each tutorial provide?- Overall, add a little bit more high-level information about what each tutorial does, for people who are not already familiar with scRNA-seq.3. It would be nice to be able to find this set of tutorials by searching MIGHTS in the GTN.4. The Single cell subpage contains more than the MIGHTS material, are they all supported the same way by the community with the same revision rate than described in table 3?5. It would be nice to have separate learning paths for BB and PE, so that users who want to focus on developing one set of skill find them more easily.6. In the Discussion: Do you have advice to give to people who want to develop similar material in their field?

Level of Interest

Please indicate how interesting you found the manuscript: Choose an item.

Quality of Written English

Please indicate the quality of language in the manuscript: Choose an item.

Declaration of Competing Interests

Please complete a declaration of competing interests, considering the following questions:

- Have you in the past five years received reimbursements, fees, funding, or salary from an organisation that may in any way gain or lose financially from the publication of this manuscript, either now or in the future?
- Do you hold any stocks or shares in an organisation that may in any way gain or lose financially from the publication of this manuscript, either now or in the future?
- Do you hold or are you currently applying for any patents relating to the content of the manuscript?
- Have you received reimbursements, fees, funding, or salary from an organization that holds or has applied for patents relating to the content of the manuscript?
- Do you have any other financial competing interests?
- Do you have any non-financial competing interests in relation to this paper?

If you can answer no to all of the above, write 'I declare that I have no competing interests' below. If your reply is yes to any, please give details below.

I declare that I have no competing interests

I agree to the open peer review policy of the journal. I understand that my name will be included on my report to the authors and, if the manuscript is accepted for publication, my named report including any attachments I upload will be posted on the website along with the authors' responses. I agree for my report to be made available under an Open Access Creative Commons CC-BY license (http://creativecommons.org/licenses/by/4.0/). I understand that any comments which I do not wish to be included in my named report can be included as confidential comments to the editors, which will not be published.

Choose an item.

To further support our reviewers, we have joined with Publons, where you can gain additional credit to further highlight your hard work (see: https://publons.com/journal/530/gigascience). On publication of this paper, your review will be automatically added to Publons, you can then choose whether or not to claim your Publons credit. I understand this statement.

Yes Choose an item.
